# Documentation system for plant transformation service and research

**DOI:** 10.1186/1746-4811-6-4

**Published:** 2010-01-27

**Authors:** Karin I Köhl, Jürgen Gremmels

**Affiliations:** 1 Max-Planck-Institute of Molecular Plant Physiology, Am Muehlenberg 1, 14476 Golm, Germany

## Abstract

**Background:**

In plant transformation, method compliance is critical for success. Transformation methods are complicated and tend to evolve over time. Until the complete method is published, method details are often partially orally transmitted and thus bound to a few people. Their documentation in text files are often a mixture of material and method description with many references to other sources especially to media description. These media are complex and often composed from several commercially available mixtures plus individually prepared stocks. The actual transformation experiment is generally documented in lab books, in which deviations from the methods and results are reported. Additionally, work schedules are planned in diaries. Both paper-based sources lack backup copies and miss unambiguous links to method descriptions and media recipes.

**Description:**

To solve the problem, we devised a standard-operation-procedure system based on a Microsoft Access database containing the interlinked modules '*Media'*, '*Methods' *and '*Experiments'*. The *Media *module contains all basic chemicals, stocks and complex media. In this module, complex media are composed from other elements of the *Media *module, thus mimicking the workflows of media preparation in the lab. The *Media *module is made attractive to the user by functions that generate file cards and labels. The *Methods *module describes each method stepwise and links the steps to the media. Copy functions allow cloning of old methods to document method evolution without alteration of the old methods. Activation and inactivation functions in the *Media *and the *Methods *module remove outdated entries from active use. The *Experiments *module links the method to experiment specific information. This module generates a lab-book like user interface and a work schedule, and it contains a simple result section.

**Conclusion:**

The system has been evolved and tested over several years in a transformation service unit, where it increased efficiency. Additionally, the system provided rapid access to data for quality control and decision making. The system can be easily modified for the use in other research environments.

## Background

In tissue-culture-based generation of genetically modified plants, minute method compliance is critical for success. Plant transformation methods are complex multi-step methods that incorporate parts of older methods (bits that worked) and thus contain references to older sources. In published methods, this results in a chain of cross-references. For example, a modern rice transformation method ([1] references to [2,3] and [4], which references to [5]). The problem is not tissue-culture specific, but can be found in many other research fields as well, to the inconvenience of the reader trying to reproduce a method.

In laboratory practice, methods are often documented on paper or in text files. The link between methods is ambiguously provided by method names and often orally transmitted. The method text generally contains both, the method description and (some) information on media composition as well as cross-references to media described elsewhere. The cross-reference is again done by media names. Media names tend to change, usually because their initial name becomes ambiguous or is too long for practical use. Thus, the same medium may have different names sometimes even in the same lab. Furthermore, tissue culture media are often very complex with 20 and more different compounds. They are thus often composed stepwise from stocks and mixtures of stocks, including commercially available mixtures. The source of the chemicals, the preparation procedure of the stocks and the complex mixtures are deemed important to the efficiency of the transformation experiment. Efficiency results from the required man power and the success rate. To increase efficiency, media and methods are improved over time. Methods are furthermore changed to adapt them to different cultivars. During these evolutionary processes, the original method can be easily lost. Thus, the reproducibility of plant transformation experiments is in jeopardy, especially if the method is unpublished, has not been used for some time or has been developed by a researcher who left the laboratory.

To address the problem, we designed a computer-based documentation system that interlinks media, methods and actual experiments unambiguously. To make its use attractive to the lab staff, we provided reporting tools that produce stock labels and 'file cards' with media description. The reporting tool additionally generates work schedules for the current experiments. The system was established and tested in a service unit that generates transgenic plants for researchers.

## Construction and content

The database system consists of the three modules *Media*, *Methods *and *Experiments *that represent the workflows preparation and storage of media, standard operation procedures (methods) and the conduction of experiments. The start page of the database (additional file 1, for readers without access to MS Access: additional file 2: SupplementaryFigures - 'Startpage') provides direct access to the most important functions of these three modules.

## Media module

**Figure 1:**
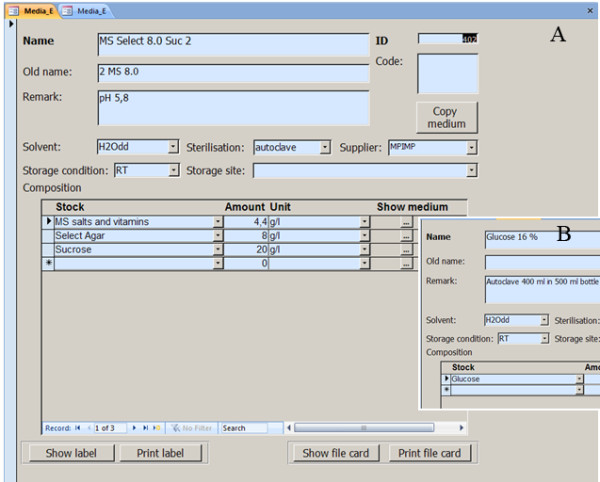
***Media *form**. The form contains unique name and id of the medium, preparation and storage information (A). The graphic entry field 'Code' contains color codes for plates. A medium can be composed from many compounds that can be selected from the list of existing media. Basic compounds purchased from external suppliers do not contain any further compounds. The 'Show media' button of each compound displays the composition of the selected compound (B). The 'print label' function prints labels on a label printer, the function 'Print file card' prints a report on a standard (A4) printer. The background color of the entry fields indicates the status of the medium (white = new, blue = approved, grey = obsolete).

The *Media *module contains information on all basic chemicals, stocks and complex media. Data entry into the module is performed on forms (Figure 1), for which customized versions (different languages, personalized forms) can be generated easily without programming skills (s. additional file 1: Method form without (Media_E_1) or with datasheet view (Media_E_2), alternatively additional file 2: Media_E_1 or Media_E_2).

On entry, each medium receives a unique media id and a unique name. For each medium, information on preparation (remark, solvents, and sterilization) and storage conditions are provided. On the form, media can be generated by selecting other media or stocks from the Media module. For example, the medium in Figure 1 is generated from three basic compounds. The complex medium Id 404 (see additional file 1, alternatively additional file 2: Media_E_1) is generated from other media and stocks. Stocks reference back to one basic chemical (see additional file 1: Glucose 16%, Id 411 alternatively additional file 2: Media_E_1, Id 411). Basic chemicals or ready-made mixtures purchased from suppliers do not reference back to any other media entry (Example Glucose Id 413 alternatively additional file 2: Media_E_1, Id 413). With this 'self-referencing', the Media module mimics the workflow of media preparation in the lab. The referencing between the media is based on the unique media id. This system allows editing of media names to correct typos. Furthermore, multi-language versions can be generated without losing the connection between media or to the Methods module.

To facilitate lab work and thus make the system more attractive to users, file cards and labels are generated automatically (Figure 1). File cards contain all information needed to make up the media in the lab including the name, id and the correct storage condition. Autoclave-proof labels display the id and the name of the medium. The print-out date on the label facilitates tracking the age of stocks.

Media entries can be put into three different stages by the scientists responsible for the database (so-called 'administrator'). Administrator functions are accessible on in the experts' module (additional file 1, alternatively additional file 2: 'expertsmodule'). The status of a medium is depicted by the background color of the entry fields. *New *media entries can be edited by all users (white background). As soon an approved date has been entered by the administrator (into the form media experts), the background turns blue. Then, changes to the media are no longer possible to avoid accidental changes by a user. When a date has been entered in the 'obsolete' field (grey background) the user receives a warning when he tries to choose the outdated medium to compose a new medium.

## Methods module

**Figure 2:**
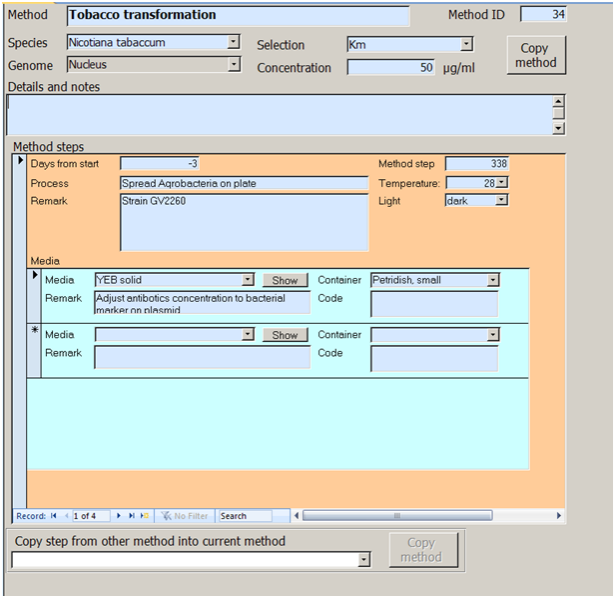
***Method *form**. The form contains unique name and id of the method, information on the species, on which it can be used, and the selection. The method is divided in distinct steps that are performed at different times. Media can be linked to each step by choosing them from the media list. The function 'copy method' copies the entire method including all steps and media linked to them. The function 'Copy step' allows copying a step from another method into a method.

The *Methods *module describes each method stepwise (Figure 2, additional file 1: Method form with (Method form_E_1) or without datasheet view (Method_form E_2), alternatively additional file 2: 'Method form_E_1'). Each step represents a logical unit performed at a time. The information when a step is to be performed is given relative to the beginning of the experiment. In our example, the start was defined as the transformation date. This method allows calculating the real date for each step of an experiment (s. '*Experiment *module') based on the experiment-specific transformation date. Each step links to the media required for this step and the recommended containers (tubes, plates). Furthermore, it lists incubation conditions.

To facilitate the documentation of new methods, two copy functions were designed. The 'copy method' function copies the entire method including all steps. For each step, it copies all media linked to it. The second copy function ('copy step') allows copying single steps from existing methods into a new method to build new methods from well-established parts of other methods. Both copy functions assigns new ids and new names to the method and each step. Modified methods can thus be generated from existing methods by altering a few details like the incubation conditions or the media composition. If the new method replaces the old method, the administrator enters the 'obsolete' date for the old method (see additional file 1 Form 'Methods Experts', alternatively additional file 2: 'MethodsExperts'). Outdated methods remain in the actual database and are available for referencing. Thus, information on experiments performed with older methods remains unaltered.

## Experiments module

**Figure 3:**
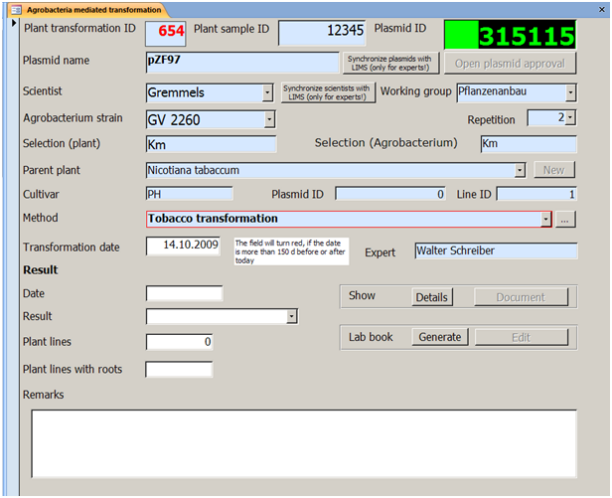
***Experiment *form**. The form for the documentation of plant transformations collects the information on the parent plant and the plasmid used for a transformation experiment. The plasmid identifier links to a plasmid table, in which relevant details (e.g. resistance marker genes, approval status) are documented. The approval status of the construct is displayed as color code. The experiment is started by choosing a method from the method list and entering the start date. Results are entered in the lower section of the form. 'Details' generates a schedule for the experiment with a short method description. 'Document' generates the final filing document. For 'Lab book' function see Figure 4.

The *Experiments *module contains the information on an actual experiment, in our case a plant transformation, and links it to the method (Form see Figure 3). The basic information for a transformation experiment is the transformation date, the plasmid information and the details on the parent plant that is to be transformed.

The plasmid information is stored in a separate table (construct, see additional file 1: Experts' module; alternatively additional file 2: 'Construct'). Plasmid information links to the experiment by the plasmid id. Information relevant for the experiment (namely plasmid name and selection system) is displayed on the experiment form but cannot be changed there. The plasmid entries can be approved by an expert, when the plasmid check was positive. Depending on the status of the plasmid, the plasmid id field is red (not approved) or green (approved). The plasmid information can be entered and changed in the table construct that is accessible from the experts' page. In our case, the plasmid information is read from an Oracle database of a laboratory information management system (LIMS) to save the time for double entries in both systems.

The details on the parent plants are stored in a separate parent table, from which they can be selected on the experiment form. Again, relevant information like species and variety is displayed on the experiment form, but can only be altered in the parent table (experts module screen: Parents experts).

Depending on the transformation method, namely ballistic or *Agrobacterium tumefaciens *mediated transformation, different additional information is required. These differences are catered for by designing modified forms that feed into the same table. The form for ballistic transformation requests information on the plasmid concentration and the cannon used (see additional file 1, 'Enter Ballistic transformation', alternatively additional file 2: 'BallisticTransformation'). The Experiment form for Agrobacterium mediated transformation (Figure 3) collects the name of the Agrobacterium strain. On each form, only the respective methods for ballistic or Agrobacterium mediated transformation are displayed. As transformations are often performed by specialized researchers and technicians (experts) for other researchers (scientist), information on both experts and scientists are requested in the form.

**Figure 4:**
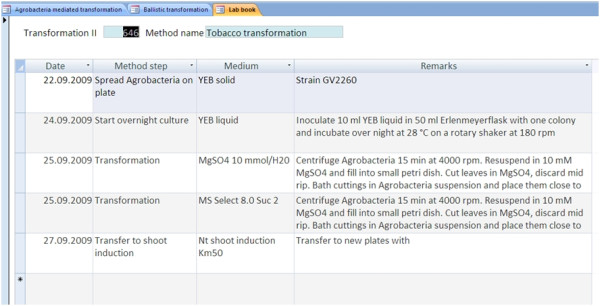
**Lab book**. For each transformation experiment, a laboratory book can be generated once by activating the function 'Lab book'. The function calculates the due dates for each step from the start date and the method. Deviations with respect to timing and media can be entered without changing the original method.

When a transformation experiment is started, the method is selected and the transformation date entered. Now, a method report containing the real due-dates for each step and a short description can be generated (function 'Details'). The function 'Generate' produces a laboratory book (Figure 4); this function can be run only once on each experiment. In this computer-based lab book, the most frequent deviations from standard methods namely date and media can be altered without changing the description of the original method. The text field 'Remarks' provides space to describe further method deviations or observations.

Work schedules for the next 9 or 31 days are automatically generated for all active experiments (s. additional file 1, Work overview 'Next 31 days', alternatively additional file 2: 'Next 31 days'). The due dates are calculated from the transformation date and the selected method. For the schedules, the due dates are filtered for the time between today and today + 9 (31) days. For each day, the ids of the transformations that are due for processing and a short description of each step are listed (Table 1).

**Table 1:** Work schedule shows an example of an automatically generated work schedule listing the work for the next nine days.

ID	Date	CV	Step	Method		Selection	Plasmid Id	Transformation date
**590**	15.09.2009	MM	Hand over	Tomatotransformation_Blau		Hyg	312121	17.06.2009
**628**	16.09.2009	IPA-6	Transfer Shoot induction	Tomatotransformation_IPA6		Km	167	19.08.2009
**618**	16.09.2009	PH	Transfer Shoot induction	Tobaccotransformation Kanamycin		Km	309084	28.07.2009
**617**	16.09.2009	SNN	Exchange medium	Tobaccotransformation Hygromycin		Hyg	314778	28.07.2009
**619**	18.09.2009	MM	Transfer Shoot induction	Tomatotransformation_Blau		Hyg	312853	29.07.2009
**614**	18.09.2009	MM	Transfer Shoot induction	Tomatotransformation_Blau		Hyg	312852	29.07.2009
**635**	20.09.2009	MM	Transfer Shoot induction	Tomatotransformation_Blau		Hyg	312121	14.08.2009
**634**	20.09.2009	MM	Transfer Shoot induction	Tomatotransformation_Blau		Km	305498	14.08.2009
**633**	23.09.2009	SNN	Transfer Shoot induction	Tobaccotransformation Hygromycin		Hyg	315635	14.08.2009
**643**	24.09.2009	PH	Transfer Shoot induction	Tobaccotransformation Hygromycin		Hyg	315285	04.09.2009

(ID = Experiment identifier, CV Cultivar)

The end of the experiment is documented by keywords (handover, withdrawn, contaminated) and, if successful, with the number of selected plants. Finished experiments are withdrawn from the work schedule. From these data, reports on success rates and failure reasons can be easily generated (example see Table 2).

**Table 2:** Evaluation example shows success rates and failure rates for the transformation of different species in four years.

Species	Year	Plasmids	Sum of independent lines	Lines per transformation	
				Min	Max
Lycopersicon esculentum	2005	34	284	0	65
Lycopersicon esculentum	2006	18	102	0	25
Lycopersicon esculentum	2007	40	226	0	48
Lycopersicon esculentum	2008	31	433	0	61
Nicotiana tabaccum	2005	13	484	0	135
Nicotiana tabaccum	2006	64	1452	0	130
Nicotiana tabaccum	2007	54	2329	0	111
Nicotiana tabaccum	2008	14	396	0	102
Oryza sativa	2005	3	0	0	0
Oryza sativa	2006	56	23	0	15
Oryza sativa	2007	40	51	0	22
Oryza sativa	2008	27	7	0	6
Solanum tuberosum	2005	21	978	0	91
Solanum tuberosum	2006	3	94	0	48
Solanum tuberosum	2007	4	52	8	22
Solanum tuberosum	2008	5	238	13	77

## Utility and discussion

In large research institutions and academic communities, documentation of resources and results in databases is state of the art. Database systems are used to manage genetic resources ([6-10]). Databases make scientific results available to the scientific community, especially in the areas of transcriptomics [11], proteomics [12] and metabolomics [13]. The general availability of these data in standardized formats enable derived knowledge and integrative approaches [14-17]. The big exception from standardized formats is the documentation of experimental methods that link the genetic resources with the result and the knowledge. Well established, reliably repeatable methods - so-called standard operation procedures (SOPs) - are a valuable part of the success of any research institution. SOPs are, however, mainly documented in text style as part of a publication or - in case of method evolution - several publications linked by references. Alternatively, methods are made available as text files on web sites [18,19]. There are academic approaches to document SOPs in databases [20,21]. In these databases, text files with the method description are stored and linked to the result. As pointed out in the introduction, text-based documents have their drawbacks. Some methods are better stored in a database, especially those that involve complicated media preparation or many steps performed over a long period of time. Most users are, however, more familiar with word processing programs than databases. The 'interface' problem can be solved by so-called laboratory information management systems (LIMS). A LIMS provides a user-friendly graphic user interface to a database. In these systems, methods can be modeled as workflows. However, commercial LIMS systems are unavailable for most academic institutions, because of the considerable investment to set up such a system and to keep it running. For transcript profiling, an excellent open-source database system allows to document parameters of transcript profiling experiments [22]. For plant transformation, we devised a simple system to store standard operation procedures. The system is based on the widespread database system MS-Access and thus can be adjusted to specific requirements with a minimum of programming knowledge.

One of important features of our system is the 'self-referencing' *Media *module that allows generating complex media stepwise from more simple stock solutions. Thus, the entire process of making media from - at the bottom - commercially available compounds is completely and easily documented. The second important feature of the *Media *module is the reporting tool that provides printouts. These are indispensable in daily work at the bench, as the computer screen is usually not close to the lab bench. The database-generated printouts also facilitate the identification of the right medium for a step of a method by the matching name and identifier number (id) in the method description and on the media container. The identifier does not only increase safety by double-coding but also makes multi-language versions of the method much easier. This is especially important in international labs, in which English speaking scientist cooperate with local staff less fluent in English. Media and method printouts are admittedly also risky, as it is tempting to document changes in the material or method on the printout. Thus, staff needs to be trained to use the database system as the primary data source and document any deviation from the standard in the database.

An important feature of the system is its ability to cope with media and method evolution. If the deviation from the standard becomes a new standard, the new medium or method can be easily documented by duplicating the old method. The administrator in charge of the database can then approve of the new method and prevent its further alteration. At the same time, old methods can be taken out of use, but remain available for cross referencing.

The *Experiment *module of our SOP system is the module, in which the user can document deviations from the standard. This module automatically produces the plan for the experiment from the standard method description and generates an electronic lab book. In this lab book, any deviation from the plan concerning timing, media or method can be documented. For long-term experiments, the automatic generation of work schedules is another comfortable asset of the system. The work schedule allows early detection of work-peaks and facilitates hand-over of work between technical staff for times of absence.

In the beginning, the SOP system was run as a standalone solution at the MPI-MP and all data were entered directly. However, some of the information required in an experiment e.g. on plasmids and users were also present in the LIMS plant database system of the institute [10], which resulted in double entries. To save time, we devised a read access for expert users. The read function imports the information needed in the transformation process from the LIMS into the SOP database.

Finally, the system documents the result (number of lines) of the experiment. Failures are documented in a standardized way, thus problems are detected in an early state. Thus, data are rapidly available for decision making, e.g. about the stop of a method because of low efficiency.

## Conclusion

We constructed a database system to document media, methods and experiments in a transformation service unit. The system increased efficiency and reduced the risk of information loss when coworkers leave. Additionally, the system provided rapid access to data for quality control and decision making. Altogether, the introduction of the system was thus worth the effort. The system can be easily modified for the use in other research environments.

## Availability and requirements

The documentation system was first implemented in MS Access 2003 (Microsoft) and subsequently upgraded to MS Access 2007. A MS Access 2003 version is available as additional file 1 (transformation2003.mdb). A short introduction is given in additional file 3: readme. The database scheme can be displayed with the Access 'relationship' function. For readers without access to the program MS Access, the entity relation diagram (additional file 4: ERdiagramTransformation2003.pdf) and the database definition file (additional file 5: Object Definition for Transformation2003.pdf) are provided as supplemental material.

The actively used 'live' database is stored on a shared folder of the MS Windows XP operation system. User access is regulated by the security settings to the folder. Developments on the system are performed in a separate database and subsequently imported into the live database. Backup copies are generated every ten minutes based on the Windows XP backup system. Backup copies to an independent storage system are produced every night.

For less experienced users, an entry page and MS Access custom groups were generated. The entry page provides access to the most important features of the system. The custom groups in the example database are 'Favorites', 'Media module', 'Methods module', 'Experiments module', 'Experts module' and 'Experts module administration'. The custom groups contain links to the database objects relevant to the respective subject and thus facilitate work.

Autoclave-proof labels are printed on a Datamax E4304 label printer (AISCI, Bad Salzuflen, Germany).

## Abbreviations

LIMS: laboratory information management system; SOP: standard operation procedure.

## Competing interests

The authors declare that they have no competing interests.

## Authors' contributions

KK designed the database scheme, implemented the database, tested the live system and wrote the manuscript. JG programmed the visual basic code, suggested improvements and implemented the printer system. All authors read and approved the final manuscript.

## Supplementary Material

Additional file 1 **MSTransformation2003**. MS Access 2003 file, enable macros for full functionality. Click here for file

Additional file 2 **SupplementaryFigures**. The file contains pdf-files with screenshots on various forms of MSTransformation2003 to enable readers without access to MS-Access to view the forms. The content of each screenshot is addressed in the manuscript. Click here for file

Additional file 3 **Readme**. The file contains a short introduction to the system. It is assumed that the user is generally familiar with the software MS-Access. Thus, the introduction repeats only the most important features in handling the software and otherwise restricts itself to the features specific to our database. Click here for file

Additional file 4 **ERdiagramTransformation2003**. The file contains the entity relationship diagram for the database MSTransformation2003.mdb. Click here for file

Additional file 5 **Object Definition for Transformation2003.pdf**. The file contains the object definition for the database MSTransformation2003.mdb Click here for file
