# Supplementary material for: Documentation system for plant transformation service and research
Source: Plant Methods. 2010 Jan 27;6:4. doi: 10.1186/1746-4811-6-4 (PMC2835674; doi:10.1186/1746-4811-6-4)
Supplement: Additional file 2 — SupplementaryFigures. The file contains pdf-files with screenshots on various forms of MSTransformation2003 to enable readers without access to MS-Access to view the forms. The content of each screenshot is addressed in the manuscript. [file 1746-4811-6-4-S2.ZIP › AgrobacteriaMediatedTransformation.pdf]

657

0

315995

2150-GFP

Synchronize plasmids with LIMS (only for experts!)

Open plasmid approval

Koehl

Synchronize scientists with LIMS (only for experts!)

Pflanzenanbau

GV 2260

1

Km

Spec.

Nicotiana tabaccum

New

SNN

0

1

Tobacco transformation

...

Transformation date

24.12.2009

Brigitte Buchwald

Details

Document

0

Generate

Edit

658 | 0 | **315861**

pRC4334 | Synchronize plasmids with LIMS (only for experts!) | Open plasmid approval

Gremmels | Synchronize scientists with LIMS (only for experts!) | Pflanzenanbau

GV 2260 | 1

Km | Spec.

Nicotiana tabaccum | New

SNN | 0 | 1

Tobacco transformation | ...

Transformation date | 21.12.2009 | Brigitte Buchwald

Details

Document

0

Generate

Edit
