# Supplementary material for: Documentation system for plant transformation service and research
Source: Plant Methods. 2010 Jan 27;6:4. doi: 10.1186/1746-4811-6-4 (PMC2835674; doi:10.1186/1746-4811-6-4)
Supplement: Additional file 2 — SupplementaryFigures. The file contains pdf-files with screenshots on various forms of MSTransformation2003 to enable readers without access to MS-Access to view the forms. The content of each screenshot is addressed in the manuscript. [file 1746-4811-6-4-S2.ZIP › BallisticTransformation.pdf]

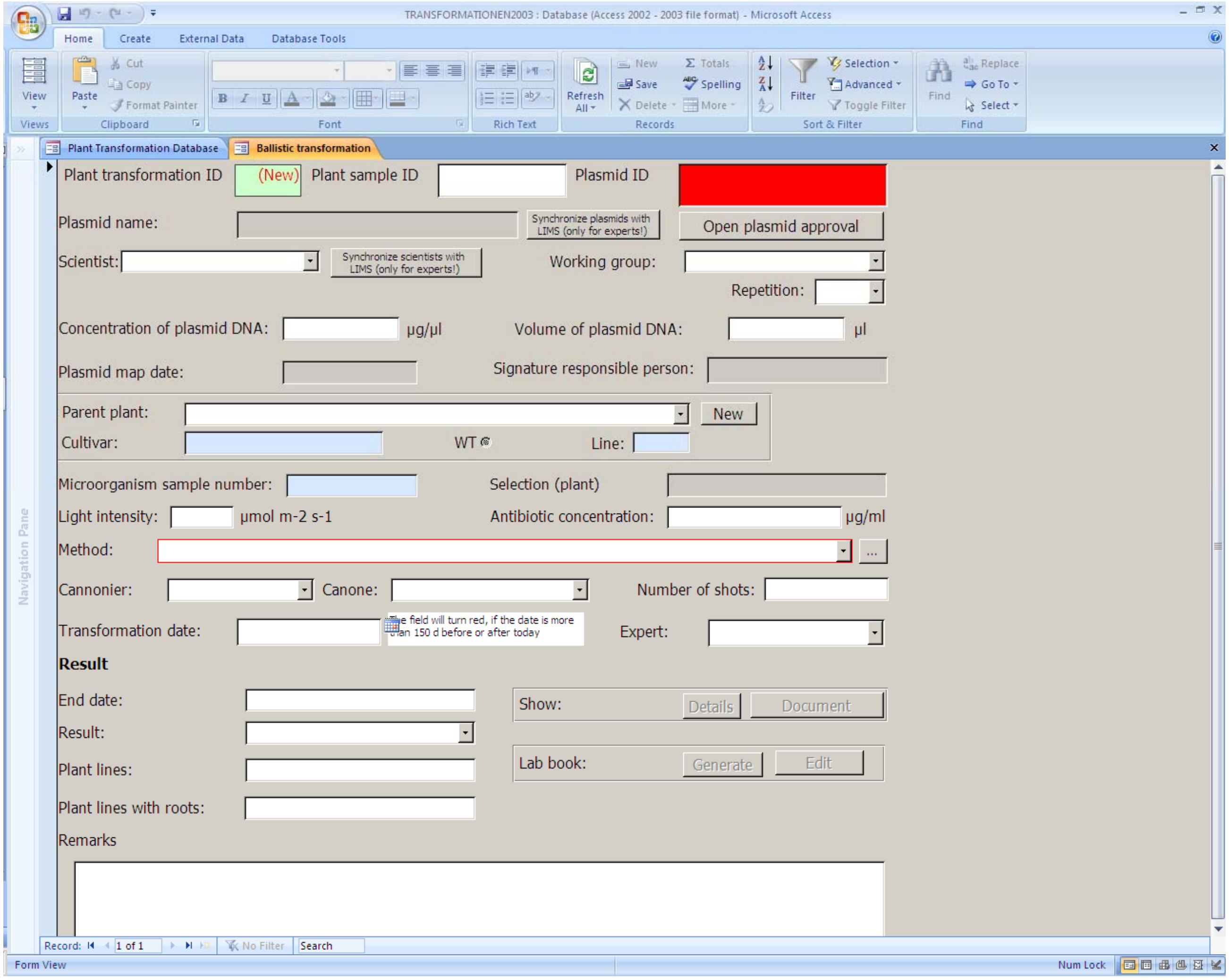
TRANSFORMATIONEN2003 : Database (Access 2002 - 2003 file format) - Microsoft Access
Home
Create
External Data
Database Tools
View
Paste
Cut
Copy
Format Painter
Views
Clipboard
Font
Rich Text
Refresh All
New
Save
Delete
Totals
Spelling
More
Records
Filter
Selection
Advanced
Toggle Filter
Sort & Filter
Find
Replace
Go To
Select
Find
Plant Transformation Database
Ballistic transformation
Plant transformation ID
(New)
Plant sample ID
Plasmid ID
Plasmid name:
Synchronize plasmids with LIMS (only for experts!)
Open plasmid approval
Scientist:
Synchronize scientists with LIMS (only for experts!)
Working group:
Repetition:
Concentration of plasmid DNA:
µg/µl
Volume of plasmid DNA:
µl
Plasmid map date:
Signature responsible person:
Parent plant:
New
Cultivar:
WT
Line:
Microorganism sample number:
Selection (plant)
Light intensity:
µmol m-2 s-1
Antibiotic concentration:
µg/ml
Method:
...
Cannonier:
Canone:
Number of shots:
Transformation date:
The field will turn red, if the date is more than 150 d before or after today
Expert:
Result
End date:
Show:
Details
Document
Result:
Plant lines:
Lab book:
Generate
Edit
Plant lines with roots:
Remarks
Navigation Pane
Record: 1 of 1
No Filter
Search
Form View
Num Lock
