# Supplementary material for: Documentation system for plant transformation service and research
Source: Plant Methods. 2010 Jan 27;6:4. doi: 10.1186/1746-4811-6-4 (PMC2835674; doi:10.1186/1746-4811-6-4)
Supplement: Additional file 2 — SupplementaryFigures. The file contains pdf-files with screenshots on various forms of MSTransformation2003 to enable readers without access to MS-Access to view the forms. The content of each screenshot is addressed in the manuscript. [file 1746-4811-6-4-S2.ZIP › Construct.pdf]

ConstructId	ConstructName	M Resistance_text	PlantResistance_text
315861	pRC4334	Spectinomycin	Kanamycin
315875	pKDftrgr	Kan, Strep/Spec	Kanamycin
315876	Xcs-abd	Amp, Kan	BASTA_Phosphinotrycin
315895	COE3g34545	Kanamycin	Hygromycin
315896	COE4g34534	Kanamycin	Hygromycin
315915	gmiRNA At5g3	Kanamycin	Kanamycin
315916	tmiRNA At	Kanamycin	Kanamycin
315935	35S::Lfff	Streptomycin/Spectino	Kanamycin
315936	RNAt	Spectinomycin	Kanamycin
315937	RNAt4g	Spectinomycin	Kanamycin
315955	pSA21	Amp, Spec	
315956	pSA22	Amp, Spec	Spectinomycin
315975	amiRNA ginger	Kanamycin	Kanamycin
315995	2150-GFP	Spectinomycin	Kanamycin
316015	56 GLN1;4 marta HA Strep	Ampicilllin	BASTA_Phosphinotrycin
316035	pOpoff2k_fuzzy	Spectinomycin	Kanamycin

M Resistance	Plant Resistance
4	3
12	3
7	1
3	2
3	2
3	3
3	3
6	3
4	3
4	3
8	
8	4
3	3
4	3
1	1
4	3
