# Supplementary material for: Documentation system for plant transformation service and research
Source: Plant Methods. 2010 Jan 27;6:4. doi: 10.1186/1746-4811-6-4 (PMC2835674; doi:10.1186/1746-4811-6-4)
Supplement: Additional file 2 — SupplementaryFigures. The file contains pdf-files with screenshots on various forms of MSTransformation2003 to enable readers without access to MS-Access to view the forms. The content of each screenshot is addressed in the manuscript. [file 1746-4811-6-4-S2.ZIP › ExpertsModulexxx.pdf]

PLANT TRANSFORMATION - Advanced Mode

Forms

- Add species to parent table
- Media experts
- Methods experts
- Parents experts

Queries

- Append to lab book (agrobateria mediated transformation)
- Append to lab book (ballistic transformation)
- Append stock media
- Construct - plasmid approval
- "Karteikartenabfrage"

Tables

- Agrobacteria
- Construct
- Container
- Working groups

Back to overview
