# Supplementary material for: Documentation system for plant transformation service and research
Source: Plant Methods. 2010 Jan 27;6:4. doi: 10.1186/1746-4811-6-4 (PMC2835674; doi:10.1186/1746-4811-6-4)
Supplement: Additional file 2 — SupplementaryFigures. The file contains pdf-files with screenshots on various forms of MSTransformation2003 to enable readers without access to MS-Access to view the forms. The content of each screenshot is addressed in the manuscript. [file 1746-4811-6-4-S2.ZIP › Media_E_1.pdf]

Name MgSO4 1M H20_new **ID** 4

Code:

Old name:

Remark:

Copy medium

Solvent: H2Odd Sterilisation: non sterile Supplier: MPIMP

Storage condition: RT Storage site:

Composition

Stock	Amount	Unit	Show medium
MgSO4 x 7 H20	246,48	g/l	...

Show label Print label Show file card Print file card

Name Nt shoot induction Km50_new **ID** 5

Code:

Old name:

Remark:

Copy medium

Solvent: Sterilisation: Supplier: MPIMP

Storage condition: Storage site:

Composition

Show label | Print label | Show file card | Print file card

**Name**	Glucose 16 %_new	**ID**	7
Old name:		Code:	
Remark:	Autoclave 400 ml in 500 ml bottle		Copy medium

Solvent:	H2Odd	Sterilisation:	autoclave	Supplier:	MPIMP
Storage condition:	RT	Storage site:			

Composition

Show label | Print label | Show file card | Print file card

Name MS Select 8.0 Suc 2_new

ID 8

Code:

Old name: 2 MS 8.0_neu

Remark: pH 5,8

Copy medium

Solvent: H2Odd
Sterilisation: autoclave
Supplier: MPIMP

Storage condition: RT
Storage site:

Composition

Stock	Amount	Unit	Show medium
MS salts and vitamins	4,4	g/l	...
Select Agar	8	g/l	...
Sucrose	20	g/l	...

Show label | Print label | Show file card | Print file card

Name MS Select 6.8_new

ID 9

Code:

Old name:

Remark:

Copy medium

Solvent: H2Odd
Sterilisation: autoclave
Supplier: MPIMP

Storage condition: RT
Storage site:

Composition

Show label | Print label | Show file card | Print file card

Name Km 50 mg/ml H20_new

ID 11

Code:

Old name:

Remark:

Copy medium

Solvent: H2Odd

Sterilisation: sterile filtration

Supplier: MPIMP

Storage condition: -20°C

Storage site:

Composition

Show label | Print label | Show file card | Print file card

Name BAP 1 mg/ml DMSO_new **ID** 12

Code:

Old name:

Remark:

Copy medium

Solvent: DMSO Sterilisation: sterile filtration Supplier: MPIMP

Storage condition: -20°C Storage site:

Composition

Stock	Amount	Unit	Show medium
Benzylaminopurin	1	mg/ml	...

Show label | Print label | Show file card | Print file card

Name YEB liquid_new **ID** 15

Code:

Old name:

Remark:

Copy medium

Solvent: H2Odd Sterilisation: autoclave Supplier: MPIMP

Storage condition: RT Storage site:

Composition

Stock	Amount	Unit	Show medium
Beef extract (Difco)	1	g/l	...
Sucrose	5	g/l	...
Peptone (casein hydrolysat)	5	g/l	...
Yeast extract	1	g/l	...

Show label | Print label | Show file card | Print file card

Name YEB solid_new

ID 17

Old name:

Code:

Remark:

Copy medium

Solvent: H2Odd

Sterilisation: autoclave

Supplier: MPIMP

Storage condition: RT

Storage site:

Composition

Stock	Amount	Unit	Show medium
Beef extract (Difco)	5	g/l	...
Yeast extract	1	g/l	...
Peptone (casein hydrolysat)	5	g/l	...
Sucrose	5	g/l	...
Select Agar	15	g/l	...

Show label | Print label | Show file card | Print file card

Name YEB solid_new_new **ID** 18

Old name:

Code:

Remark:

Copy medium

Solvent: H2Odd Sterilisation: autoclave Supplier: MPIMP

Storage condition: RT Storage site:

Composition

Stock	Amount	Unit	Show medium
Beef extract (Difco)	5	g/l	...
Yeast extract	1	g/l	...
Peptone (casein hydrolysat)	5	g/l	...
Sucrose	5	g/l	...
Select Agar	15	g/l	...

Show label | Print label | Show file card | Print file card

Name YEB solid **ID** 400

Old name:

Code:

Remark:

Copy medium

Solvent: H2Odd Sterilisation: autoclave Supplier: MPIMP

Storage condition: RT Storage site:

Composition

Stock	Amount	Unit	Show medium
Beef extract (Difco)	5	g/l	...
Yeast extract	1	g/l	...
Peptone (casein hydrolysat)	5	g/l	...
Sucrose	5	g/l	...
Select Agar	15	g/l	...

Show label | Print label | Show file card | Print file card

Name YEB liquid

ID 401

Old name:

Code:

Remark:

Copy medium

Solvent: H2Odd

Sterilisation: autoclave

Supplier: MPIMP

Storage condition: RT

Storage site:

Composition

Stock	Amount	Unit	Show medium
Peptone (casein hydrolysat)	5	g/l	...
Yeast extract	1	g/l	...
Beef extract (Difco)	1	g/l	...
Sucrose	5	g/l	...

Show label | Print label | Show file card | Print file card

Name MS Select 8.0 Suc 2

ID 402

Code:

Old name: 2 MS 8.0

Remark: pH 5,8

Copy medium

Solvent: H2Odd

Sterilisation: autoclave

Supplier: MPIMP

Storage condition: RT

Storage site:

Composition

Stock	Amount	Unit	Show medium
MS salts and vitamins	4,4	g/l	...
Select Agar	8	g/l	...
Sucrose	20	g/l	...

Show label | Print label | Show file card | Print file card

Name MgSO4 10 mmol/H20

ID 403

Code:

Old name:

Remark:

Copy medium

Solvent: H2Odd

Sterilisation: autoclave

Supplier: MPIMP

Storage condition: 4°C

Storage site:

Composition

Stock	Amount	Unit	Show medium
MgSO4 1M H20	10	ml/l	...

Show label | Print label | Show file card | Print file card

Name Nt shoot induction Km50

ID 404

Old name:

Code:

Remark:

Copy medium

Solvent:

Sterilisation:

Supplier: MPIMP

Storage condition:

Storage site:

Composition

Stock	Amount	Unit	Show medium
MS Select 6.8	900	ml/l	...
Glucose 16 %	100	ml/l	...
BAP 1 mg/ml DMSO	1	ml/l	...
Km 50 mg/ml H20	1	ml/l	...
NAA 1 mg/ml EtOH	100	ml/l	...
Cla 125 mg/ml H20	2	ml/l	...

Show label | Print label | Show file card | Print file card

Name MS Select 8.0 Suc 2 Cla **ID** 405

Old name:

Code:

Remark:

Copy medium

Solvent: Sterilisation: Supplier: MPIMP

Storage condition: Storage site:

Composition

Stock	Amount	Unit	Show medium
MS Select 8.0 Suc 2	0		...
Cla 125 mg/ml H20	0		...

Show label | Print label | Show file card | Print file card

Name Select Agar **ID** 406

Old name:

Code:

Remark:

Copy medium

Solvent: solid Sterilisation: non sterile Supplier: MPIMP

Storage condition: RT Storage site:

Composition

Show label | Print label | Show file card | Print file card

Name Sucrose

ID 407

Code:

Old name:

Remark:

Copy medium

Solvent: solid

Sterilisation: non sterile

Supplier: MPIMP

Storage condition: RT

Storage site:

Composition

Show label | Print label | Show file card | Print file card

Name Peptone (casein hydrolysat) **ID** 408

Old name:

Code:

Remark:

Copy medium

Solvent: solid Sterilisation: non sterile Supplier: MPIMP

Storage condition: RT Storage site:

Composition

Show label | Print label | Show file card | Print file card

Name Beef extract (Difco) **ID** 409

Old name:

Code:

Remark:

Copy medium

Solvent: solid Sterilisation: non sterile Supplier: MPIMP

Storage condition: RT Storage site:

Composition

Show label | Print label | Show file card | Print file card

**Name**	Yeast extract	**ID**	410
Old name:		Code:	
Remark:			Copy medium

Solvent:	solid	Sterilisation:	non sterile	Supplier:	MPIMP
Storage condition:	RT	Storage site:			

Composition

Show label | Print label | Show file card | Print file card

Name Glucose 16 % **ID** 411

Code:

Old name:

Remark: Autoclave 400 ml in 500 ml bottle

Copy medium

Solvent: H_2Odd Sterilisation: autoclave Supplier: MPIMP

Storage condition: RT Storage site:

Composition

Stock	Amount	Unit	Show medium
Glucose	160	g/l	...

Show label | Print label | Show file card | Print file card

Name MS salts and vitamins **ID** 412

Code:

Old name:

Remark:

Copy medium

Solvent: solid Sterilisation: non sterile Supplier: MPIMP

Storage condition: RT Storage site:

Composition

Show label | Print label | Show file card | Print file card

**Name**	Glucose	**ID**	413
Old name:		Code:	
Remark:			Copy medium
Solvent:	solid	Sterilisation:	non sterile
Supplier:	MPIMP		
Storage condition:	RT	Storage site:	

Composition

Show label | Print label | Show file card | Print file card

Name MS Select 6.8

ID 414

Old name:

Code:

Remark:

Copy medium

Solvent: H2Odd

Sterilisation: autoclave

Supplier: MPIMP

Storage condition: RT

Storage site:

Composition

Stock	Amount	Unit	Show medium
Select Agar	6,8	g/900 ml	...
MS salts and vitamins	4,4	g/900 ml	...

Show label | Print label | Show file card | Print file card

Name NAA 1 mg/ml EtOH

ID 415

Old name:

Code:

Remark:

Copy medium

Solvent: Ethanol

Sterilisation: sterile filtration

Supplier: MPIMP

Storage condition: -20°C

Storage site:

Composition

Stock	Amount	Unit	Show medium
Nicotinamide	1	mg/ml	...

Show label | Print label | Show file card | Print file card

Name Cla 125 mg/ml H20

ID 416

Code:

Old name:

Remark:

Copy medium

Solvent: H2Odd

Sterilisation: sterile filtration

Supplier: MPIMP

Storage condition: -20°C

Storage site:

Composition

Stock	Amount	Unit	Show medium
Claforan	125	mg/ml	...

Show label | Print label | Show file card | Print file card

Name Km 50 mg/ml H20

ID 417

Code:

Old name:

Remark:

Copy medium

Solvent: H2Odd

Sterilisation: sterile filtration

Supplier: MPIMP

Storage condition: -20°C

Storage site:

Composition

Stock	Amount	Unit	Show medium
Kanamycin	50	mg/ml	...

Show label | Print label | Show file card | Print file card

Name BAP 1 mg/ml DMSO

ID 418

Code:

Old name:

Remark:

Copy medium

Solvent: DMSO

Sterilisation: sterile filtration

Supplier: MPIMP

Storage condition: -20°C

Storage site:

Composition

Stock	Amount	Unit	Show medium
Benzylaminopurin	1	mg/ml	...

Show label | Print label | Show file card | Print file card

Name Sucrose 20 %

ID 419

Code:

Old name:

Remark:

Copy medium

Solvent: H2Odd

Sterilisation: autoclave

Supplier: MPIMP

Storage condition: RT

Storage site:

Composition

Stock	Amount	Unit	Show medium
Sucrose	200	g/l	...

Show label | Print label | Show file card | Print file card

Name Kanamycin **ID** 420

Code:

Old name:

Remark:

Copy medium

Solvent: solid Sterilisation: non sterile Supplier: MPIMP

Storage condition: -20°C Storage site:

Composition

Show label | Print label | Show file card | Print file card

Name MgSO4 x 7 H20 **ID** 421

Code:

Old name:

Remark:

Copy medium

Solvent: solid Sterilisation: non sterile Supplier: MPIMP

Storage condition: RT Storage site:

Composition

Show label | Print label

Show file card | Print file card

Name Benzylaminopurin

ID 422

Old name:

Code:

Remark:

Copy medium

Solvent: solid

Sterilisation: non sterile

Supplier: MPIMP

Storage condition: -20°C

Storage site:

Composition

Show label | Print label | Show file card | Print file card

Name Claforan **ID** 423

Code:

Old name:

Remark: Cefotaxime Sodium

Copy medium

Solvent: solid Sterilisation: non sterile Supplier: MPIMP

Storage condition: -20°C Storage site:

Composition

Show label | Print label | Show file card | Print file card

Name MgSO4 1M H20 **ID** 424

Code:

Old name:

Remark:

Copy medium

Solvent: H2Odd Sterilisation: non sterile Supplier: MPIMP

Storage condition: RT Storage site:

Composition

Stock	Amount	Unit	Show medium
MgSO4 x 7 H20	246,48	g/l	...

Show label | Print label | Show file card | Print file card

Name Nicotinamide

ID 425

Old name:

Code:

Remark:

Copy medium

Solvent: solid

Sterilisation: non sterile

Supplier: MPIMP

Storage condition: RT

Storage site:

Composition

Show label Print label

Show file card Print file card
