# Supplementary material for: Documentation system for plant transformation service and research
Source: Plant Methods. 2010 Jan 27;6:4. doi: 10.1186/1746-4811-6-4 (PMC2835674; doi:10.1186/1746-4811-6-4)
Supplement: Additional file 2 — SupplementaryFigures. The file contains pdf-files with screenshots on various forms of MSTransformation2003 to enable readers without access to MS-Access to view the forms. The content of each screenshot is addressed in the manuscript. [file 1746-4811-6-4-S2.ZIP › Media_E_2.pdf]

Method **Test Protokoll_new** Method ID 52

Species Nicotiana tabaccum

Selection Km

Copy method

Genome Nucleus

Concentration 0 µg/ml

Details and notes

Method steps

Process	Days from start	Remark	Method step ID	L
	-2	first step	378	
	-1	second step	379	
Transformation	0	Centrifuge Agrobacteria 15 min at 4000 r	380	low lig

Copy step from other method into current method

Copy method step
