# Supplementary material for: Documentation system for plant transformation service and research
Source: Plant Methods. 2010 Jan 27;6:4. doi: 10.1186/1746-4811-6-4 (PMC2835674; doi:10.1186/1746-4811-6-4)
Supplement: Additional file 2 — SupplementaryFigures. The file contains pdf-files with screenshots on various forms of MSTransformation2003 to enable readers without access to MS-Access to view the forms. The content of each screenshot is addressed in the manuscript. [file 1746-4811-6-4-S2.ZIP › MethodsExperts.pdf]

Method ID	34
Method Name	Tobacco transformation
Selection	Km
Selection concentration	50
Species	Nicotiana tabaccum
Details	
Genom	Nucleus
Obsolet	
Approved	02.07.2009

Method ID	37
Method Name	Test Protokoll
Selection	Km
Selection concentration	0
Species	Nicotiana tabaccum
Details	
Genom	Nucleus
Obsolet	12.10.2009
Approved	01.10.2009

Method ID	52
Method Name	Test Protokoll_new
Selection	Km
Selection concentration	0
Species	Nicotiana tabaccum
Details	
Genom	Nucleus
Obsolet	
Approved	
