# Supplementary material for: Documentation system for plant transformation service and research
Source: Plant Methods. 2010 Jan 27;6:4. doi: 10.1186/1746-4811-6-4 (PMC2835674; doi:10.1186/1746-4811-6-4)
Supplement: Additional file 2 — SupplementaryFigures. The file contains pdf-files with screenshots on various forms of MSTransformation2003 to enable readers without access to MS-Access to view the forms. The content of each screenshot is addressed in the manuscript. [file 1746-4811-6-4-S2.ZIP › Method_E_1.pdf]

Method **Test Protokoll_new** Method ID 52

Species Nicotiana tabaccum

Genome Nucleus

Selection Km

Concentration 0 µg/ml

Copy method

Details and notes

Method steps

Days from start -2

Method step 378

Process

Temperature: 0

Remark first step

Light

Media

Media Benzylaminopurin Show

Container

Remark

Code

Days from start -1

Method step 379

Process

Temperature: 0

Remark second step

Light

Media

Media Glucose Show

Container

Remark

Code

Days from start: 0

Method step: 380

Process: Transformation

Temperature: 22

Remark: Centrifuge Agrobacteria 15 min at 4000 rpm. Resuspend in 10 mM MgSO4 and fill into small petri dish. Cut leaves in MgSO4, discard mid rip. Bath cuttings in Agrobacteria suspension and place them close to each other on MS Select 8.0 Suc2

Light: low light

Media

Media	MgSO4 1M H20	Show	Container	Petridish, very small
Remark			Code	
Media	MS Select 8.0 Suc 2	Show	Container	Petridish, small
Remark			Code	

Copy step from other method into current method

Copy method step

Method: **Test Protokoll**

Method ID: 37

Species: Nicotiana tabaccum

Selection: Km

Genome: Nucleus

Concentration: 0 µg/ml

Copy method

Details and notes

Method steps

Days from start	-2	Method step	353
Process		Temperature:	0
Remark	first step	Light	

Media

Media	Benzylaminopurin	Show	Container	
Remark			Code	

Days from start	-1	Method step	354
Process		Temperature:	0
Remark	second step	Light	

Media

Media	Glucose	Show	Container	
Remark			Code	

Copy step from other method into current method

Copy method step

Method	**Tobacco transformation**		Method ID	34
Species	Nicotiana tabaccum	Selection	Km	Copy method
Genome	Nucleus	Concentration	50 µg/ml	

Details and notes

Method steps

Days from start	-3	Method step	338
Process	Spread Agrobacteria on plate	Temperature:	28
Remark	Strain GV2260	Light	dark

Media

Media	YEB solid	Show	Container	Petridish, small
Remark	Adjust antibotics concentration to bacterial marker on plasmid		Code	

Days from start	-1	Method step	339
Process	Start overnight culture	Temperature:	28
Remark	Inoculate 10 ml YEB liquid in 50 ml Erlenmeyerflask with one colony and incubate over night at 28 °C on a rotary shaker at 180 rpm	Light	dark

Media

Media	YEB liquid	Show	Container	Erlenmeyerflask, 50 ml
Remark	Adjust antibotics concentration to bacterial marker on plasmid		Code	

Days from start	0	Method step	340
Process	Transformation	Temperature:	22

Remark	Centrifuge Agrobacteria 15 min at 4000 rpm. Resuspend in 10 mM MgSO4 and fill into small petri dish. Cut leaves in MgSO4, discard mid rip. Bath cuttings in Agrobacteria suspension and place them close to each other on MS Select 8.0 Suc2	Light	low light

Media

Media	MgSO4 1M H20	Show	Container	Petridish, very small
Remark			Code	
Media	MS Select 8.0 Suc 2	Show	Container	Petridish, small
Remark			Code	

Days from start	2	Method step	341
Process	Transfer to shoot induction	Temperature:	22
Remark	Transfer to new plates with	Light	low light

Media

Media	Nt shoot induction Km50	Show	Container	Petridish, big
Remark			Code	

Copy step from other method into current method

Copy method step
