# Supplementary material for: Documentation system for plant transformation service and research
Source: Plant Methods. 2010 Jan 27;6:4. doi: 10.1186/1746-4811-6-4 (PMC2835674; doi:10.1186/1746-4811-6-4)
Supplement: Additional file 2 — SupplementaryFigures. The file contains pdf-files with screenshots on various forms of MSTransformation2003 to enable readers without access to MS-Access to view the forms. The content of each screenshot is addressed in the manuscript. [file 1746-4811-6-4-S2.ZIP › Next31days.pdf]

ID	Date7	Variety	Step	Construct	Protocol	Resist	Transformation	Operator
659	30.01.2010	SNN	Spread Agrobacteria on plate	315995	Tobacco transformation	Km	02.02.2010	Gremmels
659	01.02.2010	SNN	Start overnight culture	315995	Tobacco transformation	Km	02.02.2010	Gremmels
659	02.02.2010	SNN	Transformation	315995	Tobacco transformation	Km	02.02.2010	Gremmels
659	04.02.2010	SNN	Transfer to shoot induction	315995	Tobacco transformation	Km	02.02.2010	Gremmels

Number of Lines
0
0
0
0
