# Supplementary material for: Documentation system for plant transformation service and research
Source: Plant Methods. 2010 Jan 27;6:4. doi: 10.1186/1746-4811-6-4 (PMC2835674; doi:10.1186/1746-4811-6-4)
Supplement: Additional file 2 — SupplementaryFigures. The file contains pdf-files with screenshots on various forms of MSTransformation2003 to enable readers without access to MS-Access to view the forms. The content of each screenshot is addressed in the manuscript. [file 1746-4811-6-4-S2.ZIP › Startpage.pdf]

PLANT TRANSFORMATION

Experiments module

Enter ballistic transformation | Open lab book

Enter agrobacteria mediated transformation | Open lab book

Work overview

Today | Next 7 days | Next 9 days

Methods module

Method form | ☐ with datasheet view

Media module

Media form | ☐ with datasheet view

Where are the media?

Go to experts module

Exit Database
