# Supplementary material for: Documentation system for plant transformation service and research
Source: Plant Methods. 2010 Jan 27;6:4. doi: 10.1186/1746-4811-6-4 (PMC2835674; doi:10.1186/1746-4811-6-4)
Supplement: Additional file 3 — Readme. The file contains a short introduction to the system. It is assumed that the user is generally familiar with the software MS-Access. Thus, the introduction repeats only the most important features in handling the software and otherwise restricts itself to the features specific to our database. [file 1746-4811-6-4-S3.PDF]

Readme for Transformation2003.mdb

Requirements

The database is in Access2003 format and can be opened with MSAccess 2003 or MSAccess 2007. It can be upgraded to MSAccess2007 format. However, the early versions of MSAccess2007 messed up the 'custom groups' links during the upgrade. The following introduction is written for MSAccess2007 users that use the database in the Access2003 format. It is assumed that readers have worked with MS Access 2003 or 2007 before.

Start

1. Download the additional file 'MSTransformation2003.zip' from the webpage, extract the file 'transformation2003.mdb' from it and double-click the file to open the database.
2. Enable Macros for the database. Choose the Display format 'Tabbed documents' in 'Access Options – Current Database, Application Options'.
3. The database opens with the 'Plant Transformation Welcome screen' that gives access to the most important data entry forms and reports for the user. On clicking a function, an additional window opens and displays the form. The form can be closed again by clicking the 'x' in the upper right corner of a form.
4. The' Navigation Pane' of the left side of the database window is collapsed. It opens when the 'Welcome Screen' is closed. The 'Navigation Pane' will be in the state it was left when the last user closed the database. If 'Custom groups' are not displayed, click on the ∇ Symbol on the upper right corner of the 'Navigation Pane' and choose 'Custom groups' from the menu. You can open the 'WelcomeScreen' again by double-clicking 'WelcomeScreen' in the custom group 'Favorites'.
5. Expert forms (like adding new species, parents, users and workgroups) can be accessed from the 'ExpertsModuleScreen'. This screen is accessible from the 'WelcomeScreen' and from the custom group 'Experts module administration'.

Data entry

- To see the data, open the appropriate form ('Media form', 'Method form' or 'Enter (..) transformation'). The system generates a view on the data at the time of opening the form. To enter new data, generate a new entry sheet of the form by clicking the 'new record' button at the bottom of the form (>*). Data are saved immediately after each entry. In the same way, Access overwrites existing entries without warning!
- Data need to be entered in a certain order. Media and container data need to be entered first before these data can be used in the method fields. Likewise, method data need to be entered before the method can be used in the experiment module. However, you can start the process by just entering the name of the method or a medium and put 'flesh on the bone' later on. This way, you can start top-down (as we did) instead of bottom-up (as the system is meant to be).
- You can have several forms open at the same time. Beware, that if a form A shows data entered on another form B. Data that have been newly entered into form B after opening form A, will NOT directly show on form A. Refresh the view by pressing 'F5-key' or clicking the 'Refresh All' button.

Reports

- Work overview reports that are accessible from the ‘WelcomeScreen’ are automatically filtering the work based on the current date. Thus, these reports may by completely empty if no transformations have been entered in the time frame when you open the report.
- The’ file card’ function on the Media form shows the content of the ‘safety copy’ of the media table. This safety copy is generated by clicking the ‘Update filecards’ function on the ’ExpertModuleScreen’.

Recommendation

Avoid changing variable names, rather change the label in a form or in a report. NEVER change the name of a variable that is a primary key (key symbol in the table design view).
