# Supplementary figures and images for: Documentation system for plant transformation service and research
Source: Plant Methods. 2010 Jan 27;6:4. doi: 10.1186/1746-4811-6-4 (PMC2835674; doi:10.1186/1746-4811-6-4)

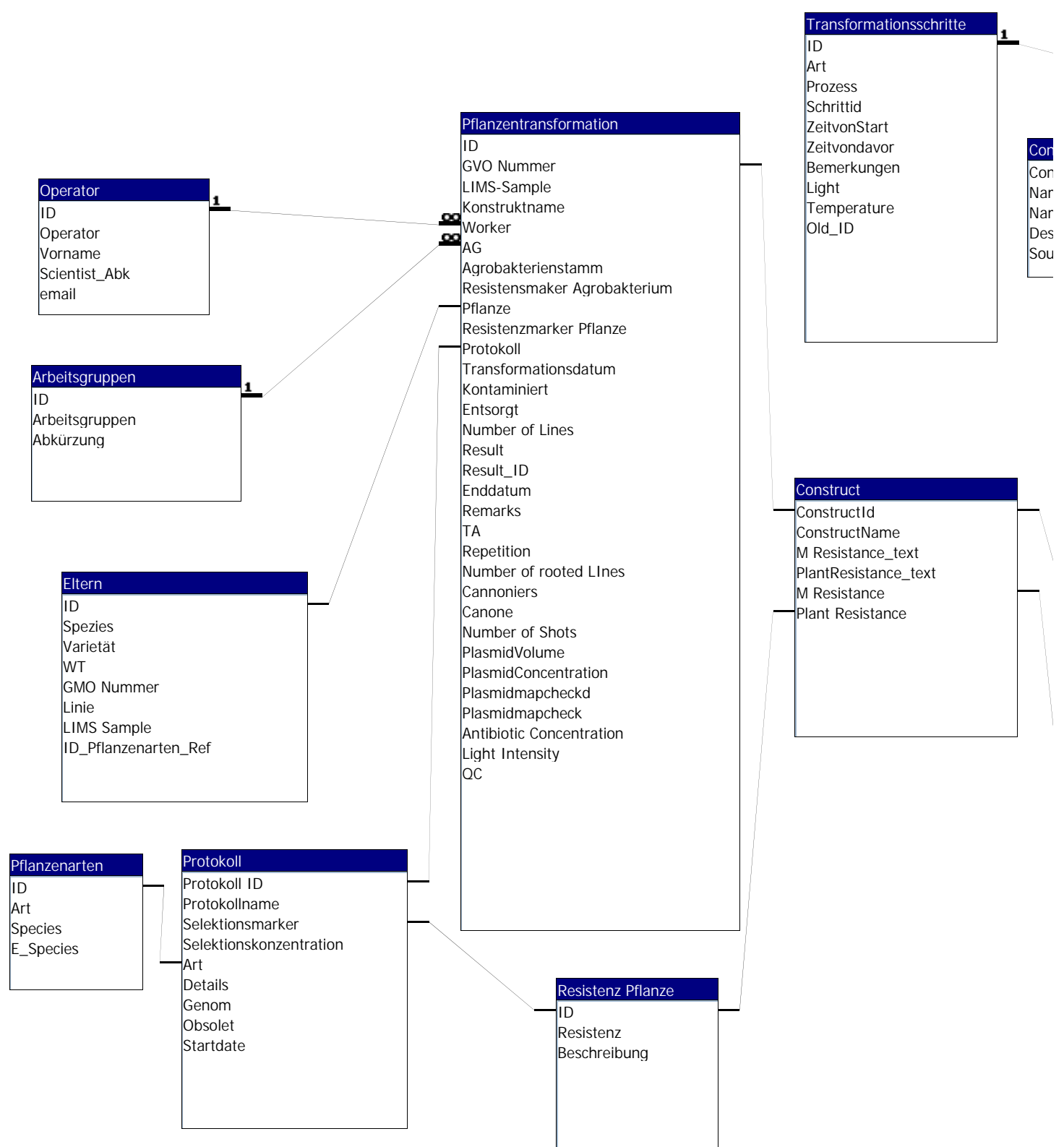

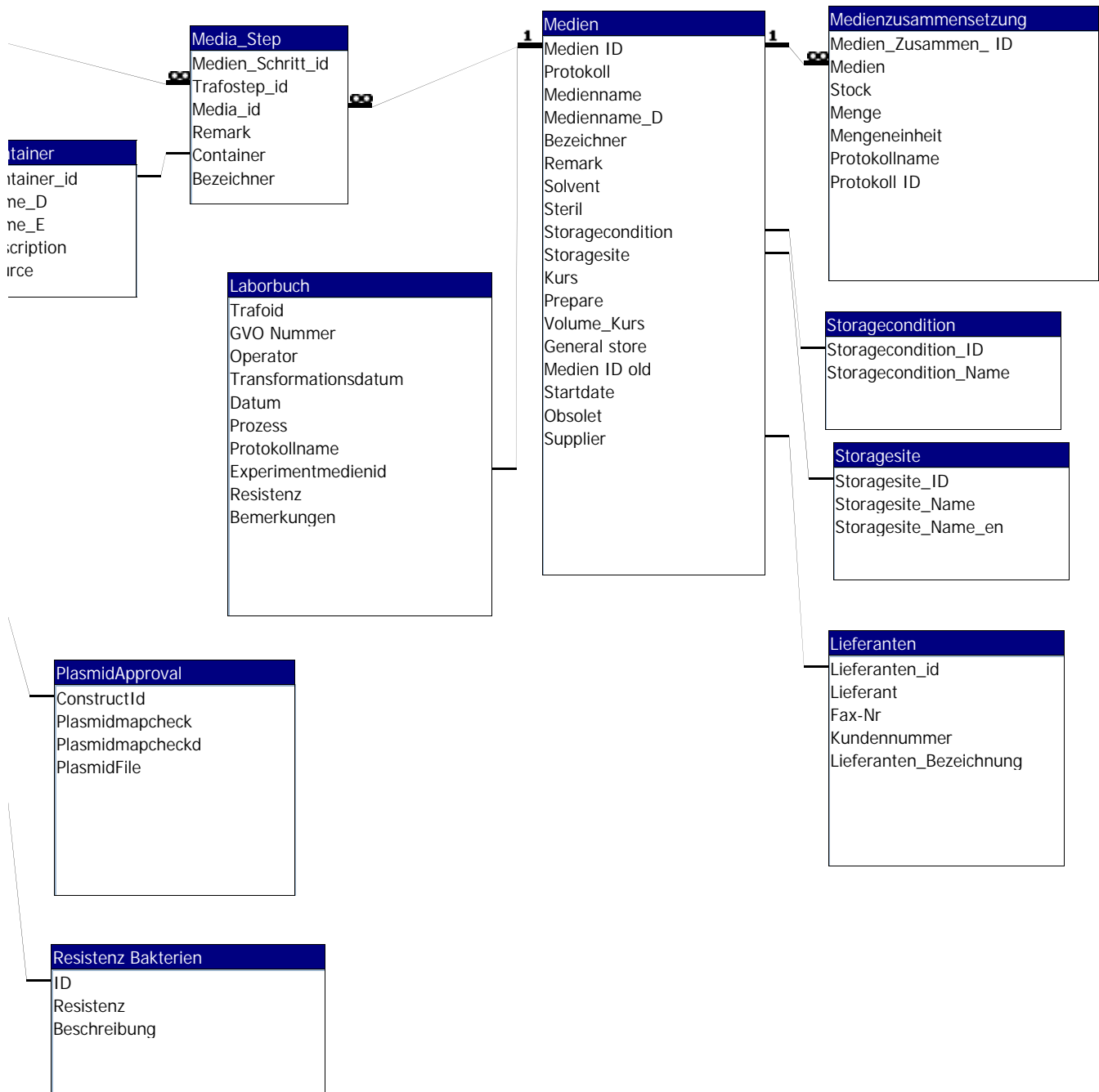

Supplement: Additional file 4 — ERdiagramTransformation2003. The file contains the entity relationship diagram for the database MSTransformation2003.mdb. [file 1746-4811-6-4-S4.PDF]
