# Supplementary material for: Documentation system for plant transformation service and research
Source: Plant Methods. 2010 Jan 27;6:4. doi: 10.1186/1746-4811-6-4 (PMC2835674; doi:10.1186/1746-4811-6-4)
Supplement: Additional file 5 — Object Definition for Transformation2003.pdf. The file contains the object definition for the database MSTransformation2003.mdb [file 1746-4811-6-4-S5.PDF]

Properties

DateCreated:	30.06.2009 13:54:34	DefaultView:	2
DOL:	Long binary data	FilterOnLoad:	False
GUID:	{guid {BF1DFA00-E28A-4A94-A8C5-2AAE518B14C9}}	LastUpdated:	06.10.2009 11:31:08
MaxRecords:	0	ODBCTimeout:	60
OrderByOn:	False	OrderByOnLoad:	True
Orientation:	Left-to-Right	RecordLocks:	No Locks
RecordsAffected:	0	RecordsetType:	Dynaset
ReturnsRecords:	True	TotalsRow:	False
Type:	0	Updatable:	True

SQL

```
SELECT Pflanzentransformation.ID, Pflanzentransformation.[GVO Nummer], Construct.ConstructName,
Pflanzentransformation.Worker, Pflanzentransformation.AG, Pflanzentransformation.Agrobakterienstamm,
Pflanzentransformation.Pflanze, [Resistenz Pflanze].Resistenz, Pflanzentransformation.Protokoll,
Pflanzentransformation.Transformationsdatum, Pflanzentransformation.Kontaminiert,
Pflanzentransformation.Entsorgt, Pflanzentransformation.[Number of Lines], Pflanzentransformation.Result,
Pflanzentransformation.Enddatum, Pflanzentransformation.Remarks, Eltern.Spezies, Eltern.Varietät, Eltern.WT,
Eltern.[GMO Nummer], Eltern.Linie, [Resistenz Bakterien].Resistenz, Pflanzentransformation.[LIMS-Sample],
Protokoll.Protokollname, Pflanzentransformation.TA, Pflanzentransformation.Repetition,
Pflanzentransformation.[Number of rooted LInes], Pflanzentransformation.Cannoniers,
Pflanzentransformation.Canone, Pflanzentransformation.[Number of Shots],
Pflanzentransformation.PlasmidVolume, Pflanzentransformation.PlasmidConcentration,
Pflanzentransformation.Plasmidmapcheckd, Pflanzentransformation.Plasmidmapcheck,
Pflanzentransformation.Result_ID
FROM [Resistenz Bakterien] RIGHT JOIN ([Resistenz Pflanze] RIGHT JOIN (Construct INNER JOIN (Protokoll
INNER JOIN (Eltern INNER JOIN Pflanzentransformation ON Eltern.ID = Pflanzentransformation.Pflanze) ON
Protokoll.[Protokoll ID] = Pflanzentransformation.Protokoll) ON Construct.ConstructId =
Pflanzentransformation.[GVO Nummer]) ON [Resistenz Pflanze].ID = Construct.[Plant Resistance]) ON
[Resistenz Bakterien].ID = Construct.[M Resistance]
WHERE (((Pflanzentransformation.Enddatum) Is Null Or (Pflanzentransformation.Enddatum)>Date()-28) AND
((Pflanzentransformation.TA) Like "* Buch*"))
ORDER BY Pflanzentransformation.ID DESC;
```

Columns

Name	Type	Size
ID	Long Integer	4

AggregateType:	-1
AllowZeroLength:	False
AppendOnly:	False
Attributes:	Fixed Size; Auto-Increment; Updatable
CollatingOrder:	General
ColumnHidden:	False
ColumnOrder:	Default
ColumnWidth:	Default
DataUpdatable:	True
GUID:	{guid {491011A1-F233-4D8B-AF6D-E85AC96DC00A}}
OrdinalPosition:	0
Required:	False
SourceField:	ID
SourceTable:	Pflanzentransformation
TextAlign:	General

GVO Nummer	Long Integer	4
AggregateType:	-1	
AllowZeroLength:	False	
AppendOnly:	False	
Attributes:	Fixed Size; Updatable	
CollatingOrder:	General	
ColumnHidden:	False	
ColumnOrder:	Default	
ColumnWidth:	1650	
DataUpdatable:	True	
DecimalPlaces:	Auto	
DefaultValue:	0	
DisplayControl:	Text Box	
GUID:	{guid {78A41B5D-B257-469B-8FCD-4CB99C084269}}	
OrdinalPosition:	1	
Required:	True	
SourceField:	GVO Nummer	
SourceTable:	Pflanzentransformation	
TextAlign:	General	
ConstructName	Text	255
AggregateType:	-1	
AllowZeroLength:	True	
AppendOnly:	False	
Attributes:	Variable Length; Updatable	
CollatingOrder:	General	
ColumnHidden:	False	
ColumnOrder:	Default	
ColumnWidth:	2700	
DataUpdatable:	True	
DisplayControl:	Text Box	
IMEMode:	0	
IMESentenceMode:	3	
OrdinalPosition:	2	
Required:	False	
SourceField:	ConstructName	
SourceTable:	Construct	
TextAlign:	General	
UnicodeCompression:	False	
Worker	Long Integer	4
AggregateType:	-1	
AllowZeroLength:	False	
AppendOnly:	False	
Attributes:	Fixed Size; Updatable	
CollatingOrder:	General	
ColumnHidden:	False	
ColumnOrder:	Default	
ColumnWidth:	Default	
DataUpdatable:	True	
DecimalPlaces:	Auto	
DefaultValue:	0	
DisplayControl:	Text Box	
GUID:	{guid {2352BCDA-60BE-487E-B967-3F210FA0BEE0}}	
OrdinalPosition:	3	
Required:	False	

SourceField:	Worker	
SourceTable:	Pflanzentransformation	
TextAlign:	General	

AG	Long Integer	4
AggregateType:	-1	
AllowZeroLength:	False	
AppendOnly:	False	
Attributes:	Fixed Size; Updatable	
CollatingOrder:	General	
ColumnHidden:	False	
ColumnOrder:	Default	
ColumnWidth:	Default	
DataUpdatable:	True	
DecimalPlaces:	Auto	
DefaultValue:	0	
DisplayControl:	Text Box	
GUID:	{guid {856AEBC1-28B9-4ECD-B43A-F74E08572BEC}}	
OrdinalPosition:	4	
Required:	False	
SourceField:	AG	
SourceTable:	Pflanzentransformation	
TextAlign:	General	

Agrobakterienstamm	Long Integer	4
AggregateType:	-1	
AllowZeroLength:	False	
AppendOnly:	False	
Attributes:	Fixed Size; Updatable	
CollatingOrder:	General	
ColumnHidden:	False	
ColumnOrder:	Default	
ColumnWidth:	2010	
DataUpdatable:	True	
DecimalPlaces:	Auto	
DefaultValue:	0	
DisplayControl:	Text Box	
GUID:	{guid {F51D32DD-0FB9-410A-9B6B-D205562601B3}}	
OrdinalPosition:	5	
Required:	False	
SourceField:	Agrobakterienstamm	
SourceTable:	Pflanzentransformation	
TextAlign:	General	

Pflanze	Long Integer	4
AggregateType:	-1	
AllowMultipleValues:	False	
AllowValueListEdits:	False	
AllowZeroLength:	False	
AppendOnly:	False	
Attributes:	Fixed Size; Updatable	
BoundColumn:	1	
CollatingOrder:	General	
ColumnCount:	6	
ColumnHeads:	False	
ColumnHidden:	False	

ColumnOrder:	Default
ColumnWidth:	2520
ColumnWidths:	0;1830;1650;630;1260;705
DataUpdatable:	True
DecimalPlaces:	Auto
DefaultValue:	0
DisplayControl:	Combo Box
GUID:	{guid {DEC170A3-C295-476F-A829-A424275F639C}}
LimitToList:	True
ListRows:	8
ListWidth:	6075twip
OrdinalPosition:	6
Required:	False
RowSource:	SELECT Eltern.ID, Eltern.Spezies, Eltern.Varietät, Eltern.WT, Eltern.[GMO Nummer], Eltern.Linie FROM Eltern;
RowSourceType:	Table/Query
ShowOnlyRowSourceValues:	False
SourceField:	Pflanze
SourceTable:	Pflanzentransformation
TextAlign:	General

Resistenz Pflanze.Resistenz Text 50

AggregateType:	-1
AllowZeroLength:	True
AppendOnly:	False
Attributes:	Variable Length; Updatable
CollatingOrder:	General
ColumnHidden:	False
ColumnOrder:	Default
ColumnWidth:	Default
DataUpdatable:	True
DisplayControl:	Text Box
GUID:	{guid {8D71BD08-D542-4B6A-8796-02E82F37D7E2}}
IMEMode:	0
IMESentenceMode:	3
OrdinalPosition:	7
Required:	False
SourceField:	Resistenz
SourceTable:	Resistenz Pflanze
TextAlign:	General
UnicodeCompression:	True

Protokoll Long Integer 4

AggregateType:	-1
AllowZeroLength:	False
AppendOnly:	False
Attributes:	Fixed Size; Updatable
CollatingOrder:	General
ColumnHidden:	False
ColumnOrder:	Default
ColumnWidth:	Default
DataUpdatable:	True
DecimalPlaces:	Auto
DefaultValue:	0
DisplayControl:	Text Box

GUID:	{guid {66638D24-E387-4387-948D-82A060A41F76}}	
OrdinalPosition:	8	
Required:	False	
SourceField:	Protokoll	
SourceTable:	Pflanzentransformation	
TextAlign:	General	
**Transformationsdatum**	**Date/Time**	**8**
AggregateType:	-1	
AllowZeroLength:	False	
AppendOnly:	False	
Attributes:	Fixed Size; Updatable	
CollatingOrder:	General	
ColumnHidden:	False	
ColumnOrder:	Default	
ColumnWidth:	2850	
DataUpdatable:	True	
GUID:	{guid {001E5420-4F1C-4D41-9502-4F9521AA6AF1}}	
IMEMode:	0	
IMESentenceMode:	3	
OrdinalPosition:	9	
Required:	False	
ShowDatePicker:	For dates	
SourceField:	Transformationsdatum	
SourceTable:	Pflanzentransformation	
TextAlign:	General	
**Kontaminiert**	**Yes/No**	**1**
AggregateType:	-1	
AllowZeroLength:	False	
AppendOnly:	False	
Attributes:	Fixed Size; Updatable	
CollatingOrder:	General	
ColumnHidden:	False	
ColumnOrder:	Default	
ColumnWidth:	Default	
DataUpdatable:	True	
DefaultValue:	False	
DisplayControl:	106	
Format:	Yes/No	
GUID:	{guid {EB0D9BA0-D280-440E-A86B-C50C95043FD0}}	
OrdinalPosition:	10	
Required:	False	
SourceField:	Kontaminiert	
SourceTable:	Pflanzentransformation	
TextAlign:	General	
**Entsorgt**	**Yes/No**	**1**
AggregateType:	-1	
AllowZeroLength:	False	
AppendOnly:	False	
Attributes:	Fixed Size; Updatable	
CollatingOrder:	General	
ColumnHidden:	False	
ColumnOrder:	Default	
ColumnWidth:	Default	

DataUpdatable:	True	
DefaultValue:	False	
DisplayControl:	106	
Format:	Yes/No	
GUID:	{guid {725C28E0-8B07-4DB8-84A2-2B1F3C9F0516}}	
OrdinalPosition:	11	
Required:	False	
SourceField:	Entsorgt	
SourceTable:	Pflanzentransformation	
TextAlign:	General	

Number of Lines	Long Integer	4
AggregateType:	-1	
AllowZeroLength:	False	
AppendOnly:	False	
Attributes:	Fixed Size; Updatable	
CollatingOrder:	General	
ColumnHidden:	False	
ColumnOrder:	Default	
ColumnWidth:	2130	
DataUpdatable:	True	
DecimalPlaces:	Auto	
DefaultValue:	0	
Description:	Number of Lines that have been cut (festgelegt 06.05.09)	
DisplayControl:	Text Box	
GUID:	{guid {85595EC7-656A-4467-A6CE-21630FBEB0B4}}	
OrdinalPosition:	12	
Required:	False	
SourceField:	Number of Lines	
SourceTable:	Pflanzentransformation	
TextAlign:	General	

Result	Text	50
AggregateType:	-1	
AllowMultipleValues:	False	
AllowValueListEdits:	False	
AllowZeroLength:	True	
AppendOnly:	False	
Attributes:	Variable Length; Updatable	
BoundColumn:	1	
CollatingOrder:	General	
ColumnCount:	1	
ColumnHeads:	False	
ColumnHidden:	False	
ColumnOrder:	Default	
ColumnWidth:	2280	
ColumnWidths:	2385	
DataUpdatable:	True	
DisplayControl:	Combo Box	
GUID:	{guid {678F1CC4-5A06-4A1D-A7D4-BBBE683B3FA1}}	
IMEMode:	0	
IMESentenceMode:	3	
LimitToList:	False	
ListRows:	8	
ListWidth:	2385twip	
OrdinalPosition:	13	

	Required:	False
	RowSource:	"übergeben";"kontaminiert";"zurückgezogen";"entsorgt: kein Kallus";"entsorgt: keine Regeneration";"andere"
	RowSourceType:	Value List
	ShowOnlyRowSourceValues:	False
	SourceField:	Result
	SourceTable:	Pflanzentransformation
	TextAlign:	General
	UnicodeCompression:	True

Enddatum		Date/Time	8
	AggregateType:	-1	
	AllowZeroLength:	False	
	AppendOnly:	False	
	Attributes:	Fixed Size; Updatable	
	CollatingOrder:	General	
	ColumnHidden:	False	
	ColumnOrder:	Default	
	ColumnWidth:	Default	
	DataUpdatable:	True	
	GUID:	{guid {9256514F-89E7-4417-895A-7E12A2ABA75E}}	
	IMEMode:	0	
	IMESentenceMode:	3	
	OrdinalPosition:	14	
	Required:	False	
	ShowDatePicker:	For dates	
	SourceField:	Enddatum	
	SourceTable:	Pflanzentransformation	
	TextAlign:	General	
Remarks		Memo	N/A
	AggregateType:	-1	
	AllowZeroLength:	True	
	AppendOnly:	False	
	Attributes:	Variable Length; Updatable	
	CollatingOrder:	General	
	ColumnHidden:	False	
	ColumnOrder:	Default	
	ColumnWidth:	Default	
	DataUpdatable:	True	
	GUID:	{guid {9E3C6856-13CF-4E9C-8E22-8E72D8B91407}}	
	IMEMode:	0	
	IMESentenceMode:	3	
	OrdinalPosition:	15	
	Required:	False	
	SourceField:	Remarks	
	SourceTable:	Pflanzentransformation	
	TextAlign:	General	
	TextFormat:	Plain Text	
	UnicodeCompression:	True	
Spezies		Text	50
	AggregateType:	-1	
	AllowZeroLength:	True	
	AppendOnly:	False	

Attributes:	Variable Length; Updatable	
CollatingOrder:	General	
ColumnHidden:	False	
ColumnOrder:	Default	
ColumnWidth:	3855	
DataUpdatable:	True	
DisplayControl:	Text Box	
GUID:	{guid {FA3945B0-6E6C-423D-8BEE-0935B5FD7526}}	
IMEMode:	0	
IMESentenceMode:	3	
OrdinalPosition:	16	
Required:	False	
SourceField:	Spezies	
SourceTable:	Eltern	
TextAlign:	General	
UnicodeCompression:	True	

Varietät	Text	50
AggregateType:	-1	
AllowZeroLength:	True	
AppendOnly:	False	
Attributes:	Variable Length; Updatable	
CollatingOrder:	General	
ColumnHidden:	False	
ColumnOrder:	Default	
ColumnWidth:	1695	
DataUpdatable:	True	
DisplayControl:	Text Box	
GUID:	{guid {1AF19923-4CB9-4A37-BDF6-9A285C5D1D00}}	
IMEMode:	0	
IMESentenceMode:	3	
OrdinalPosition:	17	
Required:	False	
SourceField:	Varietät	
SourceTable:	Eltern	
TextAlign:	General	
UnicodeCompression:	True	

WT	Yes/No	1
AggregateType:	-1	
AllowZeroLength:	False	
AppendOnly:	False	
Attributes:	Fixed Size; Updatable	
CollatingOrder:	General	
ColumnHidden:	False	
ColumnOrder:	Default	
ColumnWidth:	Default	
DataUpdatable:	True	
DisplayControl:	106	
Format:	Yes/No	
GUID:	{guid {C2BE4715-B4A6-4077-A3F6-D1026FADBAC3}}	
OrdinalPosition:	18	
Required:	False	
SourceField:	WT	
SourceTable:	Eltern	
TextAlign:	General	

GMO Nummer	Long Integer	4
AggregateType:	-1	
AllowZeroLength:	False	
AppendOnly:	False	
Attributes:	Fixed Size; Updatable	
CollatingOrder:	General	
ColumnHidden:	False	
ColumnOrder:	Default	
ColumnWidth:	Default	
DataUpdatable:	True	
DecimalPlaces:	Auto	
DefaultValue:	0	
DisplayControl:	Text Box	
GUID:	{guid {0374CE7E-E119-4264-8426-B9502000A391}}	
OrdinalPosition:	19	
Required:	False	
SourceField:	GMO Nummer	
SourceTable:	Eltern	
TextAlign:	General	
Linie	Long Integer	4
AggregateType:	-1	
AllowZeroLength:	False	
AppendOnly:	False	
Attributes:	Fixed Size; Updatable	
CollatingOrder:	General	
ColumnHidden:	False	
ColumnOrder:	Default	
ColumnWidth:	Default	
DataUpdatable:	True	
DecimalPlaces:	Auto	
DefaultValue:	0	
DisplayControl:	Text Box	
GUID:	{guid {D523E511-9A67-4956-9BF5-D1965960274A}}	
OrdinalPosition:	20	
Required:	False	
SourceField:	Linie	
SourceTable:	Eltern	
TextAlign:	General	
Resistenz Bakterien.Resistenz	Text	50
AggregateType:	-1	
AllowZeroLength:	True	
AppendOnly:	False	
Attributes:	Variable Length; Updatable	
CollatingOrder:	General	
ColumnHidden:	False	
ColumnOrder:	Default	
ColumnWidth:	2160	
DataUpdatable:	True	
DisplayControl:	Text Box	
GUID:	{guid {E2D30250-2F7D-4321-8B7F-BF08B22AB9CF}}	
IMEMode:	0	
IMESentenceMode:	3	
OrdinalPosition:	21	
Required:	False	

SourceField:	Resistenz	
SourceTable:	Resistenz Bakterien	
TextAlign:	General	
UnicodeCompression:	True	

LIMS-Sample	Long Integer	4
AggregateType:	-1	
AllowZeroLength:	False	
AppendOnly:	False	
Attributes:	Fixed Size; Updatable	
CollatingOrder:	General	
ColumnHidden:	False	
ColumnOrder:	Default	
ColumnWidth:	Default	
DataUpdatable:	True	
DecimalPlaces:	Auto	
DefaultValue:	0	
DisplayControl:	Text Box	
GUID:	{guid {9805BDD4-B4BE-4462-A759-897F11DBAC0C}}	
OrdinalPosition:	22	
Required:	False	
SourceField:	LIMS-Sample	
SourceTable:	Pflanzentransformation	
TextAlign:	General	

Protokollname	Text	50
AggregateType:	-1	
AllowZeroLength:	True	
AppendOnly:	False	
Attributes:	Variable Length; Updatable	
CollatingOrder:	General	
ColumnHidden:	False	
ColumnOrder:	Default	
ColumnWidth:	4905	
DataUpdatable:	True	
Description:	Eindeutiger Name für das Protokoll	
DisplayControl:	Text Box	
GUID:	{guid {BA35B293-EC1A-433F-A9CA-F649675F83D2}}	
IMEMode:	0	
IMESentenceMode:	3	
OrdinalPosition:	23	
Required:	False	
SourceField:	Protokollname	
SourceTable:	Protokoll	
TextAlign:	General	
UnicodeCompression:	True	

TA	Text	100
AggregateType:	-1	
AllowZeroLength:	True	
AppendOnly:	False	
Attributes:	Variable Length; Updatable	
CollatingOrder:	General	
ColumnHidden:	False	
ColumnOrder:	Default	
ColumnWidth:	Default	

DataUpdatable:	True
Description:	Namen der Personen, die die Agrotransformation gemacht oder die Transformationen umgesetzt haben
DisplayControl:	Text Box
GUID:	{guid {A3206D48-5F98-4A4F-AE0C-8EA15AE16163}}
IMEMode:	0
IMESentenceMode:	3
OrdinalPosition:	24
Required:	False
SourceField:	TA
SourceTable:	Pflanzentransformation
TextAlign:	General
UnicodeCompression:	True

Repetition Long Integer 4

AggregateType:	-1
AllowZeroLength:	False
AppendOnly:	False
Attributes:	Fixed Size; Updatable
CollatingOrder:	General
ColumnHidden:	False
ColumnOrder:	Default
ColumnWidth:	Default
DataUpdatable:	True
DecimalPlaces:	Auto
DefaultValue:	1
DisplayControl:	Text Box
GUID:	{guid {E029C503-EE88-4FD9-A435-387B18ECE3FB}}
OrdinalPosition:	25
Required:	False
SourceField:	Repetition
SourceTable:	Pflanzentransformation
TextAlign:	General

Number of rooted LInes Long Integer 4

AggregateType:	-1
AllowZeroLength:	False
AppendOnly:	False
Attributes:	Fixed Size; Updatable
CollatingOrder:	General
ColumnHidden:	False
ColumnOrder:	Default
ColumnWidth:	2700
DataUpdatable:	True
DecimalPlaces:	Auto
Description:	Number of Lines that made roots (festgelegt 06.05.09)
DisplayControl:	Text Box
GUID:	{guid {740AD5D9-15DC-4816-BF77-8126337931D0}}
OrdinalPosition:	26
Required:	False
SourceField:	Number of rooted LInes
SourceTable:	Pflanzentransformation
TextAlign:	General

Cannoniers Text 255

AggregateType:	-1
AllowZeroLength:	True
AppendOnly:	False
Attributes:	Variable Length; Updatable
CollatingOrder:	General
ColumnHidden:	False
ColumnOrder:	Default
ColumnWidth:	Default
DataUpdatable:	True
Description:	Namen der Personen, die die Schüsse durchgeführt haben
DisplayControl:	Text Box
GUID:	{guid {D8D89C86-F22D-41BD-8FB2-55AA99E7DDB9}}
IMEMode:	0
IMESentenceMode:	3
OrdinalPosition:	27
Required:	False
SourceField:	Cannoniers
SourceTable:	Pflanzentransformation
TextAlign:	General
UnicodeCompression:	True

Canone Text 255

AggregateType:	-1
AllowZeroLength:	True
AppendOnly:	False
Attributes:	Variable Length; Updatable
CollatingOrder:	General
ColumnHidden:	False
ColumnOrder:	Default
ColumnWidth:	Default
DataUpdatable:	True
Description:	Name der Partikelkanone
DisplayControl:	Text Box
GUID:	{guid {04F0873F-9C94-42D7-A6FB-CDA3592F3BDF}}
IMEMode:	0
IMESentenceMode:	3
OrdinalPosition:	28
Required:	False
SourceField:	Canone
SourceTable:	Pflanzentransformation
TextAlign:	General
UnicodeCompression:	True

Number of Shots Long Integer 4

AggregateType:	-1
AllowZeroLength:	False
AppendOnly:	False
Attributes:	Fixed Size; Updatable
CollatingOrder:	General
ColumnHidden:	False
ColumnOrder:	Default
ColumnWidth:	Default
DataUpdatable:	True
DecimalPlaces:	Auto
DisplayControl:	Text Box
GUID:	{guid {3E145FCA-523D-4D35-83C0-84382FDCBEB9}}

OrdinalPosition:	29	
Required:	False	
SourceField:	Number of Shots	
SourceTable:	Pflanzentransformation	
TextAlign:	General	

**PlasmidVolume**	Long Integer	4
AggregateType:	-1	
AllowZeroLength:	False	
AppendOnly:	False	
Attributes:	Fixed Size; Updatable	
CollatingOrder:	General	
ColumnHidden:	False	
ColumnOrder:	Default	
ColumnWidth:	Default	
DataUpdatable:	True	
DecimalPlaces:	Auto	
Description:	Volume of plasmid preparation in ul	
DisplayControl:	Text Box	
GUID:	{guid {3087E22D-2946-4B1A-B695-6586FCB4CFB8}}	
OrdinalPosition:	30	
Required:	False	
SourceField:	PlasmidVolume	
SourceTable:	Pflanzentransformation	
TextAlign:	General	

**PlasmidConcentration**	Long Integer	4
AggregateType:	-1	
AllowZeroLength:	False	
AppendOnly:	False	
Attributes:	Fixed Size; Updatable	
CollatingOrder:	General	
ColumnHidden:	False	
ColumnOrder:	Default	
ColumnWidth:	Default	
DataUpdatable:	True	
DecimalPlaces:	Auto	
Description:	Concentration of DNA in plasmid preparation	
DisplayControl:	Text Box	
GUID:	{guid {3257FE43-DF38-42B2-A4B1-91B6F49D9CEB}}	
OrdinalPosition:	31	
Required:	False	
SourceField:	PlasmidConcentration	
SourceTable:	Pflanzentransformation	
TextAlign:	General	

**Plasmidmapcheckd**	Date/Time	8
AggregateType:	-1	
AllowZeroLength:	False	
AppendOnly:	False	
Attributes:	Fixed Size; Updatable	
CollatingOrder:	General	
ColumnHidden:	False	
ColumnOrder:	Default	
ColumnWidth:	Default	
DataUpdatable:	True	

GUID:	{guid {B13E6BB7-77AB-4F94-80BF-0C90E67DC42B}}	
IMEMode:	0	
IMESentenceMode:	3	
OrdinalPosition:	32	
Required:	False	
ShowDatePicker:	For dates	
SourceField:	Plasmidmapcheckd	
SourceTable:	Pflanzentransformation	
TextAlign:	General	

Plasmidmapcheck	Text	255
AggregateType:	-1	
AllowZeroLength:	True	
AppendOnly:	False	
Attributes:	Variable Length; Updatable	
CollatingOrder:	General	
ColumnHidden:	False	
ColumnOrder:	Default	
ColumnWidth:	Default	
DataUpdatable:	True	
Description:	Name of the responsible person that approved the plasmid map	
DisplayControl:	Text Box	
GUID:	{guid {8CD3C03A-46F3-4F77-A317-2CC2A556BF28}}	
IMEMode:	0	
IMESentenceMode:	3	
OrdinalPosition:	33	
Required:	False	
SourceField:	Plasmidmapcheck	
SourceTable:	Pflanzentransformation	
TextAlign:	General	
UnicodeCompression:	True	

Result_ID	Long Integer	4
AggregateType:	-1	
AllowZeroLength:	False	
AppendOnly:	False	
Attributes:	Fixed Size; Updatable	
CollatingOrder:	General	
ColumnHidden:	False	
ColumnOrder:	Default	
ColumnWidth:	Default	
DataUpdatable:	True	
DecimalPlaces:	Auto	
Description:	Reference to "Result" table	
DisplayControl:	Text Box	
OrdinalPosition:	34	
Required:	False	
SourceField:	Result_ID	
SourceTable:	Pflanzentransformation	
TextAlign:	General	

Table Indexes

Name	Number of Fields

ArbeitsgruppenPflanzentransformation		1
	Clustered:	False
	DistinctCount:	1
	Foreign:	True
	IgnoreNulls:	False
	Name:	ArbeitsgruppenPflanzentransformation
	Primary:	False
	Required:	False
	Unique:	False
	Fields:	
	AG	Ascending
Id Nummer		1
	Clustered:	False
	DistinctCount:	4
	Foreign:	False
	IgnoreNulls:	False
	Name:	Id Nummer
	Primary:	False
	Required:	False
	Unique:	False
	Fields:	
	GVO Nummer	Ascending
Number of rooted LInes		1
	Clustered:	False
	DistinctCount:	3
	Foreign:	False
	IgnoreNulls:	False
	Name:	Number of rooted LInes
	Primary:	False
	Required:	False
	Unique:	False
	Fields:	
	Number of rooted LInes	Ascending
Number of Shots		1
	Clustered:	False
	DistinctCount:	2
	Foreign:	False
	IgnoreNulls:	False
	Name:	Number of Shots
	Primary:	False
	Required:	False
	Unique:	False
	Fields:	
	Number of Shots	Ascending
OperatorPflanzentransformation		1
	Clustered:	False
	DistinctCount:	2
	Foreign:	True
	IgnoreNulls:	False
	Name:	OperatorPflanzentransformation
	Primary:	False
	Required:	False
	Unique:	False

	Fields:	
	Worker	Ascending
PrimaryKey		1
	Clustered:	False
	DistinctCount:	7
	Foreign:	False
	IgnoreNulls:	False
	Name:	PrimaryKey
	Primary:	True
	Required:	True
	Unique:	True
	Fields:	
	ID	Ascending
Result_ID		1
	Clustered:	False
	DistinctCount:	2
	Foreign:	False
	IgnoreNulls:	False
	Name:	Result_ID
	Primary:	False
	Required:	False
	Unique:	False
	Fields:	
	Result_ID	Ascending
ConstructId		1
	Clustered:	False
	DistinctCount:	16
	Foreign:	False
	IgnoreNulls:	False
	Name:	ConstructId
	Primary:	True
	Required:	True
	Unique:	True
	Fields:	
	ConstructId	Ascending
ArbeitsgruppenPflanzentransformation		1
	Clustered:	False
	DistinctCount:	1
	Foreign:	True
	IgnoreNulls:	False
	Name:	ArbeitsgruppenPflanzentransformation
	Primary:	False
	Required:	False
	Unique:	False
	Fields:	
	AG	Ascending
Id Nummer		1
	Clustered:	False
	DistinctCount:	4
	Foreign:	False
	IgnoreNulls:	False
	Name:	Id Nummer
	Primary:	False

	Required:	False
	Unique:	False
	Fields:	
	GVO Nummer	Ascending
Number of rooted LInes		1
	Clustered:	False
	DistinctCount:	3
	Foreign:	False
	IgnoreNulls:	False
	Name:	Number of rooted LInes
	Primary:	False
	Required:	False
	Unique:	False
	Fields:	
	Number of rooted LInes	Ascending
Number of Shots		1
	Clustered:	False
	DistinctCount:	2
	Foreign:	False
	IgnoreNulls:	False
	Name:	Number of Shots
	Primary:	False
	Required:	False
	Unique:	False
	Fields:	
	Number of Shots	Ascending
OperatorPflanzentransformation		1
	Clustered:	False
	DistinctCount:	2
	Foreign:	True
	IgnoreNulls:	False
	Name:	OperatorPflanzentransformation
	Primary:	False
	Required:	False
	Unique:	False
	Fields:	
	Worker	Ascending
PrimaryKey		1
	Clustered:	False
	DistinctCount:	7
	Foreign:	False
	IgnoreNulls:	False
	Name:	PrimaryKey
	Primary:	True
	Required:	True
	Unique:	True
	Fields:	
	ID	Ascending
Result_ID		1
	Clustered:	False
	DistinctCount:	2
	Foreign:	False
	IgnoreNulls:	False

	Name:	Result_ID
	Primary:	False
	Required:	False
	Unique:	False
	Fields:	
	Result_ID	Ascending
ID		1
	Clustered:	False
	DistinctCount:	12
	Foreign:	False
	IgnoreNulls:	False
	Name:	ID
	Primary:	False
	Required:	False
	Unique:	False
	Fields:	
	ID	Ascending
PrimaryKey		1
	Clustered:	False
	DistinctCount:	12
	Foreign:	False
	IgnoreNulls:	False
	Name:	PrimaryKey
	Primary:	True
	Required:	True
	Unique:	True
	Fields:	
	ID	Ascending
ArbeitsgruppenPflanzentransformation		1
	Clustered:	False
	DistinctCount:	1
	Foreign:	True
	IgnoreNulls:	False
	Name:	ArbeitsgruppenPflanzentransformation
	Primary:	False
	Required:	False
	Unique:	False
	Fields:	
	AG	Ascending
Id Nummer		1
	Clustered:	False
	DistinctCount:	4
	Foreign:	False
	IgnoreNulls:	False
	Name:	Id Nummer
	Primary:	False
	Required:	False
	Unique:	False
	Fields:	
	GVO Nummer	Ascending
Number of rooted LInes		1
	Clustered:	False
	DistinctCount:	3

	Foreign:	False
	IgnoreNulls:	False
	Name:	Number of rooted LInes
	Primary:	False
	Required:	False
	Unique:	False
	Fields:	
	Number of rooted LInes	Ascending
Number of Shots		1
	Clustered:	False
	DistinctCount:	2
	Foreign:	False
	IgnoreNulls:	False
	Name:	Number of Shots
	Primary:	False
	Required:	False
	Unique:	False
	Fields:	
	Number of Shots	Ascending
OperatorPflanzentransformation		1
	Clustered:	False
	DistinctCount:	2
	Foreign:	True
	IgnoreNulls:	False
	Name:	OperatorPflanzentransformation
	Primary:	False
	Required:	False
	Unique:	False
	Fields:	
	Worker	Ascending
PrimaryKey		1
	Clustered:	False
	DistinctCount:	7
	Foreign:	False
	IgnoreNulls:	False
	Name:	PrimaryKey
	Primary:	True
	Required:	True
	Unique:	True
	Fields:	
	ID	Ascending
Result_ID		1
	Clustered:	False
	DistinctCount:	2
	Foreign:	False
	IgnoreNulls:	False
	Name:	Result_ID
	Primary:	False
	Required:	False
	Unique:	False
	Fields:	
	Result_ID	Ascending
GMO Nummer		1

	Clustered:	False
	DistinctCount:	15
	Foreign:	False
	IgnoreNulls:	False
	Name:	GMO Nummer
	Primary:	False
	Required:	False
	Unique:	False
	Fields:	
	GMO Nummer	Ascending
ID		1
	Clustered:	False
	DistinctCount:	28
	Foreign:	False
	IgnoreNulls:	False
	Name:	ID
	Primary:	False
	Required:	False
	Unique:	False
	Fields:	
	ID	Ascending
ID_Pflanzenarten_Ref		1
	Clustered:	False
	DistinctCount:	4
	Foreign:	False
	IgnoreNulls:	False
	Name:	ID_Pflanzenarten_Ref
	Primary:	False
	Required:	False
	Unique:	False
	Fields:	
	ID_Pflanzenarten_Ref	Ascending
PrimaryKey		1
	Clustered:	False
	DistinctCount:	28
	Foreign:	False
	IgnoreNulls:	False
	Name:	PrimaryKey
	Primary:	True
	Required:	True
	Unique:	True
	Fields:	
	ID	Ascending
ID		1
	Clustered:	False
	DistinctCount:	15
	Foreign:	False
	IgnoreNulls:	False
	Name:	ID
	Primary:	False
	Required:	False
	Unique:	False

	Fields:	
	ID	Ascending
PrimaryKey		1
	Clustered:	False
	DistinctCount:	15
	Foreign:	False
	IgnoreNulls:	False
	Name:	PrimaryKey
	Primary:	True
	Required:	True
	Unique:	True
	Fields:	
	ID	Ascending
ArbeitsgruppenPflanzentransformation		1
	Clustered:	False
	DistinctCount:	1
	Foreign:	True
	IgnoreNulls:	False
	Name:	ArbeitsgruppenPflanzentransformation
	Primary:	False
	Required:	False
	Unique:	False
	Fields:	
	AG	Ascending
Id Nummer		1
	Clustered:	False
	DistinctCount:	4
	Foreign:	False
	IgnoreNulls:	False
	Name:	Id Nummer
	Primary:	False
	Required:	False
	Unique:	False
	Fields:	
	GVO Nummer	Ascending
Number of rooted LInes		1
	Clustered:	False
	DistinctCount:	3
	Foreign:	False
	IgnoreNulls:	False
	Name:	Number of rooted LInes
	Primary:	False
	Required:	False
	Unique:	False
	Fields:	
	Number of rooted LInes	Ascending
Number of Shots		1
	Clustered:	False
	DistinctCount:	2
	Foreign:	False
	IgnoreNulls:	False
	Name:	Number of Shots
	Primary:	False

	Required:	False
	Unique:	False
	Fields:	
	Number of Shots	Ascending
OperatorPflanzentransformation		1
	Clustered:	False
	DistinctCount:	2
	Foreign:	True
	IgnoreNulls:	False
	Name:	OperatorPflanzentransformation
	Primary:	False
	Required:	False
	Unique:	False
	Fields:	
	Worker	Ascending
PrimaryKey		1
	Clustered:	False
	DistinctCount:	7
	Foreign:	False
	IgnoreNulls:	False
	Name:	PrimaryKey
	Primary:	True
	Required:	True
	Unique:	True
	Fields:	
	ID	Ascending
Result_ID		1
	Clustered:	False
	DistinctCount:	2
	Foreign:	False
	IgnoreNulls:	False
	Name:	Result_ID
	Primary:	False
	Required:	False
	Unique:	False
	Fields:	
	Result_ID	Ascending
PrimaryKey		1
	Clustered:	False
	DistinctCount:	3
	Foreign:	False
	IgnoreNulls:	False
	Name:	PrimaryKey
	Primary:	True
	Required:	True
	Unique:	True
	Fields:	
	Protokoll ID	Ascending
ProtokollArt		1
	Clustered:	False
	DistinctCount:	1
	Foreign:	False
	IgnoreNulls:	False

	Name:	ProtokollArt
	Primary:	False
	Required:	False
	Unique:	False
	Fields:	
	Art	Ascending
ArbeitsgruppenPflanzentransformation		1
	Clustered:	False
	DistinctCount:	1
	Foreign:	True
	IgnoreNulls:	False
	Name:	ArbeitsgruppenPflanzentransformation
	Primary:	False
	Required:	False
	Unique:	False
	Fields:	
	AG	Ascending
Id Nummer		1
	Clustered:	False
	DistinctCount:	4
	Foreign:	False
	IgnoreNulls:	False
	Name:	Id Nummer
	Primary:	False
	Required:	False
	Unique:	False
	Fields:	
	GVO Nummer	Ascending
Number of rooted LInes		1
	Clustered:	False
	DistinctCount:	3
	Foreign:	False
	IgnoreNulls:	False
	Name:	Number of rooted LInes
	Primary:	False
	Required:	False
	Unique:	False
	Fields:	
	Number of rooted LInes	Ascending
Number of Shots		1
	Clustered:	False
	DistinctCount:	2
	Foreign:	False
	IgnoreNulls:	False
	Name:	Number of Shots
	Primary:	False
	Required:	False
	Unique:	False
	Fields:	
	Number of Shots	Ascending
OperatorPflanzentransformation		1
	Clustered:	False
	DistinctCount:	2

Foreign:	True
IgnoreNulls:	False
Name:	OperatorPflanzentransformation
Primary:	False
Required:	False
Unique:	False
Fields:	
Worker	Ascending
PrimaryKey	1
Clustered:	False
DistinctCount:	7
Foreign:	False
IgnoreNulls:	False
Name:	PrimaryKey
Primary:	True
Required:	True
Unique:	True
Fields:	
ID	Ascending
Result_ID	1
Clustered:	False
DistinctCount:	2
Foreign:	False
IgnoreNulls:	False
Name:	Result_ID
Primary:	False
Required:	False
Unique:	False
Fields:	
Result_ID	Ascending

User Permissions

admin	Delete; Read Permissions; Set Permissions; Change Owner, Read Definition; Write Definition; Read Data; Insert Data; Update Data; Delete Data

Group Permissions

Admins	Delete; Read Permissions; Set Permissions; Change Owner, Read Definition; Write Definition; Read Data; Insert Data; Update Data; Delete Data
Users	Delete; Read Permissions; Set Permissions; Change Owner, Read Definition; Write Definition; Read Data; Insert Data; Update Data; Delete Data

Properties

DateCreated:	14.08.2009 11:12:12	DefaultView:	2
DOL:	Long binary data	FilterOnLoad:	False
GUID:	{guid {77882D93-DCAB-4091-A67D-638C66835959}}	LastUpdated:	06.10.2009 11:33:14
MaxRecords:	0	ODBCTimeout:	60
OrderByOn:	False	OrderByOnLoad:	True
Orientation:	Left-to-Right	RecordLocks:	No Locks
RecordsAffected:	0	RecordsetType:	Dynaset
ReturnsRecords:	True	TotalsRow:	False
Type:	0	Updatable:	True

SQL

```
SELECT Pflanzentransformation.ID, Pflanzentransformation.[GVO Nummer], Construct.ConstructName,
Pflanzentransformation.Worker, Pflanzentransformation.AG, Pflanzentransformation.Agrobakterienstamm,
Pflanzentransformation.Pflanze, [Resistenz Pflanze].Resistenz, Pflanzentransformation.Protokoll,
Pflanzentransformation.Transformationsdatum, Pflanzentransformation.Kontaminiert,
Pflanzentransformation.Entsorgt, Pflanzentransformation.[Number of Lines], Pflanzentransformation.Result,
Pflanzentransformation.Enddatum, Pflanzentransformation.Remarks, Eltern.Spezies, Eltern.Varietät, Eltern.WT,
Eltern.[GMO Nummer], Eltern.Linie, [Resistenz Bakterien].Resistenz, Pflanzentransformation.[LIMS-Sample],
Protokoll.Protokollname, Pflanzentransformation.TA, Pflanzentransformation.Repetition,
Pflanzentransformation.[Number of rooted LInes], Pflanzentransformation.Cannoniers,
Pflanzentransformation.Canone, Pflanzentransformation.[Number of Shots],
Pflanzentransformation.PlasmidVolume, Pflanzentransformation.PlasmidConcentration,
Pflanzentransformation.Plasmidmapcheckd, Pflanzentransformation.Plasmidmapcheck,
Pflanzentransformation.Result_ID
FROM [Resistenz Bakterien] RIGHT JOIN ([Resistenz Pflanze] RIGHT JOIN (Construct INNER JOIN (Protokoll
INNER JOIN (Eltern INNER JOIN Pflanzentransformation ON Eltern.ID = Pflanzentransformation.Pflanze) ON
Protokoll.[Protokoll ID] = Pflanzentransformation.Protokoll) ON Construct.ConstructId =
Pflanzentransformation.[GVO Nummer]) ON [Resistenz Pflanze].ID = Construct.[Plant Resistance]) ON
[Resistenz Bakterien].ID = Construct.[M Resistance]
ORDER BY Pflanzentransformation.ID DESC;
```

Columns

Name	Type	Size
ID	Long Integer	4

AggregateType:	-1
AllowZeroLength:	False
AppendOnly:	False
Attributes:	Fixed Size; Auto-Increment; Updatable
CollatingOrder:	General
ColumnHidden:	False
ColumnOrder:	Default
ColumnWidth:	Default
DataUpdatable:	True
GUID:	{guid {491011A1-F233-4D8B-AF6D-E85AC96DC00A}}
OrdinalPosition:	0
Required:	False
SourceField:	ID
SourceTable:	Pflanzentransformation
TextAlign:	General

Name	Type	Size
GVO Nummer	Long Integer	4

AggregateType:	-1	
AllowZeroLength:	False	
AppendOnly:	False	
Attributes:	Fixed Size; Updatable	
CollatingOrder:	General	
ColumnHidden:	False	
ColumnOrder:	Default	
ColumnWidth:	1650	
DataUpdatable:	True	
DecimalPlaces:	Auto	
DisplayControl:	Text Box	
GUID:	{guid {78A41B5D-B257-469B-8FCD-4CB99C084269}}	
OrdinalPosition:	1	
Required:	True	
SourceField:	GVO Nummer	
SourceTable:	Pflanzentransformation	
TextAlign:	General	

ConstructName	Text	255
AggregateType:	-1	
AllowZeroLength:	True	
AppendOnly:	False	
Attributes:	Variable Length; Updatable	
CollatingOrder:	General	
ColumnHidden:	False	
ColumnOrder:	Default	
ColumnWidth:	2700	
DataUpdatable:	True	
DisplayControl:	Text Box	
IMEMode:	0	
IMESentenceMode:	3	
OrdinalPosition:	2	
Required:	False	
SourceField:	ConstructName	
SourceTable:	Construct	
TextAlign:	General	
UnicodeCompression:	False	

Worker	Long Integer	4
AggregateType:	-1	
AllowZeroLength:	False	
AppendOnly:	False	
Attributes:	Fixed Size; Updatable	
CollatingOrder:	General	
ColumnHidden:	False	
ColumnOrder:	Default	
ColumnWidth:	Default	
DataUpdatable:	True	
DecimalPlaces:	Auto	
DisplayControl:	Text Box	
GUID:	{guid {2352BCDA-60BE-487E-B967-3F210FA0BEE0}}	
OrdinalPosition:	3	
Required:	False	
SourceField:	Worker	
SourceTable:	Pflanzentransformation	
TextAlign:	General	

AG	Long Integer	4
AggregateType:	-1	
AllowZeroLength:	False	
AppendOnly:	False	
Attributes:	Fixed Size; Updatable	
CollatingOrder:	General	
ColumnHidden:	False	
ColumnOrder:	Default	
ColumnWidth:	Default	
DataUpdatable:	True	
DecimalPlaces:	Auto	
DisplayControl:	Text Box	
GUID:	{guid {856AEBC1-28B9-4ECD-B43A-F74E08572BEC}}	
OrdinalPosition:	4	
Required:	False	
SourceField:	AG	
SourceTable:	Pflanzentransformation	
TextAlign:	General	
Agrobakterienstamm	Long Integer	4
AggregateType:	-1	
AllowZeroLength:	False	
AppendOnly:	False	
Attributes:	Fixed Size; Updatable	
CollatingOrder:	General	
ColumnHidden:	False	
ColumnOrder:	Default	
ColumnWidth:	2010	
DataUpdatable:	True	
DecimalPlaces:	Auto	
DisplayControl:	Text Box	
GUID:	{guid {F51D32DD-0FB9-410A-9B6B-D205562601B3}}	
OrdinalPosition:	5	
Required:	False	
SourceField:	Agrobakterienstamm	
SourceTable:	Pflanzentransformation	
TextAlign:	General	
Pflanze	Long Integer	4
AggregateType:	-1	
AllowMultipleValues:	False	
AllowValueListEdits:	False	
AllowZeroLength:	False	
AppendOnly:	False	
Attributes:	Fixed Size; Updatable	
BoundColumn:	1	
CollatingOrder:	General	
ColumnCount:	6	
ColumnHeads:	False	
ColumnHidden:	False	
ColumnOrder:	Default	
ColumnWidth:	2520	
ColumnWidths:	0;1830;1650;630;1260;705	
DataUpdatable:	True	
DecimalPlaces:	Auto	
DisplayControl:	Combo Box	

GUID:	{guid {DEC170A3-C295-476F-A829-A424275F639C}}
LimitToList:	True
ListRows:	8
ListWidth:	6075twip
OrdinalPosition:	6
Required:	False
RowSource:	SELECT Eltern.ID, Eltern.Spezies, Eltern.Varietät, Eltern.WT, Eltern.[GMO Nummer], Eltern.Linie FROM Eltern;
RowSourceType:	Table/Query
ShowOnlyRowSourceValues:	False
SourceField:	Pflanze
SourceTable:	Pflanzentransformation
TextAlign:	General

Resistenz Pflanze.Resistenz Text 50

AggregateType:	-1
AllowZeroLength:	True
AppendOnly:	False
Attributes:	Variable Length; Updatable
CollatingOrder:	General
ColumnHidden:	False
ColumnOrder:	Default
ColumnWidth:	Default
DataUpdatable:	True
DisplayControl:	Text Box
GUID:	{guid {8D71BD08-D542-4B6A-8796-02E82F37D7E2}}
IMEMode:	0
IMESentenceMode:	3
OrdinalPosition:	7
Required:	False
SourceField:	Resistenz
SourceTable:	Resistenz Pflanze
TextAlign:	General
UnicodeCompression:	True

Protokoll Long Integer 4

AggregateType:	-1
AllowZeroLength:	False
AppendOnly:	False
Attributes:	Fixed Size; Updatable
CollatingOrder:	General
ColumnHidden:	False
ColumnOrder:	Default
ColumnWidth:	Default
DataUpdatable:	True
DecimalPlaces:	Auto
DisplayControl:	Text Box
GUID:	{guid {66638D24-E387-4387-948D-82A060A41F76}}
OrdinalPosition:	8
Required:	False
SourceField:	Protokoll
SourceTable:	Pflanzentransformation
TextAlign:	General

Transformationsdatum Date/Time 8

AggregateType:	-1
AllowZeroLength:	False
AppendOnly:	False
Attributes:	Fixed Size; Updatable
CollatingOrder:	General
ColumnHidden:	False
ColumnOrder:	Default
ColumnWidth:	2850
DataUpdatable:	True
GUID:	{guid {001E5420-4F1C-4D41-9502-4F9521AA6AF1}}
IMEMode:	0
IMESentenceMode:	3
OrdinalPosition:	9
Required:	False
ShowDatePicker:	For dates
SourceField:	Transformationsdatum
SourceTable:	Pflanzentransformation
TextAlign:	General

Kontaminiert Yes/No 1

AggregateType:	-1
AllowZeroLength:	False
AppendOnly:	False
Attributes:	Fixed Size; Updatable
CollatingOrder:	General
ColumnHidden:	False
ColumnOrder:	Default
ColumnWidth:	Default
DataUpdatable:	True
DisplayControl:	106
Format:	Yes/No
GUID:	{guid {EB0D9BA0-D280-440E-A86B-C50C95043FD0}}
OrdinalPosition:	10
Required:	False
SourceField:	Kontaminiert
SourceTable:	Pflanzentransformation
TextAlign:	General

Entsorgt Yes/No 1

AggregateType:	-1
AllowZeroLength:	False
AppendOnly:	False
Attributes:	Fixed Size; Updatable
CollatingOrder:	General
ColumnHidden:	False
ColumnOrder:	Default
ColumnWidth:	Default
DataUpdatable:	True
DisplayControl:	106
Format:	Yes/No
GUID:	{guid {725C28E0-8B07-4DB8-84A2-2B1F3C9F0516}}
OrdinalPosition:	11
Required:	False
SourceField:	Entsorgt
SourceTable:	Pflanzentransformation
TextAlign:	General

Number of Lines	Long Integer	4

AggregateType:	-1
AllowZeroLength:	False
AppendOnly:	False
Attributes:	Fixed Size; Updatable
CollatingOrder:	General
ColumnHidden:	False
ColumnOrder:	Default
ColumnWidth:	2130
DataUpdatable:	True
DecimalPlaces:	Auto
Description:	Number of Lines that have been cut (festgelegt 06.05.09)
DisplayControl:	Text Box
GUID:	{guid {85595EC7-656A-4467-A6CE-21630FBEB0B4}}
OrdinalPosition:	12
Required:	False
SourceField:	Number of Lines
SourceTable:	Pflanzentransformation
TextAlign:	General

Result	Text	50

AggregateType:	-1
AllowMultipleValues:	False
AllowValueListEdits:	False
AllowZeroLength:	True
AppendOnly:	False
Attributes:	Variable Length; Updatable
BoundColumn:	1
CollatingOrder:	General
ColumnCount:	1
ColumnHeads:	False
ColumnHidden:	False
ColumnOrder:	Default
ColumnWidth:	2280
ColumnWidths:	2385
DataUpdatable:	True
DisplayControl:	Combo Box
GUID:	{guid {678F1CC4-5A06-4A1D-A7D4-BBBE683B3FA1}}
IMEMode:	0
IMESentenceMode:	3
LimitToList:	False
ListRows:	8
ListWidth:	2385twip
OrdinalPosition:	13
Required:	False
RowSource:	"übergeben";"kontaminiert";"zurückgezogen";"entsorgt: kein Kallus";"entsorgt: keine Regeneration";"andere"
RowSourceType:	Value List
ShowOnlyRowSourceValues:	False
SourceField:	Result
SourceTable:	Pflanzentransformation
TextAlign:	General
UnicodeCompression:	True

Enddatum	Date/Time	8

AggregateType:	-1	
AllowZeroLength:	False	
AppendOnly:	False	
Attributes:	Fixed Size; Updatable	
CollatingOrder:	General	
ColumnHidden:	False	
ColumnOrder:	Default	
ColumnWidth:	Default	
DataUpdatable:	True	
GUID:	{guid {9256514F-89E7-4417-895A-7E12A2ABA75E}}	
IMEMode:	0	
IMESentenceMode:	3	
OrdinalPosition:	14	
Required:	False	
ShowDatePicker:	For dates	
SourceField:	Enddatum	
SourceTable:	Pflanzentransformation	
TextAlign:	General	

Remarks	Memo	N/A
AggregateType:	-1	
AllowZeroLength:	True	
AppendOnly:	False	
Attributes:	Variable Length; Updatable	
CollatingOrder:	General	
ColumnHidden:	False	
ColumnOrder:	Default	
ColumnWidth:	Default	
DataUpdatable:	True	
GUID:	{guid {9E3C6856-13CF-4E9C-8E22-8E72D8B91407}}	
IMEMode:	0	
IMESentenceMode:	3	
OrdinalPosition:	15	
Required:	False	
SourceField:	Remarks	
SourceTable:	Pflanzentransformation	
TextAlign:	General	
TextFormat:	Plain Text	
UnicodeCompression:	True	

Spezies	Text	50
AggregateType:	-1	
AllowZeroLength:	True	
AppendOnly:	False	
Attributes:	Variable Length; Updatable	
CollatingOrder:	General	
ColumnHidden:	False	
ColumnOrder:	Default	
ColumnWidth:	3855	
DataUpdatable:	True	
DisplayControl:	Text Box	
GUID:	{guid {FA3945B0-6E6C-423D-8BEE-0935B5FD7526}}	
IMEMode:	0	
IMESentenceMode:	3	
OrdinalPosition:	16	
Required:	False	

SourceField: Spezies
SourceTable: Eltern
TextAlign: General
UnicodeCompression: True

Varietät Text 50

AggregateType: -1
AllowZeroLength: True
AppendOnly: False
Attributes: Variable Length; Updatable
CollatingOrder: General
ColumnHidden: False
ColumnOrder: Default
ColumnWidth: 1695
DataUpdatable: True
DisplayControl: Text Box
GUID: {guid {1AF19923-4CB9-4A37-BDF6-9A285C5D1D00}}
IMEMode: 0
IMESentenceMode: 3
OrdinalPosition: 17
Required: False
SourceField: Varietät
SourceTable: Eltern
TextAlign: General
UnicodeCompression: True

WT Yes/No 1

AggregateType: -1
AllowZeroLength: False
AppendOnly: False
Attributes: Fixed Size; Updatable
CollatingOrder: General
ColumnHidden: False
ColumnOrder: Default
ColumnWidth: Default
DataUpdatable: True
DisplayControl: 106
Format: Yes/No
GUID: {guid {C2BE4715-B4A6-4077-A3F6-D1026FADBAC3}}
OrdinalPosition: 18
Required: False
SourceField: WT
SourceTable: Eltern
TextAlign: General

GMO Nummer Long Integer 4

AggregateType: -1
AllowZeroLength: False
AppendOnly: False
Attributes: Fixed Size; Updatable
CollatingOrder: General
ColumnHidden: False
ColumnOrder: Default
ColumnWidth: Default
DataUpdatable: True
DecimalPlaces: Auto

DisplayControl:	Text Box	
GUID:	{guid {0374CE7E-E119-4264-8426-B9502000A391}}	
OrdinalPosition:	19	
Required:	False	
SourceField:	GMO Nummer	
SourceTable:	Eltern	
TextAlign:	General	

**Linie**	Long Integer	4
AggregateType:	-1	
AllowZeroLength:	False	
AppendOnly:	False	
Attributes:	Fixed Size; Updatable	
CollatingOrder:	General	
ColumnHidden:	False	
ColumnOrder:	Default	
ColumnWidth:	Default	
DataUpdatable:	True	
DecimalPlaces:	Auto	
DisplayControl:	Text Box	
GUID:	{guid {D523E511-9A67-4956-9BF5-D1965960274A}}	
OrdinalPosition:	20	
Required:	False	
SourceField:	Linie	
SourceTable:	Eltern	
TextAlign:	General	

**Resistenz Bakterien.Resistenz**	Text	50
AggregateType:	-1	
AllowZeroLength:	True	
AppendOnly:	False	
Attributes:	Variable Length; Updatable	
CollatingOrder:	General	
ColumnHidden:	False	
ColumnOrder:	Default	
ColumnWidth:	2160	
DataUpdatable:	True	
DisplayControl:	Text Box	
GUID:	{guid {E2D30250-2F7D-4321-8B7F-BF08B22AB9CF}}	
IMEMode:	0	
IMESentenceMode:	3	
OrdinalPosition:	21	
Required:	False	
SourceField:	Resistenz	
SourceTable:	Resistenz Bakterien	
TextAlign:	General	
UnicodeCompression:	True	

**LIMS-Sample**	Long Integer	4
AggregateType:	-1	
AllowZeroLength:	False	
AppendOnly:	False	
Attributes:	Fixed Size; Updatable	
CollatingOrder:	General	
ColumnHidden:	False	
ColumnOrder:	Default	

ColumnWidth:	Default	
DataUpdatable:	True	
DecimalPlaces:	Auto	
DisplayControl:	Text Box	
GUID:	{guid {9805BDD4-B4BE-4462-A759-897F11DBAC0C}}	
OrdinalPosition:	22	
Required:	False	
SourceField:	LIMS-Sample	
SourceTable:	Pflanzentransformation	
TextAlign:	General	

Protokollname	Text	50
AggregateType:	-1	
AllowZeroLength:	True	
AppendOnly:	False	
Attributes:	Variable Length; Updatable	
CollatingOrder:	General	
ColumnHidden:	False	
ColumnOrder:	Default	
ColumnWidth:	4905	
DataUpdatable:	True	
Description:	Eindeutiger Name für das Protokoll	
DisplayControl:	Text Box	
GUID:	{guid {BA35B293-EC1A-433F-A9CA-F649675F83D2}}	
IMEMode:	0	
IMESentenceMode:	3	
OrdinalPosition:	23	
Required:	False	
SourceField:	Protokollname	
SourceTable:	Protokoll	
TextAlign:	General	
UnicodeCompression:	True	

TA	Text	100
AggregateType:	-1	
AllowZeroLength:	True	
AppendOnly:	False	
Attributes:	Variable Length; Updatable	
CollatingOrder:	General	
ColumnHidden:	False	
ColumnOrder:	Default	
ColumnWidth:	Default	
DataUpdatable:	True	
Description:	Namen der Personen, die die Agrotransformation gemacht oder die Transformationen umgesetzt haben	
DisplayControl:	Text Box	
GUID:	{guid {A3206D48-5F98-4A4F-AE0C-8EA15AE16163}}	
IMEMode:	0	
IMESentenceMode:	3	
OrdinalPosition:	24	
Required:	False	
SourceField:	TA	
SourceTable:	Pflanzentransformation	
TextAlign:	General	
UnicodeCompression:	True	

Repetition	Long Integer	4
AggregateType:	-1	
AllowZeroLength:	False	
AppendOnly:	False	
Attributes:	Fixed Size; Updatable	
CollatingOrder:	General	
ColumnHidden:	False	
ColumnOrder:	Default	
ColumnWidth:	Default	
DataUpdatable:	True	
DecimalPlaces:	Auto	
DisplayControl:	Text Box	
GUID:	{guid {E029C503-EE88-4FD9-A435-387B18ECE3FB}}	
OrdinalPosition:	25	
Required:	False	
SourceField:	Repetition	
SourceTable:	Pflanzentransformation	
TextAlign:	General	
Number of rooted LInes	Long Integer	4
AggregateType:	-1	
AllowZeroLength:	False	
AppendOnly:	False	
Attributes:	Fixed Size; Updatable	
CollatingOrder:	General	
ColumnHidden:	False	
ColumnOrder:	Default	
ColumnWidth:	2700	
DataUpdatable:	True	
DecimalPlaces:	Auto	
Description:	Number of Lines that made roots (festgelegt 06.05.09)	
DisplayControl:	Text Box	
GUID:	{guid {740AD5D9-15DC-4816-BF77-8126337931D0}}	
OrdinalPosition:	26	
Required:	False	
SourceField:	Number of rooted LInes	
SourceTable:	Pflanzentransformation	
TextAlign:	General	
Cannoniers	Text	255
AggregateType:	-1	
AllowZeroLength:	True	
AppendOnly:	False	
Attributes:	Variable Length; Updatable	
CollatingOrder:	General	
ColumnHidden:	False	
ColumnOrder:	Default	
ColumnWidth:	Default	
DataUpdatable:	True	
Description:	Namen der Personen, die die Schüsse durchgeführt haben	
DisplayControl:	Text Box	
GUID:	{guid {D8D89C86-F22D-41BD-8FB2-55AA99E7DDB9}}	
IMEMode:	0	
IMESentenceMode:	3	
OrdinalPosition:	27	
Required:	False	

	SourceField:	Cannoniers	
	SourceTable:	Pflanzentransformation	
	TextAlign:	General	
	UnicodeCompression:	True	
Canone		Text	255
	AggregateType:	-1	
	AllowZeroLength:	True	
	AppendOnly:	False	
	Attributes:	Variable Length; Updatable	
	CollatingOrder:	General	
	ColumnHidden:	False	
	ColumnOrder:	Default	
	ColumnWidth:	Default	
	DataUpdatable:	True	
	Description:	Name der Partikelkanone	
	DisplayControl:	Text Box	
	GUID:	{guid {04F0873F-9C94-42D7-A6FB-CDA3592F3BDF}}	
	IMEMode:	0	
	IMESentenceMode:	3	
	OrdinalPosition:	28	
	Required:	False	
	SourceField:	Canone	
	SourceTable:	Pflanzentransformation	
	TextAlign:	General	
	UnicodeCompression:	True	
Number of Shots		Long Integer	4
	AggregateType:	-1	
	AllowZeroLength:	False	
	AppendOnly:	False	
	Attributes:	Fixed Size; Updatable	
	CollatingOrder:	General	
	ColumnHidden:	False	
	ColumnOrder:	Default	
	ColumnWidth:	Default	
	DataUpdatable:	True	
	DecimalPlaces:	Auto	
	DisplayControl:	Text Box	
	GUID:	{guid {3E145FCA-523D-4D35-83C0-84382FDCBEB9}}	
	OrdinalPosition:	29	
	Required:	False	
	SourceField:	Number of Shots	
	SourceTable:	Pflanzentransformation	
	TextAlign:	General	
PlasmidVolume		Long Integer	4
	AggregateType:	-1	
	AllowZeroLength:	False	
	AppendOnly:	False	
	Attributes:	Fixed Size; Updatable	
	CollatingOrder:	General	
	ColumnHidden:	False	
	ColumnOrder:	Default	
	ColumnWidth:	Default	
	DataUpdatable:	True	

DecimalPlaces:	Auto	
Description:	Volume of plasmid preparation in ul	
DisplayControl:	Text Box	
GUID:	{guid {3087E22D-2946-4B1A-B695-6586FCB4CFB8}}	
OrdinalPosition:	30	
Required:	False	
SourceField:	PlasmidVolume	
SourceTable:	Pflanzentransformation	
TextAlign:	General	

PlasmidConcentration	Long Integer	4
AggregateType:	-1	
AllowZeroLength:	False	
AppendOnly:	False	
Attributes:	Fixed Size; Updatable	
CollatingOrder:	General	
ColumnHidden:	False	
ColumnOrder:	Default	
ColumnWidth:	Default	
DataUpdatable:	True	
DecimalPlaces:	Auto	
Description:	Concentration of DNA in plasmid preparation	
DisplayControl:	Text Box	
GUID:	{guid {3257FE43-DF38-42B2-A4B1-91B6F49D9CEB}}	
OrdinalPosition:	31	
Required:	False	
SourceField:	PlasmidConcentration	
SourceTable:	Pflanzentransformation	
TextAlign:	General	

Plasmidmapcheckd	Date/Time	8
AggregateType:	-1	
AllowZeroLength:	False	
AppendOnly:	False	
Attributes:	Fixed Size; Updatable	
CollatingOrder:	General	
ColumnHidden:	False	
ColumnOrder:	Default	
ColumnWidth:	Default	
DataUpdatable:	True	
GUID:	{guid {B13E6BB7-77AB-4F94-80BF-0C90E67DC42B}}	
IMEMode:	0	
IMESentenceMode:	3	
OrdinalPosition:	32	
Required:	False	
ShowDatePicker:	For dates	
SourceField:	Plasmidmapcheckd	
SourceTable:	Pflanzentransformation	
TextAlign:	General	

Plasmidmapcheck	Text	255
AggregateType:	-1	
AllowZeroLength:	True	
AppendOnly:	False	
Attributes:	Variable Length; Updatable	
CollatingOrder:	General	

ColumnHidden: False
ColumnOrder: Default
ColumnWidth: Default
DataUpdatable: True
Description: Name of the responsible person that approved the plasmid map
DisplayControl: Text Box
GUID: {guid {8CD3C03A-46F3-4F77-A317-2CC2A556BF28}}
IMEMode: 0
IMESentenceMode: 3
OrdinalPosition: 33
Required: False
SourceField: Plasmidmapcheck
SourceTable: Pflanzentransformation
TextAlign: General
UnicodeCompression: True

Result_ID Long Integer 4
AggregateType: -1
AllowZeroLength: False
AppendOnly: False
Attributes: Fixed Size; Updatable
CollatingOrder: General
ColumnHidden: False
ColumnOrder: Default
ColumnWidth: Default
DataUpdatable: True
DecimalPlaces: Auto
Description: Reference to "Result" table
DisplayControl: Text Box
OrdinalPosition: 34
Required: False
SourceField: Result_ID
SourceTable: Pflanzentransformation
TextAlign: General

Table Indexes

Name	Number of Fields
ArbeitsgruppenPflanzentransformation	1

Clustered: False
DistinctCount: 1
Foreign: True
IgnoreNulls: False
Name: ArbeitsgruppenPflanzentransformation
Primary: False
Required: False
Unique: False
Fields:
AG Ascending

Id Nummer 1
Clustered: False
DistinctCount: 4
Foreign: False
IgnoreNulls: False

	Name:	Id Nummer
	Primary:	False
	Required:	False
	Unique:	False
	Fields:	
	GVO Nummer	Ascending
Number of rooted LInes		1
	Clustered:	False
	DistinctCount:	3
	Foreign:	False
	IgnoreNulls:	False
	Name:	Number of rooted LInes
	Primary:	False
	Required:	False
	Unique:	False
	Fields:	
	Number of rooted LInes	Ascending
Number of Shots		1
	Clustered:	False
	DistinctCount:	2
	Foreign:	False
	IgnoreNulls:	False
	Name:	Number of Shots
	Primary:	False
	Required:	False
	Unique:	False
	Fields:	
	Number of Shots	Ascending
OperatorPflanzentransformation		1
	Clustered:	False
	DistinctCount:	2
	Foreign:	True
	IgnoreNulls:	False
	Name:	OperatorPflanzentransformation
	Primary:	False
	Required:	False
	Unique:	False
	Fields:	
	Worker	Ascending
PrimaryKey		1
	Clustered:	False
	DistinctCount:	7
	Foreign:	False
	IgnoreNulls:	False
	Name:	PrimaryKey
	Primary:	True
	Required:	True
	Unique:	True
	Fields:	
	ID	Ascending
Result_ID		1
	Clustered:	False
	DistinctCount:	2

	Foreign:	False
	IgnoreNulls:	False
	Name:	Result_ID
	Primary:	False
	Required:	False
	Unique:	False
	Fields:	
	Result_ID	Ascending
ConstructId		1
	Clustered:	False
	DistinctCount:	16
	Foreign:	False
	IgnoreNulls:	False
	Name:	ConstructId
	Primary:	True
	Required:	True
	Unique:	True
	Fields:	
	ConstructId	Ascending
ArbeitsgruppenPflanzentransformation		1
	Clustered:	False
	DistinctCount:	1
	Foreign:	True
	IgnoreNulls:	False
	Name:	ArbeitsgruppenPflanzentransformation
	Primary:	False
	Required:	False
	Unique:	False
	Fields:	
	AG	Ascending
Id Nummer		1
	Clustered:	False
	DistinctCount:	4
	Foreign:	False
	IgnoreNulls:	False
	Name:	Id Nummer
	Primary:	False
	Required:	False
	Unique:	False
	Fields:	
	GVO Nummer	Ascending
Number of rooted LInes		1
	Clustered:	False
	DistinctCount:	3
	Foreign:	False
	IgnoreNulls:	False
	Name:	Number of rooted LInes
	Primary:	False
	Required:	False
	Unique:	False
	Fields:	
	Number of rooted LInes	Ascending
Number of Shots		1

	Clustered:	False
	DistinctCount:	2
	Foreign:	False
	IgnoreNulls:	False
	Name:	Number of Shots
	Primary:	False
	Required:	False
	Unique:	False
	Fields:	
	Number of Shots	Ascending
OperatorPflanzentransformation		1
	Clustered:	False
	DistinctCount:	2
	Foreign:	True
	IgnoreNulls:	False
	Name:	OperatorPflanzentransformation
	Primary:	False
	Required:	False
	Unique:	False
	Fields:	
	Worker	Ascending
PrimaryKey		1
	Clustered:	False
	DistinctCount:	7
	Foreign:	False
	IgnoreNulls:	False
	Name:	PrimaryKey
	Primary:	True
	Required:	True
	Unique:	True
	Fields:	
	ID	Ascending
Result_ID		1
	Clustered:	False
	DistinctCount:	2
	Foreign:	False
	IgnoreNulls:	False
	Name:	Result_ID
	Primary:	False
	Required:	False
	Unique:	False
	Fields:	
	Result_ID	Ascending
ID		1
	Clustered:	False
	DistinctCount:	12
	Foreign:	False
	IgnoreNulls:	False
	Name:	ID
	Primary:	False
	Required:	False
	Unique:	False

	Fields:	
	ID	Ascending
PrimaryKey		1
	Clustered:	False
	DistinctCount:	12
	Foreign:	False
	IgnoreNulls:	False
	Name:	PrimaryKey
	Primary:	True
	Required:	True
	Unique:	True
	Fields:	
	ID	Ascending
ArbeitsgruppenPflanzentransformation		1
	Clustered:	False
	DistinctCount:	1
	Foreign:	True
	IgnoreNulls:	False
	Name:	ArbeitsgruppenPflanzentransformation
	Primary:	False
	Required:	False
	Unique:	False
	Fields:	
	AG	Ascending
Id Nummer		1
	Clustered:	False
	DistinctCount:	4
	Foreign:	False
	IgnoreNulls:	False
	Name:	Id Nummer
	Primary:	False
	Required:	False
	Unique:	False
	Fields:	
	GVO Nummer	Ascending
Number of rooted LInes		1
	Clustered:	False
	DistinctCount:	3
	Foreign:	False
	IgnoreNulls:	False
	Name:	Number of rooted LInes
	Primary:	False
	Required:	False
	Unique:	False
	Fields:	
	Number of rooted LInes	Ascending
Number of Shots		1
	Clustered:	False
	DistinctCount:	2
	Foreign:	False
	IgnoreNulls:	False
	Name:	Number of Shots
	Primary:	False

	Required:	False
	Unique:	False
	Fields:	
	Number of Shots	Ascending
OperatorPflanzentransformation		1
	Clustered:	False
	DistinctCount:	2
	Foreign:	True
	IgnoreNulls:	False
	Name:	OperatorPflanzentransformation
	Primary:	False
	Required:	False
	Unique:	False
	Fields:	
	Worker	Ascending
PrimaryKey		1
	Clustered:	False
	DistinctCount:	7
	Foreign:	False
	IgnoreNulls:	False
	Name:	PrimaryKey
	Primary:	True
	Required:	True
	Unique:	True
	Fields:	
	ID	Ascending
Result_ID		1
	Clustered:	False
	DistinctCount:	2
	Foreign:	False
	IgnoreNulls:	False
	Name:	Result_ID
	Primary:	False
	Required:	False
	Unique:	False
	Fields:	
	Result_ID	Ascending
GMO Nummer		1
	Clustered:	False
	DistinctCount:	15
	Foreign:	False
	IgnoreNulls:	False
	Name:	GMO Nummer
	Primary:	False
	Required:	False
	Unique:	False
	Fields:	
	GMO Nummer	Ascending
ID		1
	Clustered:	False
	DistinctCount:	28
	Foreign:	False
	IgnoreNulls:	False

	Name:	ID
	Primary:	False
	Required:	False
	Unique:	False
	Fields:	
	ID	Ascending
ID_Pflanzenarten_Ref		1
	Clustered:	False
	DistinctCount:	4
	Foreign:	False
	IgnoreNulls:	False
	Name:	ID_Pflanzenarten_Ref
	Primary:	False
	Required:	False
	Unique:	False
	Fields:	
	ID_Pflanzenarten_Ref	Ascending
PrimaryKey		1
	Clustered:	False
	DistinctCount:	28
	Foreign:	False
	IgnoreNulls:	False
	Name:	PrimaryKey
	Primary:	True
	Required:	True
	Unique:	True
	Fields:	
	ID	Ascending
ID		1
	Clustered:	False
	DistinctCount:	15
	Foreign:	False
	IgnoreNulls:	False
	Name:	ID
	Primary:	False
	Required:	False
	Unique:	False
	Fields:	
	ID	Ascending
PrimaryKey		1
	Clustered:	False
	DistinctCount:	15
	Foreign:	False
	IgnoreNulls:	False
	Name:	PrimaryKey
	Primary:	True
	Required:	True
	Unique:	True
	Fields:	
	ID	Ascending
ArbeitsgruppenPflanzentransformation		1
	Clustered:	False
	DistinctCount:	1

Foreign:	True
IgnoreNulls:	False
Name:	ArbeitsgruppenPflanzentransformation
Primary:	False
Required:	False
Unique:	False
Fields:	
AG	Ascending

Id Nummer	1
Clustered:	False
DistinctCount:	4
Foreign:	False
IgnoreNulls:	False
Name:	Id Nummer
Primary:	False
Required:	False
Unique:	False
Fields:	
GVO Nummer	Ascending

Number of rooted LInes	1
Clustered:	False
DistinctCount:	3
Foreign:	False
IgnoreNulls:	False
Name:	Number of rooted LInes
Primary:	False
Required:	False
Unique:	False
Fields:	
Number of rooted LInes	Ascending

Number of Shots	1
Clustered:	False
DistinctCount:	2
Foreign:	False
IgnoreNulls:	False
Name:	Number of Shots
Primary:	False
Required:	False
Unique:	False
Fields:	
Number of Shots	Ascending

OperatorPflanzentransformation	1
Clustered:	False
DistinctCount:	2
Foreign:	True
IgnoreNulls:	False
Name:	OperatorPflanzentransformation
Primary:	False
Required:	False
Unique:	False
Fields:	
Worker	Ascending

PrimaryKey	1

	Clustered:	False
	DistinctCount:	7
	Foreign:	False
	IgnoreNulls:	False
	Name:	PrimaryKey
	Primary:	True
	Required:	True
	Unique:	True
	Fields:	
	ID	Ascending
Result_ID		1
	Clustered:	False
	DistinctCount:	2
	Foreign:	False
	IgnoreNulls:	False
	Name:	Result_ID
	Primary:	False
	Required:	False
	Unique:	False
	Fields:	
	Result_ID	Ascending
PrimaryKey		1
	Clustered:	False
	DistinctCount:	3
	Foreign:	False
	IgnoreNulls:	False
	Name:	PrimaryKey
	Primary:	True
	Required:	True
	Unique:	True
	Fields:	
	Protokoll ID	Ascending
ProtokollArt		1
	Clustered:	False
	DistinctCount:	1
	Foreign:	False
	IgnoreNulls:	False
	Name:	ProtokollArt
	Primary:	False
	Required:	False
	Unique:	False
	Fields:	
	Art	Ascending
ArbeitsgruppenPflanzentransformation		1
	Clustered:	False
	DistinctCount:	1
	Foreign:	True
	IgnoreNulls:	False
	Name:	ArbeitsgruppenPflanzentransformation
	Primary:	False
	Required:	False
	Unique:	False

Fields:	
AG	Ascending
Id Nummer	1
Clustered:	False
DistinctCount:	4
Foreign:	False
IgnoreNulls:	False
Name:	Id Nummer
Primary:	False
Required:	False
Unique:	False
Fields:	
GVO Nummer	Ascending
Number of rooted LInes	1
Clustered:	False
DistinctCount:	3
Foreign:	False
IgnoreNulls:	False
Name:	Number of rooted LInes
Primary:	False
Required:	False
Unique:	False
Fields:	
Number of rooted LInes	Ascending
Number of Shots	1
Clustered:	False
DistinctCount:	2
Foreign:	False
IgnoreNulls:	False
Name:	Number of Shots
Primary:	False
Required:	False
Unique:	False
Fields:	
Number of Shots	Ascending
OperatorPflanzentransformation	1
Clustered:	False
DistinctCount:	2
Foreign:	True
IgnoreNulls:	False
Name:	OperatorPflanzentransformation
Primary:	False
Required:	False
Unique:	False
Fields:	
Worker	Ascending
PrimaryKey	1
Clustered:	False
DistinctCount:	7
Foreign:	False
IgnoreNulls:	False
Name:	PrimaryKey
Primary:	True

Required:	True
Unique:	True
Fields:	
ID	Ascending

Result_ID 1

Clustered:	False
DistinctCount:	2
Foreign:	False
IgnoreNulls:	False
Name:	Result_ID
Primary:	False
Required:	False
Unique:	False
Fields:	
Result_ID	Ascending

User Permissions

admin	Delete; Read Permissions; Set Permissions; Change Owner, Read Definition; Write Definition; Read Data; Insert Data; Update Data; Delete Data

Group Permissions

Admins	Delete; Read Permissions; Set Permissions; Change Owner, Read Definition; Write Definition; Read Data; Insert Data; Update Data; Delete Data
Users	Delete; Read Permissions; Set Permissions; Change Owner, Read Definition; Write Definition; Read Data; Insert Data; Update Data; Delete Data

Properties

DateCreated:	25.06.2009 15:36:36	DefaultView:	2
DOL:	Long binary data	FilterOnLoad:	False
GUID:	{guid {C252427D-0453-4CEA-8647-D815EDBE0565}}	LastUpdated:	06.10.2009 11:31:23
MaxRecords:	0	ODBCTimeout:	60
OrderByOn:	False	OrderByOnLoad:	True
Orientation:	Left-to-Right	RecordLocks:	No Locks
RecordsAffected:	0	RecordsetType:	Dynaset
ReturnsRecords:	True	TotalsRow:	False
Type:	0	Updatable:	True

SQL

```
SELECT Pflanzentransformation.ID, Pflanzentransformation.[GVO Nummer], Construct.ConstructName,
Pflanzentransformation.Worker, Pflanzentransformation.AG, Pflanzentransformation.Agrobakterienstamm,
Pflanzentransformation.Pflanze, [Resistenz Pflanze].Resistenz, Pflanzentransformation.Protokoll,
Pflanzentransformation.Transformationsdatum, Pflanzentransformation.Kontaminiert,
Pflanzentransformation.Entsorgt, Pflanzentransformation.[Number of Lines], Pflanzentransformation.Result,
Pflanzentransformation.Enddatum, Pflanzentransformation.Remarks, Eltern.Spezies, Eltern.Varietät, Eltern.WT,
Eltern.[GMO Nummer], Eltern.Linie, Construct.[M Resistance], Pflanzentransformation.[LIMS-Sample],
Protokoll.Protokollname, Pflanzentransformation.TA, Pflanzentransformation.Repetition,
Pflanzentransformation.[Number of rooted LInes], Pflanzentransformation.Cannoniers,
Pflanzentransformation.Canone, Pflanzentransformation.[Number of Shots],
Pflanzentransformation.PlasmidVolume, Pflanzentransformation.PlasmidConcentration,
PlasmidApproval.Plasmidmapcheckd, PlasmidApproval.Plasmidmapcheck, Pflanzentransformation.[Antibiotic
Concentration], Pflanzentransformation.[Light Intensity], Pflanzentransformation.Result_ID
FROM [Resistenz Pflanze] INNER JOIN (((Protokoll INNER JOIN (Eltern INNER JOIN Pflanzentransformation
ON Eltern.ID = Pflanzentransformation.Pflanze) ON Protokoll.[Protokoll ID] =
Pflanzentransformation.Protokoll) INNER JOIN Construct ON Pflanzentransformation.[GVO Nummer] =
Construct.ConstructId) LEFT JOIN PlasmidApproval ON Construct.ConstructId = PlasmidApproval.ConstructId)
ON [Resistenz Pflanze].ID = Construct.[Plant Resistance]
WHERE (((Pflanzentransformation.Enddatum) Is Null) AND ((Pflanzentransformation.TA) Is Null)) OR
(((Pflanzentransformation.TA) Not Like 'Brigitte Buchwald'))
ORDER BY Pflanzentransformation.ID DESC;
```

Columns

Name	Type	Size
ID	Long Integer	4

AggregateType:	-1
AllowZeroLength:	False
AppendOnly:	False
Attributes:	Fixed Size; Auto-Increment; Updatable
CollatingOrder:	General
ColumnHidden:	False
ColumnOrder:	Default
ColumnWidth:	Default
DataUpdatable:	True
GUID:	{guid {491011A1-F233-4D8B-AF6D-E85AC96DC00A}}
OrdinalPosition:	0
Required:	False
SourceField:	ID
SourceTable:	Pflanzentransformation
TextAlign:	General

GVO Nummer	Long Integer	4
AggregateType:	-1	
AllowZeroLength:	False	
AppendOnly:	False	
Attributes:	Fixed Size; Updatable	
CollatingOrder:	General	
ColumnHidden:	False	
ColumnOrder:	Default	
ColumnWidth:	1650	
DataUpdatable:	True	
DecimalPlaces:	Auto	
DisplayControl:	Text Box	
GUID:	{guid {78A41B5D-B257-469B-8FCD-4CB99C084269}}	
OrdinalPosition:	1	
Required:	True	
SourceField:	GVO Nummer	
SourceTable:	Pflanzentransformation	
TextAlign:	General	
ConstructName	Text	255
AggregateType:	-1	
AllowZeroLength:	True	
AppendOnly:	False	
Attributes:	Variable Length; Updatable	
CollatingOrder:	General	
ColumnHidden:	False	
ColumnOrder:	Default	
ColumnWidth:	2700	
DataUpdatable:	True	
DisplayControl:	Text Box	
IMEMode:	0	
IMESentenceMode:	3	
OrdinalPosition:	2	
Required:	False	
SourceField:	ConstructName	
SourceTable:	Construct	
TextAlign:	General	
UnicodeCompression:	False	
Worker	Long Integer	4
AggregateType:	-1	
AllowZeroLength:	False	
AppendOnly:	False	
Attributes:	Fixed Size; Updatable	
CollatingOrder:	General	
ColumnHidden:	False	
ColumnOrder:	Default	
ColumnWidth:	Default	
DataUpdatable:	True	
DecimalPlaces:	Auto	
DisplayControl:	Text Box	
GUID:	{guid {2352BCDA-60BE-487E-B967-3F210FA0BEE0}}	
OrdinalPosition:	3	
Required:	False	
SourceField:	Worker	
SourceTable:	Pflanzentransformation	

TextAlign: General

AG Long Integer 4

AggregateType:	-1
AllowZeroLength:	False
AppendOnly:	False
Attributes:	Fixed Size; Updatable
CollatingOrder:	General
ColumnHidden:	False
ColumnOrder:	Default
ColumnWidth:	Default
DataUpdatable:	True
DecimalPlaces:	Auto
DisplayControl:	Text Box
GUID:	{guid {856AEBC1-28B9-4ECD-B43A-F74E08572BEC}}
OrdinalPosition:	4
Required:	False
SourceField:	AG
SourceTable:	Pflanzentransformation
TextAlign:	General

Agrobakterienstamm Long Integer 4

AggregateType:	-1
AllowZeroLength:	False
AppendOnly:	False
Attributes:	Fixed Size; Updatable
CollatingOrder:	General
ColumnHidden:	False
ColumnOrder:	Default
ColumnWidth:	2010
DataUpdatable:	True
DecimalPlaces:	Auto
DisplayControl:	Text Box
GUID:	{guid {F51D32DD-0FB9-410A-9B6B-D205562601B3}}
OrdinalPosition:	5
Required:	False
SourceField:	Agrobakterienstamm
SourceTable:	Pflanzentransformation
TextAlign:	General

Pflanze Long Integer 4

AggregateType:	-1
AllowMultipleValues:	False
AllowValueListEdits:	False
AllowZeroLength:	False
AppendOnly:	False
Attributes:	Fixed Size; Updatable
BoundColumn:	1
CollatingOrder:	General
ColumnCount:	6
ColumnHeads:	False
ColumnHidden:	False
ColumnOrder:	Default
ColumnWidth:	2520
ColumnWidths:	0;1830;1650;630;1260;705
DataUpdatable:	True

	DecimalPlaces:	Auto
	DisplayControl:	Combo Box
	GUID:	{guid {DEC170A3-C295-476F-A829-A424275F639C}}
	LimitToList:	True
	ListRows:	8
	ListWidth:	6075twip
	OrdinalPosition:	6
	Required:	False
	RowSource:	SELECT Eltern.ID, Eltern.Spezies, Eltern.Varietät, Eltern.WT, Eltern.[GMO Nummer], Eltern.Linie FROM Eltern;
	RowSourceType:	Table/Query
	ShowOnlyRowSourceValues:	False
	SourceField:	Pflanze
	SourceTable:	Pflanzentransformation
	TextAlign:	General

Resistenz		Text	50
	AggregateType:	-1	
	AllowZeroLength:	True	
	AppendOnly:	False	
	Attributes:	Variable Length; Updatable	
	CollatingOrder:	General	
	ColumnHidden:	False	
	ColumnOrder:	Default	
	ColumnWidth:	Default	
	DataUpdatable:	True	
	DisplayControl:	Text Box	
	GUID:	{guid {8D71BD08-D542-4B6A-8796-02E82F37D7E2}}	
	IMEMode:	0	
	IMESentenceMode:	3	
	OrdinalPosition:	7	
	Required:	False	
	SourceField:	Resistenz	
	SourceTable:	Resistenz Pflanze	
	TextAlign:	General	
	UnicodeCompression:	True	
Protokoll		Long Integer	4
	AggregateType:	-1	
	AllowZeroLength:	False	
	AppendOnly:	False	
	Attributes:	Fixed Size; Updatable	
	CollatingOrder:	General	
	ColumnHidden:	False	
	ColumnOrder:	Default	
	ColumnWidth:	Default	
	DataUpdatable:	True	
	DecimalPlaces:	Auto	
	DisplayControl:	Text Box	
	GUID:	{guid {66638D24-E387-4387-948D-82A060A41F76}}	
	OrdinalPosition:	8	
	Required:	False	
	SourceField:	Protokoll	
	SourceTable:	Pflanzentransformation	
	TextAlign:	General	

Transformationsdatum	Date/Time	8
AggregateType:	-1	
AllowZeroLength:	False	
AppendOnly:	False	
Attributes:	Fixed Size; Updatable	
CollatingOrder:	General	
ColumnHidden:	False	
ColumnOrder:	Default	
ColumnWidth:	2850	
DataUpdatable:	True	
GUID:	{guid {001E5420-4F1C-4D41-9502-4F9521AA6AF1}}	
IMEMode:	0	
IMESentenceMode:	3	
OrdinalPosition:	9	
Required:	False	
ShowDatePicker:	For dates	
SourceField:	Transformationsdatum	
SourceTable:	Pflanzentransformation	
TextAlign:	General	
Kontaminiert	Yes/No	1
AggregateType:	-1	
AllowZeroLength:	False	
AppendOnly:	False	
Attributes:	Fixed Size; Updatable	
CollatingOrder:	General	
ColumnHidden:	False	
ColumnOrder:	Default	
ColumnWidth:	Default	
DataUpdatable:	True	
DisplayControl:	106	
Format:	Yes/No	
GUID:	{guid {EB0D9BA0-D280-440E-A86B-C50C95043FD0}}	
OrdinalPosition:	10	
Required:	False	
SourceField:	Kontaminiert	
SourceTable:	Pflanzentransformation	
TextAlign:	General	
Entsorgt	Yes/No	1
AggregateType:	-1	
AllowZeroLength:	False	
AppendOnly:	False	
Attributes:	Fixed Size; Updatable	
CollatingOrder:	General	
ColumnHidden:	False	
ColumnOrder:	Default	
ColumnWidth:	Default	
DataUpdatable:	True	
DisplayControl:	106	
Format:	Yes/No	
GUID:	{guid {725C28E0-8B07-4DB8-84A2-2B1F3C9F0516}}	
OrdinalPosition:	11	
Required:	False	
SourceField:	Entsorgt	
SourceTable:	Pflanzentransformation	

TextAlign:	General	
**Number of Lines**	Long Integer	4
AggregateType:	-1	
AllowZeroLength:	False	
AppendOnly:	False	
Attributes:	Fixed Size; Updatable	
CollatingOrder:	General	
ColumnHidden:	False	
ColumnOrder:	Default	
ColumnWidth:	2130	
DataUpdatable:	True	
DecimalPlaces:	Auto	
Description:	Number of Lines that have been cut (festgelegt 06.05.09)	
DisplayControl:	Text Box	
GUID:	{guid {85595EC7-656A-4467-A6CE-21630FBEB0B4}}	
OrdinalPosition:	12	
Required:	False	
SourceField:	Number of Lines	
SourceTable:	Pflanzentransformation	
TextAlign:	General	
**Result**	Text	50
AggregateType:	-1	
AllowMultipleValues:	False	
AllowValueListEdits:	False	
AllowZeroLength:	True	
AppendOnly:	False	
Attributes:	Variable Length; Updatable	
BoundColumn:	1	
CollatingOrder:	General	
ColumnCount:	1	
ColumnHeads:	False	
ColumnHidden:	False	
ColumnOrder:	Default	
ColumnWidth:	2280	
ColumnWidths:	2385	
DataUpdatable:	True	
DisplayControl:	Combo Box	
GUID:	{guid {678F1CC4-5A06-4A1D-A7D4-BBBE683B3FA1}}	
IMEMode:	0	
IMESentenceMode:	3	
LimitToList:	False	
ListRows:	8	
ListWidth:	2385twip	
OrdinalPosition:	13	
Required:	False	
RowSource:	"übergeben";"kontaminiert";"zurückgezogen";"entsorgt: kein Kallus";"entsorgt: keine Regeneration";"andere"	
RowSourceType:	Value List	
ShowOnlyRowSourceValues:	False	
SourceField:	Result	
SourceTable:	Pflanzentransformation	
TextAlign:	General	
UnicodeCompression:	True	

Enddatum	Date/Time	8
AggregateType:	-1	
AllowZeroLength:	False	
AppendOnly:	False	
Attributes:	Fixed Size; Updatable	
CollatingOrder:	General	
ColumnHidden:	False	
ColumnOrder:	Default	
ColumnWidth:	Default	
DataUpdatable:	True	
GUID:	{guid {9256514F-89E7-4417-895A-7E12A2ABA75E}}	
IMEMode:	0	
IMESentenceMode:	3	
OrdinalPosition:	14	
Required:	False	
ShowDatePicker:	For dates	
SourceField:	Enddatum	
SourceTable:	Pflanzentransformation	
TextAlign:	General	
Remarks	Memo	N/A
AggregateType:	-1	
AllowZeroLength:	True	
AppendOnly:	False	
Attributes:	Variable Length; Updatable	
CollatingOrder:	General	
ColumnHidden:	False	
ColumnOrder:	Default	
ColumnWidth:	Default	
DataUpdatable:	True	
GUID:	{guid {9E3C6856-13CF-4E9C-8E22-8E72D8B91407}}	
IMEMode:	0	
IMESentenceMode:	3	
OrdinalPosition:	15	
Required:	False	
SourceField:	Remarks	
SourceTable:	Pflanzentransformation	
TextAlign:	General	
TextFormat:	Plain Text	
UnicodeCompression:	True	
Spezies	Text	50
AggregateType:	-1	
AllowZeroLength:	True	
AppendOnly:	False	
Attributes:	Variable Length; Updatable	
CollatingOrder:	General	
ColumnHidden:	False	
ColumnOrder:	Default	
ColumnWidth:	3855	
DataUpdatable:	True	
DisplayControl:	Text Box	
GUID:	{guid {FA3945B0-6E6C-423D-8BEE-0935B5FD7526}}	
IMEMode:	0	
IMESentenceMode:	3	
OrdinalPosition:	16	

Required:	False	
SourceField:	Spezies	
SourceTable:	Eltern	
TextAlign:	General	
UnicodeCompression:	True	

Varietät	Text	50
AggregateType:	-1	
AllowZeroLength:	True	
AppendOnly:	False	
Attributes:	Variable Length; Updatable	
CollatingOrder:	General	
ColumnHidden:	False	
ColumnOrder:	Default	
ColumnWidth:	1695	
DataUpdatable:	True	
DisplayControl:	Text Box	
GUID:	{guid {1AF19923-4CB9-4A37-BDF6-9A285C5D1D00}}	
IMEMode:	0	
IMESentenceMode:	3	
OrdinalPosition:	17	
Required:	False	
SourceField:	Varietät	
SourceTable:	Eltern	
TextAlign:	General	
UnicodeCompression:	True	

WT	Yes/No	1
AggregateType:	-1	
AllowZeroLength:	False	
AppendOnly:	False	
Attributes:	Fixed Size; Updatable	
CollatingOrder:	General	
ColumnHidden:	False	
ColumnOrder:	Default	
ColumnWidth:	Default	
DataUpdatable:	True	
DisplayControl:	106	
Format:	Yes/No	
GUID:	{guid {C2BE4715-B4A6-4077-A3F6-D1026FADBAC3}}	
OrdinalPosition:	18	
Required:	False	
SourceField:	WT	
SourceTable:	Eltern	
TextAlign:	General	

GMO Nummer	Long Integer	4
AggregateType:	-1	
AllowZeroLength:	False	
AppendOnly:	False	
Attributes:	Fixed Size; Updatable	
CollatingOrder:	General	
ColumnHidden:	False	
ColumnOrder:	Default	
ColumnWidth:	Default	
DataUpdatable:	True	

	DecimalPlaces:	Auto	
	DisplayControl:	Text Box	
	GUID:	{guid {0374CE7E-E119-4264-8426-B9502000A391}}	
	OrdinalPosition:	19	
	Required:	False	
	SourceField:	GMO Nummer	
	SourceTable:	Eltern	
	TextAlign:	General	
Linie		Long Integer	4
	AggregateType:	-1	
	AllowZeroLength:	False	
	AppendOnly:	False	
	Attributes:	Fixed Size; Updatable	
	CollatingOrder:	General	
	ColumnHidden:	False	
	ColumnOrder:	Default	
	ColumnWidth:	Default	
	DataUpdatable:	True	
	DecimalPlaces:	Auto	
	DisplayControl:	Text Box	
	GUID:	{guid {D523E511-9A67-4956-9BF5-D1965960274A}}	
	OrdinalPosition:	20	
	Required:	False	
	SourceField:	Linie	
	SourceTable:	Eltern	
	TextAlign:	General	
M Resistance		Long Integer	4
	AggregateType:	-1	
	AllowZeroLength:	False	
	AppendOnly:	False	
	Attributes:	Fixed Size; Updatable	
	CollatingOrder:	General	
	ColumnHidden:	False	
	ColumnOrder:	Default	
	ColumnWidth:	1635	
	DataUpdatable:	True	
	DecimalPlaces:	Auto	
	DisplayControl:	Text Box	
	OrdinalPosition:	21	
	Required:	False	
	SourceField:	M Resistance	
	SourceTable:	Construct	
	TextAlign:	General	
LIMS-Sample		Long Integer	4
	AggregateType:	-1	
	AllowZeroLength:	False	
	AppendOnly:	False	
	Attributes:	Fixed Size; Updatable	
	CollatingOrder:	General	
	ColumnHidden:	False	
	ColumnOrder:	Default	
	ColumnWidth:	Default	
	DataUpdatable:	True	

DecimalPlaces:	Auto
DisplayControl:	Text Box
GUID:	{guid {9805BDD4-B4BE-4462-A759-897F11DBAC0C}}
OrdinalPosition:	22
Required:	False
SourceField:	LIMS-Sample
SourceTable:	Pflanzentransformation
TextAlign:	General

Protokollname Text 50

AggregateType:	-1
AllowZeroLength:	True
AppendOnly:	False
Attributes:	Variable Length; Updatable
CollatingOrder:	General
ColumnHidden:	False
ColumnOrder:	Default
ColumnWidth:	4905
DataUpdatable:	True
Description:	Eindeutiger Name für das Protokoll
DisplayControl:	Text Box
GUID:	{guid {BA35B293-EC1A-433F-A9CA-F649675F83D2}}
IMEMode:	0
IMESentenceMode:	3
OrdinalPosition:	23
Required:	False
SourceField:	Protokollname
SourceTable:	Protokoll
TextAlign:	General
UnicodeCompression:	True

TA Text 100

AggregateType:	-1
AllowZeroLength:	True
AppendOnly:	False
Attributes:	Variable Length; Updatable
CollatingOrder:	General
ColumnHidden:	False
ColumnOrder:	Default
ColumnWidth:	1950
DataUpdatable:	True
Description:	Namen der Personen, die die Agrotransformation gemacht oder die Transformationen umgesetzt haben
DisplayControl:	Text Box
GUID:	{guid {A3206D48-5F98-4A4F-AE0C-8EA15AE16163}}
IMEMode:	0
IMESentenceMode:	3
OrdinalPosition:	24
Required:	False
SourceField:	TA
SourceTable:	Pflanzentransformation
TextAlign:	General
UnicodeCompression:	True

Repetition Long Integer 4

AggregateType:	-1
AllowZeroLength:	False
AppendOnly:	False
Attributes:	Fixed Size; Updatable
CollatingOrder:	General
ColumnHidden:	False
ColumnOrder:	Default
ColumnWidth:	Default
DataUpdatable:	True
DecimalPlaces:	Auto
DisplayControl:	Text Box
GUID:	{guid {E029C503-EE88-4FD9-A435-387B18ECE3FB}}
OrdinalPosition:	25
Required:	False
SourceField:	Repetition
SourceTable:	Pflanzentransformation
TextAlign:	General

Number of rooted LInes — Long Integer — 4

AggregateType:	-1
AllowZeroLength:	False
AppendOnly:	False
Attributes:	Fixed Size; Updatable
CollatingOrder:	General
ColumnHidden:	False
ColumnOrder:	Default
ColumnWidth:	2700
DataUpdatable:	True
DecimalPlaces:	Auto
Description:	Number of Lines that made roots (festgelegt 06.05.09)
DisplayControl:	Text Box
GUID:	{guid {740AD5D9-15DC-4816-BF77-8126337931D0}}
OrdinalPosition:	26
Required:	False
SourceField:	Number of rooted LInes
SourceTable:	Pflanzentransformation
TextAlign:	General

Cannoniers — Text — 255

AggregateType:	-1
AllowZeroLength:	True
AppendOnly:	False
Attributes:	Variable Length; Updatable
CollatingOrder:	General
ColumnHidden:	False
ColumnOrder:	Default
ColumnWidth:	Default
DataUpdatable:	True
Description:	Namen der Personen, die die Schüsse durchgeführt haben
DisplayControl:	Text Box
GUID:	{guid {D8D89C86-F22D-41BD-8FB2-55AA99E7DDB9}}
IMEMode:	0
IMESentenceMode:	3
OrdinalPosition:	27
Required:	False
SourceField:	Cannoniers

SourceTable: Pflanzentransformation
TextAlign: General
UnicodeCompression: True

Canone Text 255
AggregateType: -1
AllowZeroLength: True
AppendOnly: False
Attributes: Variable Length; Updatable
CollatingOrder: General
ColumnHidden: False
ColumnOrder: Default
ColumnWidth: Default
DataUpdatable: True
Description: Name der Partikelkanone
DisplayControl: Text Box
GUID: {guid {04F0873F-9C94-42D7-A6FB-CDA3592F3BDF}}
IMEMode: 0
IMESentenceMode: 3
OrdinalPosition: 28
Required: False
SourceField: Canone
SourceTable: Pflanzentransformation
TextAlign: General
UnicodeCompression: True

Number of Shots Long Integer 4
AggregateType: -1
AllowZeroLength: False
AppendOnly: False
Attributes: Fixed Size; Updatable
CollatingOrder: General
ColumnHidden: False
ColumnOrder: Default
ColumnWidth: Default
DataUpdatable: True
DecimalPlaces: Auto
DisplayControl: Text Box
GUID: {guid {3E145FCA-523D-4D35-83C0-84382FDCBEB9}}
OrdinalPosition: 29
Required: False
SourceField: Number of Shots
SourceTable: Pflanzentransformation
TextAlign: General

PlasmidVolume Long Integer 4
AggregateType: -1
AllowZeroLength: False
AppendOnly: False
Attributes: Fixed Size; Updatable
CollatingOrder: General
ColumnHidden: False
ColumnOrder: Default
ColumnWidth: Default
DataUpdatable: True
DecimalPlaces: Auto

Description:	Volume of plasmid preparation in ul	
DisplayControl:	Text Box	
GUID:	{guid {3087E22D-2946-4B1A-B695-6586FCB4CFB8}}	
OrdinalPosition:	30	
Required:	False	
SourceField:	PlasmidVolume	
SourceTable:	Pflanzentransformation	
TextAlign:	General	

PlasmidConcentration	Long Integer	4
AggregateType:	-1	
AllowZeroLength:	False	
AppendOnly:	False	
Attributes:	Fixed Size; Updatable	
CollatingOrder:	General	
ColumnHidden:	False	
ColumnOrder:	Default	
ColumnWidth:	Default	
DataUpdatable:	True	
DecimalPlaces:	Auto	
Description:	Concentration of DNA in plasmid preparation	
DisplayControl:	Text Box	
GUID:	{guid {3257FE43-DF38-42B2-A4B1-91B6F49D9CEB}}	
OrdinalPosition:	31	
Required:	False	
SourceField:	PlasmidConcentration	
SourceTable:	Pflanzentransformation	
TextAlign:	General	

Plasmidmapcheckd	Date/Time	8
AggregateType:	-1	
AllowZeroLength:	False	
AppendOnly:	False	
Attributes:	Fixed Size; Updatable	
CollatingOrder:	General	
ColumnHidden:	False	
ColumnOrder:	Default	
ColumnWidth:	2205	
DataUpdatable:	True	
GUID:	{guid {1CB9781E-482D-45DD-B68F-7FCDEE13E2E2}}	
IMEMode:	0	
IMESentenceMode:	3	
OrdinalPosition:	32	
Required:	False	
ShowDatePicker:	For dates	
SourceField:	Plasmidmapcheckd	
SourceTable:	PlasmidApproval	
TextAlign:	General	

Plasmidmapcheck	Text	255
AggregateType:	-1	
AllowZeroLength:	True	
AppendOnly:	False	
Attributes:	Variable Length; Updatable	
CollatingOrder:	General	
ColumnHidden:	False	

ColumnOrder:	Default
ColumnWidth:	2085
DataUpdatable:	True
DisplayControl:	Text Box
GUID:	{guid {F34DE3DD-1073-4E8D-9FC7-718DE366FF6D}}
IMEMode:	0
IMESentenceMode:	3
OrdinalPosition:	33
Required:	False
SourceField:	Plasmidmapcheck
SourceTable:	PlasmidApproval
TextAlign:	General
UnicodeCompression:	True

Antibiotic Concentration Long Integer 4

AggregateType:	-1
AllowZeroLength:	False
AppendOnly:	False
Attributes:	Fixed Size; Updatable
CollatingOrder:	General
ColumnHidden:	False
ColumnOrder:	Default
ColumnWidth:	Default
DataUpdatable:	True
DecimalPlaces:	Auto
Description:	Concentration of "Resitenzmarker Pflanze" in µg/ml
DisplayControl:	Text Box
OrdinalPosition:	34
Required:	False
SourceField:	Antibiotic Concentration
SourceTable:	Pflanzentransformation
TextAlign:	General

Light Intensity Long Integer 4

AggregateType:	-1
AllowZeroLength:	False
AppendOnly:	False
Attributes:	Fixed Size; Updatable
CollatingOrder:	General
ColumnHidden:	False
ColumnOrder:	Default
ColumnWidth:	Default
DataUpdatable:	True
DecimalPlaces:	Auto
Description:	in µmol m-2 s-1
DisplayControl:	Text Box
OrdinalPosition:	35
Required:	False
SourceField:	Light Intensity
SourceTable:	Pflanzentransformation
TextAlign:	General

Result_ID Long Integer 4

AggregateType:	-1
AllowZeroLength:	False
AppendOnly:	False

Attributes:	Fixed Size; Updatable
CollatingOrder:	General
ColumnHidden:	False
ColumnOrder:	Default
ColumnWidth:	Default
DataUpdatable:	True
DecimalPlaces:	Auto
Description:	Reference to "Result" table
DisplayControl:	Text Box
OrdinalPosition:	36
Required:	False
SourceField:	Result_ID
SourceTable:	Pflanzentransformation
TextAlign:	General

Table Indexes

Name		Number of Fields
ArbeitsgruppenPflanzentransformation		1
	Clustered:	False
	DistinctCount:	1
	Foreign:	True
	IgnoreNulls:	False
	Name:	ArbeitsgruppenPflanzentransformation
	Primary:	False
	Required:	False
	Unique:	False
	Fields:	
	AG	Ascending
Id Nummer		1
	Clustered:	False
	DistinctCount:	4
	Foreign:	False
	IgnoreNulls:	False
	Name:	Id Nummer
	Primary:	False
	Required:	False
	Unique:	False
	Fields:	
	GVO Nummer	Ascending
Number of rooted LInes		1
	Clustered:	False
	DistinctCount:	3
	Foreign:	False
	IgnoreNulls:	False
	Name:	Number of rooted LInes
	Primary:	False
	Required:	False
	Unique:	False
	Fields:	
	Number of rooted LInes	Ascending
Number of Shots		1
	Clustered:	False

	DistinctCount:	2
	Foreign:	False
	IgnoreNulls:	False
	Name:	Number of Shots
	Primary:	False
	Required:	False
	Unique:	False
	Fields:	
	Number of Shots	Ascending
OperatorPflanzentransformation		1
	Clustered:	False
	DistinctCount:	2
	Foreign:	True
	IgnoreNulls:	False
	Name:	OperatorPflanzentransformation
	Primary:	False
	Required:	False
	Unique:	False
	Fields:	
	Worker	Ascending
PrimaryKey		1
	Clustered:	False
	DistinctCount:	7
	Foreign:	False
	IgnoreNulls:	False
	Name:	PrimaryKey
	Primary:	True
	Required:	True
	Unique:	True
	Fields:	
	ID	Ascending
Result_ID		1
	Clustered:	False
	DistinctCount:	2
	Foreign:	False
	IgnoreNulls:	False
	Name:	Result_ID
	Primary:	False
	Required:	False
	Unique:	False
	Fields:	
	Result_ID	Ascending
ConstructId		1
	Clustered:	False
	DistinctCount:	16
	Foreign:	False
	IgnoreNulls:	False
	Name:	ConstructId
	Primary:	True
	Required:	True
	Unique:	True
	Fields:	
	ConstructId	Ascending

ArbeitsgruppenPflanzentransformation		1
	Clustered:	False
	DistinctCount:	1
	Foreign:	True
	IgnoreNulls:	False
	Name:	ArbeitsgruppenPflanzentransformation
	Primary:	False
	Required:	False
	Unique:	False
	Fields:	
	AG	Ascending
Id Nummer		1
	Clustered:	False
	DistinctCount:	4
	Foreign:	False
	IgnoreNulls:	False
	Name:	Id Nummer
	Primary:	False
	Required:	False
	Unique:	False
	Fields:	
	GVO Nummer	Ascending
Number of rooted LInes		1
	Clustered:	False
	DistinctCount:	3
	Foreign:	False
	IgnoreNulls:	False
	Name:	Number of rooted LInes
	Primary:	False
	Required:	False
	Unique:	False
	Fields:	
	Number of rooted LInes	Ascending
Number of Shots		1
	Clustered:	False
	DistinctCount:	2
	Foreign:	False
	IgnoreNulls:	False
	Name:	Number of Shots
	Primary:	False
	Required:	False
	Unique:	False
	Fields:	
	Number of Shots	Ascending
OperatorPflanzentransformation		1
	Clustered:	False
	DistinctCount:	2
	Foreign:	True
	IgnoreNulls:	False
	Name:	OperatorPflanzentransformation
	Primary:	False
	Required:	False
	Unique:	False

	Fields:	
	Worker	Ascending
PrimaryKey		1
	Clustered:	False
	DistinctCount:	7
	Foreign:	False
	IgnoreNulls:	False
	Name:	PrimaryKey
	Primary:	True
	Required:	True
	Unique:	True
	Fields:	
	ID	Ascending
Result_ID		1
	Clustered:	False
	DistinctCount:	2
	Foreign:	False
	IgnoreNulls:	False
	Name:	Result_ID
	Primary:	False
	Required:	False
	Unique:	False
	Fields:	
	Result_ID	Ascending
ID		1
	Clustered:	False
	DistinctCount:	12
	Foreign:	False
	IgnoreNulls:	False
	Name:	ID
	Primary:	False
	Required:	False
	Unique:	False
	Fields:	
	ID	Ascending
PrimaryKey		1
	Clustered:	False
	DistinctCount:	12
	Foreign:	False
	IgnoreNulls:	False
	Name:	PrimaryKey
	Primary:	True
	Required:	True
	Unique:	True
	Fields:	
	ID	Ascending
ArbeitsgruppenPflanzentransformation		1
	Clustered:	False
	DistinctCount:	1
	Foreign:	True
	IgnoreNulls:	False
	Name:	ArbeitsgruppenPflanzentransformation
	Primary:	False

	Required:	False
	Unique:	False
	Fields:	
	AG	Ascending
Id Nummer		1
	Clustered:	False
	DistinctCount:	4
	Foreign:	False
	IgnoreNulls:	False
	Name:	Id Nummer
	Primary:	False
	Required:	False
	Unique:	False
	Fields:	
	GVO Nummer	Ascending
Number of rooted LInes		1
	Clustered:	False
	DistinctCount:	3
	Foreign:	False
	IgnoreNulls:	False
	Name:	Number of rooted LInes
	Primary:	False
	Required:	False
	Unique:	False
	Fields:	
	Number of rooted LInes	Ascending
Number of Shots		1
	Clustered:	False
	DistinctCount:	2
	Foreign:	False
	IgnoreNulls:	False
	Name:	Number of Shots
	Primary:	False
	Required:	False
	Unique:	False
	Fields:	
	Number of Shots	Ascending
OperatorPflanzentransformation		1
	Clustered:	False
	DistinctCount:	2
	Foreign:	True
	IgnoreNulls:	False
	Name:	OperatorPflanzentransformation
	Primary:	False
	Required:	False
	Unique:	False
	Fields:	
	Worker	Ascending
PrimaryKey		1
	Clustered:	False
	DistinctCount:	7
	Foreign:	False
	IgnoreNulls:	False

	Name:	PrimaryKey
	Primary:	True
	Required:	True
	Unique:	True
	Fields:	
	ID	Ascending
Result_ID		1
	Clustered:	False
	DistinctCount:	2
	Foreign:	False
	IgnoreNulls:	False
	Name:	Result_ID
	Primary:	False
	Required:	False
	Unique:	False
	Fields:	
	Result_ID	Ascending
GMO Nummer		1
	Clustered:	False
	DistinctCount:	15
	Foreign:	False
	IgnoreNulls:	False
	Name:	GMO Nummer
	Primary:	False
	Required:	False
	Unique:	False
	Fields:	
	GMO Nummer	Ascending
ID		1
	Clustered:	False
	DistinctCount:	28
	Foreign:	False
	IgnoreNulls:	False
	Name:	ID
	Primary:	False
	Required:	False
	Unique:	False
	Fields:	
	ID	Ascending
ID_Pflanzenarten_Ref		1
	Clustered:	False
	DistinctCount:	4
	Foreign:	False
	IgnoreNulls:	False
	Name:	ID_Pflanzenarten_Ref
	Primary:	False
	Required:	False
	Unique:	False
	Fields:	
	ID_Pflanzenarten_Ref	Ascending
PrimaryKey		1
	Clustered:	False
	DistinctCount:	28

	Foreign:	False
	IgnoreNulls:	False
	Name:	PrimaryKey
	Primary:	True
	Required:	True
	Unique:	True
	Fields:	
	ID	Ascending
ConstructId		1
	Clustered:	False
	DistinctCount:	16
	Foreign:	False
	IgnoreNulls:	False
	Name:	ConstructId
	Primary:	True
	Required:	True
	Unique:	True
	Fields:	
	ConstructId	Ascending
ArbeitsgruppenPflanzentransformation		1
	Clustered:	False
	DistinctCount:	1
	Foreign:	True
	IgnoreNulls:	False
	Name:	ArbeitsgruppenPflanzentransformation
	Primary:	False
	Required:	False
	Unique:	False
	Fields:	
	AG	Ascending
Id Nummer		1
	Clustered:	False
	DistinctCount:	4
	Foreign:	False
	IgnoreNulls:	False
	Name:	Id Nummer
	Primary:	False
	Required:	False
	Unique:	False
	Fields:	
	GVO Nummer	Ascending
Number of rooted LInes		1
	Clustered:	False
	DistinctCount:	3
	Foreign:	False
	IgnoreNulls:	False
	Name:	Number of rooted LInes
	Primary:	False
	Required:	False
	Unique:	False
	Fields:	
	Number of rooted LInes	Ascending
Number of Shots		1

	Clustered:	False
	DistinctCount:	2
	Foreign:	False
	IgnoreNulls:	False
	Name:	Number of Shots
	Primary:	False
	Required:	False
	Unique:	False
	Fields:	
	Number of Shots	Ascending
OperatorPflanzentransformation		1
	Clustered:	False
	DistinctCount:	2
	Foreign:	True
	IgnoreNulls:	False
	Name:	OperatorPflanzentransformation
	Primary:	False
	Required:	False
	Unique:	False
	Fields:	
	Worker	Ascending
PrimaryKey		1
	Clustered:	False
	DistinctCount:	7
	Foreign:	False
	IgnoreNulls:	False
	Name:	PrimaryKey
	Primary:	True
	Required:	True
	Unique:	True
	Fields:	
	ID	Ascending
Result_ID		1
	Clustered:	False
	DistinctCount:	2
	Foreign:	False
	IgnoreNulls:	False
	Name:	Result_ID
	Primary:	False
	Required:	False
	Unique:	False
	Fields:	
	Result_ID	Ascending
PrimaryKey		1
	Clustered:	False
	DistinctCount:	3
	Foreign:	False
	IgnoreNulls:	False
	Name:	PrimaryKey
	Primary:	True
	Required:	True
	Unique:	True

Fields:	
Protokoll ID	Ascending
ProtokollArt	1
Clustered:	False
DistinctCount:	1
Foreign:	False
IgnoreNulls:	False
Name:	ProtokollArt
Primary:	False
Required:	False
Unique:	False
Fields:	
Art	Ascending
ArbeitsgruppenPflanzentransformation	1
Clustered:	False
DistinctCount:	1
Foreign:	True
IgnoreNulls:	False
Name:	ArbeitsgruppenPflanzentransformation
Primary:	False
Required:	False
Unique:	False
Fields:	
AG	Ascending
Id Nummer	1
Clustered:	False
DistinctCount:	4
Foreign:	False
IgnoreNulls:	False
Name:	Id Nummer
Primary:	False
Required:	False
Unique:	False
Fields:	
GVO Nummer	Ascending
Number of rooted LInes	1
Clustered:	False
DistinctCount:	3
Foreign:	False
IgnoreNulls:	False
Name:	Number of rooted LInes
Primary:	False
Required:	False
Unique:	False
Fields:	
Number of rooted LInes	Ascending
Number of Shots	1
Clustered:	False
DistinctCount:	2
Foreign:	False
IgnoreNulls:	False
Name:	Number of Shots
Primary:	False

	Required:	False
	Unique:	False
	Fields:	
	Number of Shots	Ascending
OperatorPflanzentransformation		1
	Clustered:	False
	DistinctCount:	2
	Foreign:	True
	IgnoreNulls:	False
	Name:	OperatorPflanzentransformation
	Primary:	False
	Required:	False
	Unique:	False
	Fields:	
	Worker	Ascending
PrimaryKey		1
	Clustered:	False
	DistinctCount:	7
	Foreign:	False
	IgnoreNulls:	False
	Name:	PrimaryKey
	Primary:	True
	Required:	True
	Unique:	True
	Fields:	
	ID	Ascending
Result_ID		1
	Clustered:	False
	DistinctCount:	2
	Foreign:	False
	IgnoreNulls:	False
	Name:	Result_ID
	Primary:	False
	Required:	False
	Unique:	False
	Fields:	
	Result_ID	Ascending
ConstructId		1
	Clustered:	False
	DistinctCount:	6
	Foreign:	False
	IgnoreNulls:	False
	Name:	ConstructId
	Primary:	False
	Required:	False
	Unique:	False
	Fields:	
	ConstructId	Ascending
PrimaryKey		1
	Clustered:	False
	DistinctCount:	6
	Foreign:	False
	IgnoreNulls:	False

Name:	PrimaryKey
Primary:	True
Required:	True
Unique:	True
Fields:	
ConstructId	Ascending

ArbeitsgruppenPflanzentransformation 1

Clustered:	False
DistinctCount:	1
Foreign:	True
IgnoreNulls:	False
Name:	ArbeitsgruppenPflanzentransformation
Primary:	False
Required:	False
Unique:	False
Fields:	
AG	Ascending

Id Nummer 1

Clustered:	False
DistinctCount:	4
Foreign:	False
IgnoreNulls:	False
Name:	Id Nummer
Primary:	False
Required:	False
Unique:	False
Fields:	
GVO Nummer	Ascending

Number of rooted LInes 1

Clustered:	False
DistinctCount:	3
Foreign:	False
IgnoreNulls:	False
Name:	Number of rooted LInes
Primary:	False
Required:	False
Unique:	False
Fields:	
Number of rooted LInes	Ascending

Number of Shots 1

Clustered:	False
DistinctCount:	2
Foreign:	False
IgnoreNulls:	False
Name:	Number of Shots
Primary:	False
Required:	False
Unique:	False
Fields:	
Number of Shots	Ascending

OperatorPflanzentransformation 1

Clustered:	False
DistinctCount:	2

	Foreign:	True
	IgnoreNulls:	False
	Name:	OperatorPflanzentransformation
	Primary:	False
	Required:	False
	Unique:	False
	Fields:	
	Worker	Ascending
PrimaryKey		1
	Clustered:	False
	DistinctCount:	7
	Foreign:	False
	IgnoreNulls:	False
	Name:	PrimaryKey
	Primary:	True
	Required:	True
	Unique:	True
	Fields:	
	ID	Ascending
Result_ID		1
	Clustered:	False
	DistinctCount:	2
	Foreign:	False
	IgnoreNulls:	False
	Name:	Result_ID
	Primary:	False
	Required:	False
	Unique:	False
	Fields:	
	Result_ID	Ascending

User Permissions

admin	Delete; Read Permissions; Set Permissions; Change Owner, Read Definition; Write Definition; Read Data; Insert Data; Update Data; Delete Data

Group Permissions

Admins	Delete; Read Permissions; Set Permissions; Change Owner, Read Definition; Write Definition; Read Data; Insert Data; Update Data; Delete Data
Users	Delete; Read Permissions; Set Permissions; Change Owner, Read Definition; Write Definition; Read Data; Insert Data; Update Data; Delete Data

Properties

DateCreated:	15.05.2009 14:08:11	DefaultView:	2
DOL:	Long binary data	FilterOnLoad:	False
GUID:	{guid {FFDC9ECD-334D-461F-9435-98CFA7164B16}}	LastUpdated:	25.09.2009 16:07:27
MaxRecords:	0	ODBCTimeout:	60
OrderByOn:	False	OrderByOnLoad:	True
Orientation:	Left-to-Right	RecordLocks:	No Locks
RecordsAffected:	0	RecordsetType:	Dynaset
ReturnsRecords:	True	TotalsRow:	False
Type:	0	Updatable:	True

SQL

```
SELECT Pflanzentransformation.ID, Pflanzentransformation.[GVO Nummer], Construct.ConstructName AS
Konstruktname, Operator.Operator, Arbeitsgruppen.Arbeitsgruppen, Agrobakterien.Agrobakterinstamm,
[Resistenz Bakterien].Resistenz, [Resistenz Pflanze].Resistenz, Eltern.Spezies, Eltern.Varietät, Eltern.WT,
Eltern.[GMO Nummer], Eltern.Linie, Pflanzentransformation.Transformationsdatum,
Pflanzentransformation.Result, Pflanzentransformation.Enddatum, Pflanzentransformation.Remarks,
Protokoll.Protokollname, Pflanzentransformation.[Number of rooted LInes]
FROM Operator INNER JOIN (Arbeitsgruppen INNER JOIN ([Resistenz Pflanze] RIGHT JOIN ([Resistenz
Bakterien] RIGHT JOIN (Construct INNER JOIN (Protokoll INNER JOIN (Agrobakterien INNER JOIN (Eltern
INNER JOIN Pflanzentransformation ON Eltern.ID = Pflanzentransformation.Pflanze) ON Agrobakterien.ID =
Pflanzentransformation.Agrobakterienstamm) ON Protokoll.[Protokoll ID] = Pflanzentransformation.Protokoll)
ON Construct.ConstructId = Pflanzentransformation.[GVO Nummer]) ON [Resistenz Bakterien].ID =
Construct.[M Resistance]) ON [Resistenz Pflanze].ID = Construct.[Plant Resistance]) ON Arbeitsgruppen.ID =
Pflanzentransformation.AG) ON Operator.ID = Pflanzentransformation.Worker;
```

Columns

Name	Type	Size
ID	Long Integer	4

AggregateType:	-1
AllowZeroLength:	False
AppendOnly:	False
Attributes:	Fixed Size; Auto-Increment; Updatable
CollatingOrder:	General
ColumnHidden:	False
ColumnOrder:	Default
ColumnWidth:	Default
DataUpdatable:	True
GUID:	{guid {491011A1-F233-4D8B-AF6D-E85AC96DC00A}}
OrdinalPosition:	0
Required:	False
SourceField:	ID
SourceTable:	Pflanzentransformation
TextAlign:	General

Name	Type	Size
GVO Nummer	Long Integer	4

AggregateType:	-1
AllowZeroLength:	False
AppendOnly:	False
Attributes:	Fixed Size; Updatable
CollatingOrder:	General

ColumnHidden:	False
ColumnOrder:	Default
ColumnWidth:	1650
DataUpdatable:	True
DecimalPlaces:	Auto
DisplayControl:	Text Box
GUID:	{guid {78A41B5D-B257-469B-8FCD-4CB99C084269}}
OrdinalPosition:	1
Required:	True
SourceField:	GVO Nummer
SourceTable:	Pflanzentransformation
TextAlign:	General

Konstruktname Text 255

AggregateType:	-1
AllowZeroLength:	True
AppendOnly:	False
Attributes:	Variable Length; Updatable
CollatingOrder:	General
ColumnHidden:	False
ColumnOrder:	Default
ColumnWidth:	2700
DataUpdatable:	True
DisplayControl:	Text Box
IMEMode:	0
IMESentenceMode:	3
OrdinalPosition:	2
Required:	False
SourceField:	ConstructName
SourceTable:	Construct
TextAlign:	General
UnicodeCompression:	False

Operator Text 50

AggregateType:	-1
AllowZeroLength:	True
AppendOnly:	False
Attributes:	Variable Length; Updatable
CollatingOrder:	General
ColumnHidden:	False
ColumnOrder:	Default
ColumnWidth:	1935
DataUpdatable:	True
Description:	Familienname verantwortlicher Wissenschafter
DisplayControl:	Text Box
GUID:	{guid {2AA8B70A-AC14-469A-8FAC-3C278DCB2AC7}}
IMEMode:	0
IMESentenceMode:	3
OrdinalPosition:	3
Required:	False
SourceField:	Operator
SourceTable:	Operator
TextAlign:	General
UnicodeCompression:	True

Arbeitsgruppen Text 50

AggregateType:	-1
AllowZeroLength:	True
AppendOnly:	False
Attributes:	Variable Length; Updatable
CollatingOrder:	General
ColumnHidden:	False
ColumnOrder:	Default
ColumnWidth:	2805
DataUpdatable:	True
DisplayControl:	Text Box
GUID:	{guid {1EC1EE36-3D42-4A84-8641-C819F641B1F1}}
IMEMode:	0
IMESentenceMode:	3
OrdinalPosition:	4
Required:	False
SourceField:	Arbeitsgruppen
SourceTable:	Arbeitsgruppen
UnicodeCompression:	True

Agrobakterinstamm Text 50

AggregateType:	-1
AllowZeroLength:	True
AppendOnly:	False
Attributes:	Variable Length; Updatable
CollatingOrder:	General
ColumnHidden:	False
ColumnOrder:	Default
ColumnWidth:	Default
DataUpdatable:	True
DisplayControl:	Text Box
IMEMode:	0
IMESentenceMode:	3
OrdinalPosition:	5
Required:	False
SourceField:	Agrobakterinstamm
SourceTable:	Agrobakterien
TextAlign:	General
UnicodeCompression:	True

Resistenz Bakterien.Resistenz Text 50

AggregateType:	-1
AllowZeroLength:	True
AppendOnly:	False
Attributes:	Variable Length; Updatable
CollatingOrder:	General
ColumnHidden:	False
ColumnOrder:	Default
ColumnWidth:	2160
DataUpdatable:	True
DisplayControl:	Text Box
GUID:	{guid {E2D30250-2F7D-4321-8B7F-BF08B22AB9CF}}
IMEMode:	0
IMESentenceMode:	3
OrdinalPosition:	6
Required:	False
SourceField:	Resistenz

SourceTable: Resistenz Bakterien
TextAlign: General
UnicodeCompression: True

Resistenz Pflanze.Resistenz Text 50

AggregateType: -1
AllowZeroLength: True
AppendOnly: False
Attributes: Variable Length; Updatable
CollatingOrder: General
ColumnHidden: False
ColumnOrder: Default
ColumnWidth: Default
DataUpdatable: True
DisplayControl: Text Box
GUID: {guid {8D71BD08-D542-4B6A-8796-02E82F37D7E2}}
IMEMode: 0
IMESentenceMode: 3
OrdinalPosition: 7
Required: False
SourceField: Resistenz
SourceTable: Resistenz Pflanze
TextAlign: General
UnicodeCompression: True

Spezies Text 50

AggregateType: -1
AllowZeroLength: True
AppendOnly: False
Attributes: Variable Length; Updatable
CollatingOrder: General
ColumnHidden: False
ColumnOrder: Default
ColumnWidth: 3855
DataUpdatable: True
DisplayControl: Text Box
GUID: {guid {FA3945B0-6E6C-423D-8BEE-0935B5FD7526}}
IMEMode: 0
IMESentenceMode: 3
OrdinalPosition: 8
Required: False
SourceField: Spezies
SourceTable: Eltern
TextAlign: General
UnicodeCompression: True

Varietät Text 50

AggregateType: -1
AllowZeroLength: True
AppendOnly: False
Attributes: Variable Length; Updatable
CollatingOrder: General
ColumnHidden: False
ColumnOrder: Default
ColumnWidth: 1695
DataUpdatable: True

DisplayControl:	Text Box
GUID:	{guid {1AF19923-4CB9-4A37-BDF6-9A285C5D1D00}}
IMEMode:	0
IMESentenceMode:	3
OrdinalPosition:	9
Required:	False
SourceField:	Varietät
SourceTable:	Eltern
TextAlign:	General
UnicodeCompression:	True

WT Yes/No 1

AggregateType:	-1
AllowZeroLength:	False
AppendOnly:	False
Attributes:	Fixed Size; Updatable
CollatingOrder:	General
ColumnHidden:	False
ColumnOrder:	Default
ColumnWidth:	Default
DataUpdatable:	True
DisplayControl:	106
Format:	Yes/No
GUID:	{guid {C2BE4715-B4A6-4077-A3F6-D1026FADBAC3}}
OrdinalPosition:	10
Required:	False
SourceField:	WT
SourceTable:	Eltern
TextAlign:	General

GMO Nummer Long Integer 4

AggregateType:	-1
AllowZeroLength:	False
AppendOnly:	False
Attributes:	Fixed Size; Updatable
CollatingOrder:	General
ColumnHidden:	False
ColumnOrder:	Default
ColumnWidth:	Default
DataUpdatable:	True
DecimalPlaces:	Auto
DisplayControl:	Text Box
GUID:	{guid {0374CE7E-E119-4264-8426-B9502000A391}}
OrdinalPosition:	11
Required:	False
SourceField:	GMO Nummer
SourceTable:	Eltern
TextAlign:	General

Linie Long Integer 4

AggregateType:	-1
AllowZeroLength:	False
AppendOnly:	False
Attributes:	Fixed Size; Updatable
CollatingOrder:	General
ColumnHidden:	False

ColumnOrder:	Default
ColumnWidth:	Default
DataUpdatable:	True
DecimalPlaces:	Auto
DisplayControl:	Text Box
GUID:	{guid {D523E511-9A67-4956-9BF5-D1965960274A}}
OrdinalPosition:	12
Required:	False
SourceField:	Linie
SourceTable:	Eltern
TextAlign:	General

Transformationsdatum Date/Time 8

AggregateType:	-1
AllowZeroLength:	False
AppendOnly:	False
Attributes:	Fixed Size; Updatable
CollatingOrder:	General
ColumnHidden:	False
ColumnOrder:	Default
ColumnWidth:	2850
DataUpdatable:	True
GUID:	{guid {001E5420-4F1C-4D41-9502-4F9521AA6AF1}}
IMEMode:	0
IMESentenceMode:	3
OrdinalPosition:	13
Required:	False
ShowDatePicker:	For dates
SourceField:	Transformationsdatum
SourceTable:	Pflanzentransformation
TextAlign:	General

Result Text 50

AggregateType:	-1
AllowMultipleValues:	False
AllowValueListEdits:	False
AllowZeroLength:	True
AppendOnly:	False
Attributes:	Variable Length; Updatable
BoundColumn:	1
CollatingOrder:	General
ColumnCount:	1
ColumnHeads:	False
ColumnHidden:	False
ColumnOrder:	Default
ColumnWidth:	2280
ColumnWidths:	2385
DataUpdatable:	True
DisplayControl:	Combo Box
GUID:	{guid {678F1CC4-5A06-4A1D-A7D4-BBBE683B3FA1}}
IMEMode:	0
IMESentenceMode:	3
LimitToList:	False
ListRows:	8
ListWidth:	2385twip
OrdinalPosition:	14

Required:	False	
RowSource:	"übergeben";"kontaminiert";"zurückgezogen";"entsorgt: kein Kallus";"entsorgt: keine Regeneration";"andere"	
RowSourceType:	Value List	
ShowOnlyRowSourceValues:	False	
SourceField:	Result	
SourceTable:	Pflanzentransformation	
TextAlign:	General	
UnicodeCompression:	True	

**Enddatum**	Date/Time	8
AggregateType:	-1	
AllowZeroLength:	False	
AppendOnly:	False	
Attributes:	Fixed Size; Updatable	
CollatingOrder:	General	
ColumnHidden:	False	
ColumnOrder:	Default	
ColumnWidth:	Default	
DataUpdatable:	True	
GUID:	{guid {9256514F-89E7-4417-895A-7E12A2ABA75E}}	
IMEMode:	0	
IMESentenceMode:	3	
OrdinalPosition:	15	
Required:	False	
ShowDatePicker:	For dates	
SourceField:	Enddatum	
SourceTable:	Pflanzentransformation	
TextAlign:	General	

**Remarks**	Memo	N/A
AggregateType:	-1	
AllowZeroLength:	True	
AppendOnly:	False	
Attributes:	Variable Length; Updatable	
CollatingOrder:	General	
ColumnHidden:	False	
ColumnOrder:	Default	
ColumnWidth:	Default	
DataUpdatable:	True	
GUID:	{guid {9E3C6856-13CF-4E9C-8E22-8E72D8B91407}}	
IMEMode:	0	
IMESentenceMode:	3	
OrdinalPosition:	16	
Required:	False	
SourceField:	Remarks	
SourceTable:	Pflanzentransformation	
TextAlign:	General	
TextFormat:	Plain Text	
UnicodeCompression:	True	

**Protokollname**	Text	50
AggregateType:	-1	
AllowZeroLength:	True	
AppendOnly:	False	

Attributes:	Variable Length; Updatable
CollatingOrder:	General
ColumnHidden:	False
ColumnOrder:	Default
ColumnWidth:	4905
DataUpdatable:	True
Description:	Eindeutiger Name für das Protokoll
DisplayControl:	Text Box
GUID:	{guid {BA35B293-EC1A-433F-A9CA-F649675F83D2}}
IMEMode:	0
IMESentenceMode:	3
OrdinalPosition:	17
Required:	False
SourceField:	Protokollname
SourceTable:	Protokoll
TextAlign:	General
UnicodeCompression:	True

Number of rooted LInes   Long Integer   4

AggregateType:	-1
AllowZeroLength:	False
AppendOnly:	False
Attributes:	Fixed Size; Updatable
CollatingOrder:	General
ColumnHidden:	False
ColumnOrder:	Default
ColumnWidth:	Default
DataUpdatable:	True
DecimalPlaces:	Auto
Description:	Number of Lines that made roots (festgelegt 06.05.09)
DisplayControl:	Text Box
GUID:	{guid {740AD5D9-15DC-4816-BF77-8126337931D0}}
OrdinalPosition:	18
Required:	False
SourceField:	Number of rooted LInes
SourceTable:	Pflanzentransformation
TextAlign:	General

Table Indexes

Name	Number of Fields
ArbeitsgruppenPflanzentransformation	1

Clustered:	False
DistinctCount:	1
Foreign:	True
IgnoreNulls:	False
Name:	ArbeitsgruppenPflanzentransformation
Primary:	False
Required:	False
Unique:	False
Fields:	
AG	Ascending

Name	Number of Fields
Id Nummer	1

Clustered:	False

	DistinctCount:	4
	Foreign:	False
	IgnoreNulls:	False
	Name:	Id Nummer
	Primary:	False
	Required:	False
	Unique:	False
	Fields:	
	GVO Nummer	Ascending
Number of rooted LInes		1
	Clustered:	False
	DistinctCount:	3
	Foreign:	False
	IgnoreNulls:	False
	Name:	Number of rooted LInes
	Primary:	False
	Required:	False
	Unique:	False
	Fields:	
	Number of rooted LInes	Ascending
Number of Shots		1
	Clustered:	False
	DistinctCount:	2
	Foreign:	False
	IgnoreNulls:	False
	Name:	Number of Shots
	Primary:	False
	Required:	False
	Unique:	False
	Fields:	
	Number of Shots	Ascending
OperatorPflanzentransformation		1
	Clustered:	False
	DistinctCount:	2
	Foreign:	True
	IgnoreNulls:	False
	Name:	OperatorPflanzentransformation
	Primary:	False
	Required:	False
	Unique:	False
	Fields:	
	Worker	Ascending
PrimaryKey		1
	Clustered:	False
	DistinctCount:	7
	Foreign:	False
	IgnoreNulls:	False
	Name:	PrimaryKey
	Primary:	True
	Required:	True
	Unique:	True
	Fields:	
	ID	Ascending

Result_ID		1
	Clustered:	False
	DistinctCount:	2
	Foreign:	False
	IgnoreNulls:	False
	Name:	Result_ID
	Primary:	False
	Required:	False
	Unique:	False
	Fields:	
	Result_ID	Ascending
ConstructId		1
	Clustered:	False
	DistinctCount:	16
	Foreign:	False
	IgnoreNulls:	False
	Name:	ConstructId
	Primary:	True
	Required:	True
	Unique:	True
	Fields:	
	ConstructId	Ascending
ID		1
	Clustered:	False
	DistinctCount:	2
	Foreign:	False
	IgnoreNulls:	False
	Name:	ID
	Primary:	False
	Required:	False
	Unique:	False
	Fields:	
	ID	Ascending
ID_LIMS		1
	Clustered:	False
	DistinctCount:	2
	Foreign:	False
	IgnoreNulls:	False
	Name:	ID_LIMS
	Primary:	False
	Required:	False
	Unique:	False
	Fields:	
	ID_LIMS	Ascending
PrimaryKey		1
	Clustered:	False
	DistinctCount:	2
	Foreign:	False
	IgnoreNulls:	False
	Name:	PrimaryKey
	Primary:	True
	Required:	True
	Unique:	True

	Fields:	
	ID	Ascending
Abkürzung		1
	Clustered:	False
	DistinctCount:	2
	Foreign:	False
	IgnoreNulls:	False
	Name:	Abkürzung
	Primary:	False
	Required:	False
	Unique:	True
	Fields:	
	Abkürzung	Ascending
ID		1
	Clustered:	False
	DistinctCount:	21
	Foreign:	False
	IgnoreNulls:	False
	Name:	ID
	Primary:	False
	Required:	False
	Unique:	False
	Fields:	
	ID	Ascending
PrimaryKey		1
	Clustered:	False
	DistinctCount:	21
	Foreign:	False
	IgnoreNulls:	False
	Name:	PrimaryKey
	Primary:	True
	Required:	True
	Unique:	True
	Fields:	
	ID	Ascending
ID		1
	Clustered:	False
	DistinctCount:	4
	Foreign:	False
	IgnoreNulls:	False
	Name:	ID
	Primary:	False
	Required:	False
	Unique:	False
	Fields:	
	ID	Ascending
PrimaryKey		1
	Clustered:	False
	DistinctCount:	4
	Foreign:	False
	IgnoreNulls:	False
	Name:	PrimaryKey
	Primary:	True

	Required:	True
	Unique:	True
	Fields:	
	ID	Ascending
ID		1
	Clustered:	False
	DistinctCount:	15
	Foreign:	False
	IgnoreNulls:	False
	Name:	ID
	Primary:	False
	Required:	False
	Unique:	False
	Fields:	
	ID	Ascending
PrimaryKey		1
	Clustered:	False
	DistinctCount:	15
	Foreign:	False
	IgnoreNulls:	False
	Name:	PrimaryKey
	Primary:	True
	Required:	True
	Unique:	True
	Fields:	
	ID	Ascending
ID		1
	Clustered:	False
	DistinctCount:	12
	Foreign:	False
	IgnoreNulls:	False
	Name:	ID
	Primary:	False
	Required:	False
	Unique:	False
	Fields:	
	ID	Ascending
PrimaryKey		1
	Clustered:	False
	DistinctCount:	12
	Foreign:	False
	IgnoreNulls:	False
	Name:	PrimaryKey
	Primary:	True
	Required:	True
	Unique:	True
	Fields:	
	ID	Ascending
GMO Nummer		1
	Clustered:	False
	DistinctCount:	15
	Foreign:	False
	IgnoreNulls:	False

	Name:	GMO Nummer
	Primary:	False
	Required:	False
	Unique:	False
	Fields:	
	GMO Nummer	Ascending
ID		1
	Clustered:	False
	DistinctCount:	28
	Foreign:	False
	IgnoreNulls:	False
	Name:	ID
	Primary:	False
	Required:	False
	Unique:	False
	Fields:	
	ID	Ascending
ID_Pflanzenarten_Ref		1
	Clustered:	False
	DistinctCount:	4
	Foreign:	False
	IgnoreNulls:	False
	Name:	ID_Pflanzenarten_Ref
	Primary:	False
	Required:	False
	Unique:	False
	Fields:	
	ID_Pflanzenarten_Ref	Ascending
PrimaryKey		1
	Clustered:	False
	DistinctCount:	28
	Foreign:	False
	IgnoreNulls:	False
	Name:	PrimaryKey
	Primary:	True
	Required:	True
	Unique:	True
	Fields:	
	ID	Ascending
ArbeitsgruppenPflanzentransformation		1
	Clustered:	False
	DistinctCount:	1
	Foreign:	True
	IgnoreNulls:	False
	Name:	ArbeitsgruppenPflanzentransformation
	Primary:	False
	Required:	False
	Unique:	False
	Fields:	
	AG	Ascending
Id Nummer		1
	Clustered:	False
	DistinctCount:	4

	Foreign:	False
	IgnoreNulls:	False
	Name:	Id Nummer
	Primary:	False
	Required:	False
	Unique:	False
	Fields:	
	GVO Nummer	Ascending
Number of rooted LInes		1
	Clustered:	False
	DistinctCount:	3
	Foreign:	False
	IgnoreNulls:	False
	Name:	Number of rooted LInes
	Primary:	False
	Required:	False
	Unique:	False
	Fields:	
	Number of rooted LInes	Ascending
Number of Shots		1
	Clustered:	False
	DistinctCount:	2
	Foreign:	False
	IgnoreNulls:	False
	Name:	Number of Shots
	Primary:	False
	Required:	False
	Unique:	False
	Fields:	
	Number of Shots	Ascending
OperatorPflanzentransformation		1
	Clustered:	False
	DistinctCount:	2
	Foreign:	True
	IgnoreNulls:	False
	Name:	OperatorPflanzentransformation
	Primary:	False
	Required:	False
	Unique:	False
	Fields:	
	Worker	Ascending
PrimaryKey		1
	Clustered:	False
	DistinctCount:	7
	Foreign:	False
	IgnoreNulls:	False
	Name:	PrimaryKey
	Primary:	True
	Required:	True
	Unique:	True
	Fields:	
	ID	Ascending
Result_ID		1

	Clustered:	False
	DistinctCount:	2
	Foreign:	False
	IgnoreNulls:	False
	Name:	Result_ID
	Primary:	False
	Required:	False
	Unique:	False
	Fields:	
	Result_ID	Ascending
PrimaryKey		1
	Clustered:	False
	DistinctCount:	3
	Foreign:	False
	IgnoreNulls:	False
	Name:	PrimaryKey
	Primary:	True
	Required:	True
	Unique:	True
	Fields:	
	Protokoll ID	Ascending
ProtokollArt		1
	Clustered:	False
	DistinctCount:	1
	Foreign:	False
	IgnoreNulls:	False
	Name:	ProtokollArt
	Primary:	False
	Required:	False
	Unique:	False
	Fields:	
	Art	Ascending
ArbeitsgruppenPflanzentransformation		1
	Clustered:	False
	DistinctCount:	1
	Foreign:	True
	IgnoreNulls:	False
	Name:	ArbeitsgruppenPflanzentransformation
	Primary:	False
	Required:	False
	Unique:	False
	Fields:	
	AG	Ascending
Id Nummer		1
	Clustered:	False
	DistinctCount:	4
	Foreign:	False
	IgnoreNulls:	False
	Name:	Id Nummer
	Primary:	False
	Required:	False
	Unique:	False

	Fields:	
	GVO Nummer	Ascending
Number of rooted LInes		1
	Clustered:	False
	DistinctCount:	3
	Foreign:	False
	IgnoreNulls:	False
	Name:	Number of rooted LInes
	Primary:	False
	Required:	False
	Unique:	False
	Fields:	
	Number of rooted LInes	Ascending
Number of Shots		1
	Clustered:	False
	DistinctCount:	2
	Foreign:	False
	IgnoreNulls:	False
	Name:	Number of Shots
	Primary:	False
	Required:	False
	Unique:	False
	Fields:	
	Number of Shots	Ascending
OperatorPflanzentransformation		1
	Clustered:	False
	DistinctCount:	2
	Foreign:	True
	IgnoreNulls:	False
	Name:	OperatorPflanzentransformation
	Primary:	False
	Required:	False
	Unique:	False
	Fields:	
	Worker	Ascending
PrimaryKey		1
	Clustered:	False
	DistinctCount:	7
	Foreign:	False
	IgnoreNulls:	False
	Name:	PrimaryKey
	Primary:	True
	Required:	True
	Unique:	True
	Fields:	
	ID	Ascending
Result_ID		1
	Clustered:	False
	DistinctCount:	2
	Foreign:	False
	IgnoreNulls:	False
	Name:	Result_ID
	Primary:	False

Required:	False
Unique:	False
Fields:	
Result_ID	Ascending

User Permissions

admin	Delete; Read Permissions; Set Permissions; Change Owner, Read Definition; Write Definition; Read Data; Insert Data; Update Data; Delete Data

Group Permissions

Admins	Delete; Read Permissions; Set Permissions; Change Owner, Read Definition; Write Definition; Read Data; Insert Data; Update Data; Delete Data
Users	Delete; Read Permissions; Set Permissions; Change Owner, Read Definition; Write Definition; Read Data; Insert Data; Update Data; Delete Data

Properties

DateCreated:	21.09.2009 16:31:17	DefaultView:	2
DOL:	Long binary data	FilterOnLoad:	False
GUID:	{guid {FF5CDEBA-63CD-4656-B060-1F260882C7A9}}	LastUpdated:	25.09.2009 16:08:01
MaxRecords:	0	ODBCTimeout:	60
OrderByOn:	False	OrderByOnLoad:	True
Orientation:	Left-to-Right	RecordLocks:	No Locks
RecordsAffected:	0	RecordsetType:	Dynaset
ReturnsRecords:	True	TotalsRow:	False
Type:	0	Updatable:	True

SQL

```
SELECT Pflanzentransformation.ID, Pflanzentransformation.[GVO Nummer], Construct.ConstructName AS
Konstruktname, Operator.Operator, Arbeitsgruppen.Arbeitsgruppen, [Resistenz Bakterien].Resistenz,
[Resistenz Pflanze].Resistenz, Eltern.Spezies, Eltern.Varietät, Eltern.WT, Eltern.[GMO Nummer], Eltern.Linie,
Pflanzentransformation.Transformationsdatum, Pflanzentransformation.Result,
Pflanzentransformation.Enddatum, Pflanzentransformation.Remarks, Protokoll.Protokollname,
Pflanzentransformation.[Number of rooted LInes]
FROM Operator INNER JOIN (Arbeitsgruppen INNER JOIN ([Resistenz Pflanze] RIGHT JOIN ([Resistenz
Bakterien] RIGHT JOIN (Construct INNER JOIN (Protokoll INNER JOIN (Eltern INNER JOIN
Pflanzentransformation ON Eltern.ID = Pflanzentransformation.Pflanze) ON Protokoll.[Protokoll ID] =
Pflanzentransformation.Protokoll) ON Construct.ConstructId = Pflanzentransformation.[GVO Nummer]) ON
[Resistenz Bakterien].ID = Construct.[M Resistance]) ON [Resistenz Pflanze].ID = Construct.[Plant
Resistance]) ON Arbeitsgruppen.ID = Pflanzentransformation.AG) ON Operator.ID =
Pflanzentransformation.Worker;
```

Columns

Name	Type	Size
ID	Long Integer	4

AggregateType:	-1
AllowZeroLength:	False
AppendOnly:	False
Attributes:	Fixed Size; Auto-Increment; Updatable
CollatingOrder:	General
ColumnHidden:	False
ColumnOrder:	Default
ColumnWidth:	Default
DataUpdatable:	True
GUID:	{guid {491011A1-F233-4D8B-AF6D-E85AC96DC00A}}
OrdinalPosition:	0
Required:	False
SourceField:	ID
SourceTable:	Pflanzentransformation
TextAlign:	General

Name	Type	Size
GVO Nummer	Long Integer	4

AggregateType:	-1
AllowZeroLength:	False
AppendOnly:	False
Attributes:	Fixed Size; Updatable
CollatingOrder:	General

	ColumnHidden:	False
	ColumnOrder:	Default
	ColumnWidth:	1650
	DataUpdatable:	True
	DecimalPlaces:	Auto
	DisplayControl:	Text Box
	GUID:	{guid {78A41B5D-B257-469B-8FCD-4CB99C084269}}
	OrdinalPosition:	1
	Required:	True
	SourceField:	GVO Nummer
	SourceTable:	Pflanzentransformation
	TextAlign:	General
Konstruktname		Text	255
	AggregateType:	-1
	AllowZeroLength:	True
	AppendOnly:	False
	Attributes:	Variable Length; Updatable
	CollatingOrder:	General
	ColumnHidden:	False
	ColumnOrder:	Default
	ColumnWidth:	2700
	DataUpdatable:	True
	DisplayControl:	Text Box
	IMEMode:	0
	IMESentenceMode:	3
	OrdinalPosition:	2
	Required:	False
	SourceField:	ConstructName
	SourceTable:	Construct
	TextAlign:	General
	UnicodeCompression:	False
Operator		Text	50
	AggregateType:	-1
	AllowZeroLength:	True
	AppendOnly:	False
	Attributes:	Variable Length; Updatable
	CollatingOrder:	General
	ColumnHidden:	False
	ColumnOrder:	Default
	ColumnWidth:	1935
	DataUpdatable:	True
	Description:	Familienname verantwortlicher Wissenschafter
	DisplayControl:	Text Box
	GUID:	{guid {2AA8B70A-AC14-469A-8FAC-3C278DCB2AC7}}
	IMEMode:	0
	IMESentenceMode:	3
	OrdinalPosition:	3
	Required:	False
	SourceField:	Operator
	SourceTable:	Operator
	TextAlign:	General
	UnicodeCompression:	True
Arbeitsgruppen		Text	50

AggregateType:	-1
AllowZeroLength:	True
AppendOnly:	False
Attributes:	Variable Length; Updatable
CollatingOrder:	General
ColumnHidden:	False
ColumnOrder:	Default
ColumnWidth:	2805
DataUpdatable:	True
DisplayControl:	Text Box
GUID:	{guid {1EC1EE36-3D42-4A84-8641-C819F641B1F1}}
IMEMode:	0
IMESentenceMode:	3
OrdinalPosition:	4
Required:	False
SourceField:	Arbeitsgruppen
SourceTable:	Arbeitsgruppen
UnicodeCompression:	True

Resistenz Bakterien.Resistenz Text 50

AggregateType:	-1
AllowZeroLength:	True
AppendOnly:	False
Attributes:	Variable Length; Updatable
CollatingOrder:	General
ColumnHidden:	False
ColumnOrder:	Default
ColumnWidth:	2160
DataUpdatable:	True
DisplayControl:	Text Box
GUID:	{guid {E2D30250-2F7D-4321-8B7F-BF08B22AB9CF}}
IMEMode:	0
IMESentenceMode:	3
OrdinalPosition:	5
Required:	False
SourceField:	Resistenz
SourceTable:	Resistenz Bakterien
TextAlign:	General
UnicodeCompression:	True

Resistenz Pflanze.Resistenz Text 50

AggregateType:	-1
AllowZeroLength:	True
AppendOnly:	False
Attributes:	Variable Length; Updatable
CollatingOrder:	General
ColumnHidden:	False
ColumnOrder:	Default
ColumnWidth:	Default
DataUpdatable:	True
DisplayControl:	Text Box
GUID:	{guid {8D71BD08-D542-4B6A-8796-02E82F37D7E2}}
IMEMode:	0
IMESentenceMode:	3
OrdinalPosition:	6
Required:	False

SourceField:	Resistenz	
SourceTable:	Resistenz Pflanze	
TextAlign:	General	
UnicodeCompression:	True	

Spezies	Text	50
AggregateType:	-1	
AllowZeroLength:	True	
AppendOnly:	False	
Attributes:	Variable Length; Updatable	
CollatingOrder:	General	
ColumnHidden:	False	
ColumnOrder:	Default	
ColumnWidth:	3855	
DataUpdatable:	True	
DisplayControl:	Text Box	
GUID:	{guid {FA3945B0-6E6C-423D-8BEE-0935B5FD7526}}	
IMEMode:	0	
IMESentenceMode:	3	
OrdinalPosition:	7	
Required:	False	
SourceField:	Spezies	
SourceTable:	Eltern	
TextAlign:	General	
UnicodeCompression:	True	

Varietät	Text	50
AggregateType:	-1	
AllowZeroLength:	True	
AppendOnly:	False	
Attributes:	Variable Length; Updatable	
CollatingOrder:	General	
ColumnHidden:	False	
ColumnOrder:	Default	
ColumnWidth:	1695	
DataUpdatable:	True	
DisplayControl:	Text Box	
GUID:	{guid {1AF19923-4CB9-4A37-BDF6-9A285C5D1D00}}	
IMEMode:	0	
IMESentenceMode:	3	
OrdinalPosition:	8	
Required:	False	
SourceField:	Varietät	
SourceTable:	Eltern	
TextAlign:	General	
UnicodeCompression:	True	

WT	Yes/No	1
AggregateType:	-1	
AllowZeroLength:	False	
AppendOnly:	False	
Attributes:	Fixed Size; Updatable	
CollatingOrder:	General	
ColumnHidden:	False	
ColumnOrder:	Default	
ColumnWidth:	Default	

DataUpdatable:	True	
DisplayControl:	106	
Format:	Yes/No	
GUID:	{guid {C2BE4715-B4A6-4077-A3F6-D1026FADBAC3}}	
OrdinalPosition:	9	
Required:	False	
SourceField:	WT	
SourceTable:	Eltern	
TextAlign:	General	
**GMO Nummer**	Long Integer	4
AggregateType:	-1	
AllowZeroLength:	False	
AppendOnly:	False	
Attributes:	Fixed Size; Updatable	
CollatingOrder:	General	
ColumnHidden:	False	
ColumnOrder:	Default	
ColumnWidth:	Default	
DataUpdatable:	True	
DecimalPlaces:	Auto	
DisplayControl:	Text Box	
GUID:	{guid {0374CE7E-E119-4264-8426-B9502000A391}}	
OrdinalPosition:	10	
Required:	False	
SourceField:	GMO Nummer	
SourceTable:	Eltern	
TextAlign:	General	
**Linie**	Long Integer	4
AggregateType:	-1	
AllowZeroLength:	False	
AppendOnly:	False	
Attributes:	Fixed Size; Updatable	
CollatingOrder:	General	
ColumnHidden:	False	
ColumnOrder:	Default	
ColumnWidth:	Default	
DataUpdatable:	True	
DecimalPlaces:	Auto	
DisplayControl:	Text Box	
GUID:	{guid {D523E511-9A67-4956-9BF5-D1965960274A}}	
OrdinalPosition:	11	
Required:	False	
SourceField:	Linie	
SourceTable:	Eltern	
TextAlign:	General	
**Transformationsdatum**	Date/Time	8
AggregateType:	-1	
AllowZeroLength:	False	
AppendOnly:	False	
Attributes:	Fixed Size; Updatable	
CollatingOrder:	General	
ColumnHidden:	False	
ColumnOrder:	Default	

ColumnWidth:	2850
DataUpdatable:	True
GUID:	{guid {001E5420-4F1C-4D41-9502-4F9521AA6AF1}}
IMEMode:	0
IMESentenceMode:	3
OrdinalPosition:	12
Required:	False
ShowDatePicker:	For dates
SourceField:	Transformationsdatum
SourceTable:	Pflanzentransformation
TextAlign:	General

Result Text 50

AggregateType:	-1
AllowMultipleValues:	False
AllowValueListEdits:	False
AllowZeroLength:	True
AppendOnly:	False
Attributes:	Variable Length; Updatable
BoundColumn:	1
CollatingOrder:	General
ColumnCount:	1
ColumnHeads:	False
ColumnHidden:	False
ColumnOrder:	Default
ColumnWidth:	2280
ColumnWidths:	2385
DataUpdatable:	True
DisplayControl:	Combo Box
GUID:	{guid {678F1CC4-5A06-4A1D-A7D4-BBBE683B3FA1}}
IMEMode:	0
IMESentenceMode:	3
LimitToList:	False
ListRows:	8
ListWidth:	2385twip
OrdinalPosition:	13
Required:	False
RowSource:	"übergeben";"kontaminiert";"zurückgezogen";"entsorgt: kein Kallus";"entsorgt: keine Regeneration";"andere"
RowSourceType:	Value List
ShowOnlyRowSourceValues:	False
SourceField:	Result
SourceTable:	Pflanzentransformation
TextAlign:	General
UnicodeCompression:	True

Enddatum Date/Time 8

AggregateType:	-1
AllowZeroLength:	False
AppendOnly:	False
Attributes:	Fixed Size; Updatable
CollatingOrder:	General
ColumnHidden:	False
ColumnOrder:	Default
ColumnWidth:	Default

DataUpdatable:	True	
GUID:	{guid {9256514F-89E7-4417-895A-7E12A2ABA75E}}	
IMEMode:	0	
IMESentenceMode:	3	
OrdinalPosition:	14	
Required:	False	
ShowDatePicker:	For dates	
SourceField:	Enddatum	
SourceTable:	Pflanzentransformation	
TextAlign:	General	

Remarks	Memo	N/A
AggregateType:	-1	
AllowZeroLength:	True	
AppendOnly:	False	
Attributes:	Variable Length; Updatable	
CollatingOrder:	General	
ColumnHidden:	False	
ColumnOrder:	Default	
ColumnWidth:	Default	
DataUpdatable:	True	
GUID:	{guid {9E3C6856-13CF-4E9C-8E22-8E72D8B91407}}	
IMEMode:	0	
IMESentenceMode:	3	
OrdinalPosition:	15	
Required:	False	
SourceField:	Remarks	
SourceTable:	Pflanzentransformation	
TextAlign:	General	
TextFormat:	Plain Text	
UnicodeCompression:	True	

Protokollname	Text	50
AggregateType:	-1	
AllowZeroLength:	True	
AppendOnly:	False	
Attributes:	Variable Length; Updatable	
CollatingOrder:	General	
ColumnHidden:	False	
ColumnOrder:	Default	
ColumnWidth:	4905	
DataUpdatable:	True	
Description:	Eindeutiger Name für das Protokoll	
DisplayControl:	Text Box	
GUID:	{guid {BA35B293-EC1A-433F-A9CA-F649675F83D2}}	
IMEMode:	0	
IMESentenceMode:	3	
OrdinalPosition:	16	
Required:	False	
SourceField:	Protokollname	
SourceTable:	Protokoll	
TextAlign:	General	
UnicodeCompression:	True	

Number of rooted LInes	Long Integer	4
AggregateType:	-1	

AllowZeroLength:	False
AppendOnly:	False
Attributes:	Fixed Size; Updatable
CollatingOrder:	General
ColumnHidden:	False
ColumnOrder:	Default
ColumnWidth:	Default
DataUpdatable:	True
DecimalPlaces:	Auto
Description:	Number of Lines that made roots (festgelegt 06.05.09)
DisplayControl:	Text Box
GUID:	{guid {740AD5D9-15DC-4816-BF77-8126337931D0}}
OrdinalPosition:	17
Required:	False
SourceField:	Number of rooted LInes
SourceTable:	Pflanzentransformation
TextAlign:	General

Table Indexes

Name		Number of Fields
ArbeitsgruppenPflanzentransformation		1
	Clustered:	False
	DistinctCount:	1
	Foreign:	True
	IgnoreNulls:	False
	Name:	ArbeitsgruppenPflanzentransformation
	Primary:	False
	Required:	False
	Unique:	False
	Fields:	
	AG	Ascending
Id Nummer		1
	Clustered:	False
	DistinctCount:	4
	Foreign:	False
	IgnoreNulls:	False
	Name:	Id Nummer
	Primary:	False
	Required:	False
	Unique:	False
	Fields:	
	GVO Nummer	Ascending
Number of rooted LInes		1
	Clustered:	False
	DistinctCount:	3
	Foreign:	False
	IgnoreNulls:	False
	Name:	Number of rooted LInes
	Primary:	False
	Required:	False
	Unique:	False

	Fields:	
	Number of rooted LInes	Ascending
Number of Shots		1
	Clustered:	False
	DistinctCount:	2
	Foreign:	False
	IgnoreNulls:	False
	Name:	Number of Shots
	Primary:	False
	Required:	False
	Unique:	False
	Fields:	
	Number of Shots	Ascending
OperatorPflanzentransformation		1
	Clustered:	False
	DistinctCount:	2
	Foreign:	True
	IgnoreNulls:	False
	Name:	OperatorPflanzentransformation
	Primary:	False
	Required:	False
	Unique:	False
	Fields:	
	Worker	Ascending
PrimaryKey		1
	Clustered:	False
	DistinctCount:	7
	Foreign:	False
	IgnoreNulls:	False
	Name:	PrimaryKey
	Primary:	True
	Required:	True
	Unique:	True
	Fields:	
	ID	Ascending
Result_ID		1
	Clustered:	False
	DistinctCount:	2
	Foreign:	False
	IgnoreNulls:	False
	Name:	Result_ID
	Primary:	False
	Required:	False
	Unique:	False
	Fields:	
	Result_ID	Ascending
ConstructId		1
	Clustered:	False
	DistinctCount:	16
	Foreign:	False
	IgnoreNulls:	False
	Name:	ConstructId
	Primary:	True

	Required:	True
	Unique:	True
	Fields:	
	ConstructId	Ascending
ID		1
	Clustered:	False
	DistinctCount:	2
	Foreign:	False
	IgnoreNulls:	False
	Name:	ID
	Primary:	False
	Required:	False
	Unique:	False
	Fields:	
	ID	Ascending
ID_LIMS		1
	Clustered:	False
	DistinctCount:	2
	Foreign:	False
	IgnoreNulls:	False
	Name:	ID_LIMS
	Primary:	False
	Required:	False
	Unique:	False
	Fields:	
	ID_LIMS	Ascending
PrimaryKey		1
	Clustered:	False
	DistinctCount:	2
	Foreign:	False
	IgnoreNulls:	False
	Name:	PrimaryKey
	Primary:	True
	Required:	True
	Unique:	True
	Fields:	
	ID	Ascending
Abkürzung		1
	Clustered:	False
	DistinctCount:	2
	Foreign:	False
	IgnoreNulls:	False
	Name:	Abkürzung
	Primary:	False
	Required:	False
	Unique:	True
	Fields:	
	Abkürzung	Ascending
ID		1
	Clustered:	False
	DistinctCount:	21
	Foreign:	False
	IgnoreNulls:	False

	Name:	ID
	Primary:	False
	Required:	False
	Unique:	False
	Fields:	
	ID	Ascending
PrimaryKey		1
	Clustered:	False
	DistinctCount:	21
	Foreign:	False
	IgnoreNulls:	False
	Name:	PrimaryKey
	Primary:	True
	Required:	True
	Unique:	True
	Fields:	
	ID	Ascending
ID		1
	Clustered:	False
	DistinctCount:	15
	Foreign:	False
	IgnoreNulls:	False
	Name:	ID
	Primary:	False
	Required:	False
	Unique:	False
	Fields:	
	ID	Ascending
PrimaryKey		1
	Clustered:	False
	DistinctCount:	15
	Foreign:	False
	IgnoreNulls:	False
	Name:	PrimaryKey
	Primary:	True
	Required:	True
	Unique:	True
	Fields:	
	ID	Ascending
ID		1
	Clustered:	False
	DistinctCount:	12
	Foreign:	False
	IgnoreNulls:	False
	Name:	ID
	Primary:	False
	Required:	False
	Unique:	False
	Fields:	
	ID	Ascending
PrimaryKey		1
	Clustered:	False
	DistinctCount:	12

	Foreign:	False
	IgnoreNulls:	False
	Name:	PrimaryKey
	Primary:	True
	Required:	True
	Unique:	True
	Fields:	
	ID	Ascending
GMO Nummer		1
	Clustered:	False
	DistinctCount:	15
	Foreign:	False
	IgnoreNulls:	False
	Name:	GMO Nummer
	Primary:	False
	Required:	False
	Unique:	False
	Fields:	
	GMO Nummer	Ascending
ID		1
	Clustered:	False
	DistinctCount:	28
	Foreign:	False
	IgnoreNulls:	False
	Name:	ID
	Primary:	False
	Required:	False
	Unique:	False
	Fields:	
	ID	Ascending
ID_Pflanzenarten_Ref		1
	Clustered:	False
	DistinctCount:	4
	Foreign:	False
	IgnoreNulls:	False
	Name:	ID_Pflanzenarten_Ref
	Primary:	False
	Required:	False
	Unique:	False
	Fields:	
	ID_Pflanzenarten_Ref	Ascending
PrimaryKey		1
	Clustered:	False
	DistinctCount:	28
	Foreign:	False
	IgnoreNulls:	False
	Name:	PrimaryKey
	Primary:	True
	Required:	True
	Unique:	True
	Fields:	
	ID	Ascending
ArbeitsgruppenPflanzentransformation		1

Clustered:	False
DistinctCount:	1
Foreign:	True
IgnoreNulls:	False
Name:	ArbeitsgruppenPflanzentransformation
Primary:	False
Required:	False
Unique:	False
Fields:	
AG	Ascending
**Id Nummer**	1
Clustered:	False
DistinctCount:	4
Foreign:	False
IgnoreNulls:	False
Name:	Id Nummer
Primary:	False
Required:	False
Unique:	False
Fields:	
GVO Nummer	Ascending
**Number of rooted LInes**	1
Clustered:	False
DistinctCount:	3
Foreign:	False
IgnoreNulls:	False
Name:	Number of rooted LInes
Primary:	False
Required:	False
Unique:	False
Fields:	
Number of rooted LInes	Ascending
**Number of Shots**	1
Clustered:	False
DistinctCount:	2
Foreign:	False
IgnoreNulls:	False
Name:	Number of Shots
Primary:	False
Required:	False
Unique:	False
Fields:	
Number of Shots	Ascending
**OperatorPflanzentransformation**	1
Clustered:	False
DistinctCount:	2
Foreign:	True
IgnoreNulls:	False
Name:	OperatorPflanzentransformation
Primary:	False
Required:	False
Unique:	False

Fields:
Worker Ascending

PrimaryKey 1
Clustered: False
DistinctCount: 7
Foreign: False
IgnoreNulls: False
Name: PrimaryKey
Primary: True
Required: True
Unique: True
Fields:
ID Ascending

Result_ID 1
Clustered: False
DistinctCount: 2
Foreign: False
IgnoreNulls: False
Name: Result_ID
Primary: False
Required: False
Unique: False
Fields:
Result_ID Ascending

PrimaryKey 1
Clustered: False
DistinctCount: 3
Foreign: False
IgnoreNulls: False
Name: PrimaryKey
Primary: True
Required: True
Unique: True
Fields:
Protokoll ID Ascending

ProtokollArt 1
Clustered: False
DistinctCount: 1
Foreign: False
IgnoreNulls: False
Name: ProtokollArt
Primary: False
Required: False
Unique: False
Fields:
Art Ascending

ArbeitsgruppenPflanzentransformation 1
Clustered: False
DistinctCount: 1
Foreign: True
IgnoreNulls: False
Name: ArbeitsgruppenPflanzentransformation
Primary: False

	Required:	False
	Unique:	False
	Fields:	
	AG	Ascending
Id Nummer		1
	Clustered:	False
	DistinctCount:	4
	Foreign:	False
	IgnoreNulls:	False
	Name:	Id Nummer
	Primary:	False
	Required:	False
	Unique:	False
	Fields:	
	GVO Nummer	Ascending
Number of rooted LInes		1
	Clustered:	False
	DistinctCount:	3
	Foreign:	False
	IgnoreNulls:	False
	Name:	Number of rooted LInes
	Primary:	False
	Required:	False
	Unique:	False
	Fields:	
	Number of rooted LInes	Ascending
Number of Shots		1
	Clustered:	False
	DistinctCount:	2
	Foreign:	False
	IgnoreNulls:	False
	Name:	Number of Shots
	Primary:	False
	Required:	False
	Unique:	False
	Fields:	
	Number of Shots	Ascending
OperatorPflanzentransformation		1
	Clustered:	False
	DistinctCount:	2
	Foreign:	True
	IgnoreNulls:	False
	Name:	OperatorPflanzentransformation
	Primary:	False
	Required:	False
	Unique:	False
	Fields:	
	Worker	Ascending
PrimaryKey		1
	Clustered:	False
	DistinctCount:	7
	Foreign:	False
	IgnoreNulls:	False

	Name:	PrimaryKey
	Primary:	True
	Required:	True
	Unique:	True
	Fields:	
	ID	Ascending
Result_ID		1
	Clustered:	False
	DistinctCount:	2
	Foreign:	False
	IgnoreNulls:	False
	Name:	Result_ID
	Primary:	False
	Required:	False
	Unique:	False
	Fields:	
	Result_ID	Ascending

User Permissions

admin	Delete; Read Permissions; Set Permissions; Change Owner, Read Definition; Write Definition; Read Data; Insert Data; Update Data; Delete Data

Group Permissions

Admins	Delete; Read Permissions; Set Permissions; Change Owner, Read Definition; Write Definition; Read Data; Insert Data; Update Data; Delete Data
Users	Delete; Read Permissions; Set Permissions; Change Owner, Read Definition; Write Definition; Read Data; Insert Data; Update Data; Delete Data

Properties

DateCreated:	07.07.2005 19:38:58	DefaultView:	2
DOL:	Long binary data	GUID:	{guid {E7B14E14-DCB8-4BFC-92F5-7DD0CBEF2E9F}}
LastUpdated:	13.09.2006 10:05:49	MaxRecords:	0
ODBCTimeout:	60	OrderByOn:	True
Orientation:	Left-to-Right	RecordLocks:	No Locks
RecordsAffected:	0	RecordsetType:	Dynaset
ReturnsRecords:	True	Type:	0
Updatable:	True		

SQL

```
SELECT Pflanzentransformation.[GVO Nummer], Operator.Operator, Protokoll.Protokollname,
Pflanzentransformation.Transformationsdatum, [Transformationsdatum]+[ZeitvonStart] AS Datum,
Transformationsschritte.Prozess, Pflanzentransformation.Protokoll, Pflanzentransformation.[Resistenzmarker
Pflanze]
FROM (Protokoll INNER JOIN (Operator INNER JOIN Pflanzentransformation ON
Operator.ID=Pflanzentransformation.Worker) ON Protokoll.[Protokoll ID]=Pflanzentransformation.Protokoll)
INNER JOIN Transformationsschritte ON Protokoll.[Protokoll ID]=Transformationsschritte.Art
WHERE ((([Transformationsdatum]+[ZeitvonStart])>=[von] And
([Transformationsdatum]+[ZeitvonStart])<=[bis]) AND ((Pflanzentransformation.Enddatum) Is Null Or
(Pflanzentransformation.Enddatum)>=Date()))
ORDER BY [Transformationsdatum]+[ZeitvonStart];
```

Query Parameters

Name	Type
[von]	Text
[bis]	Text

Columns

Name	Type	Size
GVO Nummer	Long Integer	4

AggregateType:	-1
AllowZeroLength:	False
AppendOnly:	False
Attributes:	Fixed Size
CollatingOrder:	General
ColumnHidden:	False
ColumnOrder:	Default
ColumnWidth:	1650
DataUpdatable:	False
DecimalPlaces:	Auto
DefaultValue:	0
DisplayControl:	Text Box
GUID:	{guid {78A41B5D-B257-469B-8FCD-4CB99C084269}}
OrdinalPosition:	0
Required:	True
SourceField:	GVO Nummer
SourceTable:	Pflanzentransformation
TextAlign:	General

Operator	Text	50
AggregateType:	-1	
AllowZeroLength:	True	
AppendOnly:	False	
Attributes:	Variable Length	
CollatingOrder:	General	
ColumnHidden:	False	
ColumnOrder:	Default	
ColumnWidth:	1935	
DataUpdatable:	False	
Description:	Familienname verantwortlicher Wissenschafter	
DisplayControl:	Text Box	
GUID:	{guid {2AA8B70A-AC14-469A-8FAC-3C278DCB2AC7}}	
IMEMode:	0	
IMESentenceMode:	3	
OrdinalPosition:	1	
Required:	False	
SourceField:	Operator	
SourceTable:	Operator	
TextAlign:	General	
UnicodeCompression:	True	
Protokollname	Text	50
AggregateType:	-1	
AllowZeroLength:	True	
AppendOnly:	False	
Attributes:	Variable Length	
CollatingOrder:	General	
ColumnHidden:	False	
ColumnOrder:	Default	
ColumnWidth:	3165	
DataUpdatable:	False	
Description:	Eindeutiger Name für das Protokoll	
DisplayControl:	Text Box	
GUID:	{guid {BA35B293-EC1A-433F-A9CA-F649675F83D2}}	
IMEMode:	0	
IMESentenceMode:	3	
OrdinalPosition:	2	
Required:	False	
SourceField:	Protokollname	
SourceTable:	Protokoll	
TextAlign:	General	
UnicodeCompression:	True	
Transformationsdatum	Date/Time	8
AggregateType:	-1	
AllowZeroLength:	False	
AppendOnly:	False	
Attributes:	Fixed Size	
CollatingOrder:	General	
ColumnHidden:	False	
ColumnOrder:	Default	
ColumnWidth:	1560	
DataUpdatable:	False	
GUID:	{guid {001E5420-4F1C-4D41-9502-4F9521AA6AF1}}	
IMEMode:	0	

	IMESentenceMode:	3	
	OrdinalPosition:	3	
	Required:	False	
	ShowDatePicker:	For dates	
	SourceField:	Transformationsdatum	
	SourceTable:	Pflanzentransformation	
	TextAlign:	General	
Datum		Date/Time	8
	AllowZeroLength:	False	
	AppendOnly:	False	
	Attributes:	Fixed Size	
	CollatingOrder:	General	
	DataUpdatable:	False	
	GUID:	{guid {35FB5C4D-E5BF-45BB-AA4C-13556CCB2554}}	
	OrdinalPosition:	4	
	Required:	False	
Prozess		Text	50
	AggregateType:	-1	
	AllowZeroLength:	True	
	AppendOnly:	False	
	Attributes:	Variable Length	
	CollatingOrder:	General	
	ColumnHidden:	False	
	ColumnOrder:	Default	
	ColumnWidth:	2910	
	DataUpdatable:	False	
	Description:	Arbeitsprozess	
	DisplayControl:	Text Box	
	GUID:	{guid {F145B5A2-C291-40CF-8D68-20B48F7A3039}}	
	IMEMode:	0	
	IMESentenceMode:	3	
	OrdinalPosition:	5	
	Required:	False	
	SourceField:	Prozess	
	SourceTable:	Transformationsschritte	
	TextAlign:	General	
	UnicodeCompression:	True	
Protokoll		Long Integer	4
	AggregateType:	-1	
	AllowZeroLength:	False	
	AppendOnly:	False	
	Attributes:	Fixed Size	
	CollatingOrder:	General	
	ColumnHidden:	False	
	ColumnOrder:	Default	
	ColumnWidth:	Default	
	DataUpdatable:	False	
	DecimalPlaces:	Auto	
	DefaultValue:	0	
	DisplayControl:	Text Box	
	GUID:	{guid {66638D24-E387-4387-948D-82A060A41F76}}	
	OrdinalPosition:	6	
	Required:	False	

SourceField:	Protokoll
SourceTable:	Pflanzentransformation
TextAlign:	General

Resistenzmarker Pflanze Long Integer 4

AggregateType:	-1
AllowZeroLength:	False
AppendOnly:	False
Attributes:	Fixed Size
CollatingOrder:	General
ColumnHidden:	False
ColumnOrder:	Default
ColumnWidth:	Default
DataUpdatable:	False
DecimalPlaces:	Auto
DisplayControl:	Text Box
GUID:	{guid {83CFEFFF-1F25-4FF3-89A6-BB5706AFD14C}}
OrdinalPosition:	7
Required:	False
SourceField:	Resistenzmarker Pflanze
SourceTable:	Pflanzentransformation
TextAlign:	General

Table Indexes

Name	Number of Fields
ArbeitsgruppenPflanzentransformation	1

Clustered:	False
DistinctCount:	1
Foreign:	True
IgnoreNulls:	False
Name:	ArbeitsgruppenPflanzentransformation
Primary:	False
Required:	False
Unique:	False
Fields:	
AG	Ascending

Id Nummer 1

Clustered:	False
DistinctCount:	4
Foreign:	False
IgnoreNulls:	False
Name:	Id Nummer
Primary:	False
Required:	False
Unique:	False
Fields:	
GVO Nummer	Ascending

Number of rooted LInes 1

Clustered:	False
DistinctCount:	3
Foreign:	False
IgnoreNulls:	False
Name:	Number of rooted LInes

	Primary:	False
	Required:	False
	Unique:	False
	Fields:	
	Number of rooted LInes	Ascending
Number of Shots		1
	Clustered:	False
	DistinctCount:	2
	Foreign:	False
	IgnoreNulls:	False
	Name:	Number of Shots
	Primary:	False
	Required:	False
	Unique:	False
	Fields:	
	Number of Shots	Ascending
OperatorPflanzentransformation		1
	Clustered:	False
	DistinctCount:	2
	Foreign:	True
	IgnoreNulls:	False
	Name:	OperatorPflanzentransformation
	Primary:	False
	Required:	False
	Unique:	False
	Fields:	
	Worker	Ascending
PrimaryKey		1
	Clustered:	False
	DistinctCount:	7
	Foreign:	False
	IgnoreNulls:	False
	Name:	PrimaryKey
	Primary:	True
	Required:	True
	Unique:	True
	Fields:	
	ID	Ascending
Result_ID		1
	Clustered:	False
	DistinctCount:	2
	Foreign:	False
	IgnoreNulls:	False
	Name:	Result_ID
	Primary:	False
	Required:	False
	Unique:	False
	Fields:	
	Result_ID	Ascending
ID		1
	Clustered:	False
	DistinctCount:	2
	Foreign:	False

	IgnoreNulls:	False
	Name:	ID
	Primary:	False
	Required:	False
	Unique:	False
	Fields:	
	ID	Ascending
ID_LIMS		1
	Clustered:	False
	DistinctCount:	2
	Foreign:	False
	IgnoreNulls:	False
	Name:	ID_LIMS
	Primary:	False
	Required:	False
	Unique:	False
	Fields:	
	ID_LIMS	Ascending
PrimaryKey		1
	Clustered:	False
	DistinctCount:	2
	Foreign:	False
	IgnoreNulls:	False
	Name:	PrimaryKey
	Primary:	True
	Required:	True
	Unique:	True
	Fields:	
	ID	Ascending
PrimaryKey		1
	Clustered:	False
	DistinctCount:	3
	Foreign:	False
	IgnoreNulls:	False
	Name:	PrimaryKey
	Primary:	True
	Required:	True
	Unique:	True
	Fields:	
	Protokoll ID	Ascending
ProtokollArt		1
	Clustered:	False
	DistinctCount:	1
	Foreign:	False
	IgnoreNulls:	False
	Name:	ProtokollArt
	Primary:	False
	Required:	False
	Unique:	False
	Fields:	
	Art	Ascending
ArbeitsgruppenPflanzentransformation		1
	Clustered:	False

	DistinctCount:	1
	Foreign:	True
	IgnoreNulls:	False
	Name:	ArbeitsgruppenPflanzentransformation
	Primary:	False
	Required:	False
	Unique:	False
	Fields:	
	AG	Ascending
Id Nummer		1
	Clustered:	False
	DistinctCount:	4
	Foreign:	False
	IgnoreNulls:	False
	Name:	Id Nummer
	Primary:	False
	Required:	False
	Unique:	False
	Fields:	
	GVO Nummer	Ascending
Number of rooted LInes		1
	Clustered:	False
	DistinctCount:	3
	Foreign:	False
	IgnoreNulls:	False
	Name:	Number of rooted LInes
	Primary:	False
	Required:	False
	Unique:	False
	Fields:	
	Number of rooted LInes	Ascending
Number of Shots		1
	Clustered:	False
	DistinctCount:	2
	Foreign:	False
	IgnoreNulls:	False
	Name:	Number of Shots
	Primary:	False
	Required:	False
	Unique:	False
	Fields:	
	Number of Shots	Ascending
OperatorPflanzentransformation		1
	Clustered:	False
	DistinctCount:	2
	Foreign:	True
	IgnoreNulls:	False
	Name:	OperatorPflanzentransformation
	Primary:	False
	Required:	False
	Unique:	False
	Fields:	
	Worker	Ascending

PrimaryKey		1
	Clustered:	False
	DistinctCount:	7
	Foreign:	False
	IgnoreNulls:	False
	Name:	PrimaryKey
	Primary:	True
	Required:	True
	Unique:	True
	Fields:	
	ID	Ascending
Result_ID		1
	Clustered:	False
	DistinctCount:	2
	Foreign:	False
	IgnoreNulls:	False
	Name:	Result_ID
	Primary:	False
	Required:	False
	Unique:	False
	Fields:	
	Result_ID	Ascending
Old_ID		1
	Clustered:	False
	DistinctCount:	4
	Foreign:	False
	IgnoreNulls:	False
	Name:	Old_ID
	Primary:	False
	Required:	False
	Unique:	False
	Fields:	
	Old_ID	Ascending
PrimaryKey		1
	Clustered:	False
	DistinctCount:	9
	Foreign:	False
	IgnoreNulls:	False
	Name:	PrimaryKey
	Primary:	True
	Required:	True
	Unique:	True
	Fields:	
	ID	Ascending
ArbeitsgruppenPflanzentransformation		1
	Clustered:	False
	DistinctCount:	1
	Foreign:	True
	IgnoreNulls:	False
	Name:	ArbeitsgruppenPflanzentransformation
	Primary:	False
	Required:	False
	Unique:	False

	Fields:	
	AG	Ascending
Id Nummer		1
	Clustered:	False
	DistinctCount:	4
	Foreign:	False
	IgnoreNulls:	False
	Name:	Id Nummer
	Primary:	False
	Required:	False
	Unique:	False
	Fields:	
	GVO Nummer	Ascending
Number of rooted LInes		1
	Clustered:	False
	DistinctCount:	3
	Foreign:	False
	IgnoreNulls:	False
	Name:	Number of rooted LInes
	Primary:	False
	Required:	False
	Unique:	False
	Fields:	
	Number of rooted LInes	Ascending
Number of Shots		1
	Clustered:	False
	DistinctCount:	2
	Foreign:	False
	IgnoreNulls:	False
	Name:	Number of Shots
	Primary:	False
	Required:	False
	Unique:	False
	Fields:	
	Number of Shots	Ascending
OperatorPflanzentransformation		1
	Clustered:	False
	DistinctCount:	2
	Foreign:	True
	IgnoreNulls:	False
	Name:	OperatorPflanzentransformation
	Primary:	False
	Required:	False
	Unique:	False
	Fields:	
	Worker	Ascending
PrimaryKey		1
	Clustered:	False
	DistinctCount:	7
	Foreign:	False
	IgnoreNulls:	False
	Name:	PrimaryKey
	Primary:	True

	Required:	True
	Unique:	True
	Fields:	
	ID	Ascending
Result_ID		1
	Clustered:	False
	DistinctCount:	2
	Foreign:	False
	IgnoreNulls:	False
	Name:	Result_ID
	Primary:	False
	Required:	False
	Unique:	False
	Fields:	
	Result_ID	Ascending

User Permissions

admin	Delete; Read Permissions; Set Permissions; Change Owner, Read Definition; Write Definition; Read Data; Insert Data; Update Data; Delete Data

Group Permissions

Admins	Delete; Read Permissions; Set Permissions; Change Owner, Read Definition; Write Definition; Read Data; Insert Data; Update Data; Delete Data
Users	Delete; Read Permissions; Set Permissions; Change Owner, Read Definition; Write Definition; Read Data; Insert Data; Update Data; Delete Data

Properties

DateCreated:	15.05.2009 14:08:11	DefaultView:	2
DOL:	Long binary data	FilterOnLoad:	False
GUID:	{guid {96E8EE05-3D22-4D15-BACA-C7DF55EB1F1D}}	LastUpdated:	26.10.2009 15:26:32
MaxRecords:	0	ODBCTimeout:	60
OrderByOn:	False	OrderByOnLoad:	True
Orientation:	Left-to-Right	RecordLocks:	Edited Record
RecordsAffected:	0	RecordsetType:	Dynaset
ReturnsRecords:	True	TotalsRow:	False
Type:	64	Updatable:	True
UseTransaction:	True		

SQL

```
INSERT INTO Laborbuch ( Trafoid, [GVO Nummer], Operator, Transformationsdatum, Datum, Prozess,
Protokollname, Experimentmedienid, Resistenz, Bemerkungen )
SELECT Pflanzentransformation.ID AS Trafoid, Pflanzentransformation.[GVO Nummer], Operator.Operator,
Pflanzentransformation.Transformationsdatum, [Transformationsdatum]+[ZeitvonStart] AS Datum,
Transformationsschritte.Prozess, Protokoll.Protokollname, ([Medien ID]*1) AS Experimentmedienid, [Resistenz
Pflanze].Resistenz, Transformationsschritte.Bemerkungen
FROM (Operator INNER JOIN ([Resistenz Pflanze] INNER JOIN (Construct INNER JOIN ((Protokoll INNER
JOIN Pflanzentransformation ON Protokoll.[Protokoll ID] = Pflanzentransformation.Protokoll) INNER JOIN
Transformationsschritte ON Protokoll.[Protokoll ID] = Transformationsschritte.Art) ON Construct.ConstructId =
Pflanzentransformation.[GVO Nummer]) ON [Resistenz Pflanze].ID = Construct.[Plant Resistance]) ON
Operator.ID = Pflanzentransformation.Worker) INNER JOIN (Medien INNER JOIN Media_Step ON
Medien.[Medien ID] = Media_Step.Media_id) ON Transformationsschritte.ID = Media_Step.Trafostep_id
WHERE (((Pflanzentransformation.ID)=[Forms]![Agrobacteria mediated transformation]![ID]))
ORDER BY [Transformationsdatum]+[ZeitvonStart];
```

Query Parameters

Name	Type
[Forms]![Agrobacteria mediated transformation]![ID]	Text

User Permissions

admin	Delete; Read Permissions; Set Permissions; Change Owner, Read Definition; Write Definition; Read Data; Insert Data; Update Data; Delete Data

Group Permissions

Admins	Delete; Read Permissions; Set Permissions; Change Owner, Read Definition; Write Definition; Read Data; Insert Data; Update Data; Delete Data
Users	Delete; Read Permissions; Set Permissions; Change Owner, Read Definition; Write Definition; Read Data; Insert Data; Update Data; Delete Data

Properties

DateCreated:	15.05.2009 14:08:11	DefaultView:	2
DOL:	Long binary data	FilterOnLoad:	False
GUID:	{guid {ABCF268A-4659-4845-B896-9CAEA3B4A54F}}	LastUpdated:	26.10.2009 15:27:02
MaxRecords:	0	ODBCTimeout:	60
OrderByOn:	False	OrderByOnLoad:	True
Orientation:	Left-to-Right	RecordLocks:	Edited Record
RecordsAffected:	0	RecordsetType:	Dynaset
ReturnsRecords:	True	TotalsRow:	False
Type:	64	Updatable:	True
UseTransaction:	True		

SQL

```
INSERT INTO Laborbuch ( Trafoid, [GVO Nummer], Operator, Transformationsdatum, Datum, Prozess,
Protokollname, Experimentmedienid, Bemerkungen )
SELECT Pflanzentransformation.ID AS Trafoid, Pflanzentransformation.[GVO Nummer], Operator.Operator,
Pflanzentransformation.Transformationsdatum, [Transformationsdatum]+[ZeitvonStart] AS Datum,
Transformationsschritte.Prozess, Protokoll.Protokollname, ([Medien ID]*1) AS Experimentmedienid,
Transformationsschritte.Bemerkungen
FROM (Operator INNER JOIN ((Protokoll INNER JOIN Pflanzentransformation ON Protokoll.[Protokoll ID] =
Pflanzentransformation.Protokoll) INNER JOIN Transformationsschritte ON Protokoll.[Protokoll ID] =
Transformationsschritte.Art) ON Operator.ID = Pflanzentransformation.Worker) INNER JOIN (Medien INNER
JOIN Media_Step ON Medien.[Medien ID] = Media_Step.Media_id) ON Transformationsschritte.ID =
Media_Step.Trafostep_id
WHERE (((Pflanzentransformation.ID)=[Forms]![Ballistic transformation]![ID]))
ORDER BY [Transformationsdatum]+[ZeitvonStart];
```

Query Parameters

Name	Type
[Forms]![Ballistic transformation]![ID]	Text

User Permissions

admin	Delete; Read Permissions; Set Permissions; Change Owner, Read Definition; Write Definition; Read Data; Insert Data; Update Data; Delete Data

Group Permissions

Admins	Delete; Read Permissions; Set Permissions; Change Owner, Read Definition; Write Definition; Read Data; Insert Data; Update Data; Delete Data
Users	Delete; Read Permissions; Set Permissions; Change Owner, Read Definition; Write Definition; Read Data; Insert Data; Update Data; Delete Data

Properties

DateCreated:	25.06.2009 10:46:15	DefaultView:	2
DOL:	Long binary data	FilterOnLoad:	False
GUID:	{guid {3115E549-E8AE-49BC-BCEE-DC81E474EAF0}}	LastUpdated:	14.08.2009 14:37:40
MaxRecords:	0	ODBCTimeout:	60
OrderByOn:	False	OrderByOnLoad:	True
Orientation:	Left-to-Right	RecordLocks:	No Locks
RecordsAffected:	0	RecordsetType:	Dynaset
ReturnsRecords:	True	TotalsRow:	False
Type:	0	Updatable:	True

SQL

```
SELECT Construct.ConstructId, Construct.ConstructName, [Resistenz Bakterien].Resistenz, [Resistenz
Pflanze].Resistenz, PlasmidApproval.Plasmidmapcheck, PlasmidApproval.Plasmidmapcheckd,
PlasmidApproval.PlasmidFile
FROM [Resistenz Pflanze] RIGHT JOIN ([Resistenz Bakterien] RIGHT JOIN (Construct LEFT JOIN
PlasmidApproval ON Construct.ConstructId = PlasmidApproval.ConstructId) ON [Resistenz Bakterien].ID =
Construct.[M Resistance]) ON [Resistenz Pflanze].ID = Construct.[Plant Resistance];
```

Columns

Name	Type	Size
ConstructId	Long Integer	4

AggregateType:	-1
AllowZeroLength:	False
AppendOnly:	False
Attributes:	Fixed Size; Updatable
CollatingOrder:	General
ColumnHidden:	False
ColumnOrder:	Default
ColumnWidth:	Default
DataUpdatable:	True
DecimalPlaces:	Auto
DisplayControl:	Text Box
OrdinalPosition:	0
Required:	False
SourceField:	ConstructId
SourceTable:	Construct
TextAlign:	General

Name	Type	Size
ConstructName	Text	255

AggregateType:	-1
AllowZeroLength:	True
AppendOnly:	False
Attributes:	Variable Length; Updatable
CollatingOrder:	General
ColumnHidden:	False
ColumnOrder:	Default
ColumnWidth:	2700
DataUpdatable:	True
DisplayControl:	Text Box

IMEMode: 0
IMESentenceMode: 3
OrdinalPosition: 1
Required: False
SourceField: ConstructName
SourceTable: Construct
TextAlign: General
UnicodeCompression: False

Resistenz Bakterien.Resistenz Text 50
AggregateType: -1
AllowZeroLength: True
AppendOnly: False
Attributes: Variable Length; Updatable
CollatingOrder: General
ColumnHidden: False
ColumnOrder: Default
ColumnWidth: 2160
DataUpdatable: True
DisplayControl: Text Box
GUID: {guid {E2D30250-2F7D-4321-8B7F-BF08B22AB9CF}}
IMEMode: 0
IMESentenceMode: 3
OrdinalPosition: 2
Required: False
SourceField: Resistenz
SourceTable: Resistenz Bakterien
TextAlign: General
UnicodeCompression: True

Resistenz Pflanze.Resistenz Text 50
AggregateType: -1
AllowZeroLength: True
AppendOnly: False
Attributes: Variable Length; Updatable
CollatingOrder: General
ColumnHidden: False
ColumnOrder: Default
ColumnWidth: Default
DataUpdatable: True
DisplayControl: Text Box
GUID: {guid {8D71BD08-D542-4B6A-8796-02E82F37D7E2}}
IMEMode: 0
IMESentenceMode: 3
OrdinalPosition: 3
Required: False
SourceField: Resistenz
SourceTable: Resistenz Pflanze
TextAlign: General
UnicodeCompression: True

Plasmidmapcheck Text 255
AggregateType: -1
AllowZeroLength: True
AppendOnly: False
Attributes: Variable Length; Updatable

CollatingOrder:	General
ColumnHidden:	False
ColumnOrder:	Default
ColumnWidth:	2085
DataUpdatable:	True
DisplayControl:	Text Box
GUID:	{guid {F34DE3DD-1073-4E8D-9FC7-718DE366FF6D}}
IMEMode:	0
IMESentenceMode:	3
OrdinalPosition:	4
Required:	False
SourceField:	Plasmidmapcheck
SourceTable:	PlasmidApproval
TextAlign:	General
UnicodeCompression:	True

Plasmidmapcheckd Date/Time 8

AggregateType:	-1
AllowZeroLength:	False
AppendOnly:	False
Attributes:	Fixed Size; Updatable
CollatingOrder:	General
ColumnHidden:	False
ColumnOrder:	Default
ColumnWidth:	2205
DataUpdatable:	True
GUID:	{guid {1CB9781E-482D-45DD-B68F-7FCDEE13E2E2}}
IMEMode:	0
IMESentenceMode:	3
OrdinalPosition:	5
Required:	False
ShowDatePicker:	For dates
SourceField:	Plasmidmapcheckd
SourceTable:	PlasmidApproval
TextAlign:	General

PlasmidFile Text 255

AggregateType:	-1
AllowZeroLength:	True
AppendOnly:	False
Attributes:	Variable Length; Updatable
CollatingOrder:	General
ColumnHidden:	False
ColumnOrder:	Default
ColumnWidth:	Default
DataUpdatable:	True
DisplayControl:	Text Box
GUID:	{guid {E0F98D8E-EE64-4603-AF91-90D24FC2832D}}
IMEMode:	0
IMESentenceMode:	3
OrdinalPosition:	6
Required:	False
SourceField:	PlasmidFile
SourceTable:	PlasmidApproval
TextAlign:	General
UnicodeCompression:	True

Table Indexes

Name	Number of Fields
ConstructId	1
Clustered:	False
DistinctCount:	16
Foreign:	False
IgnoreNulls:	False
Name:	ConstructId
Primary:	True
Required:	True
Unique:	True
Fields:	
ConstructId	Ascending
ID	1
Clustered:	False
DistinctCount:	15
Foreign:	False
IgnoreNulls:	False
Name:	ID
Primary:	False
Required:	False
Unique:	False
Fields:	
ID	Ascending
PrimaryKey	1
Clustered:	False
DistinctCount:	15
Foreign:	False
IgnoreNulls:	False
Name:	PrimaryKey
Primary:	True
Required:	True
Unique:	True
Fields:	
ID	Ascending
ID	1
Clustered:	False
DistinctCount:	12
Foreign:	False
IgnoreNulls:	False
Name:	ID
Primary:	False
Required:	False
Unique:	False
Fields:	
ID	Ascending
PrimaryKey	1
Clustered:	False
DistinctCount:	12
Foreign:	False
IgnoreNulls:	False

	Name:	PrimaryKey
	Primary:	True
	Required:	True
	Unique:	True
	Fields:	
	ID	Ascending
ConstructId		1
	Clustered:	False
	DistinctCount:	6
	Foreign:	False
	IgnoreNulls:	False
	Name:	ConstructId
	Primary:	False
	Required:	False
	Unique:	False
	Fields:	
	ConstructId	Ascending
PrimaryKey		1
	Clustered:	False
	DistinctCount:	6
	Foreign:	False
	IgnoreNulls:	False
	Name:	PrimaryKey
	Primary:	True
	Required:	True
	Unique:	True
	Fields:	
	ConstructId	Ascending

User Permissions

admin	Delete; Read Permissions; Set Permissions; Change Owner, Read Definition; Write Definition; Read Data; Insert Data; Update Data; Delete Data

Group Permissions

Admins	Delete; Read Permissions; Set Permissions; Change Owner, Read Definition; Write Definition; Read Data; Insert Data; Update Data; Delete Data
Users	Delete; Read Permissions; Set Permissions; Change Owner, Read Definition; Write Definition; Read Data; Insert Data; Update Data; Delete Data

Properties

DateCreated:	16.10.2009 10:53:31	DefaultView:	2
DOL:	Long binary data	FilterOnLoad:	False
GUID:	{guid {939B3C0D-2AAF-4FE3-98D5-CC2FBD9204B7}}	LastUpdated:	16.10.2009 11:02:58
MaxRecords:	0	ODBCTimeout:	60
OrderByOn:	False	OrderByOnLoad:	True
Orientation:	Left-to-Right	RecordLocks:	Edited Record
RecordsAffected:	0	RecordsetType:	Dynaset
ReturnsRecords:	True	TotalsRow:	False
Type:	64	Updatable:	True
UseTransaction:	True		

SQL

```
INSERT INTO Media_Step ( Trafostep_id, Media_id, Remark, [Container], Bezeichner )
SELECT CopyMedia_StepsForSingleTrafoStep_select_1.ID,
CopyMedia_StepsForSingleTrafoStep_select_1.Media_id,
[CopyMedia_StepsForSingleTrafoStep_select_1.Remark]+'_new' AS Expr1,
CopyMedia_StepsForSingleTrafoStep_select_1.Container,
CopyMedia_StepsForSingleTrafoStep_select_1.Bezeichner
FROM CopyMedia_StepsForSingleTrafoStep_select_1;
```

Query Parameters

Name	Type
[Forms]![Method form E_1]![SelectedTrafoStep]	Text

User Permissions

admin	Delete; Read Permissions; Set Permissions; Change Owner, Read Definition; Write Definition; Read Data; Insert Data; Update Data; Delete Data

Group Permissions

Admins	Delete; Read Permissions; Set Permissions; Change Owner, Read Definition; Write Definition; Read Data; Insert Data; Update Data; Delete Data
Users	Delete; Read Permissions; Set Permissions; Change Owner, Read Definition; Write Definition; Read Data; Insert Data; Update Data; Delete Data

Properties

DateCreated:	27.03.2009 12:41:20	DefaultView:	2
DOL:	Long binary data	FilterOnLoad:	False
GUID:	{guid {9AB0ABEB-6072-4731-BCF6-1A91D0F792CC}}	LastUpdated:	16.10.2009 11:04:27
MaxRecords:	0	ODBCTimeout:	60
OrderByOn:	False	OrderByOnLoad:	True
Orientation:	Left-to-Right	RecordLocks:	Edited Record
RecordsAffected:	0	RecordsetType:	Dynaset
ReturnsRecords:	True	TotalsRow:	False
Type:	64	Updatable:	True
UseTransaction:	True		

SQL

```
INSERT INTO Media_Step ( Trafostep_id, Media_id, Remark, [Container], Bezeichner )
SELECT CopyMedia_StepsForSingleTrafoStep_select_2.ID,
CopyMedia_StepsForSingleTrafoStep_select_2.Media_id,
[CopyMedia_StepsForSingleTrafoStep_select_2.Remark]+'_new' AS Expr1,
CopyMedia_StepsForSingleTrafoStep_select_2.Container,
CopyMedia_StepsForSingleTrafoStep_select_2.Bezeichner
FROM CopyMedia_StepsForSingleTrafoStep_select_2;
```

Query Parameters

Name	Type
[Forms]![Method form E_2]![SelectedTrafoStep]	Text

User Permissions

admin	Delete; Read Permissions; Set Permissions; Change Owner, Read Definition; Write Definition; Read Data; Insert Data; Update Data; Delete Data

Group Permissions

Admins	Delete; Read Permissions; Set Permissions; Change Owner, Read Definition; Write Definition; Read Data; Insert Data; Update Data; Delete Data
Users	Delete; Read Permissions; Set Permissions; Change Owner, Read Definition; Write Definition; Read Data; Insert Data; Update Data; Delete Data

Properties

DateCreated:	16.10.2009 10:53:31	DefaultView:	2
DOL:	Long binary data	FilterOnLoad:	False
LastUpdated:	16.10.2009 10:54:22	MaxRecords:	0
ODBCTimeout:	60	OrderByOn:	False
OrderByOnLoad:	True	Orientation:	Left-to-Right
RecordLocks:	No Locks	RecordsAffected:	0
RecordsetType:	Dynaset	ReturnsRecords:	True
TotalsRow:	False	Type:	0
Updatable:	True		

SQL

```
SELECT Media_Step.Medien_Schritt_id, Media_Step.Trafostep_id, Media_Step.Media_id, Media_Step.Remark,
Media_Step.Container, Media_Step.Bezeichner, Transformationsschritte.ID, Transformationsschritte.Art
FROM Media_Step INNER JOIN Transformationsschritte ON Media_Step.Trafostep_id =
Transformationsschritte.Old_ID
WHERE (((Media_Step.Trafostep_id)=[Forms]![Method form E_1]![SelectedTrafoStep]));
```

Query Parameters

Name	Type
[Forms]![Method form E_1]![SelectedTrafoStep]	Text

Columns

Name	Type	Size
Medien_Schritt_id	Long Integer	4

AggregateType:	-1
AllowZeroLength:	False
AppendOnly:	False
Attributes:	Fixed Size; Auto-Increment
CollatingOrder:	General
ColumnHidden:	False
ColumnOrder:	1
ColumnWidth:	2130
DataUpdatable:	False
Description:	Primary key
GUID:	{guid {D0AEE457-068A-4190-9CA2-6A61B7803852}}
OrdinalPosition:	0
Required:	False
SourceField:	Medien_Schritt_id
SourceTable:	Media_Step
TextAlign:	General

Name	Type	Size
Trafostep_id	Long Integer	4

AggregateType:	-1
AllowZeroLength:	False
AppendOnly:	False
Attributes:	Fixed Size
CollatingOrder:	General

ColumnHidden:	False
ColumnOrder:	Default
ColumnWidth:	2070
DataUpdatable:	False
DecimalPlaces:	Auto
Description:	Foreign key to Trafosteps_id
DisplayControl:	Text Box
GUID:	{guid {9824CF5E-038F-429C-9D3B-070EBBA00C15}}
OrdinalPosition:	1
Required:	False
SourceField:	Trafostep_id
SourceTable:	Media_Step
TextAlign:	General

Media_id Long Integer 4

AggregateType:	-1
AllowZeroLength:	False
AppendOnly:	False
Attributes:	Fixed Size
CollatingOrder:	General
ColumnHidden:	False
ColumnOrder:	Default
ColumnWidth:	Default
DataUpdatable:	False
DecimalPlaces:	Auto
Description:	Foreign key to Media_id
DisplayControl:	Text Box
GUID:	{guid {BE2523A6-108A-40D8-9386-392BDB8A3332}}
OrdinalPosition:	2
Required:	False
SourceField:	Media_id
SourceTable:	Media_Step
TextAlign:	General

Remark Text 250

AggregateType:	-1
AllowZeroLength:	True
AppendOnly:	False
Attributes:	Variable Length
CollatingOrder:	General
ColumnHidden:	False
ColumnOrder:	Default
ColumnWidth:	Default
DataUpdatable:	False
DisplayControl:	Text Box
GUID:	{guid {FB0F8A63-FF1D-4AB8-B5D4-6C90F0B1FDE1}}
IMEMode:	0
IMESentenceMode:	3
OrdinalPosition:	3
Required:	False
SourceField:	Remark
SourceTable:	Media_Step
TextAlign:	General
UnicodeCompression:	True

Container Long Integer 4

AggregateType:	-1
AllowZeroLength:	False
AppendOnly:	False
Attributes:	Fixed Size
CollatingOrder:	General
ColumnHidden:	False
ColumnOrder:	Default
ColumnWidth:	Default
DataUpdatable:	False
DecimalPlaces:	Auto
DisplayControl:	Text Box
GUID:	{guid {DB8D28F9-A03B-4989-8CA6-34B20203050F}}
OrdinalPosition:	4
Required:	False
SourceField:	Container
SourceTable:	Media_Step
TextAlign:	General

Bezeichner Text 200

AggregateType:	-1
AllowZeroLength:	True
AppendOnly:	False
Attributes:	Variable Length
CollatingOrder:	General
ColumnHidden:	False
ColumnOrder:	Default
ColumnWidth:	Default
DataUpdatable:	False
DisplayControl:	Text Box
GUID:	{guid {7E66055C-35F1-4926-BA22-D7E6221C143A}}
IMEMode:	0
IMESentenceMode:	3
OrdinalPosition:	5
Required:	False
SourceField:	Bezeichner
SourceTable:	Media_Step
TextAlign:	General
UnicodeCompression:	True

ID Long Integer 4

AggregateType:	-1
AllowZeroLength:	False
AppendOnly:	False
Attributes:	Fixed Size; Auto-Increment
CollatingOrder:	General
ColumnHidden:	False
ColumnOrder:	Default
ColumnWidth:	1035
DataUpdatable:	False
GUID:	{guid {C3183DBA-40CD-487D-B59E-DEBC1DA2563C}}
OrdinalPosition:	6
Required:	False
SourceField:	ID
SourceTable:	Transformationsschritte
TextAlign:	General

Art	Long Integer	4

AggregateType:	-1
AllowValueListEdits:	False
AllowZeroLength:	False
AppendOnly:	False
Attributes:	Fixed Size
BoundColumn:	1
CollatingOrder:	General
ColumnCount:	2
ColumnHeads:	False
ColumnHidden:	False
ColumnOrder:	Default
ColumnWidth:	3765
ColumnWidths:	0;3420
DataUpdatable:	False
DecimalPlaces:	Auto
DisplayControl:	Combo Box
GUID:	{guid {E1009E6F-96BB-4EC6-BA36-284C7914E778}}
LimitToList:	True
ListRows:	8
ListWidth:	3420twip
OrdinalPosition:	7
Required:	False
RowSource:	SELECT Protokoll.[Protokoll ID], Protokoll.Protokollname FROM Protokoll;
RowSourceType:	Table/Query
ShowOnlyRowSourceValues:	False
SourceField:	Art
SourceTable:	Transformationsschritte
TextAlign:	General

Table Indexes

Name	Number of Fields
Media_id	1
Clustered:	False
DistinctCount:	7
Foreign:	False
IgnoreNulls:	False
Name:	Media_id
Primary:	False
Required:	False
Unique:	False
Fields:	
Media_id	Ascending
Medien_Schritt_id	1
Clustered:	False
DistinctCount:	11
Foreign:	False
IgnoreNulls:	False
Name:	Medien_Schritt_id
Primary:	False
Required:	False
Unique:	False

	Fields:	
	Medien_Schritt_id	Ascending
MedienMedia_Step		1
	Clustered:	False
	DistinctCount:	7
	Foreign:	True
	IgnoreNulls:	False
	Name:	MedienMedia_Step
	Primary:	False
	Required:	False
	Unique:	False
	Fields:	
	Media_id	Ascending
PrimaryKey		1
	Clustered:	False
	DistinctCount:	11
	Foreign:	False
	IgnoreNulls:	False
	Name:	PrimaryKey
	Primary:	True
	Required:	True
	Unique:	True
	Fields:	
	Medien_Schritt_id	Ascending
TransformationsschritteMedia_Step		1
	Clustered:	False
	DistinctCount:	9
	Foreign:	True
	IgnoreNulls:	False
	Name:	TransformationsschritteMedia_Step
	Primary:	False
	Required:	False
	Unique:	False
	Fields:	
	Trafostep_id	Ascending
Old_ID		1
	Clustered:	False
	DistinctCount:	4
	Foreign:	False
	IgnoreNulls:	False
	Name:	Old_ID
	Primary:	False
	Required:	False
	Unique:	False
	Fields:	
	Old_ID	Ascending
PrimaryKey		1
	Clustered:	False
	DistinctCount:	9
	Foreign:	False
	IgnoreNulls:	False
	Name:	PrimaryKey
	Primary:	True

Required:	True
Unique:	True
Fields:	
ID	Ascending

User Permissions

admin	Delete; Read Permissions; Set Permissions; Change Owner, Read Definition; Write Definition; Read Data; Insert Data; Update Data; Delete Data

Group Permissions

Admins	Delete; Read Permissions; Set Permissions; Change Owner, Read Definition; Write Definition; Read Data; Insert Data; Update Data; Delete Data
Users	Delete; Read Permissions; Set Permissions; Change Owner, Read Definition; Write Definition; Read Data; Insert Data; Update Data; Delete Data

Properties

DateCreated:	27.03.2009 12:41:20	DefaultView:	2
DOL:	Long binary data	FilterOnLoad:	False
GUID:	{guid {743C6E8D-BF21-4DC5-8753-7A3386570B0A}}	LastUpdated:	16.10.2009 11:05:04
MaxRecords:	0	ODBCTimeout:	60
OrderByOn:	False	OrderByOnLoad:	True
Orientation:	Left-to-Right	RecordLocks:	No Locks
RecordsAffected:	0	RecordsetType:	Dynaset
ReturnsRecords:	True	TotalsRow:	False
Type:	0	Updatable:	True

SQL

```
SELECT Media_Step.Medien_Schritt_id, Media_Step.Trafostep_id, Media_Step.Media_id, Media_Step.Remark,
Media_Step.Container, Media_Step.Bezeichner, Transformationsschritte.ID, Transformationsschritte.Art
FROM Media_Step INNER JOIN Transformationsschritte ON Media_Step.Trafostep_id =
Transformationsschritte.Old_ID
WHERE (((Media_Step.Trafostep_id)=[Forms]![Method form E_2]![SelectedTrafoStep]));
```

Query Parameters

Name	Type
[Forms]![Method form E_2]![SelectedTrafoStep]	Text

Columns

Name	Type	Size
Medien_Schritt_id	Long Integer	4

AggregateType:	-1
AllowZeroLength:	False
AppendOnly:	False
Attributes:	Fixed Size; Auto-Increment
CollatingOrder:	General
ColumnHidden:	False
ColumnOrder:	1
ColumnWidth:	2130
DataUpdatable:	False
Description:	Primary key
GUID:	{guid {D0AEE457-068A-4190-9CA2-6A61B7803852}}
OrdinalPosition:	0
Required:	False
SourceField:	Medien_Schritt_id
SourceTable:	Media_Step
TextAlign:	General

Name	Type	Size
Trafostep_id	Long Integer	4

AggregateType:	-1
AllowZeroLength:	False
AppendOnly:	False
Attributes:	Fixed Size

CollatingOrder:	General	
ColumnHidden:	False	
ColumnOrder:	Default	
ColumnWidth:	2070	
DataUpdatable:	False	
DecimalPlaces:	Auto	
Description:	Foreign key to Trafosteps_id	
DisplayControl:	Text Box	
GUID:	{guid {9824CF5E-038F-429C-9D3B-070EBBA00C15}}	
OrdinalPosition:	1	
Required:	False	
SourceField:	Trafostep_id	
SourceTable:	Media_Step	
TextAlign:	General	

Media_id Long Integer 4

AggregateType:	-1
AllowZeroLength:	False
AppendOnly:	False
Attributes:	Fixed Size
CollatingOrder:	General
ColumnHidden:	False
ColumnOrder:	Default
ColumnWidth:	Default
DataUpdatable:	False
DecimalPlaces:	Auto
Description:	Foreign key to Media_id
DisplayControl:	Text Box
GUID:	{guid {BE2523A6-108A-40D8-9386-392BDB8A3332}}
OrdinalPosition:	2
Required:	False
SourceField:	Media_id
SourceTable:	Media_Step
TextAlign:	General

Remark Text 250

AggregateType:	-1
AllowZeroLength:	True
AppendOnly:	False
Attributes:	Variable Length
CollatingOrder:	General
ColumnHidden:	False
ColumnOrder:	Default
ColumnWidth:	Default
DataUpdatable:	False
DisplayControl:	Text Box
GUID:	{guid {FB0F8A63-FF1D-4AB8-B5D4-6C90F0B1FDE1}}
IMEMode:	0
IMESentenceMode:	3
OrdinalPosition:	3
Required:	False
SourceField:	Remark
SourceTable:	Media_Step
TextAlign:	General
UnicodeCompression:	True

Container	Long Integer	4
AggregateType:	-1	
AllowZeroLength:	False	
AppendOnly:	False	
Attributes:	Fixed Size	
CollatingOrder:	General	
ColumnHidden:	False	
ColumnOrder:	Default	
ColumnWidth:	Default	
DataUpdatable:	False	
DecimalPlaces:	Auto	
DisplayControl:	Text Box	
GUID:	{guid {DB8D28F9-A03B-4989-8CA6-34B20203050F}}	
OrdinalPosition:	4	
Required:	False	
SourceField:	Container	
SourceTable:	Media_Step	
TextAlign:	General	
Bezeichner	Text	200
AggregateType:	-1	
AllowZeroLength:	True	
AppendOnly:	False	
Attributes:	Variable Length	
CollatingOrder:	General	
ColumnHidden:	False	
ColumnOrder:	Default	
ColumnWidth:	Default	
DataUpdatable:	False	
DisplayControl:	Text Box	
GUID:	{guid {7E66055C-35F1-4926-BA22-D7E6221C143A}}	
IMEMode:	0	
IMESentenceMode:	3	
OrdinalPosition:	5	
Required:	False	
SourceField:	Bezeichner	
SourceTable:	Media_Step	
TextAlign:	General	
UnicodeCompression:	True	
ID	Long Integer	4
AggregateType:	-1	
AllowZeroLength:	False	
AppendOnly:	False	
Attributes:	Fixed Size; Auto-Increment	
CollatingOrder:	General	
ColumnHidden:	False	
ColumnOrder:	Default	
ColumnWidth:	1035	
DataUpdatable:	False	
GUID:	{guid {C3183DBA-40CD-487D-B59E-DEBC1DA2563C}}	
OrdinalPosition:	6	
Required:	False	
SourceField:	ID	
SourceTable:	Transformationsschritte	
TextAlign:	General	

Art Long Integer 4

AggregateType:	-1
AllowValueListEdits:	False
AllowZeroLength:	False
AppendOnly:	False
Attributes:	Fixed Size
BoundColumn:	1
CollatingOrder:	General
ColumnCount:	2
ColumnHeads:	False
ColumnHidden:	False
ColumnOrder:	Default
ColumnWidth:	3765
ColumnWidths:	0;3420
DataUpdatable:	False
DecimalPlaces:	Auto
DisplayControl:	Combo Box
GUID:	{guid {E1009E6F-96BB-4EC6-BA36-284C7914E778}}
LimitToList:	True
ListRows:	8
ListWidth:	3420twip
OrdinalPosition:	7
Required:	False
RowSource:	SELECT Protokoll.[Protokoll ID], Protokoll.Protokollname FROM Protokoll;
RowSourceType:	Table/Query
ShowOnlyRowSourceValues:	False
SourceField:	Art
SourceTable:	Transformationsschritte
TextAlign:	General

Table Indexes

Name	Number of Fields
Media_id	1

Clustered:	False
DistinctCount:	7
Foreign:	False
IgnoreNulls:	False
Name:	Media_id
Primary:	False
Required:	False
Unique:	False
Fields:	
Media_id	Ascending

Name	Number of Fields
Medien_Schritt_id	1

Clustered:	False
DistinctCount:	11
Foreign:	False
IgnoreNulls:	False
Name:	Medien_Schritt_id
Primary:	False
Required:	False
Unique:	False

	Fields:	
	Medien_Schritt_id	Ascending
MedienMedia_Step		1
	Clustered:	False
	DistinctCount:	7
	Foreign:	True
	IgnoreNulls:	False
	Name:	MedienMedia_Step
	Primary:	False
	Required:	False
	Unique:	False
	Fields:	
	Media_id	Ascending
PrimaryKey		1
	Clustered:	False
	DistinctCount:	11
	Foreign:	False
	IgnoreNulls:	False
	Name:	PrimaryKey
	Primary:	True
	Required:	True
	Unique:	True
	Fields:	
	Medien_Schritt_id	Ascending
TransformationsschritteMedia_Step		1
	Clustered:	False
	DistinctCount:	9
	Foreign:	True
	IgnoreNulls:	False
	Name:	TransformationsschritteMedia_Step
	Primary:	False
	Required:	False
	Unique:	False
	Fields:	
	Trafostep_id	Ascending
Old_ID		1
	Clustered:	False
	DistinctCount:	4
	Foreign:	False
	IgnoreNulls:	False
	Name:	Old_ID
	Primary:	False
	Required:	False
	Unique:	False
	Fields:	
	Old_ID	Ascending
PrimaryKey		1
	Clustered:	False
	DistinctCount:	9
	Foreign:	False
	IgnoreNulls:	False
	Name:	PrimaryKey
	Primary:	True

Required:	True
Unique:	True
Fields:	
ID	Ascending

User Permissions

admin	Delete; Read Permissions; Set Permissions; Change Owner, Read Definition; Write Definition; Read Data; Insert Data; Update Data; Delete Data

Group Permissions

Admins	Delete; Read Permissions; Set Permissions; Change Owner, Read Definition; Write Definition; Read Data; Insert Data; Update Data; Delete Data
Users	Delete; Read Permissions; Set Permissions; Change Owner, Read Definition; Write Definition; Read Data; Insert Data; Update Data; Delete Data

Properties

DateCreated:	16.10.2009 10:53:32	DefaultView:	2
DOL:	Long binary data	FilterOnLoad:	False
GUID:	{guid {3711DADE-A5BD-4E15-9D5B-9795D407D54C}}	LastUpdated:	16.10.2009 11:08:57
MaxRecords:	0	ODBCTimeout:	60
OrderByOn:	False	OrderByOnLoad:	True
Orientation:	Left-to-Right	RecordLocks:	Edited Record
RecordsAffected:	0	ReturnsRecords:	True
Type:	64	Updatable:	True
UseTransaction:	True		

SQL

```
INSERT INTO Media_Step ( Trafostep_id, Media_id, Bezeichner, [Container], Remark )
SELECT CopyMediaStep_select_1.ID, CopyMediaStep_select_1.Media_id, CopyMediaStep_select_1.Bezeichner,
CopyMediaStep_select_1.Container, CopyMediaStep_select_1.Remark
FROM CopyMediaStep_select_1;
```

User Permissions

admin	Delete; Read Permissions; Set Permissions; Change Owner, Read Definition; Write Definition; Read Data; Insert Data; Update Data; Delete Data

Group Permissions

Admins	Delete; Read Permissions; Set Permissions; Change Owner, Read Definition; Write Definition; Read Data; Insert Data; Update Data; Delete Data
Users	Delete; Read Permissions; Set Permissions; Change Owner, Read Definition; Write Definition; Read Data; Insert Data; Update Data; Delete Data

Properties

DateCreated:	27.03.2009 12:41:20	DefaultView:	2
DOL:	Long binary data	FilterOnLoad:	False
GUID:	{guid {57A22062-1BFA-4BF6-A162-A5C0CA0B8A9C}}	LastUpdated:	16.10.2009 11:10:17
MaxRecords:	0	ODBCTimeout:	60
OrderByOn:	False	OrderByOnLoad:	True
Orientation:	Left-to-Right	RecordLocks:	Edited Record
RecordsAffected:	0	ReturnsRecords:	True
Type:	64	Updatable:	True
UseTransaction:	True		

SQL

```
INSERT INTO Media_Step ( Trafostep_id, Media_id, Bezeichner, [Container], Remark )
SELECT CopyMediaStep_select_2.ID, CopyMediaStep_select_2.Media_id, CopyMediaStep_select_2.Bezeichner,
CopyMediaStep_select_2.Container, CopyMediaStep_select_2.Remark
FROM CopyMediaStep_select_2;
```

User Permissions

admin	Delete; Read Permissions; Set Permissions; Change Owner, Read Definition; Write Definition; Read Data; Insert Data; Update Data; Delete Data

Group Permissions

Admins	Delete; Read Permissions; Set Permissions; Change Owner, Read Definition; Write Definition; Read Data; Insert Data; Update Data; Delete Data
Users	Delete; Read Permissions; Set Permissions; Change Owner, Read Definition; Write Definition; Read Data; Insert Data; Update Data; Delete Data

Properties

DateCreated:	16.10.2009 10:53:32	DefaultView:	2
DOL:	Long binary data	FilterOnLoad:	False
GUID:	{guid {7B9DA172-336E-43E6-98B6-BE282DD9D888}}	LastUpdated:	16.10.2009 11:11:14
MaxRecords:	0	ODBCTimeout:	60
OrderByOn:	False	OrderByOnLoad:	True
Orientation:	Left-to-Right	RecordLocks:	No Locks
RecordsAffected:	0	RecordsetType:	Dynaset
ReturnsRecords:	True	TotalsRow:	False
Type:	0	Updatable:	True

SQL

```
SELECT Media_Step.Medien_Schritt_id, Media_Step.Trafostep_id, Media_Step.Media_id, Media_Step.Remark,
Media_Step.Container, Media_Step.Bezeichner, Transformationsschritte.ID
FROM (Media_Step INNER JOIN Transformationsschritte ON
Media_Step.Trafostep_id=Transformationsschritte.Old_ID) INNER JOIN
CopyProtokoll_Select_NewProtokollID_1 ON
Transformationsschritte.Art=CopyProtokoll_Select_NewProtokollID_1.[Protokoll ID];
```

Columns

Name	Type	Size
Medien_Schritt_id	Long Integer	4

AggregateType:	-1
AllowZeroLength:	False
AppendOnly:	False
Attributes:	Fixed Size; Auto-Increment
CollatingOrder:	General
ColumnHidden:	False
ColumnOrder:	1
ColumnWidth:	2130
DataUpdatable:	False
Description:	Primary key
GUID:	{guid {D0AEE457-068A-4190-9CA2-6A61B7803852}}
OrdinalPosition:	0
Required:	False
SourceField:	Medien_Schritt_id
SourceTable:	Media_Step
TextAlign:	General

Name	Type	Size
Trafostep_id	Long Integer	4

AggregateType:	-1
AllowZeroLength:	False
AppendOnly:	False
Attributes:	Fixed Size
CollatingOrder:	General
ColumnHidden:	False
ColumnOrder:	Default
ColumnWidth:	2070
DataUpdatable:	False
DecimalPlaces:	Auto

Description:	Foreign key to Trafosteps_id	
DisplayControl:	Text Box	
GUID:	{guid {9824CF5E-038F-429C-9D3B-070EBBA00C15}}	
OrdinalPosition:	1	
Required:	False	
SourceField:	Trafostep_id	
SourceTable:	Media_Step	
TextAlign:	General	

Media_id	Long Integer	4
AggregateType:	-1	
AllowZeroLength:	False	
AppendOnly:	False	
Attributes:	Fixed Size	
CollatingOrder:	General	
ColumnHidden:	False	
ColumnOrder:	Default	
ColumnWidth:	Default	
DataUpdatable:	False	
DecimalPlaces:	Auto	
Description:	Foreign key to Media_id	
DisplayControl:	Text Box	
GUID:	{guid {BE2523A6-108A-40D8-9386-392BDB8A3332}}	
OrdinalPosition:	2	
Required:	False	
SourceField:	Media_id	
SourceTable:	Media_Step	
TextAlign:	General	

Remark	Text	250
AggregateType:	-1	
AllowZeroLength:	True	
AppendOnly:	False	
Attributes:	Variable Length	
CollatingOrder:	General	
ColumnHidden:	False	
ColumnOrder:	Default	
ColumnWidth:	Default	
DataUpdatable:	False	
DisplayControl:	Text Box	
GUID:	{guid {FB0F8A63-FF1D-4AB8-B5D4-6C90F0B1FDE1}}	
IMEMode:	0	
IMESentenceMode:	3	
OrdinalPosition:	3	
Required:	False	
SourceField:	Remark	
SourceTable:	Media_Step	
TextAlign:	General	
UnicodeCompression:	True	

Container	Long Integer	4
AggregateType:	-1	
AllowZeroLength:	False	
AppendOnly:	False	
Attributes:	Fixed Size	
CollatingOrder:	General	

ColumnHidden:	False
ColumnOrder:	Default
ColumnWidth:	Default
DataUpdatable:	False
DecimalPlaces:	Auto
DisplayControl:	Text Box
GUID:	{guid {DB8D28F9-A03B-4989-8CA6-34B20203050F}}
OrdinalPosition:	4
Required:	False
SourceField:	Container
SourceTable:	Media_Step
TextAlign:	General

Bezeichner Text 200

AggregateType:	-1
AllowZeroLength:	True
AppendOnly:	False
Attributes:	Variable Length
CollatingOrder:	General
ColumnHidden:	False
ColumnOrder:	Default
ColumnWidth:	Default
DataUpdatable:	False
DisplayControl:	Text Box
GUID:	{guid {7E66055C-35F1-4926-BA22-D7E6221C143A}}
IMEMode:	0
IMESentenceMode:	3
OrdinalPosition:	5
Required:	False
SourceField:	Bezeichner
SourceTable:	Media_Step
TextAlign:	General
UnicodeCompression:	True

ID Long Integer 4

AggregateType:	-1
AllowZeroLength:	False
AppendOnly:	False
Attributes:	Fixed Size; Auto-Increment
CollatingOrder:	General
ColumnHidden:	False
ColumnOrder:	Default
ColumnWidth:	1035
DataUpdatable:	False
GUID:	{guid {C3183DBA-40CD-487D-B59E-DEBC1DA2563C}}
OrdinalPosition:	6
Required:	False
SourceField:	ID
SourceTable:	Transformationsschritte
TextAlign:	General

Table Indexes

Name	Number of Fields
Media_id	1

	Clustered:	False
	DistinctCount:	7
	Foreign:	False
	IgnoreNulls:	False
	Name:	Media_id
	Primary:	False
	Required:	False
	Unique:	False
	Fields:	
	Media_id	Ascending
Medien_Schritt_id		1
	Clustered:	False
	DistinctCount:	11
	Foreign:	False
	IgnoreNulls:	False
	Name:	Medien_Schritt_id
	Primary:	False
	Required:	False
	Unique:	False
	Fields:	
	Medien_Schritt_id	Ascending
MedienMedia_Step		1
	Clustered:	False
	DistinctCount:	7
	Foreign:	True
	IgnoreNulls:	False
	Name:	MedienMedia_Step
	Primary:	False
	Required:	False
	Unique:	False
	Fields:	
	Media_id	Ascending
PrimaryKey		1
	Clustered:	False
	DistinctCount:	11
	Foreign:	False
	IgnoreNulls:	False
	Name:	PrimaryKey
	Primary:	True
	Required:	True
	Unique:	True
	Fields:	
	Medien_Schritt_id	Ascending
TransformationsschritteMedia_Step		1
	Clustered:	False
	DistinctCount:	9
	Foreign:	True
	IgnoreNulls:	False
	Name:	TransformationsschritteMedia_Step
	Primary:	False
	Required:	False
	Unique:	False

	Fields:	
	Trafostep_id	Ascending
Old_ID		1
	Clustered:	False
	DistinctCount:	4
	Foreign:	False
	IgnoreNulls:	False
	Name:	Old_ID
	Primary:	False
	Required:	False
	Unique:	False
	Fields:	
	Old_ID	Ascending
PrimaryKey		1
	Clustered:	False
	DistinctCount:	9
	Foreign:	False
	IgnoreNulls:	False
	Name:	PrimaryKey
	Primary:	True
	Required:	True
	Unique:	True
	Fields:	
	ID	Ascending

User Permissions

admin	Delete; Read Permissions; Set Permissions; Change Owner, Read Definition; Write Definition; Read Data; Insert Data; Update Data; Delete Data

Group Permissions

Admins	Delete; Read Permissions; Set Permissions; Change Owner, Read Definition; Write Definition; Read Data; Insert Data; Update Data; Delete Data
Users	Delete; Read Permissions; Set Permissions; Change Owner, Read Definition; Write Definition; Read Data; Insert Data; Update Data; Delete Data

Properties

DateCreated:	27.03.2009 12:41:20	DefaultView:	2
DOL:	Long binary data	FilterOnLoad:	False
GUID:	{guid {95C81B53-7F99-4576-8C52-C9D7BCCDC81E}}	LastUpdated:	16.10.2009 11:11:43
MaxRecords:	0	ODBCTimeout:	60
OrderByOn:	False	OrderByOnLoad:	True
Orientation:	Left-to-Right	RecordLocks:	No Locks
RecordsAffected:	0	RecordsetType:	Dynaset
ReturnsRecords:	True	TotalsRow:	False
Type:	0	Updatable:	True

SQL

```
SELECT Media_Step.Medien_Schritt_id, Media_Step.Trafostep_id, Media_Step.Media_id, Media_Step.Remark,
Media_Step.Container, Media_Step.Bezeichner, Transformationsschritte.ID
FROM (Media_Step INNER JOIN Transformationsschritte ON
Media_Step.Trafostep_id=Transformationsschritte.Old_ID) INNER JOIN
CopyProtokoll_Select_NewProtokollID_2 ON
Transformationsschritte.Art=CopyProtokoll_Select_NewProtokollID_2.[Protokoll ID];
```

Columns

Name	Type	Size
Medien_Schritt_id	Long Integer	4

AggregateType:	-1
AllowZeroLength:	False
AppendOnly:	False
Attributes:	Fixed Size; Auto-Increment
CollatingOrder:	General
ColumnHidden:	False
ColumnOrder:	1
ColumnWidth:	2130
DataUpdatable:	False
Description:	Primary key
GUID:	{guid {D0AEE457-068A-4190-9CA2-6A61B7803852}}
OrdinalPosition:	0
Required:	False
SourceField:	Medien_Schritt_id
SourceTable:	Media_Step
TextAlign:	General

Name	Type	Size
Trafostep_id	Long Integer	4

AggregateType:	-1
AllowZeroLength:	False
AppendOnly:	False
Attributes:	Fixed Size
CollatingOrder:	General
ColumnHidden:	False
ColumnOrder:	Default
ColumnWidth:	2070
DataUpdatable:	False
DecimalPlaces:	Auto

	Description:	Foreign key to Trafosteps_id
	DisplayControl:	Text Box
	GUID:	{guid {9824CF5E-038F-429C-9D3B-070EBBA00C15}}
	OrdinalPosition:	1
	Required:	False
	SourceField:	Trafostep_id
	SourceTable:	Media_Step
	TextAlign:	General
Media_id		Long Integer 4
	AggregateType:	-1
	AllowZeroLength:	False
	AppendOnly:	False
	Attributes:	Fixed Size
	CollatingOrder:	General
	ColumnHidden:	False
	ColumnOrder:	Default
	ColumnWidth:	Default
	DataUpdatable:	False
	DecimalPlaces:	Auto
	Description:	Foreign key to Media_id
	DisplayControl:	Text Box
	GUID:	{guid {BE2523A6-108A-40D8-9386-392BDB8A3332}}
	OrdinalPosition:	2
	Required:	False
	SourceField:	Media_id
	SourceTable:	Media_Step
	TextAlign:	General
Remark		Text 250
	AggregateType:	-1
	AllowZeroLength:	True
	AppendOnly:	False
	Attributes:	Variable Length
	CollatingOrder:	General
	ColumnHidden:	False
	ColumnOrder:	Default
	ColumnWidth:	Default
	DataUpdatable:	False
	DisplayControl:	Text Box
	GUID:	{guid {FB0F8A63-FF1D-4AB8-B5D4-6C90F0B1FDE1}}
	IMEMode:	0
	IMESentenceMode:	3
	OrdinalPosition:	3
	Required:	False
	SourceField:	Remark
	SourceTable:	Media_Step
	TextAlign:	General
	UnicodeCompression:	True
Container		Long Integer 4
	AggregateType:	-1
	AllowZeroLength:	False
	AppendOnly:	False
	Attributes:	Fixed Size
	CollatingOrder:	General

ColumnHidden:	False
ColumnOrder:	Default
ColumnWidth:	Default
DataUpdatable:	False
DecimalPlaces:	Auto
DisplayControl:	Text Box
GUID:	{guid {DB8D28F9-A03B-4989-8CA6-34B20203050F}}
OrdinalPosition:	4
Required:	False
SourceField:	Container
SourceTable:	Media_Step
TextAlign:	General

Bezeichner Text 200

AggregateType:	-1
AllowZeroLength:	True
AppendOnly:	False
Attributes:	Variable Length
CollatingOrder:	General
ColumnHidden:	False
ColumnOrder:	Default
ColumnWidth:	Default
DataUpdatable:	False
DisplayControl:	Text Box
GUID:	{guid {7E66055C-35F1-4926-BA22-D7E6221C143A}}
IMEMode:	0
IMESentenceMode:	3
OrdinalPosition:	5
Required:	False
SourceField:	Bezeichner
SourceTable:	Media_Step
TextAlign:	General
UnicodeCompression:	True

ID Long Integer 4

AggregateType:	-1
AllowZeroLength:	False
AppendOnly:	False
Attributes:	Fixed Size; Auto-Increment
CollatingOrder:	General
ColumnHidden:	False
ColumnOrder:	Default
ColumnWidth:	1035
DataUpdatable:	False
GUID:	{guid {C3183DBA-40CD-487D-B59E-DEBC1DA2563C}}
OrdinalPosition:	6
Required:	False
SourceField:	ID
SourceTable:	Transformationsschritte
TextAlign:	General

Table Indexes

Name	Number of Fields
Media_id	1

Clustered:	False
DistinctCount:	7
Foreign:	False
IgnoreNulls:	False
Name:	Media_id
Primary:	False
Required:	False
Unique:	False
Fields:	
Media_id	Ascending
**Medien_Schritt_id**	1
Clustered:	False
DistinctCount:	11
Foreign:	False
IgnoreNulls:	False
Name:	Medien_Schritt_id
Primary:	False
Required:	False
Unique:	False
Fields:	
Medien_Schritt_id	Ascending
**MedienMedia_Step**	1
Clustered:	False
DistinctCount:	7
Foreign:	True
IgnoreNulls:	False
Name:	MedienMedia_Step
Primary:	False
Required:	False
Unique:	False
Fields:	
Media_id	Ascending
**PrimaryKey**	1
Clustered:	False
DistinctCount:	11
Foreign:	False
IgnoreNulls:	False
Name:	PrimaryKey
Primary:	True
Required:	True
Unique:	True
Fields:	
Medien_Schritt_id	Ascending
**TransformationsschritteMedia_Step**	1
Clustered:	False
DistinctCount:	9
Foreign:	True
IgnoreNulls:	False
Name:	TransformationsschritteMedia_Step
Primary:	False
Required:	False
Unique:	False

	Fields:	
	Trafostep_id	Ascending
Old_ID		1
	Clustered:	False
	DistinctCount:	4
	Foreign:	False
	IgnoreNulls:	False
	Name:	Old_ID
	Primary:	False
	Required:	False
	Unique:	False
	Fields:	
	Old_ID	Ascending
PrimaryKey		1
	Clustered:	False
	DistinctCount:	9
	Foreign:	False
	IgnoreNulls:	False
	Name:	PrimaryKey
	Primary:	True
	Required:	True
	Unique:	True
	Fields:	
	ID	Ascending

User Permissions

admin	Delete; Read Permissions; Set Permissions; Change Owner, Read Definition; Write Definition; Read Data; Insert Data; Update Data; Delete Data

Group Permissions

Admins	Delete; Read Permissions; Set Permissions; Change Owner, Read Definition; Write Definition; Read Data; Insert Data; Update Data; Delete Data
Users	Delete; Read Permissions; Set Permissions; Change Owner, Read Definition; Write Definition; Read Data; Insert Data; Update Data; Delete Data

Properties

DateCreated:	06.08.2009 08:55:26	DefaultView:	2
FilterOnLoad:	False	GUID:	{guid {7B57096E-8608-4C79-8A50-16DDFE668A09}}
LastUpdated:	16.10.2009 11:48:17	MaxRecords:	0
ODBCTimeout:	60	OrderByOn:	False
OrderByOnLoad:	True	Orientation:	Left-to-Right
RecordLocks:	Edited Record	RecordsAffected:	0
ReturnsRecords:	True	Type:	64
Updatable:	True	UseTransaction:	True

SQL

```
INSERT INTO Medien ( Protokoll, Medienname, Medienname_D, Bezeichner, Remark, Solvent, Steril,
Storagecondition, Storagesite, Kurs, [Prepare], Volume_Kurs, [General store], [Medien ID old], Supplier )
SELECT CopyMedien_select_1.Protokoll, [Medienname]+'_new' AS Expr1, [Medienname_D]+'_neu' AS Expr2,
CopyMedien_select_1.Bezeichner, CopyMedien_select_1.Remark, CopyMedien_select_1.Solvent,
CopyMedien_select_1.Steril, CopyMedien_select_1.Storagecondition, CopyMedien_select_1.Storagesite,
CopyMedien_select_1.Kurs, CopyMedien_select_1.Prepare, CopyMedien_select_1.Volume_Kurs,
CopyMedien_select_1.[General store], CopyMedien_select_1.[Medien ID], CopyMedien_select_1.Supplier
FROM CopyMedien_select_1;
```

Query Parameters

Name	Type
[Forms]![Media_E_1]![Medien ID]	Text

User Permissions

admin	Delete; Read Permissions; Set Permissions; Change Owner, Read Definition; Write Definition; Read Data; Insert Data; Update Data; Delete Data

Group Permissions

Admins	Delete; Read Permissions; Set Permissions; Change Owner, Read Definition; Write Definition; Read Data; Insert Data; Update Data; Delete Data
Users	Delete; Read Permissions; Set Permissions; Change Owner, Read Definition; Write Definition; Read Data; Insert Data; Update Data; Delete Data

Properties

DateCreated:	16.10.2009 11:45:42	DefaultView:	2
FilterOnLoad:	False	GUID:	{guid {E8FA1462-593C-4EE6-AD36-A2C336C33EAD}}
LastUpdated:	16.10.2009 11:50:45	MaxRecords:	0
ODBCTimeout:	60	OrderByOn:	False
OrderByOnLoad:	True	Orientation:	Left-to-Right
RecordLocks:	Edited Record	RecordsAffected:	0
ReturnsRecords:	True	Type:	64
Updatable:	True	UseTransaction:	True

SQL

```
INSERT INTO Medien ( Protokoll, Medienname, Medienname_D, Bezeichner, Remark, Solvent, Steril,
Storagecondition, Storagesite, Kurs, [Prepare], Volume_Kurs, [General store], [Medien ID old], Supplier )
SELECT CopyMedien_select_2.Protokoll, [Medienname]+'_new' AS Expr1, [Medienname_D]+'_neu' AS Expr2,
CopyMedien_select_2.Bezeichner, CopyMedien_select_2.Remark, CopyMedien_select_2.Solvent,
CopyMedien_select_2.Steril, CopyMedien_select_2.Storagecondition, CopyMedien_select_2.Storagesite,
CopyMedien_select_2.Kurs, CopyMedien_select_2.Prepare, CopyMedien_select_2.Volume_Kurs,
CopyMedien_select_2.[General store], CopyMedien_select_2.[Medien ID], CopyMedien_select_2.Supplier
FROM CopyMedien_select_2;
```

Query Parameters

Name	Type
[Forms]![Media_E_2]![Medien ID]	Text

User Permissions

admin	Delete; Read Permissions; Set Permissions; Change Owner, Read Definition; Write Definition; Read Data; Insert Data; Update Data; Delete Data

Group Permissions

Admins	Delete; Read Permissions; Set Permissions; Change Owner, Read Definition; Write Definition; Read Data; Insert Data; Update Data; Delete Data
Users	Delete; Read Permissions; Set Permissions; Change Owner, Read Definition; Write Definition; Read Data; Insert Data; Update Data; Delete Data

Properties

DateCreated:	06.08.2009 08:55:26	DefaultView:	2
DisplayViewsOnSharePointSit	1	FilterOnLoad:	False
GUID:	{guid {60C57A23-8F2F-440F-A5FE-0F5FD7452258}}	HideNewField:	False
LastUpdated:	16.10.2009 12:10:51	MaxRecords:	0
NameMap:	Long binary data	ODBCTimeout:	60
OrderByOn:	False	OrderByOnLoad:	True
Orientation:	Left-to-Right	RecordLocks:	No Locks
RecordsAffected:	0	RecordsetType:	Dynaset
ReturnsRecords:	True	TotalsRow:	False
Type:	0	Updatable:	True

SQL

```
SELECT Max(Medien.[Medien ID]) AS [Medien ID]
FROM Medien
WHERE (((Medien.[Medien ID old])=[Forms]![Media_E_1]![Medien ID]) AND
((Medien.Medienname)=(SELECT Medien.Medienname FROM Medien WHERE (Medien.[Medien
ID]=[Forms]![Media_E_1]![Medien ID]))+'_new'));
```

Query Parameters

Name	Type
[Forms]![Media_E_1]![Medien ID]	Text

Columns

Name	Type	Size
Medien ID	Long Integer	4

AllowZeroLength:	False
AppendOnly:	False
Attributes:	Fixed Size
CollatingOrder:	General
DataUpdatable:	False
OrdinalPosition:	0
Required:	False

User Permissions

admin	Delete; Read Permissions; Set Permissions; Change Owner, Read Definition; Write Definition; Read Data; Insert Data; Update Data; Delete Data

Group Permissions

Admins	Delete; Read Permissions; Set Permissions; Change Owner, Read Definition; Write Definition; Read Data; Insert Data; Update Data; Delete Data
Users	Delete; Read Permissions; Set Permissions; Change Owner, Read Definition;

Write Definition; Read Data; Insert Data; Update Data; Delete Data

Properties

DateCreated:	16.10.2009 11:51:41	DefaultView:	2
DisplayViewsOnSharePointSit	1	FilterOnLoad:	False
GUID:	{guid {7CC81C76-D337-4E5B-9329-5517945DC902}}	HideNewField:	False
LastUpdated:	16.10.2009 12:11:19	MaxRecords:	0
NameMap:	Long binary data	ODBCTimeout:	60
OrderByOn:	False	OrderByOnLoad:	True
Orientation:	Left-to-Right	RecordLocks:	No Locks
RecordsAffected:	0	RecordsetType:	Dynaset
ReturnsRecords:	True	TotalsRow:	False
Type:	0	Updatable:	True

SQL

```
SELECT Max(Medien.[Medien ID]) AS [Medien ID]
FROM Medien
WHERE (((Medien.[Medien ID old])=[Forms]![Media_E_2]![Medien ID]) AND
((Medien.Medienname)=(SELECT Medien.Medienname FROM Medien WHERE (Medien.[Medien
ID]=[Forms]![Media_E_2]![Medien ID]))+'_new'));
```

Query Parameters

Name	Type
[Forms]![Media_E_2]![Medien ID]	Text

Columns

Name	Type	Size
Medien ID	Long Integer	4

AllowZeroLength:	False
AppendOnly:	False
Attributes:	Fixed Size
CollatingOrder:	General
DataUpdatable:	False
OrdinalPosition:	0
Required:	False

User Permissions

admin	Delete; Read Permissions; Set Permissions; Change Owner, Read Definition; Write Definition; Read Data; Insert Data; Update Data; Delete Data

Group Permissions

Admins	Delete; Read Permissions; Set Permissions; Change Owner, Read Definition; Write Definition; Read Data; Insert Data; Update Data; Delete Data
Users	Delete; Read Permissions; Set Permissions; Change Owner, Read Definition;

Write Definition; Read Data; Insert Data; Update Data; Delete Data

Properties

DateCreated:	06.08.2009 08:55:26	DefaultView:	2
DisplayViewsOnSharePointSit	1	FilterOnLoad:	False
GUID:	{guid {BAEEE97A-46E3-4E88-B934-733F0EC91229}}	HideNewField:	False
LastUpdated:	16.10.2009 11:44:38	MaxRecords:	0
NameMap:	Long binary data	ODBCTimeout:	60
OrderByOn:	False	OrderByOnLoad:	True
Orientation:	Left-to-Right	RecordLocks:	No Locks
RecordsAffected:	0	RecordsetType:	Dynaset
ReturnsRecords:	True	TotalsRow:	False
Type:	0	Updatable:	True

SQL

```
SELECT Medien.Protokoll, Medien.Medienname, Medien.Medienname_D, Medien.Bezeichner, Medien.Remark,
Medien.Solvent, Medien.Steril, Medien.Storagecondition, Medien.Storagesite, Medien.Kurs, Medien.Prepare,
Medien.Volume_Kurs, Medien.[General store], Medien.[Medien ID], Medien.Supplier
FROM Medien
WHERE (((Medien.[Medien ID])=[Forms]![Media_E_1]![Medien ID]));
```

Query Parameters

Name	Type
[Forms]![Media_E_1]![Medien ID]	Text

Columns

Name	Type	Size
Protokoll	Long Integer	4

AggregateType:	-1
AllowZeroLength:	False
AppendOnly:	False
Attributes:	Fixed Size; Updatable
CollatingOrder:	General
ColumnHidden:	False
ColumnOrder:	Default
ColumnWidth:	Default
DataUpdatable:	True
DecimalPlaces:	Auto
DisplayControl:	Text Box
GUID:	{guid {24E1DD39-D007-443F-ACDA-217642A9BE4F}}
OrdinalPosition:	0
Required:	False
SourceField:	Protokoll
SourceTable:	Medien
TextAlign:	General

Name	Type	Size
Medienname	Text	50

AggregateType:	-1
AllowZeroLength:	True

AppendOnly:	False
Attributes:	Variable Length; Updatable
CollatingOrder:	General
ColumnHidden:	False
ColumnOrder:	Default
ColumnWidth:	3615
DataUpdatable:	True
DisplayControl:	Text Box
GUID:	{guid {39A7EAC5-34A3-4BBD-A594-4E88A4399D39}}
IMEMode:	0
IMESentenceMode:	3
OrdinalPosition:	1
Required:	True
SourceField:	Medienname
SourceTable:	Medien
TextAlign:	General
UnicodeCompression:	True

Medienname_D Text 50

AggregateType:	-1
AllowZeroLength:	True
AppendOnly:	False
Attributes:	Variable Length; Updatable
CollatingOrder:	General
ColumnHidden:	False
ColumnOrder:	Default
ColumnWidth:	2955
DataUpdatable:	True
DisplayControl:	Text Box
GUID:	{guid {C9276239-CF36-4089-B820-A7A34A309B03}}
IMEMode:	0
IMESentenceMode:	3
OrdinalPosition:	2
Required:	False
SourceField:	Medienname_D
SourceTable:	Medien
TextAlign:	General
UnicodeCompression:	True

Bezeichner OLE Object N/A

AggregateType:	-1
AllowZeroLength:	False
AppendOnly:	False
Attributes:	Variable Length; Updatable
CollatingOrder:	General
ColumnHidden:	False
ColumnOrder:	Default
ColumnWidth:	1965
DataUpdatable:	True
GUID:	{guid {A52CDB4F-F76D-43DE-B348-217A2AD512CB}}
OrdinalPosition:	3
Required:	False
SourceField:	Bezeichner
SourceTable:	Medien
TextAlign:	General

Remark		Memo	N/A
	AggregateType:	-1	
	AllowZeroLength:	True	
	AppendOnly:	False	
	Attributes:	Variable Length; Updatable	
	CollatingOrder:	General	
	ColumnHidden:	False	
	ColumnOrder:	Default	
	ColumnWidth:	2745	
	DataUpdatable:	True	
	GUID:	{guid {F6E09F04-6090-435D-AAE4-5157DDB09E51}}	
	IMEMode:	0	
	IMESentenceMode:	3	
	OrdinalPosition:	4	
	Required:	False	
	SourceField:	Remark	
	SourceTable:	Medien	
	TextAlign:	General	
	TextFormat:	Plain Text	
	UnicodeCompression:	True	
Solvent		Text	50
	AggregateType:	-1	
	AllowZeroLength:	True	
	AppendOnly:	False	
	Attributes:	Variable Length; Updatable	
	CollatingOrder:	General	
	ColumnHidden:	False	
	ColumnOrder:	Default	
	ColumnWidth:	Default	
	DataUpdatable:	True	
	DisplayControl:	Text Box	
	GUID:	{guid {07F6C1A3-CFAD-4E65-819D-ABBA6FF7B366}}	
	IMEMode:	0	
	IMESentenceMode:	3	
	OrdinalPosition:	5	
	Required:	False	
	SourceField:	Solvent	
	SourceTable:	Medien	
	TextAlign:	General	
	UnicodeCompression:	True	
Steril		Text	50
	AggregateType:	-1	
	AllowZeroLength:	True	
	AppendOnly:	False	
	Attributes:	Variable Length; Updatable	
	CollatingOrder:	General	
	ColumnHidden:	False	
	ColumnOrder:	Default	
	ColumnWidth:	Default	
	DataUpdatable:	True	
	DisplayControl:	Text Box	
	GUID:	{guid {9C185040-3E47-44D4-B4A1-96698F823224}}	
	IMEMode:	0	
	IMESentenceMode:	3	

OrdinalPosition:	6
Required:	False
SourceField:	Steril
SourceTable:	Medien
TextAlign:	General
UnicodeCompression:	True

Storagecondition Long Integer 4

AggregateType:	-1
AllowValueListEdits:	False
AllowZeroLength:	False
AppendOnly:	False
Attributes:	Fixed Size; Updatable
BoundColumn:	1
CollatingOrder:	General
ColumnCount:	2
ColumnHeads:	False
ColumnHidden:	False
ColumnOrder:	Default
ColumnWidth:	2070
ColumnWidths:	0;1440
DataUpdatable:	True
DecimalPlaces:	Auto
DisplayControl:	Combo Box
LimitToList:	True
ListRows:	8
ListWidth:	1440twip
OrdinalPosition:	7
Required:	False
RowSource:	SELECT [Storagecondition].[Storagecondition_ID], [Storagecondition].[Storagecondition_Name] FROM [Storagecondition] ORDER BY [Storagecondition_Name];
RowSourceType:	Table/Query
ShowOnlyRowSourceValues:	False
SourceField:	Storagecondition
SourceTable:	Medien
TextAlign:	General

Storagesite Long Integer 4

AggregateType:	-1
AllowValueListEdits:	False
AllowZeroLength:	False
AppendOnly:	False
Attributes:	Fixed Size; Updatable
BoundColumn:	1
CollatingOrder:	General
ColumnCount:	2
ColumnHeads:	False
ColumnHidden:	False
ColumnOrder:	Default
ColumnWidth:	2925
ColumnWidths:	0;1440
DataUpdatable:	True
DecimalPlaces:	Auto

	DisplayControl:	Combo Box
	LimitToList:	True
	ListRows:	16
	ListWidth:	1440twip
	OrdinalPosition:	8
	Required:	False
	RowSource:	SELECT [Storagesite].[Storagesite_ID], [Storagesite].[Storagesite_Name] FROM [Storagesite] ORDER BY [Storagesite_Name];
	RowSourceType:	Table/Query
	ShowOnlyRowSourceValues:	False
	SourceField:	Storagesite
	SourceTable:	Medien
	TextAlign:	General

Kurs		Yes/No	1
	AggregateType:	-1	
	AllowZeroLength:	False	
	AppendOnly:	False	
	Attributes:	Fixed Size; Updatable	
	CollatingOrder:	General	
	ColumnHidden:	False	
	ColumnOrder:	Default	
	ColumnWidth:	Default	
	DataUpdatable:	True	
	DisplayControl:	106	
	Format:	Yes/No	
	GUID:	{guid {F9E180AA-9465-4BB3-9964-5F2BE293C4EA}}	
	OrdinalPosition:	9	
	Required:	False	
	SourceField:	Kurs	
	SourceTable:	Medien	
	TextAlign:	General	

Prepare		Text	50
	AggregateType:	-1	
	AllowMultipleValues:	False	
	AllowValueListEdits:	False	
	AllowZeroLength:	True	
	AppendOnly:	False	
	Attributes:	Variable Length; Updatable	
	BoundColumn:	1	
	CollatingOrder:	General	
	ColumnCount:	1	
	ColumnHeads:	False	
	ColumnHidden:	False	
	ColumnOrder:	Default	
	ColumnWidth:	Default	
	ColumnWidths:	1440	
	DataUpdatable:	True	
	DisplayControl:	Combo Box	
	GUID:	{guid {E2159DA9-862E-4E06-B5A8-EB44C2D01841}}	
	IMEMode:	0	
	IMESentenceMode:	3	

LimitToList:	False	
ListRows:	8	
ListWidth:	1440twip	
OrdinalPosition:	10	
Required:	False	
RowSource:	"Kurs";"Vorbereitung";"General stock"	
RowSourceType:	Value List	
ShowOnlyRowSourceValues:	False	
SourceField:	Prepare	
SourceTable:	Medien	
TextAlign:	General	
UnicodeCompression:	True	

Volume_Kurs	Text	50
AggregateType:	-1	
AllowZeroLength:	True	
AppendOnly:	False	
Attributes:	Variable Length; Updatable	
CollatingOrder:	General	
ColumnHidden:	False	
ColumnOrder:	Default	
ColumnWidth:	Default	
DataUpdatable:	True	
DisplayControl:	Text Box	
GUID:	{guid {8A93C24F-47CB-4D02-9D55-A8EC6CB8A9CC}}	
IMEMode:	0	
IMESentenceMode:	3	
OrdinalPosition:	11	
Required:	False	
SourceField:	Volume_Kurs	
SourceTable:	Medien	
TextAlign:	General	
UnicodeCompression:	True	

General store	Text	50
AggregateType:	-1	
AllowZeroLength:	True	
AppendOnly:	False	
Attributes:	Variable Length; Updatable	
CollatingOrder:	General	
ColumnHidden:	False	
ColumnOrder:	Default	
ColumnWidth:	Default	
DataUpdatable:	True	
DisplayControl:	Text Box	
GUID:	{guid {D1357FAA-8670-4AB9-9AB2-C3383EB364D3}}	
IMEMode:	0	
IMESentenceMode:	3	
OrdinalPosition:	12	
Required:	False	
SourceField:	General store	
SourceTable:	Medien	
TextAlign:	General	
UnicodeCompression:	True	

Medien ID	Long Integer	4

AggregateType:	-1
AllowZeroLength:	False
AppendOnly:	False
Attributes:	Fixed Size; Auto-Increment; Updatable
CollatingOrder:	General
ColumnHidden:	False
ColumnOrder:	1
ColumnWidth:	Default
DataUpdatable:	True
GUID:	{guid {FBE4AC72-E216-4CE4-ACB3-246BABE85369}}
OrdinalPosition:	13
Required:	False
SourceField:	Medien ID
SourceTable:	Medien
TextAlign:	General

Supplier Long Integer 4

AggregateType:	-1
AllowValueListEdits:	True
AllowZeroLength:	False
AppendOnly:	False
Attributes:	Fixed Size; Updatable
BoundColumn:	1
CollatingOrder:	General
ColumnCount:	2
ColumnHeads:	False
ColumnHidden:	False
ColumnOrder:	Default
ColumnWidth:	Default
ColumnWidths:	0;1440
DataUpdatable:	True
DecimalPlaces:	Auto
DisplayControl:	Combo Box
LimitToList:	True
ListRows:	16
ListWidth:	1440twip
OrdinalPosition:	14
Required:	False
RowSource:	SELECT [Lieferanten].[Lieferanten_id], [Lieferanten].[Lieferant] FROM [Lieferanten] ORDER BY [Lieferant] DESC;
RowSourceType:	Table/Query
ShowOnlyRowSourceValues:	False
SourceField:	Supplier
SourceTable:	Medien
TextAlign:	General

Table Indexes

Name	Number of Fields
Medien ID	1

Clustered:	False
DistinctCount:	36
Foreign:	False
IgnoreNulls:	False
Name:	Medien ID

	Primary:	False
	Required:	False
	Unique:	False
	Fields:	
	Medien ID	Ascending
Medien ID old		1
	Clustered:	False
	DistinctCount:	11
	Foreign:	False
	IgnoreNulls:	False
	Name:	Medien ID old
	Primary:	False
	Required:	False
	Unique:	False
	Fields:	
	Medien ID old	Ascending
Medienname		1
	Clustered:	False
	DistinctCount:	36
	Foreign:	False
	IgnoreNulls:	False
	Name:	Medienname
	Primary:	False
	Required:	False
	Unique:	True
	Fields:	
	Medienname	Ascending
PrimaryKey		1
	Clustered:	False
	DistinctCount:	36
	Foreign:	False
	IgnoreNulls:	False
	Name:	PrimaryKey
	Primary:	True
	Required:	True
	Unique:	True
	Fields:	
	Medien ID	Ascending

User Permissions

admin	Delete; Read Permissions; Set Permissions; Change Owner, Read Definition; Write Definition; Read Data; Insert Data; Update Data; Delete Data

Group Permissions

Admins	Delete; Read Permissions; Set Permissions; Change Owner, Read Definition; Write Definition; Read Data; Insert Data; Update Data; Delete Data
Users	Delete; Read Permissions; Set Permissions; Change Owner, Read Definition; Write Definition; Read Data; Insert Data; Update Data; Delete Data

Properties

DateCreated:	16.10.2009 11:44:47	DefaultView:	2
DisplayViewsOnSharePointSit	1	FilterOnLoad:	False
GUID:	{guid {CC08B390-D7A7-4555-8529-49E54C387580}}	HideNewField:	False
LastUpdated:	16.10.2009 12:50:54	MaxRecords:	0
NameMap:	Long binary data	ODBCTimeout:	60
OrderByOn:	False	OrderByOnLoad:	True
Orientation:	Left-to-Right	RecordLocks:	No Locks
RecordsAffected:	0	RecordsetType:	Dynaset
ReturnsRecords:	True	TotalsRow:	False
Type:	0	Updatable:	True

SQL

```
SELECT Medien.Protokoll, Medien.Medienname, Medien.Medienname_D, Medien.Bezeichner, Medien.Remark,
Medien.Solvent, Medien.Steril, Medien.Storagecondition, Medien.Storagesite, Medien.Kurs, Medien.Prepare,
Medien.Volume_Kurs, Medien.[General store], Medien.[Medien ID], Medien.Supplier
FROM Medien
WHERE (((Medien.[Medien ID])=[Forms]![Media_E_2]![Medien ID]));
```

Query Parameters

Name	Type
[Forms]![Media_E_2]![Medien ID]	Text

Columns

Name	Type	Size
Protokoll	Long Integer	4

AggregateType:	-1
AllowZeroLength:	False
AppendOnly:	False
Attributes:	Fixed Size; Updatable
CollatingOrder:	General
ColumnHidden:	False
ColumnOrder:	Default
ColumnWidth:	Default
DataUpdatable:	True
DecimalPlaces:	Auto
DisplayControl:	Text Box
GUID:	{guid {24E1DD39-D007-443F-ACDA-217642A9BE4F}}
OrdinalPosition:	0
Required:	False
SourceField:	Protokoll
SourceTable:	Medien
TextAlign:	General

Name	Type	Size
Medienname	Text	50

AggregateType:	-1
AllowZeroLength:	True

AppendOnly:	False	
Attributes:	Variable Length; Updatable	
CollatingOrder:	General	
ColumnHidden:	False	
ColumnOrder:	Default	
ColumnWidth:	3615	
DataUpdatable:	True	
DisplayControl:	Text Box	
GUID:	{guid {39A7EAC5-34A3-4BBD-A594-4E88A4399D39}}	
IMEMode:	0	
IMESentenceMode:	3	
OrdinalPosition:	1	
Required:	True	
SourceField:	Medienname	
SourceTable:	Medien	
TextAlign:	General	
UnicodeCompression:	True	

Medienname_D	Text	50
AggregateType:	-1	
AllowZeroLength:	True	
AppendOnly:	False	
Attributes:	Variable Length; Updatable	
CollatingOrder:	General	
ColumnHidden:	False	
ColumnOrder:	Default	
ColumnWidth:	2955	
DataUpdatable:	True	
DisplayControl:	Text Box	
GUID:	{guid {C9276239-CF36-4089-B820-A7A34A309B03}}	
IMEMode:	0	
IMESentenceMode:	3	
OrdinalPosition:	2	
Required:	False	
SourceField:	Medienname_D	
SourceTable:	Medien	
TextAlign:	General	
UnicodeCompression:	True	

Bezeichner	OLE Object	N/A
AggregateType:	-1	
AllowZeroLength:	False	
AppendOnly:	False	
Attributes:	Variable Length; Updatable	
CollatingOrder:	General	
ColumnHidden:	False	
ColumnOrder:	Default	
ColumnWidth:	1965	
DataUpdatable:	True	
GUID:	{guid {A52CDB4F-F76D-43DE-B348-217A2AD512CB}}	
OrdinalPosition:	3	
Required:	False	
SourceField:	Bezeichner	
SourceTable:	Medien	
TextAlign:	General	

Remark		Memo	N/A
	AggregateType:	-1	
	AllowZeroLength:	True	
	AppendOnly:	False	
	Attributes:	Variable Length; Updatable	
	CollatingOrder:	General	
	ColumnHidden:	False	
	ColumnOrder:	Default	
	ColumnWidth:	2745	
	DataUpdatable:	True	
	GUID:	{guid {F6E09F04-6090-435D-AAE4-5157DDB09E51}}	
	IMEMode:	0	
	IMESentenceMode:	3	
	OrdinalPosition:	4	
	Required:	False	
	SourceField:	Remark	
	SourceTable:	Medien	
	TextAlign:	General	
	TextFormat:	Plain Text	
	UnicodeCompression:	True	
Solvent		Text	50
	AggregateType:	-1	
	AllowZeroLength:	True	
	AppendOnly:	False	
	Attributes:	Variable Length; Updatable	
	CollatingOrder:	General	
	ColumnHidden:	False	
	ColumnOrder:	Default	
	ColumnWidth:	Default	
	DataUpdatable:	True	
	DisplayControl:	Text Box	
	GUID:	{guid {07F6C1A3-CFAD-4E65-819D-ABBA6FF7B366}}	
	IMEMode:	0	
	IMESentenceMode:	3	
	OrdinalPosition:	5	
	Required:	False	
	SourceField:	Solvent	
	SourceTable:	Medien	
	TextAlign:	General	
	UnicodeCompression:	True	
Steril		Text	50
	AggregateType:	-1	
	AllowZeroLength:	True	
	AppendOnly:	False	
	Attributes:	Variable Length; Updatable	
	CollatingOrder:	General	
	ColumnHidden:	False	
	ColumnOrder:	Default	
	ColumnWidth:	Default	
	DataUpdatable:	True	
	DisplayControl:	Text Box	
	GUID:	{guid {9C185040-3E47-44D4-B4A1-96698F823224}}	
	IMEMode:	0	
	IMESentenceMode:	3	

OrdinalPosition:	6	
Required:	False	
SourceField:	Steril	
SourceTable:	Medien	
TextAlign:	General	
UnicodeCompression:	True	

Storagecondition	Long Integer	4
AggregateType:	-1	
AllowValueListEdits:	False	
AllowZeroLength:	False	
AppendOnly:	False	
Attributes:	Fixed Size; Updatable	
BoundColumn:	1	
CollatingOrder:	General	
ColumnCount:	2	
ColumnHeads:	False	
ColumnHidden:	False	
ColumnOrder:	Default	
ColumnWidth:	2070	
ColumnWidths:	0;1440	
DataUpdatable:	True	
DecimalPlaces:	Auto	
DisplayControl:	Combo Box	
LimitToList:	True	
ListRows:	8	
ListWidth:	1440twip	
OrdinalPosition:	7	
Required:	False	
RowSource:	SELECT [Storagecondition].[Storagecondition_ID], [Storagecondition].[Storagecondition_Name] FROM [Storagecondition] ORDER BY [Storagecondition_Name];	
RowSourceType:	Table/Query	
ShowOnlyRowSourceValues:	False	
SourceField:	Storagecondition	
SourceTable:	Medien	
TextAlign:	General	

Storagesite	Long Integer	4
AggregateType:	-1	
AllowValueListEdits:	False	
AllowZeroLength:	False	
AppendOnly:	False	
Attributes:	Fixed Size; Updatable	
BoundColumn:	1	
CollatingOrder:	General	
ColumnCount:	2	
ColumnHeads:	False	
ColumnHidden:	False	
ColumnOrder:	Default	
ColumnWidth:	2925	
ColumnWidths:	0;1440	
DataUpdatable:	True	
DecimalPlaces:	Auto	

	DisplayControl:	Combo Box
	LimitToList:	True
	ListRows:	16
	ListWidth:	1440twip
	OrdinalPosition:	8
	Required:	False
	RowSource:	SELECT [Storagesite].[Storagesite_ID], [Storagesite].[Storagesite_Name] FROM [Storagesite] ORDER BY [Storagesite_Name];
	RowSourceType:	Table/Query
	ShowOnlyRowSourceValues:	False
	SourceField:	Storagesite
	SourceTable:	Medien
	TextAlign:	General

Kurs		Yes/No	1
	AggregateType:	-1	
	AllowZeroLength:	False	
	AppendOnly:	False	
	Attributes:	Fixed Size; Updatable	
	CollatingOrder:	General	
	ColumnHidden:	False	
	ColumnOrder:	Default	
	ColumnWidth:	Default	
	DataUpdatable:	True	
	DisplayControl:	106	
	Format:	Yes/No	
	GUID:	{guid {F9E180AA-9465-4BB3-9964-5F2BE293C4EA}}	
	OrdinalPosition:	9	
	Required:	False	
	SourceField:	Kurs	
	SourceTable:	Medien	
	TextAlign:	General	
Prepare		Text	50
	AggregateType:	-1	
	AllowMultipleValues:	False	
	AllowValueListEdits:	False	
	AllowZeroLength:	True	
	AppendOnly:	False	
	Attributes:	Variable Length; Updatable	
	BoundColumn:	1	
	CollatingOrder:	General	
	ColumnCount:	1	
	ColumnHeads:	False	
	ColumnHidden:	False	
	ColumnOrder:	Default	
	ColumnWidth:	Default	
	ColumnWidths:	1440	
	DataUpdatable:	True	
	DisplayControl:	Combo Box	
	GUID:	{guid {E2159DA9-862E-4E06-B5A8-EB44C2D01841}}	
	IMEMode:	0	
	IMESentenceMode:	3	

	LimitToList:	False	
	ListRows:	8	
	ListWidth:	1440twip	
	OrdinalPosition:	10	
	Required:	False	
	RowSource:	"Kurs";"Vorbereitung";"General stock"	
	RowSourceType:	Value List	
	ShowOnlyRowSourceValues:	False	
	SourceField:	Prepare	
	SourceTable:	Medien	
	TextAlign:	General	
	UnicodeCompression:	True	
Volume_Kurs		Text	50
	AggregateType:	-1	
	AllowZeroLength:	True	
	AppendOnly:	False	
	Attributes:	Variable Length; Updatable	
	CollatingOrder:	General	
	ColumnHidden:	False	
	ColumnOrder:	Default	
	ColumnWidth:	Default	
	DataUpdatable:	True	
	DisplayControl:	Text Box	
	GUID:	{guid {8A93C24F-47CB-4D02-9D55-A8EC6CB8A9CC}}	
	IMEMode:	0	
	IMESentenceMode:	3	
	OrdinalPosition:	11	
	Required:	False	
	SourceField:	Volume_Kurs	
	SourceTable:	Medien	
	TextAlign:	General	
	UnicodeCompression:	True	
General store		Text	50
	AggregateType:	-1	
	AllowZeroLength:	True	
	AppendOnly:	False	
	Attributes:	Variable Length; Updatable	
	CollatingOrder:	General	
	ColumnHidden:	False	
	ColumnOrder:	Default	
	ColumnWidth:	Default	
	DataUpdatable:	True	
	DisplayControl:	Text Box	
	GUID:	{guid {D1357FAA-8670-4AB9-9AB2-C3383EB364D3}}	
	IMEMode:	0	
	IMESentenceMode:	3	
	OrdinalPosition:	12	
	Required:	False	
	SourceField:	General store	
	SourceTable:	Medien	
	TextAlign:	General	
	UnicodeCompression:	True	
Medien ID		Long Integer	4

AggregateType:	-1
AllowZeroLength:	False
AppendOnly:	False
Attributes:	Fixed Size; Auto-Increment; Updatable
CollatingOrder:	General
ColumnHidden:	False
ColumnOrder:	1
ColumnWidth:	Default
DataUpdatable:	True
GUID:	{guid {FBE4AC72-E216-4CE4-ACB3-246BABE85369}}
OrdinalPosition:	13
Required:	False
SourceField:	Medien ID
SourceTable:	Medien
TextAlign:	General

Supplier Long Integer 4

AggregateType:	-1
AllowValueListEdits:	True
AllowZeroLength:	False
AppendOnly:	False
Attributes:	Fixed Size; Updatable
BoundColumn:	1
CollatingOrder:	General
ColumnCount:	2
ColumnHeads:	False
ColumnHidden:	False
ColumnOrder:	Default
ColumnWidth:	Default
ColumnWidths:	0;1440
DataUpdatable:	True
DecimalPlaces:	Auto
DisplayControl:	Combo Box
LimitToList:	True
ListRows:	16
ListWidth:	1440twip
OrdinalPosition:	14
Required:	False
RowSource:	SELECT [Lieferanten].[Lieferanten_id], [Lieferanten].[Lieferant] FROM [Lieferanten] ORDER BY [Lieferant] DESC;
RowSourceType:	Table/Query
ShowOnlyRowSourceValues:	False
SourceField:	Supplier
SourceTable:	Medien
TextAlign:	General

Table Indexes

Name	Number of Fields
Medien ID	1

Clustered:	False
DistinctCount:	36
Foreign:	False
IgnoreNulls:	False
Name:	Medien ID

	Primary:	False
	Required:	False
	Unique:	False
	Fields:	
	Medien ID	Ascending
Medien ID old		1
	Clustered:	False
	DistinctCount:	11
	Foreign:	False
	IgnoreNulls:	False
	Name:	Medien ID old
	Primary:	False
	Required:	False
	Unique:	False
	Fields:	
	Medien ID old	Ascending
Medienname		1
	Clustered:	False
	DistinctCount:	36
	Foreign:	False
	IgnoreNulls:	False
	Name:	Medienname
	Primary:	False
	Required:	False
	Unique:	True
	Fields:	
	Medienname	Ascending
PrimaryKey		1
	Clustered:	False
	DistinctCount:	36
	Foreign:	False
	IgnoreNulls:	False
	Name:	PrimaryKey
	Primary:	True
	Required:	True
	Unique:	True
	Fields:	
	Medien ID	Ascending

User Permissions

admin	Delete; Read Permissions; Set Permissions; Change Owner, Read Definition; Write Definition; Read Data; Insert Data; Update Data; Delete Data

Group Permissions

Admins	Delete; Read Permissions; Set Permissions; Change Owner, Read Definition; Write Definition; Read Data; Insert Data; Update Data; Delete Data
Users	Delete; Read Permissions; Set Permissions; Change Owner, Read Definition; Write Definition; Read Data; Insert Data; Update Data; Delete Data

Properties

DateCreated:	06.08.2009 08:55:27	DefaultView:	2
FilterOnLoad:	False	GUID:	{guid {5C6C4C18-5D56-4B4A-8D39-696E860601CF}}
LastUpdated:	16.10.2009 11:55:01	MaxRecords:	0
ODBCTimeout:	60	OrderByOn:	False
OrderByOnLoad:	True	Orientation:	Left-to-Right
RecordLocks:	Edited Record	RecordsAffected:	0
ReturnsRecords:	True	Type:	64
Updatable:	True	UseTransaction:	True

SQL

```
INSERT INTO Medienzusammensetzung ( Medien, Stock, Menge, Mengeneinheit, Protokollname, [Protokoll ID]
)
SELECT CopyMedien_NewMedienID_1.[Medien ID], CopyMedienzusammensetzung_select_1.Stock,
CopyMedienzusammensetzung_select_1.Menge, CopyMedienzusammensetzung_select_1.Mengeneinheit,
CopyMedienzusammensetzung_select_1.Protokollname, CopyMedienzusammensetzung_select_1.[Protokoll ID]
FROM CopyMedienzusammensetzung_select_1, CopyMedien_NewMedienID_1;
```

Query Parameters

Name	Type
[Forms]![Media_E_1]![Medien ID]	Text

User Permissions

admin	Delete; Read Permissions; Set Permissions; Change Owner, Read Definition; Write Definition; Read Data; Insert Data; Update Data; Delete Data

Group Permissions

Admins	Delete; Read Permissions; Set Permissions; Change Owner, Read Definition; Write Definition; Read Data; Insert Data; Update Data; Delete Data
Users	Delete; Read Permissions; Set Permissions; Change Owner, Read Definition; Write Definition; Read Data; Insert Data; Update Data; Delete Data

Properties

DateCreated:	16.10.2009 11:53:23	DefaultView:	2
FilterOnLoad:	False	GUID:	{guid {A7F2A2CD-F8AA-42F6-A33D-ED72CC824834}}
LastUpdated:	16.10.2009 11:56:30	MaxRecords:	0
ODBCTimeout:	60	OrderByOn:	False
OrderByOnLoad:	True	Orientation:	Left-to-Right
RecordLocks:	Edited Record	RecordsAffected:	0
ReturnsRecords:	True	Type:	64
Updatable:	True	UseTransaction:	True

SQL

```
INSERT INTO Medienzusammensetzung ( Medien, Stock, Menge, Mengeneinheit, Protokollname, [Protokoll ID] )
SELECT CopyMedien_NewMedienID_2.[Medien ID], CopyMedienzusammensetzung_select_2.Stock,
CopyMedienzusammensetzung_select_2.Menge, CopyMedienzusammensetzung_select_2.Mengeneinheit,
CopyMedienzusammensetzung_select_2.Protokollname, CopyMedienzusammensetzung_select_2.[Protokoll ID]
FROM CopyMedienzusammensetzung_select_2, CopyMedien_NewMedienID_2;
```

Query Parameters

Name	Type
[Forms]![Media_E_2]![Medien ID]	Text

User Permissions

admin	Delete; Read Permissions; Set Permissions; Change Owner, Read Definition; Write Definition; Read Data; Insert Data; Update Data; Delete Data

Group Permissions

Admins	Delete; Read Permissions; Set Permissions; Change Owner, Read Definition; Write Definition; Read Data; Insert Data; Update Data; Delete Data
Users	Delete; Read Permissions; Set Permissions; Change Owner, Read Definition; Write Definition; Read Data; Insert Data; Update Data; Delete Data

Properties

DateCreated:	06.08.2009 08:55:27	DefaultView:	2
DisplayViewsOnSharePointSit	1	FilterOnLoad:	False
GUID:	{guid {098CC7E4-96F6-489C-9819-AB9C7C88CE98}}	HideNewField:	False
LastUpdated:	16.10.2009 11:56:52	MaxRecords:	0
NameMap:	Long binary data	ODBCTimeout:	60
OrderByOn:	False	OrderByOnLoad:	True
Orientation:	Left-to-Right	RecordLocks:	No Locks
RecordsAffected:	0	RecordsetType:	Dynaset
ReturnsRecords:	True	TotalsRow:	False
Type:	0	Updatable:	True

SQL

```
SELECT Medienzusammensetzung.Medien, Medienzusammensetzung.Stock, Medienzusammensetzung.Menge,
Medienzusammensetzung.Mengeneinheit, Medienzusammensetzung.Protokollname,
Medienzusammensetzung.[Protokoll ID]
FROM Medienzusammensetzung
WHERE (((Medienzusammensetzung.Medien)=[Forms]![Media_E_1]![Medien ID]));
```

Query Parameters

Name	Type
[Forms]![Media_E_1]![Medien ID]	Text

Columns

Name	Type	Size
Medien	Long Integer	4

AggregateType:	-1
AllowValueListEdits:	False
AllowZeroLength:	False
AppendOnly:	False
Attributes:	Fixed Size; Updatable
BoundColumn:	1
CollatingOrder:	General
ColumnCount:	2
ColumnHeads:	False
ColumnHidden:	False
ColumnOrder:	Default
ColumnWidth:	3660
ColumnWidths:	0;3258
DataUpdatable:	True
DecimalPlaces:	Auto
DisplayControl:	Combo Box
GUID:	{guid {568B9D25-BA8B-4774-B214-F36020A2E255}}
LimitToList:	True
ListRows:	8
ListWidth:	3255twip
OrdinalPosition:	0

	Required:	False	
	RowSource:	SELECT Medien.[Medien ID] FROM Medien;	
	RowSourceType:	Table/Query	
	ShowOnlyRowSourceValues:	False	
	SourceField:	Medien	
	SourceTable:	Medienzusammensetzung	
	TextAlign:	General	
Stock		Long Integer	4
	AggregateType:	-1	
	AllowValueListEdits:	False	
	AllowZeroLength:	False	
	AppendOnly:	False	
	Attributes:	Fixed Size; Updatable	
	BoundColumn:	1	
	CollatingOrder:	General	
	ColumnCount:	2	
	ColumnHeads:	False	
	ColumnHidden:	False	
	ColumnOrder:	Default	
	ColumnWidth:	2580	
	ColumnWidths:	0;2730	
	DataUpdatable:	True	
	DecimalPlaces:	Auto	
	DisplayControl:	Combo Box	
	GUID:	{guid {A8A0F210-4ECF-44E0-9B28-8C90ACC20154}}	
	LimitToList:	True	
	ListRows:	8	
	ListWidth:	2730twip	
	OrdinalPosition:	1	
	Required:	False	
	RowSource:	SELECT Stocks.Stock_id, Stocks.Stock_name FROM Stocks;	
	RowSourceType:	Table/Query	
	ShowOnlyRowSourceValues:	False	
	SourceField:	Stock	
	SourceTable:	Medienzusammensetzung	
	TextAlign:	General	
Menge		Double	8
	AggregateType:	-1	
	AllowZeroLength:	False	
	AppendOnly:	False	
	Attributes:	Fixed Size; Updatable	
	CollatingOrder:	General	
	ColumnHidden:	False	
	ColumnOrder:	Default	
	ColumnWidth:	1140	
	DataUpdatable:	True	
	DecimalPlaces:	Auto	
	DisplayControl:	Text Box	
	GUID:	{guid {C238FB2E-3901-4526-9015-8653957C8B6E}}	
	OrdinalPosition:	2	
	Required:	False	
	SourceField:	Menge	
	SourceTable:	Medienzusammensetzung	
	TextAlign:	General	

Mengeneinheit	Text	50
AggregateType:	-1	
AllowValueListEdits:	False	
AllowZeroLength:	True	
AppendOnly:	False	
Attributes:	Variable Length; Updatable	
BoundColumn:	1	
CollatingOrder:	General	
ColumnCount:	1	
ColumnHeads:	False	
ColumnHidden:	False	
ColumnOrder:	Default	
ColumnWidth:	Default	
ColumnWidths:	1440	
DataUpdatable:	True	
DisplayControl:	Combo Box	
GUID:	{guid {DB67B6DD-8A3A-4595-9033-E79B08EA7937}}	
IMEMode:	0	
IMESentenceMode:	3	
LimitToList:	False	
ListRows:	8	
ListWidth:	1440twip	
OrdinalPosition:	3	
Required:	False	
RowSource:	"ml/l";"ul/l";"g/l";"mg/, ";"ul/900 ml";"g/900ml"	
RowSourceType:	Value List	
ShowOnlyRowSourceValues:	False	
SourceField:	Mengeneinheit	
SourceTable:	Medienzusammensetzung	
TextAlign:	General	
UnicodeCompression:	True	
Protokollname	Text	50
AggregateType:	-1	
AllowZeroLength:	True	
AppendOnly:	False	
Attributes:	Variable Length; Updatable	
CollatingOrder:	General	
ColumnHidden:	False	
ColumnOrder:	Default	
ColumnWidth:	3090	
DataUpdatable:	True	
DisplayControl:	Text Box	
GUID:	{guid {52E3AF2F-012F-416E-97E9-2AE3605BA211}}	
IMEMode:	0	
IMESentenceMode:	3	
OrdinalPosition:	4	
Required:	False	
SourceField:	Protokollname	
SourceTable:	Medienzusammensetzung	
TextAlign:	General	
UnicodeCompression:	True	
Protokoll ID	Long Integer	4
AggregateType:	-1	
AllowZeroLength:	False	

AppendOnly:	False
Attributes:	Fixed Size; Updatable
CollatingOrder:	General
ColumnHidden:	False
ColumnOrder:	Default
ColumnWidth:	Default
DataUpdatable:	True
DecimalPlaces:	Auto
DisplayControl:	Text Box
GUID:	{guid {39341760-6CC8-477B-A095-BFEAA231AB5B}}
OrdinalPosition:	5
Required:	False
SourceField:	Protokoll ID
SourceTable:	Medienzusammensetzung
TextAlign:	General

Table Indexes

Name		Number of Fields
Medien ID		1
	Clustered:	False
	DistinctCount:	54
	Foreign:	False
	IgnoreNulls:	False
	Name:	Medien ID
	Primary:	False
	Required:	False
	Unique:	False
	Fields:	
	Medien_Zusammen_ ID	Ascending
MedienMedienzusammensetzung		1
	Clustered:	False
	DistinctCount:	21
	Foreign:	True
	IgnoreNulls:	False
	Name:	MedienMedienzusammensetzung
	Primary:	False
	Required:	False
	Unique:	False
	Fields:	
	Medien	Ascending
PrimaryKey		1
	Clustered:	False
	DistinctCount:	54
	Foreign:	False
	IgnoreNulls:	False
	Name:	PrimaryKey
	Primary:	True
	Required:	True
	Unique:	True
	Fields:	
	Medien_Zusammen_ ID	Ascending

User Permissions

admin	Delete; Read Permissions; Set Permissions; Change Owner, Read Definition; Write Definition; Read Data; Insert Data; Update Data; Delete Data

Group Permissions

Admins	Delete; Read Permissions; Set Permissions; Change Owner, Read Definition; Write Definition; Read Data; Insert Data; Update Data; Delete Data
Users	Delete; Read Permissions; Set Permissions; Change Owner, Read Definition; Write Definition; Read Data; Insert Data; Update Data; Delete Data

Properties

DateCreated:	16.10.2009 11:53:31	DefaultView:	2
DisplayViewsOnSharePointSit	1	FilterOnLoad:	False
GUID:	{guid {E8B5F88C-1724-46DD-9514-ACE73EDBE40B}}	HideNewField:	False
LastUpdated:	16.10.2009 12:52:47	MaxRecords:	0
NameMap:	Long binary data	ODBCTimeout:	60
OrderByOn:	False	OrderByOnLoad:	True
Orientation:	Left-to-Right	RecordLocks:	No Locks
RecordsAffected:	0	RecordsetType:	Dynaset
ReturnsRecords:	True	TotalsRow:	False
Type:	0	Updatable:	True

SQL

```
SELECT Medienzusammensetzung.Medien, Medienzusammensetzung.Stock, Medienzusammensetzung.Menge,
Medienzusammensetzung.Mengeneinheit, Medienzusammensetzung.Protokollname,
Medienzusammensetzung.[Protokoll ID]
FROM Medienzusammensetzung
WHERE (((Medienzusammensetzung.Medien)=[Forms]![Media_E_2]![Medien ID]));
```

Query Parameters

Name	Type
[Forms]![Media_E_2]![Medien ID]	Text

Columns

Name	Type	Size
Medien	Long Integer	4

AggregateType:	-1
AllowValueListEdits:	False
AllowZeroLength:	False
AppendOnly:	False
Attributes:	Fixed Size; Updatable
BoundColumn:	1
CollatingOrder:	General
ColumnCount:	2
ColumnHeads:	False
ColumnHidden:	False
ColumnOrder:	Default
ColumnWidth:	3660
ColumnWidths:	0;3258
DataUpdatable:	True
DecimalPlaces:	Auto
DisplayControl:	Combo Box
GUID:	{guid {568B9D25-BA8B-4774-B214-F36020A2E255}}
LimitToList:	True
ListRows:	8
ListWidth:	3255twip
OrdinalPosition:	0

Required:	False
RowSource:	SELECT Medien.[Medien ID] FROM Medien;
RowSourceType:	Table/Query
ShowOnlyRowSourceValues:	False
SourceField:	Medien
SourceTable:	Medienzusammensetzung
TextAlign:	General

Stock Long Integer 4

AggregateType:	-1
AllowValueListEdits:	False
AllowZeroLength:	False
AppendOnly:	False
Attributes:	Fixed Size; Updatable
BoundColumn:	1
CollatingOrder:	General
ColumnCount:	2
ColumnHeads:	False
ColumnHidden:	False
ColumnOrder:	Default
ColumnWidth:	2580
ColumnWidths:	0;2730
DataUpdatable:	True
DecimalPlaces:	Auto
DisplayControl:	Combo Box
GUID:	{guid {A8A0F210-4ECF-44E0-9B28-8C90ACC20154}}
LimitToList:	True
ListRows:	8
ListWidth:	2730twip
OrdinalPosition:	1
Required:	False
RowSource:	SELECT Stocks.Stock_id, Stocks.Stock_name FROM Stocks;
RowSourceType:	Table/Query
ShowOnlyRowSourceValues:	False
SourceField:	Stock
SourceTable:	Medienzusammensetzung
TextAlign:	General

Menge Double 8

AggregateType:	-1
AllowZeroLength:	False
AppendOnly:	False
Attributes:	Fixed Size; Updatable
CollatingOrder:	General
ColumnHidden:	False
ColumnOrder:	Default
ColumnWidth:	1140
DataUpdatable:	True
DecimalPlaces:	Auto
DisplayControl:	Text Box
GUID:	{guid {C238FB2E-3901-4526-9015-8653957C8B6E}}
OrdinalPosition:	2
Required:	False
SourceField:	Menge
SourceTable:	Medienzusammensetzung
TextAlign:	General

Mengeneinheit	Text	50
AggregateType:	-1	
AllowValueListEdits:	False	
AllowZeroLength:	True	
AppendOnly:	False	
Attributes:	Variable Length; Updatable	
BoundColumn:	1	
CollatingOrder:	General	
ColumnCount:	1	
ColumnHeads:	False	
ColumnHidden:	False	
ColumnOrder:	Default	
ColumnWidth:	Default	
ColumnWidths:	1440	
DataUpdatable:	True	
DisplayControl:	Combo Box	
GUID:	{guid {DB67B6DD-8A3A-4595-9033-E79B08EA7937}}	
IMEMode:	0	
IMESentenceMode:	3	
LimitToList:	False	
ListRows:	8	
ListWidth:	1440twip	
OrdinalPosition:	3	
Required:	False	
RowSource:	"ml/l";"ul/l";"g/l";"mg/, ";"ul/900 ml";"g/900ml"	
RowSourceType:	Value List	
ShowOnlyRowSourceValues:	False	
SourceField:	Mengeneinheit	
SourceTable:	Medienzusammensetzung	
TextAlign:	General	
UnicodeCompression:	True	
Protokollname	Text	50
AggregateType:	-1	
AllowZeroLength:	True	
AppendOnly:	False	
Attributes:	Variable Length; Updatable	
CollatingOrder:	General	
ColumnHidden:	False	
ColumnOrder:	Default	
ColumnWidth:	3090	
DataUpdatable:	True	
DisplayControl:	Text Box	
GUID:	{guid {52E3AF2F-012F-416E-97E9-2AE3605BA211}}	
IMEMode:	0	
IMESentenceMode:	3	
OrdinalPosition:	4	
Required:	False	
SourceField:	Protokollname	
SourceTable:	Medienzusammensetzung	
TextAlign:	General	
UnicodeCompression:	True	
Protokoll ID	Long Integer	4
AggregateType:	-1	
AllowZeroLength:	False	

AppendOnly:	False
Attributes:	Fixed Size; Updatable
CollatingOrder:	General
ColumnHidden:	False
ColumnOrder:	Default
ColumnWidth:	Default
DataUpdatable:	True
DecimalPlaces:	Auto
DisplayControl:	Text Box
GUID:	{guid {39341760-6CC8-477B-A095-BFEAA231AB5B}}
OrdinalPosition:	5
Required:	False
SourceField:	Protokoll ID
SourceTable:	Medienzusammensetzung
TextAlign:	General

Table Indexes

Name		Number of Fields
Medien ID		1
	Clustered:	False
	DistinctCount:	54
	Foreign:	False
	IgnoreNulls:	False
	Name:	Medien ID
	Primary:	False
	Required:	False
	Unique:	False
	Fields:	
	Medien_Zusammen_ ID	Ascending
MedienMedienzusammensetzung		1
	Clustered:	False
	DistinctCount:	21
	Foreign:	True
	IgnoreNulls:	False
	Name:	MedienMedienzusammensetzung
	Primary:	False
	Required:	False
	Unique:	False
	Fields:	
	Medien	Ascending
PrimaryKey		1
	Clustered:	False
	DistinctCount:	54
	Foreign:	False
	IgnoreNulls:	False
	Name:	PrimaryKey
	Primary:	True
	Required:	True
	Unique:	True
	Fields:	
	Medien_Zusammen_ ID	Ascending

User Permissions

admin	Delete; Read Permissions; Set Permissions; Change Owner, Read Definition; Write Definition; Read Data; Insert Data; Update Data; Delete Data

Group Permissions

Admins	Delete; Read Permissions; Set Permissions; Change Owner, Read Definition; Write Definition; Read Data; Insert Data; Update Data; Delete Data
Users	Delete; Read Permissions; Set Permissions; Change Owner, Read Definition; Write Definition; Read Data; Insert Data; Update Data; Delete Data

Properties

DateCreated:	16.10.2009 10:53:33	DefaultView:	2
DOL:	Long binary data	FilterOnLoad:	False
GUID:	{guid {1BD44BBF-0E75-40A1-9BAC-3A2D678AA7B4}}	LastUpdated:	16.10.2009 11:13:15
MaxRecords:	0	ODBCTimeout:	60
OrderByOn:	False	OrderByOnLoad:	True
Orientation:	Left-to-Right	RecordLocks:	Edited Record
RecordsAffected:	0	ReturnsRecords:	True
Type:	64	Updatable:	True
UseTransaction:	True		

SQL

```
INSERT INTO Protokoll ( Protokollname, Selektionsmarker, Selektionskonzentration, Art, Genom, Details )
SELECT [Protokollname]+'_new' AS Expr1, CopyProtokoll_select_1.Selektionsmarker,
CopyProtokoll_select_1.Selektionskonzentration, CopyProtokoll_select_1.Protokoll.Art,
CopyProtokoll_select_1.Genom, CopyProtokoll_select_1.Details
FROM CopyProtokoll_select_1;
```

Query Parameters

Name	Type
[Forms]![Method form E_1]![Protokoll ID]	Text

User Permissions

admin	Delete; Read Permissions; Set Permissions; Change Owner, Read Definition; Write Definition; Read Data; Insert Data; Update Data; Delete Data

Group Permissions

Admins	Delete; Read Permissions; Set Permissions; Change Owner, Read Definition; Write Definition; Read Data; Insert Data; Update Data; Delete Data
Users	Delete; Read Permissions; Set Permissions; Change Owner, Read Definition; Write Definition; Read Data; Insert Data; Update Data; Delete Data

Properties

DateCreated:	15.05.2009 14:08:12	DefaultView:	2
DOL:	Long binary data	FilterOnLoad:	False
GUID:	{guid {EF1A5DF4-D065-48C4-BC6E-51C20C929DDE}}	LastUpdated:	16.10.2009 11:14:24
MaxRecords:	0	ODBCTimeout:	60
OrderByOn:	False	OrderByOnLoad:	True
Orientation:	Left-to-Right	RecordLocks:	Edited Record
RecordsAffected:	0	ReturnsRecords:	True
Type:	64	Updatable:	True
UseTransaction:	True		

SQL

```
INSERT INTO Protokoll ( Protokollname, Selektionsmarker, Selektionskonzentration, Art, Genom, Details )
SELECT [Protokollname]+'_new' AS Expr1, CopyProtokoll_select_2.Selektionsmarker,
CopyProtokoll_select_2.Selektionskonzentration, CopyProtokoll_select_2.Protokoll.Art,
CopyProtokoll_select_2.Genom, CopyProtokoll_select_2.Details
FROM CopyProtokoll_select_2;
```

Query Parameters

Name	Type
[Forms]![Method form E_2]![Protokoll ID]	Text

User Permissions

admin	Delete; Read Permissions; Set Permissions; Change Owner, Read Definition; Write Definition; Read Data; Insert Data; Update Data; Delete Data

Group Permissions

Admins	Delete; Read Permissions; Set Permissions; Change Owner, Read Definition; Write Definition; Read Data; Insert Data; Update Data; Delete Data
Users	Delete; Read Permissions; Set Permissions; Change Owner, Read Definition; Write Definition; Read Data; Insert Data; Update Data; Delete Data

Properties

DateCreated:	16.10.2009 10:53:33	DefaultView:	2
DisplayViewsOnSharePointSit	1	DOL:	Long binary data
FilterOnLoad:	False	GUID:	{guid {9407B43F-DB49-45E7-A886-D83211DE3654}}
HideNewField:	False	LastUpdated:	16.10.2009 10:55:11
MaxRecords:	0	NameMap:	Long binary data
ODBCTimeout:	60	OrderByOn:	False
OrderByOnLoad:	True	Orientation:	Left-to-Right
RecordLocks:	No Locks	RecordsAffected:	0
RecordsetType:	Dynaset	ReturnsRecords:	True
TotalsRow:	False	Type:	0
Updatable:	True		

SQL

```
SELECT Protokoll.Protokollname, Protokoll.Selektionsmarker, Protokoll.Selektionskonzentration, Protokoll.Art,
Protokoll.Details, Protokoll.Genom, Protokoll.[Protokoll ID]
FROM Protokoll
WHERE (((Protokoll.[Protokoll ID])=[Forms]![Method form E_1]![Protokoll ID]));
```

Query Parameters

Name	Type
[Forms]![Method form E_1]![Protokoll ID]	Text

Columns

Name	Type	Size
Protokollname	Text	50

AggregateType:	-1
AllowZeroLength:	True
AppendOnly:	False
Attributes:	Variable Length; Updatable
CollatingOrder:	General
ColumnHidden:	False
ColumnOrder:	Default
ColumnWidth:	4905
DataUpdatable:	True
Description:	Eindeutiger Name für das Protokoll
DisplayControl:	Text Box
GUID:	{guid {BA35B293-EC1A-433F-A9CA-F649675F83D2}}
IMEMode:	0
IMESentenceMode:	3
OrdinalPosition:	0
Required:	False
SourceField:	Protokollname
SourceTable:	Protokoll
TextAlign:	General
UnicodeCompression:	True

Selektionsmarker	Long Integer	4

AggregateType:	-1
AllowValueListEdits:	True
AllowZeroLength:	False
AppendOnly:	False
Attributes:	Fixed Size; Updatable
BoundColumn:	1
CollatingOrder:	General
ColumnCount:	2
ColumnHeads:	False
ColumnHidden:	False
ColumnOrder:	Default
ColumnWidth:	Default
ColumnWidths:	0;1440
DataUpdatable:	True
DecimalPlaces:	Auto
Description:	Selektionsmarker, der im Protokoll verwendet wird
DisplayControl:	Combo Box
GUID:	{guid {E71F0FE8-862F-425C-A419-6A2B4C864997}}
LimitToList:	True
ListRows:	16
ListWidth:	1440twip
OrdinalPosition:	1
Required:	False
RowSource:	SELECT [Resistenz Pflanze].[ID], [Resistenz Pflanze].[Resistenz] FROM [Resistenz Pflanze] ORDER BY [ID];
RowSourceType:	Table/Query
ShowOnlyRowSourceValues:	False
SourceField:	Selektionsmarker
SourceTable:	Protokoll
TextAlign:	General

Selektionskonzentration	Long Integer	4

AggregateType:	-1
AllowZeroLength:	False
AppendOnly:	False
Attributes:	Fixed Size; Updatable
CollatingOrder:	General
ColumnHidden:	False
ColumnOrder:	Default
ColumnWidth:	2190
DataUpdatable:	True
DecimalPlaces:	Auto
DisplayControl:	Text Box
GUID:	{guid {5C6536FC-148D-4E2B-8FDA-82B5D77D41D0}}
OrdinalPosition:	2
Required:	False
SourceField:	Selektionskonzentration
SourceTable:	Protokoll
TextAlign:	General

Art	Long Integer	4

AggregateType:	-1
AllowValueListEdits:	False
AllowZeroLength:	False

	AppendOnly:	False
	Attributes:	Fixed Size; Updatable
	BoundColumn:	1
	CollatingOrder:	General
	ColumnCount:	2
	ColumnHeads:	False
	ColumnHidden:	False
	ColumnOrder:	Default
	ColumnWidth:	2205
	ColumnWidths:	0;2610
	DataUpdatable:	True
	DecimalPlaces:	Auto
	Description:	Art, die transformiert wird
	DisplayControl:	Combo Box
	GUID:	{guid {95DD1FAB-F52C-4A5D-9D7C-1BA59FA1F350}}
	LimitToList:	True
	ListRows:	8
	ListWidth:	2610twip
	OrdinalPosition:	3
	Required:	False
	RowSource:	SELECT Pflanzenarten.ID, Pflanzenarten.Species FROM Pflanzenarten;
	RowSourceType:	Table/Query
	ShowOnlyRowSourceValues:	False
	SourceField:	Art
	SourceTable:	Protokoll
	TextAlign:	General
Details		Memo N/A
	AggregateType:	-1
	AllowZeroLength:	True
	AppendOnly:	False
	Attributes:	Variable Length; Updatable
	CollatingOrder:	General
	ColumnHidden:	False
	ColumnOrder:	Default
	ColumnWidth:	2760
	DataUpdatable:	True
	GUID:	{guid {40223548-3FAF-4211-9885-B44B965E6533}}
	IMEMode:	0
	IMESentenceMode:	3
	OrdinalPosition:	4
	Required:	False
	SourceField:	Details
	SourceTable:	Protokoll
	TextAlign:	General
	TextFormat:	Plain Text
	UnicodeCompression:	True
Genom		Text 50
	AggregateType:	-1
	AllowValueListEdits:	False
	AllowZeroLength:	True
	AppendOnly:	False
	Attributes:	Variable Length; Updatable
	BoundColumn:	1
	CollatingOrder:	General

ColumnCount:	1
ColumnHeads:	False
ColumnHidden:	False
ColumnOrder:	Default
ColumnWidth:	Default
ColumnWidths:	1440
DataUpdatable:	True
DisplayControl:	Combo Box
GUID:	{guid {B3D19F8E-9909-407C-8A31-4B6AB22D3C16}}
IMEMode:	0
IMESentenceMode:	3
LimitToList:	False
ListRows:	8
ListWidth:	1440twip
OrdinalPosition:	5
Required:	False
RowSource:	"Kern";"Chloroplast";"Mitochondrien"
RowSourceType:	Value List
ShowOnlyRowSourceValues:	False
SourceField:	Genom
SourceTable:	Protokoll
TextAlign:	General
UnicodeCompression:	True

Protokoll ID Long Integer 4

AggregateType:	-1
AllowZeroLength:	False
AppendOnly:	False
Attributes:	Fixed Size; Auto-Increment; Updatable
CollatingOrder:	General
ColumnHidden:	False
ColumnOrder:	1
ColumnWidth:	1507
DataUpdatable:	True
GUID:	{guid {8CB33A61-EAEE-4BB7-A2F0-71206DFBD2A9}}
OrdinalPosition:	6
Required:	False
SourceField:	Protokoll ID
SourceTable:	Protokoll
TextAlign:	General

Table Indexes

Name	Number of Fields
PrimaryKey	1

Clustered:	False
DistinctCount:	3
Foreign:	False
IgnoreNulls:	False
Name:	PrimaryKey
Primary:	True
Required:	True
Unique:	True

Fields:	
Protokoll ID	Ascending
ProtokollArt	1
Clustered:	False
DistinctCount:	1
Foreign:	False
IgnoreNulls:	False
Name:	ProtokollArt
Primary:	False
Required:	False
Unique:	False
Fields:	
Art	Ascending

User Permissions

admin	Delete; Read Permissions; Set Permissions; Change Owner, Read Definition; Write Definition; Read Data; Insert Data; Update Data; Delete Data

Group Permissions

Admins	Delete; Read Permissions; Set Permissions; Change Owner, Read Definition; Write Definition; Read Data; Insert Data; Update Data; Delete Data
Users	Delete; Read Permissions; Set Permissions; Change Owner, Read Definition; Write Definition; Read Data; Insert Data; Update Data; Delete Data

Properties

DateCreated:	15.05.2009 14:08:12	DefaultView:	2
DisplayViewsOnSharePointSit	1	DOL:	Long binary data
FilterOnLoad:	False	GUID:	{guid {399641A9-CC22-4AC6-B27B-853FB3C0C2F2}}
HideNewField:	False	LastUpdated:	16.10.2009 11:14:55
MaxRecords:	0	NameMap:	Long binary data
ODBCTimeout:	60	OrderByOn:	False
OrderByOnLoad:	True	Orientation:	Left-to-Right
RecordLocks:	No Locks	RecordsAffected:	0
RecordsetType:	Dynaset	ReturnsRecords:	True
TotalsRow:	False	Type:	0
Updatable:	True		

SQL

```
SELECT Protokoll.Protokollname, Protokoll.Selektionsmarker, Protokoll.Selektionskonzentration, Protokoll.Art,
Protokoll.Details, Protokoll.Genom, Protokoll.[Protokoll ID]
FROM Protokoll
WHERE (((Protokoll.[Protokoll ID])=[Forms]![Method form E_2]![Protokoll ID]));
```

Query Parameters

Name	Type
[Forms]![Method form E_2]![Protokoll ID]	Text

Columns

Name	Type	Size
Protokollname	Text	50

AggregateType:	-1
AllowZeroLength:	True
AppendOnly:	False
Attributes:	Variable Length; Updatable
CollatingOrder:	General
ColumnHidden:	False
ColumnOrder:	Default
ColumnWidth:	4905
DataUpdatable:	True
Description:	Eindeutiger Name für das Protokoll
DisplayControl:	Text Box
GUID:	{guid {BA35B293-EC1A-433F-A9CA-F649675F83D2}}
IMEMode:	0
IMESentenceMode:	3
OrdinalPosition:	0
Required:	False
SourceField:	Protokollname
SourceTable:	Protokoll
TextAlign:	General
UnicodeCompression:	True

Selektionsmarker	Long Integer	4

AggregateType:	-1
AllowValueListEdits:	True
AllowZeroLength:	False
AppendOnly:	False
Attributes:	Fixed Size; Updatable
BoundColumn:	1
CollatingOrder:	General
ColumnCount:	2
ColumnHeads:	False
ColumnHidden:	False
ColumnOrder:	Default
ColumnWidth:	Default
ColumnWidths:	0;1440
DataUpdatable:	True
DecimalPlaces:	Auto
Description:	Selektionsmarker, der im Protokoll verwendet wird
DisplayControl:	Combo Box
GUID:	{guid {E71F0FE8-862F-425C-A419-6A2B4C864997}}
LimitToList:	True
ListRows:	16
ListWidth:	1440twip
OrdinalPosition:	1
Required:	False
RowSource:	SELECT [Resistenz Pflanze].[ID], [Resistenz Pflanze].[Resistenz] FROM [Resistenz Pflanze] ORDER BY [ID];
RowSourceType:	Table/Query
ShowOnlyRowSourceValues:	False
SourceField:	Selektionsmarker
SourceTable:	Protokoll
TextAlign:	General

Selektionskonzentration	Long Integer	4

AggregateType:	-1
AllowZeroLength:	False
AppendOnly:	False
Attributes:	Fixed Size; Updatable
CollatingOrder:	General
ColumnHidden:	False
ColumnOrder:	Default
ColumnWidth:	2190
DataUpdatable:	True
DecimalPlaces:	Auto
DisplayControl:	Text Box
GUID:	{guid {5C6536FC-148D-4E2B-8FDA-82B5D77D41D0}}
OrdinalPosition:	2
Required:	False
SourceField:	Selektionskonzentration
SourceTable:	Protokoll
TextAlign:	General

Art	Long Integer	4

AggregateType:	-1
AllowValueListEdits:	False
AllowZeroLength:	False

	AppendOnly:	False
	Attributes:	Fixed Size; Updatable
	BoundColumn:	1
	CollatingOrder:	General
	ColumnCount:	2
	ColumnHeads:	False
	ColumnHidden:	False
	ColumnOrder:	Default
	ColumnWidth:	2205
	ColumnWidths:	0;2610
	DataUpdatable:	True
	DecimalPlaces:	Auto
	Description:	Art, die transformiert wird
	DisplayControl:	Combo Box
	GUID:	{guid {95DD1FAB-F52C-4A5D-9D7C-1BA59FA1F350}}
	LimitToList:	True
	ListRows:	8
	ListWidth:	2610twip
	OrdinalPosition:	3
	Required:	False
	RowSource:	SELECT Pflanzenarten.ID, Pflanzenarten.Species FROM Pflanzenarten;
	RowSourceType:	Table/Query
	ShowOnlyRowSourceValues:	False
	SourceField:	Art
	SourceTable:	Protokoll
	TextAlign:	General
Details		Memo N/A
	AggregateType:	-1
	AllowZeroLength:	True
	AppendOnly:	False
	Attributes:	Variable Length; Updatable
	CollatingOrder:	General
	ColumnHidden:	False
	ColumnOrder:	Default
	ColumnWidth:	2760
	DataUpdatable:	True
	GUID:	{guid {40223548-3FAF-4211-9885-B44B965E6533}}
	IMEMode:	0
	IMESentenceMode:	3
	OrdinalPosition:	4
	Required:	False
	SourceField:	Details
	SourceTable:	Protokoll
	TextAlign:	General
	TextFormat:	Plain Text
	UnicodeCompression:	True
Genom		Text 50
	AggregateType:	-1
	AllowValueListEdits:	False
	AllowZeroLength:	True
	AppendOnly:	False
	Attributes:	Variable Length; Updatable
	BoundColumn:	1
	CollatingOrder:	General

ColumnCount:	1
ColumnHeads:	False
ColumnHidden:	False
ColumnOrder:	Default
ColumnWidth:	Default
ColumnWidths:	1440
DataUpdatable:	True
DisplayControl:	Combo Box
GUID:	{guid {B3D19F8E-9909-407C-8A31-4B6AB22D3C16}}
IMEMode:	0
IMESentenceMode:	3
LimitToList:	False
ListRows:	8
ListWidth:	1440twip
OrdinalPosition:	5
Required:	False
RowSource:	"Kern";"Chloroplast";"Mitochondrien"
RowSourceType:	Value List
ShowOnlyRowSourceValues:	False
SourceField:	Genom
SourceTable:	Protokoll
TextAlign:	General
UnicodeCompression:	True

Protokoll ID Long Integer 4

AggregateType:	-1
AllowZeroLength:	False
AppendOnly:	False
Attributes:	Fixed Size; Auto-Increment; Updatable
CollatingOrder:	General
ColumnHidden:	False
ColumnOrder:	1
ColumnWidth:	1507
DataUpdatable:	True
GUID:	{guid {8CB33A61-EAEE-4BB7-A2F0-71206DFBD2A9}}
OrdinalPosition:	6
Required:	False
SourceField:	Protokoll ID
SourceTable:	Protokoll
TextAlign:	General

Table Indexes

Name	Number of Fields
PrimaryKey	1

Clustered:	False
DistinctCount:	3
Foreign:	False
IgnoreNulls:	False
Name:	PrimaryKey
Primary:	True
Required:	True
Unique:	True

Fields:	
Protokoll ID	Ascending
ProtokollArt	1
Clustered:	False
DistinctCount:	1
Foreign:	False
IgnoreNulls:	False
Name:	ProtokollArt
Primary:	False
Required:	False
Unique:	False
Fields:	
Art	Ascending

User Permissions

admin	Delete; Read Permissions; Set Permissions; Change Owner, Read Definition; Write Definition; Read Data; Insert Data; Update Data; Delete Data

Group Permissions

Admins	Delete; Read Permissions; Set Permissions; Change Owner, Read Definition; Write Definition; Read Data; Insert Data; Update Data; Delete Data
Users	Delete; Read Permissions; Set Permissions; Change Owner, Read Definition; Write Definition; Read Data; Insert Data; Update Data; Delete Data

Properties

DateCreated:	16.10.2009 10:53:34	DefaultView:	2
DOL:	Long binary data	FilterOnLoad:	False
GUID:	{guid {C1171BD1-14CB-4961-8264-97F7A3FBEFCD}}	LastUpdated:	16.10.2009 11:15:57
MaxRecords:	0	ODBCTimeout:	60
OrderByOn:	False	OrderByOnLoad:	True
Orientation:	Left-to-Right	RecordLocks:	No Locks
RecordsAffected:	0	RecordsetType:	Dynaset
ReturnsRecords:	True	TotalsRow:	False
Type:	0	Updatable:	True

SQL

```
SELECT Protokoll.[Protokoll ID]
FROM Protokoll, CopyProtokoll_Select_Protokollname_1
WHERE (((Protokoll.Protokollname)=[CopyProtokoll_Select_Protokollname_1.Protokollname]+'_new'));
```

Columns

Name	Type	Size
Protokoll ID	Long Integer	4

AggregateType:	-1
AllowZeroLength:	False
AppendOnly:	False
Attributes:	Fixed Size; Auto-Increment
CollatingOrder:	General
ColumnHidden:	False
ColumnOrder:	1
ColumnWidth:	1507
DataUpdatable:	False
GUID:	{guid {8CB33A61-EAEE-4BB7-A2F0-71206DFBD2A9}}
OrdinalPosition:	0
Required:	False
SourceField:	Protokoll ID
SourceTable:	Protokoll
TextAlign:	General

Table Indexes

Name	Number of Fields
PrimaryKey	1

Clustered:	False
DistinctCount:	3
Foreign:	False
IgnoreNulls:	False
Name:	PrimaryKey
Primary:	True
Required:	True
Unique:	True

	Fields:	
	Protokoll ID	Ascending
ProtokollArt		1
	Clustered:	False
	DistinctCount:	1
	Foreign:	False
	IgnoreNulls:	False
	Name:	ProtokollArt
	Primary:	False
	Required:	False
	Unique:	False
	Fields:	
	Art	Ascending

User Permissions

admin	Delete; Read Permissions; Set Permissions; Change Owner, Read Definition; Write Definition; Read Data; Insert Data; Update Data; Delete Data

Group Permissions

Admins	Delete; Read Permissions; Set Permissions; Change Owner, Read Definition; Write Definition; Read Data; Insert Data; Update Data; Delete Data
Users	Delete; Read Permissions; Set Permissions; Change Owner, Read Definition; Write Definition; Read Data; Insert Data; Update Data; Delete Data

Properties

DateCreated:	27.03.2009 12:41:21	DefaultView:	2
DOL:	Long binary data	FilterOnLoad:	False
GUID:	{guid {B40B7D11-DAE0-4EE0-B5F5-B74F5FD776AB}}	LastUpdated:	16.10.2009 11:16:17
MaxRecords:	0	ODBCTimeout:	60
OrderByOn:	False	OrderByOnLoad:	True
Orientation:	Left-to-Right	RecordLocks:	No Locks
RecordsAffected:	0	RecordsetType:	Dynaset
ReturnsRecords:	True	TotalsRow:	False
Type:	0	Updatable:	True

SQL

```
SELECT Protokoll.[Protokoll ID]
FROM Protokoll, CopyProtokoll_Select_Protokollname_2
WHERE (((Protokoll.Protokollname)=[CopyProtokoll_Select_Protokollname_2.Protokollname]+'_new'));
```

Columns

Name	Type	Size
Protokoll ID	Long Integer	4

AggregateType:	-1
AllowZeroLength:	False
AppendOnly:	False
Attributes:	Fixed Size; Auto-Increment
CollatingOrder:	General
ColumnHidden:	False
ColumnOrder:	1
ColumnWidth:	1507
DataUpdatable:	False
GUID:	{guid {8CB33A61-EAEE-4BB7-A2F0-71206DFBD2A9}}
OrdinalPosition:	0
Required:	False
SourceField:	Protokoll ID
SourceTable:	Protokoll
TextAlign:	General

Table Indexes

Name	Number of Fields
PrimaryKey	1

Clustered:	False
DistinctCount:	3
Foreign:	False
IgnoreNulls:	False
Name:	PrimaryKey
Primary:	True
Required:	True
Unique:	True

	Fields:	
	Protokoll ID	Ascending
ProtokollArt		1
	Clustered:	False
	DistinctCount:	1
	Foreign:	False
	IgnoreNulls:	False
	Name:	ProtokollArt
	Primary:	False
	Required:	False
	Unique:	False
	Fields:	
	Art	Ascending

User Permissions

admin	Delete; Read Permissions; Set Permissions; Change Owner, Read Definition; Write Definition; Read Data; Insert Data; Update Data; Delete Data

Group Permissions

Admins	Delete; Read Permissions; Set Permissions; Change Owner, Read Definition; Write Definition; Read Data; Insert Data; Update Data; Delete Data
Users	Delete; Read Permissions; Set Permissions; Change Owner, Read Definition; Write Definition; Read Data; Insert Data; Update Data; Delete Data

Properties

DateCreated:	16.10.2009 10:53:34	DefaultView:	2
DisplayViewsOnSharePointSit	1	DOL:	Long binary data
FilterOnLoad:	False	GUID:	{guid {9407B43F-DB49-45E7-A886-D83211DE3654}}
HideNewField:	False	LastUpdated:	16.10.2009 10:55:31
MaxRecords:	0	NameMap:	Long binary data
ODBCTimeout:	60	OrderByOn:	False
OrderByOnLoad:	True	Orientation:	Left-to-Right
RecordLocks:	No Locks	RecordsAffected:	0
RecordsetType:	Dynaset	ReturnsRecords:	True
TotalsRow:	False	Type:	0
Updatable:	True		

SQL

```
SELECT Protokoll.Protokollname
FROM Protokoll
WHERE (((Protokoll.[Protokoll ID])=[Protokoll ID]));
```

Columns

Name	Type	Size
Protokollname	Text	50

AggregateType:	-1
AllowZeroLength:	True
AppendOnly:	False
Attributes:	Variable Length; Updatable
CollatingOrder:	General
ColumnHidden:	False
ColumnOrder:	Default
ColumnWidth:	4905
DataUpdatable:	True
Description:	Eindeutiger Name für das Protokoll
DisplayControl:	Text Box
GUID:	{guid {BA35B293-EC1A-433F-A9CA-F649675F83D2}}
IMEMode:	0
IMESentenceMode:	3
OrdinalPosition:	0
Required:	False
SourceField:	Protokollname
SourceTable:	Protokoll
TextAlign:	General
UnicodeCompression:	True

Table Indexes

Name	Number of Fields
PrimaryKey	1

Clustered:	False
DistinctCount:	3
Foreign:	False

	IgnoreNulls:	False
	Name:	PrimaryKey
	Primary:	True
	Required:	True
	Unique:	True
	Fields:	
	Protokoll ID	Ascending
ProtokollArt		1
	Clustered:	False
	DistinctCount:	1
	Foreign:	False
	IgnoreNulls:	False
	Name:	ProtokollArt
	Primary:	False
	Required:	False
	Unique:	False
	Fields:	
	Art	Ascending

User Permissions

admin	Delete; Read Permissions; Set Permissions; Change Owner, Read Definition; Write Definition; Read Data; Insert Data; Update Data; Delete Data

Group Permissions

Admins	Delete; Read Permissions; Set Permissions; Change Owner, Read Definition; Write Definition; Read Data; Insert Data; Update Data; Delete Data
Users	Delete; Read Permissions; Set Permissions; Change Owner, Read Definition; Write Definition; Read Data; Insert Data; Update Data; Delete Data

Properties

DateCreated:	27.03.2009 12:41:21	DefaultView:	2
DisplayViewsOnSharePointSit	1	DOL:	Long binary data
FilterOnLoad:	False	GUID:	{guid {FF221FE9-829B-4F06-BEAF-4F279A12D706}}
HideNewField:	False	LastUpdated:	16.10.2009 10:58:08
MaxRecords:	0	NameMap:	Long binary data
ODBCTimeout:	60	OrderByOn:	False
OrderByOnLoad:	True	Orientation:	Left-to-Right
RecordLocks:	No Locks	RecordsAffected:	0
RecordsetType:	Dynaset	ReturnsRecords:	True
TotalsRow:	False	Type:	0
Updatable:	True		

SQL

```
SELECT Protokoll.Protokollname
FROM Protokoll
WHERE (((Protokoll.[Protokoll ID])=[Protokoll ID]));
```

Columns

Name	Type	Size
Protokollname	Text	50

AggregateType:	-1
AllowZeroLength:	True
AppendOnly:	False
Attributes:	Variable Length; Updatable
CollatingOrder:	General
ColumnHidden:	False
ColumnOrder:	Default
ColumnWidth:	4905
DataUpdatable:	True
Description:	Eindeutiger Name für das Protokoll
DisplayControl:	Text Box
GUID:	{guid {BA35B293-EC1A-433F-A9CA-F649675F83D2}}
IMEMode:	0
IMESentenceMode:	3
OrdinalPosition:	0
Required:	False
SourceField:	Protokollname
SourceTable:	Protokoll
TextAlign:	General
UnicodeCompression:	True

Table Indexes

Name	Number of Fields
PrimaryKey	1

Clustered:	False
DistinctCount:	3
Foreign:	False

IgnoreNulls:	False
Name:	PrimaryKey
Primary:	True
Required:	True
Unique:	True
Fields:	
Protokoll ID	Ascending

ProtokollArt 1

Clustered:	False
DistinctCount:	1
Foreign:	False
IgnoreNulls:	False
Name:	ProtokollArt
Primary:	False
Required:	False
Unique:	False
Fields:	
Art	Ascending

User Permissions

admin	Delete; Read Permissions; Set Permissions; Change Owner, Read Definition; Write Definition; Read Data; Insert Data; Update Data; Delete Data

Group Permissions

Admins	Delete; Read Permissions; Set Permissions; Change Owner, Read Definition; Write Definition; Read Data; Insert Data; Update Data; Delete Data
Users	Delete; Read Permissions; Set Permissions; Change Owner, Read Definition; Write Definition; Read Data; Insert Data; Update Data; Delete Data

Properties

DateCreated:	16.10.2009 10:53:35	DefaultView:	2
DOL:	Long binary data	FilterOnLoad:	False
GUID:	{guid {E12C147A-5625-4634-B3BE-3D487B32250B}}	LastUpdated:	16.10.2009 11:17:55
MaxRecords:	0	ODBCTimeout:	60
OrderByOn:	False	OrderByOnLoad:	True
Orientation:	Left-to-Right	RecordLocks:	Edited Record
RecordsAffected:	0	ReturnsRecords:	True
Type:	64	Updatable:	True
UseTransaction:	True		

SQL

```
INSERT INTO Transformationsschritte ( Art, Old_ID, Prozess, Schrittid, ZeitvonStart, Zeitvondavor,
Bemerkungen, Light, Temperature )
SELECT [Forms]![Method form E_1]![Protokoll ID], CopySingleTrafoStep_select_1.ID,
CopySingleTrafoStep_select_1.Prozess, CopySingleTrafoStep_select_1.Schrittid,
CopySingleTrafoStep_select_1.ZeitvonStart, CopySingleTrafoStep_select_1.Zeitvondavor,
CopySingleTrafoStep_select_1.Bemerkungen, CopySingleTrafoStep_select_1.Light,
CopySingleTrafoStep_select_1.Temperature
FROM CopySingleTrafoStep_select_1;
```

Query Parameters

Name	Type
[Forms]![Method form E_1]![SelectedTrafoStep]	Text
[Forms]![Method form E_1]![Protokoll ID]	Text

User Permissions

admin	Delete; Read Permissions; Set Permissions; Change Owner, Read Definition; Write Definition; Read Data; Insert Data; Update Data; Delete Data

Group Permissions

Admins	Delete; Read Permissions; Set Permissions; Change Owner, Read Definition; Write Definition; Read Data; Insert Data; Update Data; Delete Data
Users	Delete; Read Permissions; Set Permissions; Change Owner, Read Definition; Write Definition; Read Data; Insert Data; Update Data; Delete Data

Properties

DateCreated:	27.03.2009 12:41:21	DefaultView:	2
DOL:	Long binary data	FilterOnLoad:	False
GUID:	{guid {880DFBB6-8C8C-48DE-98C9-11CC7E9F2A82}}	LastUpdated:	16.10.2009 11:19:09
MaxRecords:	0	ODBCTimeout:	60
OrderByOn:	False	OrderByOnLoad:	True
Orientation:	Left-to-Right	RecordLocks:	Edited Record
RecordsAffected:	0	ReturnsRecords:	True
Type:	64	Updatable:	True
UseTransaction:	True		

SQL

```
INSERT INTO Transformationsschritte ( Art, Old_ID, Prozess, Schrittid, ZeitvonStart, Zeitvondavor,
Bemerkungen, Light, Temperature )
SELECT [Forms]![Method form E_2]![Protokoll ID], CopySingleTrafoStep_select_2.ID,
CopySingleTrafoStep_select_2.Prozess, CopySingleTrafoStep_select_2.Schrittid,
CopySingleTrafoStep_select_2.ZeitvonStart, CopySingleTrafoStep_select_2.Zeitvondavor,
CopySingleTrafoStep_select_2.Bemerkungen, CopySingleTrafoStep_select_2.Light,
CopySingleTrafoStep_select_2.Temperature
FROM CopySingleTrafoStep_select_2;
```

Query Parameters

Name	Type
[Forms]![Method form E_2]![SelectedTrafoStep]	Text
[Forms]![Method form E_2]![Protokoll ID]	Text

User Permissions

admin	Delete; Read Permissions; Set Permissions; Change Owner, Read Definition; Write Definition; Read Data; Insert Data; Update Data; Delete Data

Group Permissions

Admins	Delete; Read Permissions; Set Permissions; Change Owner, Read Definition; Write Definition; Read Data; Insert Data; Update Data; Delete Data
Users	Delete; Read Permissions; Set Permissions; Change Owner, Read Definition; Write Definition; Read Data; Insert Data; Update Data; Delete Data

Properties

DateCreated:	16.10.2009 10:53:36	DefaultView:	2
DisplayViewsOnSharePointSit	1	DOL:	Long binary data
FilterOnLoad:	False	GUID:	{guid {CDC47F1C-F3F3-41A6-A63A-7BA9F9F1DE2D}}
HideNewField:	False	LastUpdated:	16.10.2009 10:56:03
MaxRecords:	0	NameMap:	Long binary data
ODBCTimeout:	60	OrderByOn:	False
OrderByOnLoad:	True	Orientation:	Left-to-Right
RecordLocks:	No Locks	RecordsAffected:	0
RecordsetType:	Dynaset	ReturnsRecords:	True
RowHeight:	315	TotalsRow:	False
Type:	0	Updatable:	True

SQL

```
SELECT Transformationsschritte.ID, Transformationsschritte.Art, Transformationsschritte.Prozess,
Transformationsschritte.Schrittid, Transformationsschritte.ZeitvonStart, Transformationsschritte.Zeitvondavor,
Transformationsschritte.Bemerkungen, Transformationsschritte.Bemerkungen, Transformationsschritte.Light,
Transformationsschritte.Temperature
FROM Transformationsschritte
WHERE (((Transformationsschritte.ID)=[Forms]![Method form E_1]![SelectedTrafoStep]));
```

Query Parameters

Name	Type
[Forms]![Method form E_1]![SelectedTrafoStep]	Text

Columns

Name	Type	Size
ID	Long Integer	4

AggregateType:	-1
AllowZeroLength:	False
AppendOnly:	False
Attributes:	Fixed Size; Auto-Increment; Updatable
CollatingOrder:	General
ColumnHidden:	False
ColumnOrder:	Default
ColumnWidth:	1035
DataUpdatable:	True
GUID:	{guid {C3183DBA-40CD-487D-B59E-DEBC1DA2563C}}
OrdinalPosition:	0
Required:	False
SourceField:	ID
SourceTable:	Transformationsschritte
TextAlign:	General

Name	Type	Size
Art	Long Integer	4

AggregateType:	-1
AllowValueListEdits:	False

AllowZeroLength:	False
AppendOnly:	False
Attributes:	Fixed Size; Updatable
BoundColumn:	1
CollatingOrder:	General
ColumnCount:	2
ColumnHeads:	False
ColumnHidden:	False
ColumnOrder:	Default
ColumnWidth:	3765
ColumnWidths:	0;3420
DataUpdatable:	True
DecimalPlaces:	Auto
DisplayControl:	Combo Box
GUID:	{guid {E1009E6F-96BB-4EC6-BA36-284C7914E778}}
LimitToList:	True
ListRows:	8
ListWidth:	3420twip
OrdinalPosition:	1
Required:	False
RowSource:	SELECT Protokoll.[Protokoll ID], Protokoll.Protokollname FROM Protokoll;
RowSourceType:	Table/Query
ShowOnlyRowSourceValues:	False
SourceField:	Art
SourceTable:	Transformationsschritte
TextAlign:	General

Prozess Text 50

AggregateType:	-1
AllowZeroLength:	True
AppendOnly:	False
Attributes:	Variable Length; Updatable
CollatingOrder:	General
ColumnHidden:	False
ColumnOrder:	Default
ColumnWidth:	2910
DataUpdatable:	True
Description:	Arbeitsprozess
DisplayControl:	Text Box
GUID:	{guid {F145B5A2-C291-40CF-8D68-20B48F7A3039}}
IMEMode:	0
IMESentenceMode:	3
OrdinalPosition:	2
Required:	False
SourceField:	Prozess
SourceTable:	Transformationsschritte
TextAlign:	General
UnicodeCompression:	True

Schrittid Long Integer 4

AggregateType:	-1
AllowZeroLength:	False
AppendOnly:	False
Attributes:	Fixed Size; Updatable

CollatingOrder:	General	
ColumnHidden:	False	
ColumnOrder:	Default	
ColumnWidth:	1095	
DataUpdatable:	True	
DecimalPlaces:	Auto	
DisplayControl:	Text Box	
GUID:	{guid {24937494-0814-4755-B289-0B4756C3E052}}	
OrdinalPosition:	3	
Required:	False	
SourceField:	Schrittid	
SourceTable:	Transformationsschritte	
TextAlign:	General	
**ZeitvonStart**	Long Integer	4
AggregateType:	-1	
AllowZeroLength:	False	
AppendOnly:	False	
Attributes:	Fixed Size; Updatable	
CollatingOrder:	General	
ColumnHidden:	False	
ColumnOrder:	Default	
ColumnWidth:	1140	
DataUpdatable:	True	
DecimalPlaces:	Auto	
Description:	Zeit in Tagen seit dem Transformationsdatum	
DisplayControl:	Text Box	
GUID:	{guid {C998F549-C87B-453C-BBCE-2D8190464E13}}	
OrdinalPosition:	4	
Required:	False	
SourceField:	ZeitvonStart	
SourceTable:	Transformationsschritte	
TextAlign:	General	
**Zeitvondavor**	Long Integer	4
AggregateType:	-1	
AllowZeroLength:	False	
AppendOnly:	False	
Attributes:	Fixed Size; Updatable	
CollatingOrder:	General	
ColumnHidden:	False	
ColumnOrder:	Default	
ColumnWidth:	1200	
DataUpdatable:	True	
DecimalPlaces:	Auto	
Description:	Zeit in Tagen gerechnet vom davor erfolgenden Schritt	
DisplayControl:	Text Box	
GUID:	{guid {AF59A193-C17F-4340-8F4F-4B461FD7B1C0}}	
OrdinalPosition:	5	
Required:	False	
SourceField:	Zeitvondavor	
SourceTable:	Transformationsschritte	
TextAlign:	General	
**Expr1006**	Memo	N/A
AggregateType:	-1	

	AllowZeroLength:	True
	AppendOnly:	False
	Attributes:	Variable Length; Updatable
	CollatingOrder:	General
	ColumnHidden:	False
	ColumnOrder:	Default
	ColumnWidth:	7740
	DataUpdatable:	True
	GUID:	{guid {5521D885-5AA9-4E67-90F5-02DF98AE027F}}
	IMEMode:	0
	IMESentenceMode:	3
	OrdinalPosition:	6
	Required:	False
	SourceField:	Bemerkungen
	SourceTable:	Transformationsschritte
	TextAlign:	General
	TextFormat:	Plain Text
	UnicodeCompression:	True

Bemerkungen		Memo	N/A
	AggregateType:	-1	
	AllowZeroLength:	True	
	AppendOnly:	False	
	Attributes:	Variable Length; Updatable	
	CollatingOrder:	General	
	ColumnHidden:	False	
	ColumnOrder:	Default	
	ColumnWidth:	7740	
	DataUpdatable:	True	
	GUID:	{guid {5521D885-5AA9-4E67-90F5-02DF98AE027F}}	
	IMEMode:	0	
	IMESentenceMode:	3	
	OrdinalPosition:	7	
	Required:	False	
	SourceField:	Bemerkungen	
	SourceTable:	Transformationsschritte	
	TextAlign:	General	
	TextFormat:	Plain Text	
	UnicodeCompression:	True	
Light		Text	50
	AggregateType:	-1	
	AllowZeroLength:	True	
	AppendOnly:	False	
	Attributes:	Variable Length; Updatable	
	CollatingOrder:	General	
	ColumnHidden:	False	
	ColumnOrder:	Default	
	ColumnWidth:	Default	
	DataUpdatable:	True	
	Description:	Light intensity	
	DisplayControl:	Text Box	
	GUID:	{guid {02C88938-E1C3-4C76-B042-4F471E040019}}	
	IMEMode:	0	
	IMESentenceMode:	3	
	OrdinalPosition:	8	

Required:	False	
SourceField:	Light	
SourceTable:	Transformationsschritte	
TextAlign:	General	
UnicodeCompression:	False	

Temperature	Long Integer	4
AggregateType:	-1	
AllowZeroLength:	False	
AppendOnly:	False	
Attributes:	Fixed Size; Updatable	
CollatingOrder:	General	
ColumnHidden:	False	
ColumnOrder:	Default	
ColumnWidth:	Default	
DataUpdatable:	True	
DecimalPlaces:	Auto	
Description:	Temperature in °C	
DisplayControl:	Text Box	
GUID:	{guid {4EC98804-4E45-4C1A-94AF-2CE1BA22CC38}}	
OrdinalPosition:	9	
Required:	False	
SourceField:	Temperature	
SourceTable:	Transformationsschritte	
TextAlign:	General	

Table Indexes

Name	Number of Fields
Old_ID	1
Clustered:	False
DistinctCount:	4
Foreign:	False
IgnoreNulls:	False
Name:	Old_ID
Primary:	False
Required:	False
Unique:	False
Fields:	
Old_ID	Ascending
PrimaryKey	1
Clustered:	False
DistinctCount:	9
Foreign:	False
IgnoreNulls:	False
Name:	PrimaryKey
Primary:	True
Required:	True
Unique:	True
Fields:	
ID	Ascending

User Permissions

admin	Delete; Read Permissions; Set Permissions; Change Owner, Read Definition; Write Definition; Read Data; Insert Data; Update Data; Delete Data

Group Permissions

Admins	Delete; Read Permissions; Set Permissions; Change Owner, Read Definition; Write Definition; Read Data; Insert Data; Update Data; Delete Data
Users	Delete; Read Permissions; Set Permissions; Change Owner, Read Definition; Write Definition; Read Data; Insert Data; Update Data; Delete Data

Properties

DateCreated:	27.03.2009 12:41:21	DefaultView:	2
DisplayViewsOnSharePointSit	1	DOL:	Long binary data
FilterOnLoad:	False	GUID:	{guid {16DB0B34-B695-42C4-92CF-6045313D166D}}
HideNewField:	False	LastUpdated:	16.10.2009 11:19:42
MaxRecords:	0	NameMap:	Long binary data
ODBCTimeout:	60	OrderByOn:	False
OrderByOnLoad:	True	Orientation:	Left-to-Right
RecordLocks:	No Locks	RecordsAffected:	0
RecordsetType:	Dynaset	ReturnsRecords:	True
RowHeight:	315	TotalsRow:	False
Type:	0	Updatable:	True

SQL

```
SELECT Transformationsschritte.ID, Transformationsschritte.Art, Transformationsschritte.Prozess,
Transformationsschritte.Schrittid, Transformationsschritte.ZeitvonStart, Transformationsschritte.Zeitvondavor,
Transformationsschritte.Bemerkungen, Transformationsschritte.Bemerkungen, Transformationsschritte.Light,
Transformationsschritte.Temperature
FROM Transformationsschritte
WHERE (((Transformationsschritte.ID)=[Forms]![Method form E_2]![SelectedTrafoStep]));
```

Query Parameters

Name	Type
[Forms]![Method form E_2]![SelectedTrafoStep]	Text

Columns

Name	Type	Size
ID	Long Integer	4

AggregateType:	-1
AllowZeroLength:	False
AppendOnly:	False
Attributes:	Fixed Size; Auto-Increment; Updatable
CollatingOrder:	General
ColumnHidden:	False
ColumnOrder:	Default
ColumnWidth:	1035
DataUpdatable:	True
GUID:	{guid {C3183DBA-40CD-487D-B59E-DEBC1DA2563C}}
OrdinalPosition:	0
Required:	False
SourceField:	ID
SourceTable:	Transformationsschritte
TextAlign:	General

Name	Type	Size
Art	Long Integer	4

AggregateType:	-1
AllowValueListEdits:	False

AllowZeroLength:	False
AppendOnly:	False
Attributes:	Fixed Size; Updatable
BoundColumn:	1
CollatingOrder:	General
ColumnCount:	2
ColumnHeads:	False
ColumnHidden:	False
ColumnOrder:	Default
ColumnWidth:	3765
ColumnWidths:	0;3420
DataUpdatable:	True
DecimalPlaces:	Auto
DisplayControl:	Combo Box
GUID:	{guid {E1009E6F-96BB-4EC6-BA36-284C7914E778}}
LimitToList:	True
ListRows:	8
ListWidth:	3420twip
OrdinalPosition:	1
Required:	False
RowSource:	SELECT Protokoll.[Protokoll ID], Protokoll.Protokollname FROM Protokoll;
RowSourceType:	Table/Query
ShowOnlyRowSourceValues:	False
SourceField:	Art
SourceTable:	Transformationsschritte
TextAlign:	General

Prozess Text 50

AggregateType:	-1
AllowZeroLength:	True
AppendOnly:	False
Attributes:	Variable Length; Updatable
CollatingOrder:	General
ColumnHidden:	False
ColumnOrder:	Default
ColumnWidth:	2910
DataUpdatable:	True
Description:	Arbeitsprozess
DisplayControl:	Text Box
GUID:	{guid {F145B5A2-C291-40CF-8D68-20B48F7A3039}}
IMEMode:	0
IMESentenceMode:	3
OrdinalPosition:	2
Required:	False
SourceField:	Prozess
SourceTable:	Transformationsschritte
TextAlign:	General
UnicodeCompression:	True

Schrittid Long Integer 4

AggregateType:	-1
AllowZeroLength:	False
AppendOnly:	False
Attributes:	Fixed Size; Updatable

CollatingOrder:	General	
ColumnHidden:	False	
ColumnOrder:	Default	
ColumnWidth:	1095	
DataUpdatable:	True	
DecimalPlaces:	Auto	
DisplayControl:	Text Box	
GUID:	{guid {24937494-0814-4755-B289-0B4756C3E052}}	
OrdinalPosition:	3	
Required:	False	
SourceField:	Schrittid	
SourceTable:	Transformationsschritte	
TextAlign:	General	

ZeitvonStart	Long Integer	4
AggregateType:	-1	
AllowZeroLength:	False	
AppendOnly:	False	
Attributes:	Fixed Size; Updatable	
CollatingOrder:	General	
ColumnHidden:	False	
ColumnOrder:	Default	
ColumnWidth:	1140	
DataUpdatable:	True	
DecimalPlaces:	Auto	
Description:	Zeit in Tagen seit dem Transformationsdatum	
DisplayControl:	Text Box	
GUID:	{guid {C998F549-C87B-453C-BBCE-2D8190464E13}}	
OrdinalPosition:	4	
Required:	False	
SourceField:	ZeitvonStart	
SourceTable:	Transformationsschritte	
TextAlign:	General	

Zeitvondavor	Long Integer	4
AggregateType:	-1	
AllowZeroLength:	False	
AppendOnly:	False	
Attributes:	Fixed Size; Updatable	
CollatingOrder:	General	
ColumnHidden:	False	
ColumnOrder:	Default	
ColumnWidth:	1200	
DataUpdatable:	True	
DecimalPlaces:	Auto	
Description:	Zeit in Tagen gerechnet vom davor erfolgenden Schritt	
DisplayControl:	Text Box	
GUID:	{guid {AF59A193-C17F-4340-8F4F-4B461FD7B1C0}}	
OrdinalPosition:	5	
Required:	False	
SourceField:	Zeitvondavor	
SourceTable:	Transformationsschritte	
TextAlign:	General	

Expr1006	Memo	N/A
AggregateType:	-1	

AllowZeroLength:	True
AppendOnly:	False
Attributes:	Variable Length; Updatable
CollatingOrder:	General
ColumnHidden:	False
ColumnOrder:	Default
ColumnWidth:	7740
DataUpdatable:	True
GUID:	{guid {5521D885-5AA9-4E67-90F5-02DF98AE027F}}
IMEMode:	0
IMESentenceMode:	3
OrdinalPosition:	6
Required:	False
SourceField:	Bemerkungen
SourceTable:	Transformationsschritte
TextAlign:	General
TextFormat:	Plain Text
UnicodeCompression:	True

Bemerkungen Memo N/A

AggregateType:	-1
AllowZeroLength:	True
AppendOnly:	False
Attributes:	Variable Length; Updatable
CollatingOrder:	General
ColumnHidden:	False
ColumnOrder:	Default
ColumnWidth:	7740
DataUpdatable:	True
GUID:	{guid {5521D885-5AA9-4E67-90F5-02DF98AE027F}}
IMEMode:	0
IMESentenceMode:	3
OrdinalPosition:	7
Required:	False
SourceField:	Bemerkungen
SourceTable:	Transformationsschritte
TextAlign:	General
TextFormat:	Plain Text
UnicodeCompression:	True

Light Text 50

AggregateType:	-1
AllowZeroLength:	True
AppendOnly:	False
Attributes:	Variable Length; Updatable
CollatingOrder:	General
ColumnHidden:	False
ColumnOrder:	Default
ColumnWidth:	Default
DataUpdatable:	True
Description:	Light intensity
DisplayControl:	Text Box
GUID:	{guid {02C88938-E1C3-4C76-B042-4F471E040019}}
IMEMode:	0
IMESentenceMode:	3
OrdinalPosition:	8

Required:	False
SourceField:	Light
SourceTable:	Transformationsschritte
TextAlign:	General
UnicodeCompression:	False

Temperature Long Integer 4

AggregateType:	-1
AllowZeroLength:	False
AppendOnly:	False
Attributes:	Fixed Size; Updatable
CollatingOrder:	General
ColumnHidden:	False
ColumnOrder:	Default
ColumnWidth:	Default
DataUpdatable:	True
DecimalPlaces:	Auto
Description:	Temperature in °C
DisplayControl:	Text Box
GUID:	{guid {4EC98804-4E45-4C1A-94AF-2CE1BA22CC38}}
OrdinalPosition:	9
Required:	False
SourceField:	Temperature
SourceTable:	Transformationsschritte
TextAlign:	General

Table Indexes

Name	Number of Fields
Old_ID	1

Clustered:	False
DistinctCount:	4
Foreign:	False
IgnoreNulls:	False
Name:	Old_ID
Primary:	False
Required:	False
Unique:	False
Fields:	
Old_ID	Ascending

Name	Number of Fields
PrimaryKey	1

Clustered:	False
DistinctCount:	9
Foreign:	False
IgnoreNulls:	False
Name:	PrimaryKey
Primary:	True
Required:	True
Unique:	True
Fields:	
ID	Ascending

User Permissions

admin	Delete; Read Permissions; Set Permissions; Change Owner, Read Definition; Write Definition; Read Data; Insert Data; Update Data; Delete Data

Group Permissions

Admins	Delete; Read Permissions; Set Permissions; Change Owner, Read Definition; Write Definition; Read Data; Insert Data; Update Data; Delete Data
Users	Delete; Read Permissions; Set Permissions; Change Owner, Read Definition; Write Definition; Read Data; Insert Data; Update Data; Delete Data

Properties

DateCreated:	16.10.2009 10:53:36	DefaultView:	2
DOL:	Long binary data	FilterOnLoad:	False
GUID:	{guid {50DFCFE3-548F-4B5F-87D0-E1EEE4DCD832}}	LastUpdated:	16.10.2009 11:21:10
MaxRecords:	0	ODBCTimeout:	60
OrderByOn:	False	OrderByOnLoad:	True
Orientation:	Left-to-Right	RecordLocks:	Edited Record
RecordsAffected:	0	ReturnsRecords:	True
Type:	64	Updatable:	True
UseTransaction:	True		

SQL

```
INSERT INTO Transformationsschritte ( Prozess, Schrittid, ZeitvonStart, Zeitvondavor, Bemerkungen, Light,
Temperature, Art, Old_ID )
SELECT [CopyTransformationsschritte_select_1.Prozess]+'_new' AS Expr1,
CopyTransformationsschritte_select_1.Schrittid, CopyTransformationsschritte_select_1.ZeitvonStart,
CopyTransformationsschritte_select_1.Zeitvondavor, CopyTransformationsschritte_select_1.Bemerkungen,
CopyTransformationsschritte_select_1.Light, CopyTransformationsschritte_select_1.Temperature,
CopyProtokoll_Select_NewProtokollID_1.[Protokoll ID], CopyTransformationsschritte_select_1.ID
FROM CopyTransformationsschritte_select_1, CopyProtokoll_Select_NewProtokollID_1;
```

Query Parameters

Name	Type
[Forms]![Method form E_1]![Protokoll ID]	Text

User Permissions

admin	Delete; Read Permissions; Set Permissions; Change Owner, Read Definition; Write Definition; Read Data; Insert Data; Update Data; Delete Data

Group Permissions

Admins	Delete; Read Permissions; Set Permissions; Change Owner, Read Definition; Write Definition; Read Data; Insert Data; Update Data; Delete Data
Users	Delete; Read Permissions; Set Permissions; Change Owner, Read Definition; Write Definition; Read Data; Insert Data; Update Data; Delete Data

Properties

DateCreated:	27.03.2009 12:41:21	DefaultView:	2
DOL:	Long binary data	FilterOnLoad:	False
GUID:	{guid {FF1CAD5E-4DDF-4E44-940F-DBF5A8935711}}	LastUpdated:	16.10.2009 11:22:23
MaxRecords:	0	ODBCTimeout:	60
OrderByOn:	False	OrderByOnLoad:	True
Orientation:	Left-to-Right	RecordLocks:	Edited Record
RecordsAffected:	0	ReturnsRecords:	True
Type:	64	Updatable:	True
UseTransaction:	True		

SQL

```
INSERT INTO Transformationsschritte ( Prozess, Schrittid, ZeitvonStart, Zeitvondavor, Bemerkungen, Light,
Temperature, Art, Old_ID )
SELECT [CopyTransformationsschritte_select_2.Prozess]+'_new' AS Expr1,
CopyTransformationsschritte_select_2.Schrittid, CopyTransformationsschritte_select_2.ZeitvonStart,
CopyTransformationsschritte_select_2.Zeitvondavor, CopyTransformationsschritte_select_2.Bemerkungen,
CopyTransformationsschritte_select_2.Light, CopyTransformationsschritte_select_2.Temperature,
CopyProtokoll_Select_NewProtokollID_2.[Protokoll ID], CopyTransformationsschritte_select_2.ID
FROM CopyTransformationsschritte_select_2, CopyProtokoll_Select_NewProtokollID_2;
```

Query Parameters

Name	Type
[Forms]![Method form E_2]![Protokoll ID]	Text

User Permissions

admin	Delete; Read Permissions; Set Permissions; Change Owner, Read Definition; Write Definition; Read Data; Insert Data; Update Data; Delete Data

Group Permissions

Admins	Delete; Read Permissions; Set Permissions; Change Owner, Read Definition; Write Definition; Read Data; Insert Data; Update Data; Delete Data
Users	Delete; Read Permissions; Set Permissions; Change Owner, Read Definition; Write Definition; Read Data; Insert Data; Update Data; Delete Data

Properties

DateCreated:	16.10.2009 10:59:05	DefaultView:	2
DisplayViewsOnSharePointSit	1	DOL:	Long binary data
FilterOnLoad:	False	GUID:	{guid {CDC47F1C-F3F3-41A6-A63A-7BA9F9F1DE2D}}
HideNewField:	False	LastUpdated:	16.10.2009 10:59:29
MaxRecords:	0	NameMap:	Long binary data
ODBCTimeout:	60	OrderByOn:	False
OrderByOnLoad:	True	Orientation:	Left-to-Right
RecordLocks:	No Locks	RecordsAffected:	0
RecordsetType:	Dynaset	ReturnsRecords:	True
RowHeight:	315	TotalsRow:	False
Type:	0	Updatable:	True

SQL

```
SELECT Transformationsschritte.ID, Transformationsschritte.Art, Transformationsschritte.Prozess,
Transformationsschritte.Schrittid, Transformationsschritte.ZeitvonStart, Transformationsschritte.Zeitvondavor,
Transformationsschritte.Bemerkungen, Transformationsschritte.Light, Transformationsschritte.Temperature
FROM Transformationsschritte
WHERE (((Transformationsschritte.Art)=[Forms]![Method form E_1]![Protokoll ID]));
```

Query Parameters

Name	Type
[Forms]![Method form E_1]![Protokoll ID]	Text

Columns

Name	Type	Size
ID	Long Integer	4

AggregateType:	-1
AllowZeroLength:	False
AppendOnly:	False
Attributes:	Fixed Size; Auto-Increment; Updatable
CollatingOrder:	General
ColumnHidden:	False
ColumnOrder:	Default
ColumnWidth:	1035
DataUpdatable:	True
GUID:	{guid {C3183DBA-40CD-487D-B59E-DEBC1DA2563C}}
OrdinalPosition:	0
Required:	False
SourceField:	ID
SourceTable:	Transformationsschritte
TextAlign:	General

Name	Type	Size
Art	Long Integer	4

AggregateType:	-1
AllowValueListEdits:	False
AllowZeroLength:	False

AppendOnly:	False
Attributes:	Fixed Size; Updatable
BoundColumn:	1
CollatingOrder:	General
ColumnCount:	2
ColumnHeads:	False
ColumnHidden:	False
ColumnOrder:	Default
ColumnWidth:	3765
ColumnWidths:	0;3420
DataUpdatable:	True
DecimalPlaces:	Auto
DisplayControl:	Combo Box
GUID:	{guid {E1009E6F-96BB-4EC6-BA36-284C7914E778}}
LimitToList:	True
ListRows:	8
ListWidth:	3420twip
OrdinalPosition:	1
Required:	False
RowSource:	SELECT Protokoll.[Protokoll ID], Protokoll.Protokollname FROM Protokoll;
RowSourceType:	Table/Query
ShowOnlyRowSourceValues:	False
SourceField:	Art
SourceTable:	Transformationsschritte
TextAlign:	General

Prozess Text 50

AggregateType:	-1
AllowZeroLength:	True
AppendOnly:	False
Attributes:	Variable Length; Updatable
CollatingOrder:	General
ColumnHidden:	False
ColumnOrder:	Default
ColumnWidth:	2910
DataUpdatable:	True
Description:	Arbeitsprozess
DisplayControl:	Text Box
GUID:	{guid {F145B5A2-C291-40CF-8D68-20B48F7A3039}}
IMEMode:	0
IMESentenceMode:	3
OrdinalPosition:	2
Required:	False
SourceField:	Prozess
SourceTable:	Transformationsschritte
TextAlign:	General
UnicodeCompression:	True

Schrittid Long Integer 4

AggregateType:	-1
AllowZeroLength:	False
AppendOnly:	False
Attributes:	Fixed Size; Updatable
CollatingOrder:	General

	ColumnHidden:	False
	ColumnOrder:	Default
	ColumnWidth:	1095
	DataUpdatable:	True
	DecimalPlaces:	Auto
	DisplayControl:	Text Box
	GUID:	{guid {24937494-0814-4755-B289-0B4756C3E052}}
	OrdinalPosition:	3
	Required:	False
	SourceField:	Schrittid
	SourceTable:	Transformationsschritte
	TextAlign:	General
ZeitvonStart		Long Integer 4
	AggregateType:	-1
	AllowZeroLength:	False
	AppendOnly:	False
	Attributes:	Fixed Size; Updatable
	CollatingOrder:	General
	ColumnHidden:	False
	ColumnOrder:	Default
	ColumnWidth:	1140
	DataUpdatable:	True
	DecimalPlaces:	Auto
	Description:	Zeit in Tagen seit dem Transformationsdatum
	DisplayControl:	Text Box
	GUID:	{guid {C998F549-C87B-453C-BBCE-2D8190464E13}}
	OrdinalPosition:	4
	Required:	False
	SourceField:	ZeitvonStart
	SourceTable:	Transformationsschritte
	TextAlign:	General
Zeitvondavor		Long Integer 4
	AggregateType:	-1
	AllowZeroLength:	False
	AppendOnly:	False
	Attributes:	Fixed Size; Updatable
	CollatingOrder:	General
	ColumnHidden:	False
	ColumnOrder:	Default
	ColumnWidth:	1200
	DataUpdatable:	True
	DecimalPlaces:	Auto
	Description:	Zeit in Tagen gerechnet vom davor erfolgenden Schritt
	DisplayControl:	Text Box
	GUID:	{guid {AF59A193-C17F-4340-8F4F-4B461FD7B1C0}}
	OrdinalPosition:	5
	Required:	False
	SourceField:	Zeitvondavor
	SourceTable:	Transformationsschritte
	TextAlign:	General
Bemerkungen		Memo N/A
	AggregateType:	-1
	AllowZeroLength:	True

	AppendOnly:	False
	Attributes:	Variable Length; Updatable
	CollatingOrder:	General
	ColumnHidden:	False
	ColumnOrder:	Default
	ColumnWidth:	7740
	DataUpdatable:	True
	GUID:	{guid {5521D885-5AA9-4E67-90F5-02DF98AE027F}}
	IMEMode:	0
	IMESentenceMode:	3
	OrdinalPosition:	6
	Required:	False
	SourceField:	Bemerkungen
	SourceTable:	Transformationsschritte
	TextAlign:	General
	TextFormat:	Plain Text
	UnicodeCompression:	True

Light		Text	50
	AggregateType:	-1	
	AllowZeroLength:	True	
	AppendOnly:	False	
	Attributes:	Variable Length; Updatable	
	CollatingOrder:	General	
	ColumnHidden:	False	
	ColumnOrder:	Default	
	ColumnWidth:	Default	
	DataUpdatable:	True	
	Description:	Light intensity	
	DisplayControl:	Text Box	
	GUID:	{guid {02C88938-E1C3-4C76-B042-4F471E040019}}	
	IMEMode:	0	
	IMESentenceMode:	3	
	OrdinalPosition:	7	
	Required:	False	
	SourceField:	Light	
	SourceTable:	Transformationsschritte	
	TextAlign:	General	
	UnicodeCompression:	False	

Temperature		Long Integer	4
	AggregateType:	-1	
	AllowZeroLength:	False	
	AppendOnly:	False	
	Attributes:	Fixed Size; Updatable	
	CollatingOrder:	General	
	ColumnHidden:	False	
	ColumnOrder:	Default	
	ColumnWidth:	Default	
	DataUpdatable:	True	
	DecimalPlaces:	Auto	
	Description:	Temperature in °C	
	DisplayControl:	Text Box	
	GUID:	{guid {4EC98804-4E45-4C1A-94AF-2CE1BA22CC38}}	
	OrdinalPosition:	8	
	Required:	False	

SourceField:	Temperature
SourceTable:	Transformationsschritte
TextAlign:	General

Table Indexes

Name		Number of Fields
Old_ID		1
	Clustered:	False
	DistinctCount:	4
	Foreign:	False
	IgnoreNulls:	False
	Name:	Old_ID
	Primary:	False
	Required:	False
	Unique:	False
	Fields:	
	Old_ID	Ascending
PrimaryKey		1
	Clustered:	False
	DistinctCount:	9
	Foreign:	False
	IgnoreNulls:	False
	Name:	PrimaryKey
	Primary:	True
	Required:	True
	Unique:	True
	Fields:	
	ID	Ascending

User Permissions

admin	Delete; Read Permissions; Set Permissions; Change Owner, Read Definition; Write Definition; Read Data; Insert Data; Update Data; Delete Data

Group Permissions

Admins	Delete; Read Permissions; Set Permissions; Change Owner, Read Definition; Write Definition; Read Data; Insert Data; Update Data; Delete Data
Users	Delete; Read Permissions; Set Permissions; Change Owner, Read Definition; Write Definition; Read Data; Insert Data; Update Data; Delete Data

Properties

DateCreated:	27.03.2009 12:41:21	DefaultView:	2
DisplayViewsOnSharePointSit	1	DOL:	Long binary data
FilterOnLoad:	False	GUID:	{guid {FBD43D1E-031B-47DF-859A-89688FAE8325}}
HideNewField:	False	LastUpdated:	16.10.2009 11:23:00
MaxRecords:	0	NameMap:	Long binary data
ODBCTimeout:	60	OrderByOn:	False
OrderByOnLoad:	True	Orientation:	Left-to-Right
RecordLocks:	No Locks	RecordsAffected:	0
RecordsetType:	Dynaset	ReturnsRecords:	True
RowHeight:	315	TotalsRow:	False
Type:	0	Updatable:	True

SQL

```
SELECT Transformationsschritte.ID, Transformationsschritte.Art, Transformationsschritte.Prozess,
Transformationsschritte.Schrittid, Transformationsschritte.ZeitvonStart, Transformationsschritte.Zeitvondavor,
Transformationsschritte.Bemerkungen, Transformationsschritte.Light, Transformationsschritte.Temperature
FROM Transformationsschritte
WHERE (((Transformationsschritte.Art)=[Forms]![Method form E_2]![Protokoll ID]));
```

Query Parameters

Name	Type
[Forms]![Method form E_2]![Protokoll ID]	Text

Columns

Name	Type	Size
ID	Long Integer	4

AggregateType:	-1
AllowZeroLength:	False
AppendOnly:	False
Attributes:	Fixed Size; Auto-Increment; Updatable
CollatingOrder:	General
ColumnHidden:	False
ColumnOrder:	Default
ColumnWidth:	1035
DataUpdatable:	True
GUID:	{guid {C3183DBA-40CD-487D-B59E-DEBC1DA2563C}}
OrdinalPosition:	0
Required:	False
SourceField:	ID
SourceTable:	Transformationsschritte
TextAlign:	General

Name	Type	Size
Art	Long Integer	4

AggregateType:	-1
AllowValueListEdits:	False
AllowZeroLength:	False

AppendOnly:	False
Attributes:	Fixed Size; Updatable
BoundColumn:	1
CollatingOrder:	General
ColumnCount:	2
ColumnHeads:	False
ColumnHidden:	False
ColumnOrder:	Default
ColumnWidth:	3765
ColumnWidths:	0;3420
DataUpdatable:	True
DecimalPlaces:	Auto
DisplayControl:	Combo Box
GUID:	{guid {E1009E6F-96BB-4EC6-BA36-284C7914E778}}
LimitToList:	True
ListRows:	8
ListWidth:	3420twip
OrdinalPosition:	1
Required:	False
RowSource:	SELECT Protokoll.[Protokoll ID], Protokoll.Protokollname FROM Protokoll;
RowSourceType:	Table/Query
ShowOnlyRowSourceValues:	False
SourceField:	Art
SourceTable:	Transformationsschritte
TextAlign:	General

Prozess | Text | 50

AggregateType:	-1
AllowZeroLength:	True
AppendOnly:	False
Attributes:	Variable Length; Updatable
CollatingOrder:	General
ColumnHidden:	False
ColumnOrder:	Default
ColumnWidth:	2910
DataUpdatable:	True
Description:	Arbeitsprozess
DisplayControl:	Text Box
GUID:	{guid {F145B5A2-C291-40CF-8D68-20B48F7A3039}}
IMEMode:	0
IMESentenceMode:	3
OrdinalPosition:	2
Required:	False
SourceField:	Prozess
SourceTable:	Transformationsschritte
TextAlign:	General
UnicodeCompression:	True

Schrittid | Long Integer | 4

AggregateType:	-1
AllowZeroLength:	False
AppendOnly:	False
Attributes:	Fixed Size; Updatable
CollatingOrder:	General

ColumnHidden:	False	
ColumnOrder:	Default	
ColumnWidth:	1095	
DataUpdatable:	True	
DecimalPlaces:	Auto	
DisplayControl:	Text Box	
GUID:	{guid {24937494-0814-4755-B289-0B4756C3E052}}	
OrdinalPosition:	3	
Required:	False	
SourceField:	Schrittid	
SourceTable:	Transformationsschritte	
TextAlign:	General	

ZeitvonStart	Long Integer	4
AggregateType:	-1	
AllowZeroLength:	False	
AppendOnly:	False	
Attributes:	Fixed Size; Updatable	
CollatingOrder:	General	
ColumnHidden:	False	
ColumnOrder:	Default	
ColumnWidth:	1140	
DataUpdatable:	True	
DecimalPlaces:	Auto	
Description:	Zeit in Tagen seit dem Transformationsdatum	
DisplayControl:	Text Box	
GUID:	{guid {C998F549-C87B-453C-BBCE-2D8190464E13}}	
OrdinalPosition:	4	
Required:	False	
SourceField:	ZeitvonStart	
SourceTable:	Transformationsschritte	
TextAlign:	General	

Zeitvondavor	Long Integer	4
AggregateType:	-1	
AllowZeroLength:	False	
AppendOnly:	False	
Attributes:	Fixed Size; Updatable	
CollatingOrder:	General	
ColumnHidden:	False	
ColumnOrder:	Default	
ColumnWidth:	1200	
DataUpdatable:	True	
DecimalPlaces:	Auto	
Description:	Zeit in Tagen gerechnet vom davor erfolgenden Schritt	
DisplayControl:	Text Box	
GUID:	{guid {AF59A193-C17F-4340-8F4F-4B461FD7B1C0}}	
OrdinalPosition:	5	
Required:	False	
SourceField:	Zeitvondavor	
SourceTable:	Transformationsschritte	
TextAlign:	General	

Bemerkungen	Memo	N/A
AggregateType:	-1	
AllowZeroLength:	True	

AppendOnly:	False
Attributes:	Variable Length; Updatable
CollatingOrder:	General
ColumnHidden:	False
ColumnOrder:	Default
ColumnWidth:	7740
DataUpdatable:	True
GUID:	{guid {5521D885-5AA9-4E67-90F5-02DF98AE027F}}
IMEMode:	0
IMESentenceMode:	3
OrdinalPosition:	6
Required:	False
SourceField:	Bemerkungen
SourceTable:	Transformationsschritte
TextAlign:	General
TextFormat:	Plain Text
UnicodeCompression:	True

Light Text 50

AggregateType:	-1
AllowZeroLength:	True
AppendOnly:	False
Attributes:	Variable Length; Updatable
CollatingOrder:	General
ColumnHidden:	False
ColumnOrder:	Default
ColumnWidth:	Default
DataUpdatable:	True
Description:	Light intensity
DisplayControl:	Text Box
GUID:	{guid {02C88938-E1C3-4C76-B042-4F471E040019}}
IMEMode:	0
IMESentenceMode:	3
OrdinalPosition:	7
Required:	False
SourceField:	Light
SourceTable:	Transformationsschritte
TextAlign:	General
UnicodeCompression:	False

Temperature Long Integer 4

AggregateType:	-1
AllowZeroLength:	False
AppendOnly:	False
Attributes:	Fixed Size; Updatable
CollatingOrder:	General
ColumnHidden:	False
ColumnOrder:	Default
ColumnWidth:	Default
DataUpdatable:	True
DecimalPlaces:	Auto
Description:	Temperature in °C
DisplayControl:	Text Box
GUID:	{guid {4EC98804-4E45-4C1A-94AF-2CE1BA22CC38}}
OrdinalPosition:	8
Required:	False

SourceField:	Temperature
SourceTable:	Transformationsschritte
TextAlign:	General

Table Indexes

Name		Number of Fields
Old_ID		1
	Clustered:	False
	DistinctCount:	4
	Foreign:	False
	IgnoreNulls:	False
	Name:	Old_ID
	Primary:	False
	Required:	False
	Unique:	False
	Fields:	
	Old_ID	Ascending
PrimaryKey		1
	Clustered:	False
	DistinctCount:	9
	Foreign:	False
	IgnoreNulls:	False
	Name:	PrimaryKey
	Primary:	True
	Required:	True
	Unique:	True
	Fields:	
	ID	Ascending

User Permissions

admin	Delete; Read Permissions; Set Permissions; Change Owner, Read Definition; Write Definition; Read Data; Insert Data; Update Data; Delete Data

Group Permissions

Admins	Delete; Read Permissions; Set Permissions; Change Owner, Read Definition; Write Definition; Read Data; Insert Data; Update Data; Delete Data
Users	Delete; Read Permissions; Set Permissions; Change Owner, Read Definition; Write Definition; Read Data; Insert Data; Update Data; Delete Data

Properties

DateCreated:	24.09.2009 09:54:12	GUID:	{guid {C8774ED8-0CC4-453F-A52E-BBF260B5DB7C}}
LastUpdated:	24.09.2009 09:54:13	MaxRecords:	0
ODBCTimeout:	60	Orientation:	Left-to-Right
RecordLocks:	Edited Record	RecordsAffected:	0
ReturnsRecords:	True	Type:	64
Updatable:	True	UseTransaction:	True

SQL

```
INSERT INTO Construct ( ConstructId, ConstructName, [M Resistance_text], [M Resistance],
PlantResistance_text, [Plant Resistance] )
SELECT Retrieve_Construct_Data_from_LIMS.U_CONSTRUCT_ID, Retrieve_Construct_Data_from_LIMS.NAME,
Retrieve_Construct_Data_from_LIMS.U_M_RESISTANCE,
Retrieve_Construct_Data_from_LIMS.ID_Resistenz_Bakterien,
Retrieve_Construct_Data_from_LIMS.U_PLANT_RESISTANCE,
Retrieve_Construct_Data_from_LIMS.ID_Resistenz_Pflanze
FROM Retrieve_Construct_Data_from_LIMS;
```

User Permissions

admin	Delete; Read Permissions; Set Permissions; Change Owner, Read Definition; Write Definition; Read Data; Insert Data; Update Data; Delete Data

Group Permissions

Admins	Delete; Read Permissions; Set Permissions; Change Owner, Read Definition; Write Definition; Read Data; Insert Data; Update Data; Delete Data
Users	Delete; Read Permissions; Set Permissions; Change Owner, Read Definition; Write Definition; Read Data; Insert Data; Update Data; Delete Data

Properties

DateCreated:	06.10.2009 10:32:06	DefaultView:	2
FilterOnLoad:	False	GUID:	{guid {42901101-F9DA-43D6-B58C-42B7A7719E37}}
LastUpdated:	06.10.2009 10:32:06	MaxRecords:	0
ODBCTimeout:	60	OrderByOn:	False
OrderByOnLoad:	True	Orientation:	Left-to-Right
RecordLocks:	Edited Record	RecordsAffected:	0
ReturnsRecords:	True	Type:	64
Updatable:	True	UseTransaction:	True

SQL

```
INSERT INTO Operator ( ID_LIMS, Logon_Name, Operator, active )
SELECT LIMS_SYS_OPERATOR.OPERATOR_ID, LIMS_SYS_OPERATOR.NAME,
LIMS_SYS_OPERATOR.FULL_NAME, ([LIMS_SYS_OPERATOR].[ALLOW_LOGIN]="T") AS Expr1
FROM LIMS_SYS_OPERATOR
WHERE (((LIMS_SYS_OPERATOR.OPERATOR_ID)>(select max(ID_LIMS) from  operator)));
```

User Permissions

admin	Delete; Read Permissions; Set Permissions; Change Owner, Read Definition; Write Definition; Read Data; Insert Data; Update Data; Delete Data

Group Permissions

Admins	Delete; Read Permissions; Set Permissions; Change Owner, Read Definition; Write Definition; Read Data; Insert Data; Update Data; Delete Data
Users	Delete; Read Permissions; Set Permissions; Change Owner, Read Definition; Write Definition; Read Data; Insert Data; Update Data; Delete Data

Properties

DateCreated:	12.11.2008 16:35:51	DefaultView:	2
DOL:	Long binary data	GUID:	{guid {36603041-DB64-4501-8048-E82897192C94}}
LastUpdated:	18.09.2009 14:34:02	MaxRecords:	0
ODBCTimeout:	60	OrderByOn:	False
Orientation:	Left-to-Right	RecordLocks:	Edited Record
RecordsAffected:	0	RecordsetType:	Dynaset
ReturnsRecords:	True	Type:	80
Updatable:	True	UseTransaction:	True

SQL

```
SELECT Medien.[Medien ID], Medien.Medienname, Medien.Remark, Medien.Solvent, Medien.Steril,
Medien.Storagecondition, Medien.Storagesite, Medienzusammensetzung.Medien,
Medienzusammensetzung.Menge, Medienzusammensetzung.Mengeneinheit, Medienzusammensetzung.Stock,
Medien_1.Medienname, Medien_1.Bezeichner AS BStock, Date() AS Datum, Medien.Bezeichner AS BMedium
INTO Karteikarten
FROM Medien INNER JOIN (Medienzusammensetzung INNER JOIN Medien AS Medien_1 ON
Medienzusammensetzung.Stock = Medien_1.[Medien ID]) ON Medien.[Medien ID] =
Medienzusammensetzung.Medien
WHERE (((Medien.Medienname) Not Like "*obsolet*"));
```

User Permissions

admin	Delete; Read Permissions; Set Permissions; Change Owner, Read Definition; Write Definition; Read Data; Insert Data; Update Data; Delete Data

Group Permissions

Admins	Delete; Read Permissions; Set Permissions; Change Owner, Read Definition; Write Definition; Read Data; Insert Data; Update Data; Delete Data
Users	Delete; Read Permissions; Set Permissions; Change Owner, Read Definition; Write Definition; Read Data; Insert Data; Update Data; Delete Data

Properties

DateCreated:	18.12.2008 16:03:29	DefaultView:	2
DOL:	Long binary data	FilterOnLoad:	False
GUID:	{guid {1134C266-0377-4777-8B41-BF6625B51E07}}	LastUpdated:	24.09.2009 11:28:20
MaxRecords:	0	ODBCTimeout:	60
OrderByOn:	False	OrderByOnLoad:	True
Orientation:	Left-to-Right	RecordLocks:	Edited Record
RecordsAffected:	0	RecordsetType:	Dynaset
ReturnsRecords:	True	TotalsRow:	False
Type:	0	Updatable:	True
UseTransaction:	True		

SQL

```
SELECT Medien.[Medien ID], Medien.Medienname, Medien.Remark, Medien.Solvent, Medien.Steril,
Medien.Storagesite, Date() AS Datum, Medien.Bezeichner, Storagecondition.Storagecondition_Name AS
Storagecondition
FROM Storagesite RIGHT JOIN (Storagecondition RIGHT JOIN Medien ON
Storagecondition.Storagecondition_ID = Medien.Storagecondition) ON Storagesite.Storagesite_ID =
Medien.Storagesite
WHERE (((Medien.Medienname) Not Like "*obsolet*"));
```

Columns

Name	Type	Size
Medien ID	Long Integer	4

AggregateType:	-1
AllowZeroLength:	False
AppendOnly:	False
Attributes:	Fixed Size; Auto-Increment; Updatable
CollatingOrder:	General
ColumnHidden:	False
ColumnOrder:	1
ColumnWidth:	Default
DataUpdatable:	True
GUID:	{guid {FBE4AC72-E216-4CE4-ACB3-246BABE85369}}
OrdinalPosition:	0
Required:	False
SourceField:	Medien ID
SourceTable:	Medien
TextAlign:	General

Name	Type	Size
Medienname	Text	50

AggregateType:	-1
AllowZeroLength:	True
AppendOnly:	False
Attributes:	Variable Length; Updatable
CollatingOrder:	General
ColumnHidden:	False
ColumnOrder:	Default
ColumnWidth:	3615
DataUpdatable:	True

	DisplayControl:	Text Box	
	GUID:	{guid {39A7EAC5-34A3-4BBD-A594-4E88A4399D39}}	
	IMEMode:	0	
	IMESentenceMode:	3	
	OrdinalPosition:	1	
	Required:	True	
	SourceField:	Medienname	
	SourceTable:	Medien	
	TextAlign:	General	
	UnicodeCompression:	True	
Remark		Memo	N/A
	AggregateType:	-1	
	AllowZeroLength:	True	
	AppendOnly:	False	
	Attributes:	Variable Length; Updatable	
	CollatingOrder:	General	
	ColumnHidden:	False	
	ColumnOrder:	Default	
	ColumnWidth:	2745	
	DataUpdatable:	True	
	GUID:	{guid {F6E09F04-6090-435D-AAE4-5157DDB09E51}}	
	IMEMode:	0	
	IMESentenceMode:	3	
	OrdinalPosition:	2	
	Required:	False	
	SourceField:	Remark	
	SourceTable:	Medien	
	TextAlign:	General	
	TextFormat:	Plain Text	
	UnicodeCompression:	True	
Solvent		Text	50
	AggregateType:	-1	
	AllowZeroLength:	True	
	AppendOnly:	False	
	Attributes:	Variable Length; Updatable	
	CollatingOrder:	General	
	ColumnHidden:	False	
	ColumnOrder:	Default	
	ColumnWidth:	Default	
	DataUpdatable:	True	
	DisplayControl:	Text Box	
	GUID:	{guid {07F6C1A3-CFAD-4E65-819D-ABBA6FF7B366}}	
	IMEMode:	0	
	IMESentenceMode:	3	
	OrdinalPosition:	3	
	Required:	False	
	SourceField:	Solvent	
	SourceTable:	Medien	
	TextAlign:	General	
	UnicodeCompression:	True	
Steril		Text	50
	AggregateType:	-1	
	AllowZeroLength:	True	

AppendOnly:	False
Attributes:	Variable Length; Updatable
CollatingOrder:	General
ColumnHidden:	False
ColumnOrder:	Default
ColumnWidth:	Default
DataUpdatable:	True
DisplayControl:	Text Box
GUID:	{guid {9C185040-3E47-44D4-B4A1-96698F823224}}
IMEMode:	0
IMESentenceMode:	3
OrdinalPosition:	4
Required:	False
SourceField:	Steril
SourceTable:	Medien
TextAlign:	General
UnicodeCompression:	True

Storagesite Long Integer 4

AggregateType:	-1
AllowValueListEdits:	False
AllowZeroLength:	False
AppendOnly:	False
Attributes:	Fixed Size; Updatable
BoundColumn:	1
CollatingOrder:	General
ColumnCount:	2
ColumnHeads:	False
ColumnHidden:	False
ColumnOrder:	Default
ColumnWidth:	2925
ColumnWidths:	0;1440
DataUpdatable:	True
DecimalPlaces:	Auto
DisplayControl:	Combo Box
LimitToList:	True
ListRows:	16
ListWidth:	1440twip
OrdinalPosition:	5
Required:	False
RowSource:	SELECT [Storagesite].[Storagesite_ID], [Storagesite].[Storagesite_Name] FROM [Storagesite] ORDER BY [Storagesite_Name];
RowSourceType:	Table/Query
ShowOnlyRowSourceValues:	False
SourceField:	Storagesite
SourceTable:	Medien
TextAlign:	General

Datum Date/Time 8

AggregateType:	-1
AllowZeroLength:	False
AppendOnly:	False
Attributes:	Fixed Size

CollatingOrder:	General	
DataUpdatable:	False	
GUID:	{guid {13AB639B-B6C3-42F5-B501-D2058E1D4289}}	
OrdinalPosition:	6	
Required:	False	
**Bezeichner**	OLE Object	N/A
AggregateType:	-1	
AllowZeroLength:	False	
AppendOnly:	False	
Attributes:	Variable Length; Updatable	
CollatingOrder:	General	
ColumnHidden:	False	
ColumnOrder:	Default	
ColumnWidth:	1965	
DataUpdatable:	True	
GUID:	{guid {A52CDB4F-F76D-43DE-B348-217A2AD512CB}}	
OrdinalPosition:	7	
Required:	False	
SourceField:	Bezeichner	
SourceTable:	Medien	
TextAlign:	General	
**Storagecondition**	Text	255
AggregateType:	-1	
AllowZeroLength:	True	
AppendOnly:	False	
Attributes:	Variable Length; Updatable	
CollatingOrder:	General	
ColumnHidden:	False	
ColumnOrder:	Default	
ColumnWidth:	2670	
DataUpdatable:	True	
DisplayControl:	Text Box	
IMEMode:	0	
IMESentenceMode:	3	
OrdinalPosition:	8	
Required:	False	
SourceField:	Storagecondition_Name	
SourceTable:	Storagecondition	
TextAlign:	General	
UnicodeCompression:	True	

Table Indexes

Name	Number of Fields
Medien ID	1
Clustered:	False
DistinctCount:	36
Foreign:	False
IgnoreNulls:	False
Name:	Medien ID
Primary:	False
Required:	False
Unique:	False

Fields:	
Medien ID	Ascending
**Medien ID old**	1
Clustered:	False
DistinctCount:	11
Foreign:	False
IgnoreNulls:	False
Name:	Medien ID old
Primary:	False
Required:	False
Unique:	False
Fields:	
Medien ID old	Ascending
**Medienname**	1
Clustered:	False
DistinctCount:	36
Foreign:	False
IgnoreNulls:	False
Name:	Medienname
Primary:	False
Required:	False
Unique:	True
Fields:	
Medienname	Ascending
**PrimaryKey**	1
Clustered:	False
DistinctCount:	36
Foreign:	False
IgnoreNulls:	False
Name:	PrimaryKey
Primary:	True
Required:	True
Unique:	True
Fields:	
Medien ID	Ascending
**PrimaryKey**	1
Clustered:	False
DistinctCount:	4
Foreign:	False
IgnoreNulls:	False
Name:	PrimaryKey
Primary:	True
Required:	True
Unique:	True
Fields:	
Storagecondition_ID	Ascending

User Permissions

admin	Delete; Read Permissions; Set Permissions; Change Owner, Read Definition; Write Definition; Read Data; Insert Data; Update Data; Delete Data

Group Permissions

Admins	Delete; Read Permissions; Set Permissions; Change Owner, Read Definition; Write Definition; Read Data; Insert Data; Update Data; Delete Data
Users	Delete; Read Permissions; Set Permissions; Change Owner, Read Definition; Write Definition; Read Data; Insert Data; Update Data; Delete Data

Properties

DateCreated:	27.01.2009 17:03:26	DefaultView:	2
DOL:	Long binary data	GUID:	{guid {24EAEB25-07B1-49C7-8AC4-461ED3C30724}}
LastUpdated:	27.01.2009 17:32:54	MaxRecords:	0
ODBCTimeout:	60	OrderByOn:	False
Orientation:	Left-to-Right	RecordLocks:	No Locks
RecordsAffected:	0	RecordsetType:	Dynaset
ReturnsRecords:	True	Type:	0
Updatable:	True		

SQL

```
SELECT Protokoll.Protokollname, Transformationsschritte.ZeitvonStart, Transformationsschritte.Prozess,
Transformationsschritte.Bemerkungen, Medien.Medienname, Media_Step.Remark, Medien.Bemerkung,
'14.08.2008'+[zeitvonstart] AS schedule, Media_Step.Bezeichner, Container.Name,
Transformationsschritte.Light, Transformationsschritte.Temperature, Protokoll.Genom
FROM (Protokoll INNER JOIN Transformationsschritte ON Protokoll.[Protokoll ID]=Transformationsschritte.Art)
INNER JOIN (Medien INNER JOIN ([Container] INNER JOIN Media_Step ON
Container.Container_id=Media_Step.Container) ON Medien.[Medien ID]=Media_Step.Media_id) ON
Transformationsschritte.ID=Media_Step.Trafostep_id
WHERE (((Protokoll.Protokollname) Like "Kartoffel*") AND ((Protokoll.Genom)="kern"));
```

Query Parameters

Name	Type
Medien.Bemerkung	Text
Container.Name	Text

Columns

Name	Type	Size
Protokollname	Text	50

AggregateType:	-1
AllowZeroLength:	True
AppendOnly:	False
Attributes:	Variable Length; Updatable
CollatingOrder:	General
ColumnHidden:	False
ColumnOrder:	Default
ColumnWidth:	4905
DataUpdatable:	True
Description:	Eindeutiger Name für das Protokoll
DisplayControl:	Text Box
GUID:	{guid {BA35B293-EC1A-433F-A9CA-F649675F83D2}}
IMEMode:	0
IMESentenceMode:	3
OrdinalPosition:	0
Required:	False
SourceField:	Protokollname
SourceTable:	Protokoll
TextAlign:	General
UnicodeCompression:	True

ZeitvonStart	Long Integer	4
AggregateType:	-1	
AllowZeroLength:	False	
AppendOnly:	False	
Attributes:	Fixed Size; Updatable	
CollatingOrder:	General	
ColumnHidden:	False	
ColumnOrder:	Default	
ColumnWidth:	1140	
DataUpdatable:	True	
DecimalPlaces:	Auto	
Description:	Zeit in Tagen seit dem Transformationsdatum	
DisplayControl:	Text Box	
GUID:	{guid {C998F549-C87B-453C-BBCE-2D8190464E13}}	
OrdinalPosition:	1	
Required:	False	
SourceField:	ZeitvonStart	
SourceTable:	Transformationsschritte	
TextAlign:	General	
Prozess	Text	50
AggregateType:	-1	
AllowZeroLength:	True	
AppendOnly:	False	
Attributes:	Variable Length; Updatable	
CollatingOrder:	General	
ColumnHidden:	False	
ColumnOrder:	Default	
ColumnWidth:	2910	
DataUpdatable:	True	
Description:	Arbeitsprozess	
DisplayControl:	Text Box	
GUID:	{guid {F145B5A2-C291-40CF-8D68-20B48F7A3039}}	
IMEMode:	0	
IMESentenceMode:	3	
OrdinalPosition:	2	
Required:	False	
SourceField:	Prozess	
SourceTable:	Transformationsschritte	
TextAlign:	General	
UnicodeCompression:	True	
Bemerkungen	Memo	N/A
AggregateType:	-1	
AllowZeroLength:	True	
AppendOnly:	False	
Attributes:	Variable Length; Updatable	
CollatingOrder:	General	
ColumnHidden:	False	
ColumnOrder:	Default	
ColumnWidth:	7740	
DataUpdatable:	True	
GUID:	{guid {5521D885-5AA9-4E67-90F5-02DF98AE027F}}	
IMEMode:	0	
IMESentenceMode:	3	
OrdinalPosition:	3	

	Required:	False
	SourceField:	Bemerkungen
	SourceTable:	Transformationsschritte
	TextAlign:	General
	TextFormat:	Plain Text
	UnicodeCompression:	True
Medienname	Text	50
	AggregateType:	-1
	AllowZeroLength:	True
	AppendOnly:	False
	Attributes:	Variable Length; Updatable
	CollatingOrder:	General
	ColumnHidden:	False
	ColumnOrder:	Default
	ColumnWidth:	3615
	DataUpdatable:	True
	DisplayControl:	Text Box
	GUID:	{guid {39A7EAC5-34A3-4BBD-A594-4E88A4399D39}}
	IMEMode:	0
	IMESentenceMode:	3
	OrdinalPosition:	4
	Required:	True
	SourceField:	Medienname
	SourceTable:	Medien
	TextAlign:	General
	UnicodeCompression:	True
Remark	Text	250
	AggregateType:	-1
	AllowZeroLength:	True
	AppendOnly:	False
	Attributes:	Variable Length; Updatable
	CollatingOrder:	General
	ColumnHidden:	False
	ColumnOrder:	Default
	ColumnWidth:	Default
	DataUpdatable:	True
	DisplayControl:	Text Box
	GUID:	{guid {FB0F8A63-FF1D-4AB8-B5D4-6C90F0B1FDE1}}
	IMEMode:	0
	IMESentenceMode:	3
	OrdinalPosition:	5
	Required:	False
	SourceField:	Remark
	SourceTable:	Media_Step
	TextAlign:	General
	UnicodeCompression:	True
Bemerkung	From	0
	AllowZeroLength:	False
	AppendOnly:	False
	Attributes:	Fixed Size
	CollatingOrder:	General
	DataUpdatable:	False
	OrdinalPosition:	6

Required:	False	

schedule	Double	8
AllowZeroLength:	False	
AppendOnly:	False	
Attributes:	Fixed Size	
CollatingOrder:	General	
DataUpdatable:	False	
GUID:	{guid {EF955046-0FA3-4795-8997-B2B69083DC2D}}	
OrdinalPosition:	7	
Required:	False	
SourceField:	ZeitvonStart	
SourceTable:	Transformationsschritte	

Bezeichner	Text	200
AggregateType:	-1	
AllowZeroLength:	True	
AppendOnly:	False	
Attributes:	Variable Length; Updatable	
CollatingOrder:	General	
ColumnHidden:	False	
ColumnOrder:	Default	
ColumnWidth:	Default	
DataUpdatable:	True	
DisplayControl:	Text Box	
GUID:	{guid {7E66055C-35F1-4926-BA22-D7E6221C143A}}	
IMEMode:	0	
IMESentenceMode:	3	
OrdinalPosition:	8	
Required:	False	
SourceField:	Bezeichner	
SourceTable:	Media_Step	
TextAlign:	General	
UnicodeCompression:	True	

Name	From	0
AllowZeroLength:	False	
AppendOnly:	False	
Attributes:	Fixed Size	
CollatingOrder:	General	
DataUpdatable:	False	
OrdinalPosition:	9	
Required:	False	

Light	Text	50
AggregateType:	-1	
AllowZeroLength:	True	
AppendOnly:	False	
Attributes:	Variable Length; Updatable	
CollatingOrder:	General	
ColumnHidden:	False	
ColumnOrder:	Default	
ColumnWidth:	Default	
DataUpdatable:	True	
Description:	Light intensity	
DisplayControl:	Text Box	

GUID:	{guid {02C88938-E1C3-4C76-B042-4F471E040019}}
IMEMode:	0
IMESentenceMode:	3
OrdinalPosition:	10
Required:	False
SourceField:	Light
SourceTable:	Transformationsschritte
TextAlign:	General
UnicodeCompression:	False

Temperature Long Integer 4

AggregateType:	-1
AllowZeroLength:	False
AppendOnly:	False
Attributes:	Fixed Size; Updatable
CollatingOrder:	General
ColumnHidden:	False
ColumnOrder:	Default
ColumnWidth:	Default
DataUpdatable:	True
DecimalPlaces:	Auto
Description:	Temperature in °C
DisplayControl:	Text Box
GUID:	{guid {4EC98804-4E45-4C1A-94AF-2CE1BA22CC38}}
OrdinalPosition:	11
Required:	False
SourceField:	Temperature
SourceTable:	Transformationsschritte
TextAlign:	General

Genom Text 50

AggregateType:	-1
AllowValueListEdits:	False
AllowZeroLength:	True
AppendOnly:	False
Attributes:	Variable Length; Updatable
BoundColumn:	1
CollatingOrder:	General
ColumnCount:	1
ColumnHeads:	False
ColumnHidden:	False
ColumnOrder:	Default
ColumnWidth:	Default
ColumnWidths:	1440
DataUpdatable:	True
DisplayControl:	Combo Box
GUID:	{guid {B3D19F8E-9909-407C-8A31-4B6AB22D3C16}}
IMEMode:	0
IMESentenceMode:	3
LimitToList:	False
ListRows:	8
ListWidth:	1440twip
OrdinalPosition:	12
Required:	False
RowSource:	"Kern";"Chloroplast";"Mitochondrien"
RowSourceType:	Value List

ShowOnlyRowSourceValues:	False
SourceField:	Genom
SourceTable:	Protokoll
TextAlign:	General
UnicodeCompression:	True

Table Indexes

Name		Number of Fields
PrimaryKey		1
	Clustered:	False
	DistinctCount:	3
	Foreign:	False
	IgnoreNulls:	False
	Name:	PrimaryKey
	Primary:	True
	Required:	True
	Unique:	True
	Fields:	
	Protokoll ID	Ascending
ProtokollArt		1
	Clustered:	False
	DistinctCount:	1
	Foreign:	False
	IgnoreNulls:	False
	Name:	ProtokollArt
	Primary:	False
	Required:	False
	Unique:	False
	Fields:	
	Art	Ascending
Old_ID		1
	Clustered:	False
	DistinctCount:	4
	Foreign:	False
	IgnoreNulls:	False
	Name:	Old_ID
	Primary:	False
	Required:	False
	Unique:	False
	Fields:	
	Old_ID	Ascending
PrimaryKey		1
	Clustered:	False
	DistinctCount:	9
	Foreign:	False
	IgnoreNulls:	False
	Name:	PrimaryKey
	Primary:	True
	Required:	True
	Unique:	True
	Fields:	
	ID	Ascending

Medien ID		1
	Clustered:	False
	DistinctCount:	36
	Foreign:	False
	IgnoreNulls:	False
	Name:	Medien ID
	Primary:	False
	Required:	False
	Unique:	False
	Fields:	
	Medien ID	Ascending
Medien ID old		1
	Clustered:	False
	DistinctCount:	11
	Foreign:	False
	IgnoreNulls:	False
	Name:	Medien ID old
	Primary:	False
	Required:	False
	Unique:	False
	Fields:	
	Medien ID old	Ascending
Medienname		1
	Clustered:	False
	DistinctCount:	36
	Foreign:	False
	IgnoreNulls:	False
	Name:	Medienname
	Primary:	False
	Required:	False
	Unique:	True
	Fields:	
	Medienname	Ascending
PrimaryKey		1
	Clustered:	False
	DistinctCount:	36
	Foreign:	False
	IgnoreNulls:	False
	Name:	PrimaryKey
	Primary:	True
	Required:	True
	Unique:	True
	Fields:	
	Medien ID	Ascending
Media_id		1
	Clustered:	False
	DistinctCount:	7
	Foreign:	False
	IgnoreNulls:	False
	Name:	Media_id
	Primary:	False
	Required:	False
	Unique:	False

	Fields:	
	Media_id	Ascending
Medien_Schritt_id		1
	Clustered:	False
	DistinctCount:	11
	Foreign:	False
	IgnoreNulls:	False
	Name:	Medien_Schritt_id
	Primary:	False
	Required:	False
	Unique:	False
	Fields:	
	Medien_Schritt_id	Ascending
MedienMedia_Step		1
	Clustered:	False
	DistinctCount:	7
	Foreign:	True
	IgnoreNulls:	False
	Name:	MedienMedia_Step
	Primary:	False
	Required:	False
	Unique:	False
	Fields:	
	Media_id	Ascending
PrimaryKey		1
	Clustered:	False
	DistinctCount:	11
	Foreign:	False
	IgnoreNulls:	False
	Name:	PrimaryKey
	Primary:	True
	Required:	True
	Unique:	True
	Fields:	
	Medien_Schritt_id	Ascending
TransformationsschritteMedia_Step		1
	Clustered:	False
	DistinctCount:	9
	Foreign:	True
	IgnoreNulls:	False
	Name:	TransformationsschritteMedia_Step
	Primary:	False
	Required:	False
	Unique:	False
	Fields:	
	Trafostep_id	Ascending
Old_ID		1
	Clustered:	False
	DistinctCount:	4
	Foreign:	False
	IgnoreNulls:	False
	Name:	Old_ID
	Primary:	False

	Required:	False
	Unique:	False
	Fields:	
	Old_ID	Ascending
PrimaryKey		1
	Clustered:	False
	DistinctCount:	9
	Foreign:	False
	IgnoreNulls:	False
	Name:	PrimaryKey
	Primary:	True
	Required:	True
	Unique:	True
	Fields:	
	ID	Ascending
Media_id		1
	Clustered:	False
	DistinctCount:	7
	Foreign:	False
	IgnoreNulls:	False
	Name:	Media_id
	Primary:	False
	Required:	False
	Unique:	False
	Fields:	
	Media_id	Ascending
Medien_Schritt_id		1
	Clustered:	False
	DistinctCount:	11
	Foreign:	False
	IgnoreNulls:	False
	Name:	Medien_Schritt_id
	Primary:	False
	Required:	False
	Unique:	False
	Fields:	
	Medien_Schritt_id	Ascending
MedienMedia_Step		1
	Clustered:	False
	DistinctCount:	7
	Foreign:	True
	IgnoreNulls:	False
	Name:	MedienMedia_Step
	Primary:	False
	Required:	False
	Unique:	False
	Fields:	
	Media_id	Ascending
PrimaryKey		1
	Clustered:	False
	DistinctCount:	11
	Foreign:	False
	IgnoreNulls:	False

Name:	PrimaryKey
Primary:	True
Required:	True
Unique:	True
Fields:	
Medien_Schritt_id	Ascending
**TransformationsschritteMedia_Step**	1
Clustered:	False
DistinctCount:	9
Foreign:	True
IgnoreNulls:	False
Name:	TransformationsschritteMedia_Step
Primary:	False
Required:	False
Unique:	False
Fields:	
Trafostep_id	Ascending
**Old_ID**	1
Clustered:	False
DistinctCount:	4
Foreign:	False
IgnoreNulls:	False
Name:	Old_ID
Primary:	False
Required:	False
Unique:	False
Fields:	
Old_ID	Ascending
**PrimaryKey**	1
Clustered:	False
DistinctCount:	9
Foreign:	False
IgnoreNulls:	False
Name:	PrimaryKey
Primary:	True
Required:	True
Unique:	True
Fields:	
ID	Ascending
**PrimaryKey**	1
Clustered:	False
DistinctCount:	3
Foreign:	False
IgnoreNulls:	False
Name:	PrimaryKey
Primary:	True
Required:	True
Unique:	True
Fields:	
Protokoll ID	Ascending
**ProtokollArt**	1
Clustered:	False
DistinctCount:	1

Foreign:	False
IgnoreNulls:	False
Name:	ProtokollArt
Primary:	False
Required:	False
Unique:	False
Fields:	
Art	Ascending

User Permissions

admin	Delete; Read Permissions; Set Permissions; Change Owner, Read Definition; Write Definition; Read Data; Insert Data; Update Data; Delete Data

Group Permissions

Admins	Delete; Read Permissions; Set Permissions; Change Owner, Read Definition; Write Definition; Read Data; Insert Data; Update Data; Delete Data
Users	Delete; Read Permissions; Set Permissions; Change Owner, Read Definition; Write Definition; Read Data; Insert Data; Update Data; Delete Data

Properties

DateCreated:	18.12.2009 10:52:36	DefaultView:	2
DOL:	Long binary data	FilterOnLoad:	False
GUID:	{guid {3F77F8C5-1B0D-45F8-889F-861FE003498E}}	LastUpdated:	18.12.2009 11:00:05
MaxRecords:	0	ODBCTimeout:	60
OrderByOn:	False	OrderByOnLoad:	True
Orientation:	Left-to-Right	RecordLocks:	No Locks
RecordsAffected:	0	RecordsetType:	Dynaset
ReturnsRecords:	True	TotalsRow:	False
Type:	0	Updatable:	True

SQL

```
SELECT Medien.[Medien ID], Medien.Medienname, Medien.Bezeichner, Barcode128.Barcode
FROM Medien INNER JOIN Barcode128 ON Medien.[Medien ID] = Barcode128.Id
WHERE (((Medien.[Medien ID])=[Enter Media ID]));
```

Query Parameters

Name	Type
[Enter Media ID]	Text

Columns

Name	Type	Size
Medien ID	Long Integer	4

AggregateType:	-1
AllowZeroLength:	False
AppendOnly:	False
Attributes:	Fixed Size; Auto-Increment; Updatable
CollatingOrder:	General
ColumnHidden:	False
ColumnOrder:	1
ColumnWidth:	Default
DataUpdatable:	True
GUID:	{guid {FBE4AC72-E216-4CE4-ACB3-246BABE85369}}
OrdinalPosition:	0
Required:	False
SourceField:	Medien ID
SourceTable:	Medien
TextAlign:	General

Name	Type	Size
Medienname	Text	50

AggregateType:	-1
AllowZeroLength:	True
AppendOnly:	False
Attributes:	Variable Length; Updatable
CollatingOrder:	General
ColumnHidden:	False
ColumnOrder:	Default
ColumnWidth:	3615

DataUpdatable:	True	
DisplayControl:	Text Box	
GUID:	{guid {39A7EAC5-34A3-4BBD-A594-4E88A4399D39}}	
IMEMode:	0	
IMESentenceMode:	3	
OrdinalPosition:	1	
Required:	True	
SourceField:	Medienname	
SourceTable:	Medien	
TextAlign:	General	
UnicodeCompression:	True	

Bezeichner	OLE Object	N/A
AggregateType:	-1	
AllowZeroLength:	False	
AppendOnly:	False	
Attributes:	Variable Length; Updatable	
CollatingOrder:	General	
ColumnHidden:	False	
ColumnOrder:	Default	
ColumnWidth:	1965	
DataUpdatable:	True	
GUID:	{guid {A52CDB4F-F76D-43DE-B348-217A2AD512CB}}	
OrdinalPosition:	2	
Required:	False	
SourceField:	Bezeichner	
SourceTable:	Medien	
TextAlign:	General	

Barcode	Text	255
AggregateType:	-1	
AllowZeroLength:	True	
AppendOnly:	False	
Attributes:	Variable Length; Updatable	
CollatingOrder:	General	
ColumnHidden:	False	
ColumnOrder:	Default	
ColumnWidth:	Default	
DataUpdatable:	True	
DisplayControl:	Text Box	
IMEMode:	0	
IMESentenceMode:	3	
OrdinalPosition:	3	
Required:	False	
SourceField:	Barcode	
SourceTable:	Barcode128	
TextAlign:	General	
UnicodeCompression:	False	

Table Indexes

Name	Number of Fields
Medien ID	1
Clustered:	False
DistinctCount:	36

	Foreign:	False
	IgnoreNulls:	False
	Name:	Medien ID
	Primary:	False
	Required:	False
	Unique:	False
	Fields:	
	Medien ID	Ascending
Medien ID old		1
	Clustered:	False
	DistinctCount:	11
	Foreign:	False
	IgnoreNulls:	False
	Name:	Medien ID old
	Primary:	False
	Required:	False
	Unique:	False
	Fields:	
	Medien ID old	Ascending
Medienname		1
	Clustered:	False
	DistinctCount:	36
	Foreign:	False
	IgnoreNulls:	False
	Name:	Medienname
	Primary:	False
	Required:	False
	Unique:	True
	Fields:	
	Medienname	Ascending
PrimaryKey		1
	Clustered:	False
	DistinctCount:	36
	Foreign:	False
	IgnoreNulls:	False
	Name:	PrimaryKey
	Primary:	True
	Required:	True
	Unique:	True
	Fields:	
	Medien ID	Ascending
Barcode		1
	Clustered:	False
	DistinctCount:	7483
	Foreign:	False
	IgnoreNulls:	False
	Name:	Barcode
	Primary:	False
	Required:	False
	Unique:	False
	Fields:	
	Barcode	Ascending
PrimaryKey		1

Clustered:	False
DistinctCount:	8001
Foreign:	False
IgnoreNulls:	False
Name:	PrimaryKey
Primary:	True
Required:	True
Unique:	True
Fields:	
Id	Ascending

User Permissions

admin	Delete; Read Permissions; Set Permissions; Change Owner, Read Definition; Write Definition; Read Data; Insert Data; Update Data; Delete Data

Group Permissions

Admins	Delete; Read Permissions; Set Permissions; Change Owner, Read Definition; Write Definition; Read Data; Insert Data; Update Data; Delete Data
Users	Delete; Read Permissions; Set Permissions; Change Owner, Read Definition; Write Definition; Read Data; Insert Data; Update Data; Delete Data

Properties

DateCreated:	06.01.2009 14:39:40	DefaultView:	2
DisplayViewsOnSharePointSit	1	DOL:	Long binary data
FilterOnLoad:	False	GUID:	{guid {477786B5-02FF-4C86-BACD-AAB4A1BF728B}}
HideNewField:	False	LastUpdated:	26.03.2009 14:52:53
MaxRecords:	0	NameMap:	Long binary data
ODBCTimeout:	60	OrderByOn:	False
OrderByOnLoad:	True	Orientation:	Left-to-Right
RecordLocks:	No Locks	RecordsAffected:	0
RecordsetType:	Dynaset	ReturnsRecords:	True
TotalsRow:	False	Type:	0
Updatable:	True		

SQL

```
SELECT Medien.[Medien ID], Medien.Medienname, Medien.Bezeichner
FROM Medien
WHERE (((Medien.[Medien ID])=[Mediennummer]));
```

Query Parameters

Name	Type
[Mediennummer]	Text

Columns

Name	Type	Size
Medien ID	Long Integer	4

AggregateType:	-1
AllowZeroLength:	False
AppendOnly:	False
Attributes:	Fixed Size; Auto-Increment; Updatable
CollatingOrder:	General
ColumnHidden:	False
ColumnOrder:	1
ColumnWidth:	Default
DataUpdatable:	True
GUID:	{guid {FBE4AC72-E216-4CE4-ACB3-246BABE85369}}
OrdinalPosition:	0
Required:	False
SourceField:	Medien ID
SourceTable:	Medien
TextAlign:	General

Name	Type	Size
Medienname	Text	50

AggregateType:	-1
AllowZeroLength:	True
AppendOnly:	False
Attributes:	Variable Length; Updatable
CollatingOrder:	General
ColumnHidden:	False

ColumnOrder:	Default
ColumnWidth:	3615
DataUpdatable:	True
DisplayControl:	Text Box
GUID:	{guid {39A7EAC5-34A3-4BBD-A594-4E88A4399D39}}
IMEMode:	0
IMESentenceMode:	3
OrdinalPosition:	1
Required:	True
SourceField:	Medienname
SourceTable:	Medien
TextAlign:	General
UnicodeCompression:	True

Bezeichner OLE Object N/A

AggregateType:	-1
AllowZeroLength:	False
AppendOnly:	False
Attributes:	Variable Length; Updatable
CollatingOrder:	General
ColumnHidden:	False
ColumnOrder:	Default
ColumnWidth:	1965
DataUpdatable:	True
GUID:	{guid {A52CDB4F-F76D-43DE-B348-217A2AD512CB}}
OrdinalPosition:	2
Required:	False
SourceField:	Bezeichner
SourceTable:	Medien
TextAlign:	General

Table Indexes

Name	Number of Fields
Medien ID	1
Clustered:	False
DistinctCount:	36
Foreign:	False
IgnoreNulls:	False
Name:	Medien ID
Primary:	False
Required:	False
Unique:	False
Fields:	
Medien ID	Ascending
Medien ID old	1
Clustered:	False
DistinctCount:	11
Foreign:	False
IgnoreNulls:	False
Name:	Medien ID old
Primary:	False
Required:	False
Unique:	False

Fields:	
Medien ID old	Ascending
Medienname	1
Clustered:	False
DistinctCount:	36
Foreign:	False
IgnoreNulls:	False
Name:	Medienname
Primary:	False
Required:	False
Unique:	True
Fields:	
Medienname	Ascending
PrimaryKey	1
Clustered:	False
DistinctCount:	36
Foreign:	False
IgnoreNulls:	False
Name:	PrimaryKey
Primary:	True
Required:	True
Unique:	True
Fields:	
Medien ID	Ascending

User Permissions

admin	Delete; Read Permissions; Set Permissions; Change Owner, Read Definition; Write Definition; Read Data; Insert Data; Update Data; Delete Data

Group Permissions

Admins	Delete; Read Permissions; Set Permissions; Change Owner, Read Definition; Write Definition; Read Data; Insert Data; Update Data; Delete Data
Users	Delete; Read Permissions; Set Permissions; Change Owner, Read Definition; Write Definition; Read Data; Insert Data; Update Data; Delete Data

Properties

DateCreated:	27.01.2009 17:14:29	DefaultView:	2
DisplayViewsOnSharePointSit	1	DOL:	Long binary data
FilterOnLoad:	False	GUID:	{guid {9A2A01ED-61A1-475B-8F78-BB4C697CD6DD}}
HideNewField:	False	LastUpdated:	27.02.2009 07:16:09
MaxRecords:	0	NameMap:	Long binary data
ODBCTimeout:	60	OrderByOn:	False
OrderByOnLoad:	True	Orientation:	Left-to-Right
RecordLocks:	Edited Record	RecordsAffected:	0
RecordsetType:	Dynaset	ReturnsRecords:	True
TotalsRow:	False	Type:	0
Updatable:	True	UseTransaction:	True

SQL

```
SELECT Medien.Storagesite, Medien.[Medien ID], Medien.Medienname, Medien.Storagecondition
FROM Medien
WHERE (((Medien.Medienname) Not Like "*obsolet*"))
ORDER BY Medien.Storagesite;
```

Columns

Name	Type	Size
Storagesite	Long Integer	4

AggregateType:	-1
AllowValueListEdits:	False
AllowZeroLength:	False
AppendOnly:	False
Attributes:	Fixed Size; Updatable
BoundColumn:	1
CollatingOrder:	General
ColumnCount:	2
ColumnHeads:	False
ColumnHidden:	False
ColumnOrder:	Default
ColumnWidth:	5730
ColumnWidths:	0;1440
DataUpdatable:	True
DecimalPlaces:	Auto
DisplayControl:	Combo Box
LimitToList:	True
ListRows:	16
ListWidth:	1440twip
OrdinalPosition:	0
Required:	False
RowSource:	SELECT [Storagesite].[Storagesite_ID], [Storagesite].[Storagesite_Name] FROM [Storagesite] ORDER BY [Storagesite_Name];
RowSourceType:	Table/Query
ShowOnlyRowSourceValues:	False
SourceField:	Storagesite
SourceTable:	Medien

TextAlign: General

Medien ID Long Integer 4

AggregateType: -1
AllowZeroLength: False
AppendOnly: False
Attributes: Fixed Size; Auto-Increment; Updatable
CollatingOrder: General
ColumnHidden: False
ColumnOrder: 1
ColumnWidth: Default
DataUpdatable: True
GUID: {guid {FBE4AC72-E216-4CE4-ACB3-246BABE85369}}
OrdinalPosition: 1
Required: False
SourceField: Medien ID
SourceTable: Medien
TextAlign: General

Medienname Text 50

AggregateType: -1
AllowZeroLength: True
AppendOnly: False
Attributes: Variable Length; Updatable
CollatingOrder: General
ColumnHidden: False
ColumnOrder: Default
ColumnWidth: 3615
DataUpdatable: True
DisplayControl: Text Box
GUID: {guid {39A7EAC5-34A3-4BBD-A594-4E88A4399D39}}
IMEMode: 0
IMESentenceMode: 3
OrdinalPosition: 2
Required: True
SourceField: Medienname
SourceTable: Medien
TextAlign: General
UnicodeCompression: True

Storagecondition Long Integer 4

AggregateType: -1
AllowValueListEdits: False
AllowZeroLength: False
AppendOnly: False
Attributes: Fixed Size; Updatable
BoundColumn: 1
CollatingOrder: General
ColumnCount: 2
ColumnHeads: False
ColumnHidden: False
ColumnOrder: Default
ColumnWidth: 2070
ColumnWidths: 0;1440
DataUpdatable: True
DecimalPlaces: Auto

DisplayControl:	Combo Box
LimitToList:	True
ListRows:	8
ListWidth:	1440twip
OrdinalPosition:	3
Required:	False
RowSource:	SELECT [Storagecondition].[Storagecondition_ID], [Storagecondition].[Storagecondition_Name] FROM [Storagecondition] ORDER BY [Storagecondition_Name];
RowSourceType:	Table/Query
ShowOnlyRowSourceValues:	False
SourceField:	Storagecondition
SourceTable:	Medien
TextAlign:	General

Table Indexes

Name		Number of Fields
Medien ID		1
	Clustered:	False
	DistinctCount:	36
	Foreign:	False
	IgnoreNulls:	False
	Name:	Medien ID
	Primary:	False
	Required:	False
	Unique:	False
	Fields:	
	Medien ID	Ascending
Medien ID old		1
	Clustered:	False
	DistinctCount:	11
	Foreign:	False
	IgnoreNulls:	False
	Name:	Medien ID old
	Primary:	False
	Required:	False
	Unique:	False
	Fields:	
	Medien ID old	Ascending
Medienname		1
	Clustered:	False
	DistinctCount:	36
	Foreign:	False
	IgnoreNulls:	False
	Name:	Medienname
	Primary:	False
	Required:	False
	Unique:	True
	Fields:	
	Medienname	Ascending
PrimaryKey		1
	Clustered:	False
	DistinctCount:	36

Foreign:	False
IgnoreNulls:	False
Name:	PrimaryKey
Primary:	True
Required:	True
Unique:	True
Fields:	
Medien ID	Ascending

User Permissions

admin	Delete; Read Permissions; Set Permissions; Change Owner, Read Definition; Write Definition; Read Data; Insert Data; Update Data; Delete Data

Group Permissions

Admins	Delete; Read Permissions; Set Permissions; Change Owner, Read Definition; Write Definition; Read Data; Insert Data; Update Data; Delete Data
Users	Delete; Read Permissions; Set Permissions; Change Owner, Read Definition; Write Definition; Read Data; Insert Data; Update Data; Delete Data

Properties

DateCreated:	15.03.2007 07:15:39	DefaultView:	2
DisplayViewsOnSharePointSit	1	DOL:	Long binary data
FilterOnLoad:	False	GUID:	{guid {9D11ACD0-147B-4609-B1EC-6A407F7FAD0F}}
HideNewField:	False	LastUpdated:	12.01.2009 08:26:30
MaxRecords:	0	NameMap:	Long binary data
ODBCTimeout:	60	OrderByOn:	False
OrderByOnLoad:	True	Orientation:	Left-to-Right
RecordLocks:	No Locks	RecordsAffected:	0
RecordsetType:	Dynaset	ReturnsRecords:	True
TotalsRow:	False	Type:	0
Updatable:	True		

SQL

```
SELECT Medienzusammensetzung.Medien, Medienzusammensetzung.Menge,
Medienzusammensetzung.Protokollname, Medienzusammensetzung.Stock
FROM Medienzusammensetzung;
```

Columns

Name	Type	Size
Medien	Long Integer	4

AggregateType:	-1
AllowValueListEdits:	False
AllowZeroLength:	False
AppendOnly:	False
Attributes:	Fixed Size; Updatable
BoundColumn:	1
CollatingOrder:	General
ColumnCount:	2
ColumnHeads:	False
ColumnHidden:	False
ColumnOrder:	Default
ColumnWidth:	3660
ColumnWidths:	0;3258
DataUpdatable:	True
DecimalPlaces:	Auto
DisplayControl:	Combo Box
GUID:	{guid {568B9D25-BA8B-4774-B214-F36020A2E255}}
LimitToList:	True
ListRows:	8
ListWidth:	3255twip
OrdinalPosition:	0
Required:	False
RowSource:	SELECT Medien.[Medien ID] FROM Medien;
RowSourceType:	Table/Query
ShowOnlyRowSourceValues:	False
SourceField:	Medien
SourceTable:	Medienzusammensetzung
TextAlign:	General

Menge	Double	8
AggregateType:	-1	
AllowZeroLength:	False	
AppendOnly:	False	
Attributes:	Fixed Size; Updatable	
CollatingOrder:	General	
ColumnHidden:	False	
ColumnOrder:	Default	
ColumnWidth:	1140	
DataUpdatable:	True	
DecimalPlaces:	Auto	
DisplayControl:	Text Box	
GUID:	{guid {C238FB2E-3901-4526-9015-8653957C8B6E}}	
OrdinalPosition:	1	
Required:	False	
SourceField:	Menge	
SourceTable:	Medienzusammensetzung	
TextAlign:	General	
Protokollname	Text	50
AggregateType:	-1	
AllowZeroLength:	True	
AppendOnly:	False	
Attributes:	Variable Length; Updatable	
CollatingOrder:	General	
ColumnHidden:	False	
ColumnOrder:	Default	
ColumnWidth:	3090	
DataUpdatable:	True	
DisplayControl:	Text Box	
GUID:	{guid {52E3AF2F-012F-416E-97E9-2AE3605BA211}}	
IMEMode:	0	
IMESentenceMode:	3	
OrdinalPosition:	2	
Required:	False	
SourceField:	Protokollname	
SourceTable:	Medienzusammensetzung	
TextAlign:	General	
UnicodeCompression:	True	
Stock	Long Integer	4
AggregateType:	-1	
AllowValueListEdits:	False	
AllowZeroLength:	False	
AppendOnly:	False	
Attributes:	Fixed Size; Updatable	
BoundColumn:	1	
CollatingOrder:	General	
ColumnCount:	2	
ColumnHeads:	False	
ColumnHidden:	False	
ColumnOrder:	Default	
ColumnWidth:	2580	
ColumnWidths:	0;2730	
DataUpdatable:	True	
DecimalPlaces:	Auto	

DisplayControl:	Combo Box
GUID:	{guid {A8A0F210-4ECF-44E0-9B28-8C90ACC20154}}
LimitToList:	True
ListRows:	8
ListWidth:	2730twip
OrdinalPosition:	3
Required:	False
RowSource:	SELECT Stocks.Stock_id, Stocks.Stock_name FROM Stocks;
RowSourceType:	Table/Query
ShowOnlyRowSourceValues:	False
SourceField:	Stock
SourceTable:	Medienzusammensetzung
TextAlign:	General

Table Indexes

Name	Number of Fields
Medien ID	1
Clustered:	False
DistinctCount:	54
Foreign:	False
IgnoreNulls:	False
Name:	Medien ID
Primary:	False
Required:	False
Unique:	False
Fields:	
Medien_Zusammen_ ID	Ascending
MedienMedienzusammensetzung	1
Clustered:	False
DistinctCount:	21
Foreign:	True
IgnoreNulls:	False
Name:	MedienMedienzusammensetzung
Primary:	False
Required:	False
Unique:	False
Fields:	
Medien	Ascending
PrimaryKey	1
Clustered:	False
DistinctCount:	54
Foreign:	False
IgnoreNulls:	False
Name:	PrimaryKey
Primary:	True
Required:	True
Unique:	True
Fields:	
Medien_Zusammen_ ID	Ascending

User Permissions

admin	Delete; Read Permissions; Set Permissions; Change Owner, Read Definition; Write Definition; Read Data; Insert Data; Update Data; Delete Data

Group Permissions

Admins	Delete; Read Permissions; Set Permissions; Change Owner, Read Definition; Write Definition; Read Data; Insert Data; Update Data; Delete Data
Users	Delete; Read Permissions; Set Permissions; Change Owner, Read Definition; Write Definition; Read Data; Insert Data; Update Data; Delete Data

Properties

DateCreated:	29.09.2005 12:47:38	DefaultView:	2
DOL:	Long binary data	GUID:	{guid {C03FDFF1-975F-41C7-BED6-970FDA3B8F33}}
LastUpdated:	10.09.2008 08:04:00	MaxRecords:	0
ODBCTimeout:	60	OrderBy:	Lookup_Medien.Medienname DESC
OrderByOn:	True	Orientation:	Left-to-Right
RecordLocks:	No Locks	RecordsAffected:	0
RecordsetType:	Dynaset	ReturnsRecords:	True
Type:	0	Updatable:	True

SQL

```
SELECT Medienzusammensetzung.Protokollname, Medienzusammensetzung.Medien,
Medienzusammensetzung.Stock, Medienzusammensetzung.Menge, Medienzusammensetzung.Mengeneinheit,
Medien.Medienname
FROM Medien INNER JOIN Medienzusammensetzung ON Medien.[Medien
ID]=Medienzusammensetzung.Medien
WHERE (((Medienzusammensetzung.Protokollname)=[bitte Protokollnamen eingeben]));
```

Query Parameters

Name	Type
[bitte Protokollnamen eingeben]	Text

Columns

Name	Type	Size
Protokollname	Text	50

AggregateType:	-1
AllowZeroLength:	True
AppendOnly:	False
Attributes:	Variable Length; Updatable
CollatingOrder:	General
ColumnHidden:	False
ColumnOrder:	Default
ColumnWidth:	3090
DataUpdatable:	True
DisplayControl:	Text Box
GUID:	{guid {52E3AF2F-012F-416E-97E9-2AE3605BA211}}
IMEMode:	0
IMESentenceMode:	3
OrdinalPosition:	0
Required:	False
SourceField:	Protokollname
SourceTable:	Medienzusammensetzung
TextAlign:	General
UnicodeCompression:	True

Name	Type	Size
Medien	Long Integer	4

AggregateType:	-1

AllowValueListEdits:	False
AllowZeroLength:	False
AppendOnly:	False
Attributes:	Fixed Size; Updatable
BoundColumn:	1
CollatingOrder:	General
ColumnCount:	2
ColumnHeads:	False
ColumnHidden:	False
ColumnOrder:	Default
ColumnWidth:	3660
ColumnWidths:	0;3258
DataUpdatable:	True
DecimalPlaces:	Auto
DisplayControl:	Combo Box
GUID:	{guid {568B9D25-BA8B-4774-B214-F36020A2E255}}
LimitToList:	True
ListRows:	8
ListWidth:	3255twip
OrdinalPosition:	1
Required:	False
RowSource:	SELECT Medien.[Medien ID] FROM Medien;
RowSourceType:	Table/Query
ShowOnlyRowSourceValues:	False
SourceField:	Medien
SourceTable:	Medienzusammensetzung
TextAlign:	General

Stock Long Integer 4

AggregateType:	-1
AllowValueListEdits:	False
AllowZeroLength:	False
AppendOnly:	False
Attributes:	Fixed Size; Updatable
BoundColumn:	1
CollatingOrder:	General
ColumnCount:	2
ColumnHeads:	False
ColumnHidden:	False
ColumnOrder:	Default
ColumnWidth:	2580
ColumnWidths:	0;2730
DataUpdatable:	True
DecimalPlaces:	Auto
DisplayControl:	Combo Box
GUID:	{guid {A8A0F210-4ECF-44E0-9B28-8C90ACC20154}}
LimitToList:	True
ListRows:	8
ListWidth:	2730twip
OrdinalPosition:	2
Required:	False
RowSource:	SELECT Stocks.Stock_id, Stocks.Stock_name FROM Stocks;
RowSourceType:	Table/Query
ShowOnlyRowSourceValues:	False
SourceField:	Stock
SourceTable:	Medienzusammensetzung

TextAlign: General

Menge Double 8

AggregateType: -1
AllowZeroLength: False
AppendOnly: False
Attributes: Fixed Size; Updatable
CollatingOrder: General
ColumnHidden: False
ColumnOrder: Default
ColumnWidth: 1140
DataUpdatable: True
DecimalPlaces: Auto
DisplayControl: Text Box
GUID: {guid {C238FB2E-3901-4526-9015-8653957C8B6E}}
OrdinalPosition: 3
Required: False
SourceField: Menge
SourceTable: Medienzusammensetzung
TextAlign: General

Mengeneinheit Text 50

AggregateType: -1
AllowValueListEdits: False
AllowZeroLength: True
AppendOnly: False
Attributes: Variable Length; Updatable
BoundColumn: 1
CollatingOrder: General
ColumnCount: 1
ColumnHeads: False
ColumnHidden: False
ColumnOrder: Default
ColumnWidth: Default
ColumnWidths: 1440
DataUpdatable: True
DisplayControl: Combo Box
GUID: {guid {DB67B6DD-8A3A-4595-9033-E79B08EA7937}}
IMEMode: 0
IMESentenceMode: 3
LimitToList: False
ListRows: 8
ListWidth: 1440twip
OrdinalPosition: 4
Required: False
RowSource: "ml/l";"ul/l";"g/l";"mg/, ";"ul/900 ml";"g/900ml"
RowSourceType: Value List
ShowOnlyRowSourceValues: False
SourceField: Mengeneinheit
SourceTable: Medienzusammensetzung
TextAlign: General
UnicodeCompression: True

Medienname Text 50

AggregateType: -1
AllowZeroLength: True

AppendOnly:	False
Attributes:	Variable Length; Updatable
CollatingOrder:	General
ColumnHidden:	False
ColumnOrder:	Default
ColumnWidth:	3615
DataUpdatable:	True
DisplayControl:	Text Box
GUID:	{guid {39A7EAC5-34A3-4BBD-A594-4E88A4399D39}}
IMEMode:	0
IMESentenceMode:	3
OrdinalPosition:	5
Required:	True
SourceField:	Medienname
SourceTable:	Medien
TextAlign:	General
UnicodeCompression:	True

Table Indexes

Name	Number of Fields
Medien ID	1
Clustered:	False
DistinctCount:	54
Foreign:	False
IgnoreNulls:	False
Name:	Medien ID
Primary:	False
Required:	False
Unique:	False
Fields:	
Medien_Zusammen_ ID	Ascending
MedienMedienzusammensetzung	1
Clustered:	False
DistinctCount:	21
Foreign:	True
IgnoreNulls:	False
Name:	MedienMedienzusammensetzung
Primary:	False
Required:	False
Unique:	False
Fields:	
Medien	Ascending
PrimaryKey	1
Clustered:	False
DistinctCount:	54
Foreign:	False
IgnoreNulls:	False
Name:	PrimaryKey
Primary:	True
Required:	True
Unique:	True

	Fields:	
	Medien_Zusammen_ ID	Ascending
Medien ID		1
	Clustered:	False
	DistinctCount:	36
	Foreign:	False
	IgnoreNulls:	False
	Name:	Medien ID
	Primary:	False
	Required:	False
	Unique:	False
	Fields:	
	Medien ID	Ascending
Medien ID old		1
	Clustered:	False
	DistinctCount:	11
	Foreign:	False
	IgnoreNulls:	False
	Name:	Medien ID old
	Primary:	False
	Required:	False
	Unique:	False
	Fields:	
	Medien ID old	Ascending
Medienname		1
	Clustered:	False
	DistinctCount:	36
	Foreign:	False
	IgnoreNulls:	False
	Name:	Medienname
	Primary:	False
	Required:	False
	Unique:	True
	Fields:	
	Medienname	Ascending
PrimaryKey		1
	Clustered:	False
	DistinctCount:	36
	Foreign:	False
	IgnoreNulls:	False
	Name:	PrimaryKey
	Primary:	True
	Required:	True
	Unique:	True
	Fields:	
	Medien ID	Ascending

User Permissions

admin	Delete; Read Permissions; Set Permissions; Change Owner, Read Definition; Write Definition; Read Data; Insert Data; Update Data; Delete Data

Group Permissions

Admins	Delete; Read Permissions; Set Permissions; Change Owner, Read Definition; Write Definition; Read Data; Insert Data; Update Data; Delete Data
Users	Delete; Read Permissions; Set Permissions; Change Owner, Read Definition; Write Definition; Read Data; Insert Data; Update Data; Delete Data

Properties

DateCreated:	12.09.2006 14:16:05	DefaultView:	2
DOL:	Long binary data	Filter:	(([Pflanzentransformation Abfrage].Operator="Hojka"))
FilterOnLoad:	False	GUID:	{guid {83588B3C-336C-4DD1-9BE7-30BCEAA272B8}}
LastUpdated:	17.06.2009 08:17:30	MaxRecords:	0
ODBCTimeout:	60	OrderBy:	[Pflanzentransformation Abfrage].[ID], [Pflanzentransformation Abfrage].Transformationsdatum
OrderByOn:	True	OrderByOnLoad:	True
Orientation:	Left-to-Right	RecordLocks:	No Locks
RecordsAffected:	0	RecordsetType:	Dynaset
ReturnsRecords:	True	TotalsRow:	False
Type:	0	Updatable:	True

SQL

```
SELECT [Abfrage: Transformationsdatenblatt alle].ID, [Abfrage: Transformationsdatenblatt alle].[GVO Nummer], [Abfrage: Transformationsdatenblatt alle].Konstruktname, [Abfrage: Transformationsdatenblatt alle].Operator, [Abfrage: Transformationsdatenblatt alle].[Resistenz Pflanze].Resistenz, [Abfrage: Transformationsdatenblatt alle].Spezies, [Abfrage: Transformationsdatenblatt alle].Varietät, [Abfrage: Transformationsdatenblatt alle].[GMO Nummer], [Abfrage: Transformationsdatenblatt alle].Linie, [Abfrage: Transformationsdatenblatt alle].Transformationsdatum, [Abfrage: Transformationsdatenblatt alle].Result, [Abfrage: Transformationsdatenblatt alle].Enddatum, [Abfrage: Transformationsdatenblatt alle].[Number of Lines]
FROM [Abfrage: Transformationsdatenblatt alle];
```

Query Parameters

Name	Type
[Abfrage: Transformationsdatenblatt alle].[Number of Lines]	Text

Columns

Name	Type	Size
ID	Long Integer	4

AggregateType:	-1
AllowZeroLength:	False
AppendOnly:	False
Attributes:	Fixed Size; Auto-Increment; Updatable
CollatingOrder:	General
ColumnHidden:	False
ColumnOrder:	Default
ColumnWidth:	540
DataUpdatable:	True
GUID:	{guid {491011A1-F233-4D8B-AF6D-E85AC96DC00A}}
OrdinalPosition:	0
Required:	False
SourceField:	ID

SourceTable:	Pflanzentransformation	
TextAlign:	General	

GVO Nummer	Long Integer	4
AggregateType:	-1	
AllowZeroLength:	False	
AppendOnly:	False	
Attributes:	Fixed Size; Updatable	
CollatingOrder:	General	
ColumnHidden:	False	
ColumnOrder:	Default	
ColumnWidth:	1560	
DataUpdatable:	True	
DecimalPlaces:	Auto	
DisplayControl:	Text Box	
GUID:	{guid {78A41B5D-B257-469B-8FCD-4CB99C084269}}	
OrdinalPosition:	1	
Required:	True	
SourceField:	GVO Nummer	
SourceTable:	Pflanzentransformation	
TextAlign:	General	

Konstruktname	Text	255
AggregateType:	-1	
AllowZeroLength:	True	
AppendOnly:	False	
Attributes:	Variable Length; Updatable	
CollatingOrder:	General	
ColumnHidden:	False	
ColumnOrder:	Default	
ColumnWidth:	1965	
DataUpdatable:	True	
DisplayControl:	Text Box	
IMEMode:	0	
IMESentenceMode:	3	
OrdinalPosition:	2	
Required:	False	
SourceField:	ConstructName	
SourceTable:	Construct	
TextAlign:	General	
UnicodeCompression:	False	

Operator	Text	50
AggregateType:	-1	
AllowZeroLength:	True	
AppendOnly:	False	
Attributes:	Variable Length; Updatable	
CollatingOrder:	General	
ColumnHidden:	False	
ColumnOrder:	Default	
ColumnWidth:	1650	
DataUpdatable:	True	
Description:	Familienname verantwortlicher Wissenschafter	
DisplayControl:	Text Box	
GUID:	{guid {2AA8B70A-AC14-469A-8FAC-3C278DCB2AC7}}	
IMEMode:	0	

IMESentenceMode:	3	
OrdinalPosition:	3	
Required:	False	
SourceField:	Operator	
SourceTable:	Operator	
TextAlign:	General	
UnicodeCompression:	True	

Resistenz	Text	50
AggregateType:	-1	
AllowZeroLength:	True	
AppendOnly:	False	
Attributes:	Variable Length; Updatable	
CollatingOrder:	General	
ColumnHidden:	False	
ColumnOrder:	Default	
ColumnWidth:	495	
DataUpdatable:	True	
DisplayControl:	Text Box	
GUID:	{guid {8D71BD08-D542-4B6A-8796-02E82F37D7E2}}	
IMEMode:	0	
IMESentenceMode:	3	
OrdinalPosition:	4	
Required:	False	
SourceField:	Resistenz	
SourceTable:	Resistenz Pflanze	
TextAlign:	General	
UnicodeCompression:	True	

Spezies	Text	50
AggregateType:	-1	
AllowZeroLength:	True	
AppendOnly:	False	
Attributes:	Variable Length; Updatable	
CollatingOrder:	General	
ColumnHidden:	False	
ColumnOrder:	Default	
ColumnWidth:	1500	
DataUpdatable:	True	
DisplayControl:	Text Box	
GUID:	{guid {FA3945B0-6E6C-423D-8BEE-0935B5FD7526}}	
IMEMode:	0	
IMESentenceMode:	3	
OrdinalPosition:	5	
Required:	False	
SourceField:	Spezies	
SourceTable:	Eltern	
TextAlign:	General	
UnicodeCompression:	True	

Varietät	Text	50
AggregateType:	-1	
AllowZeroLength:	True	
AppendOnly:	False	
Attributes:	Variable Length; Updatable	
CollatingOrder:	General	

	ColumnHidden:	False	
	ColumnOrder:	Default	
	ColumnWidth:	885	
	DataUpdatable:	True	
	DisplayControl:	Text Box	
	GUID:	{guid {1AF19923-4CB9-4A37-BDF6-9A285C5D1D00}}	
	IMEMode:	0	
	IMESentenceMode:	3	
	OrdinalPosition:	6	
	Required:	False	
	SourceField:	Varietät	
	SourceTable:	Eltern	
	TextAlign:	General	
	UnicodeCompression:	True	
GMO Nummer		Long Integer	4
	AggregateType:	-1	
	AllowZeroLength:	False	
	AppendOnly:	False	
	Attributes:	Fixed Size; Updatable	
	CollatingOrder:	General	
	ColumnHidden:	False	
	ColumnOrder:	Default	
	ColumnWidth:	1380	
	DataUpdatable:	True	
	DecimalPlaces:	Auto	
	DisplayControl:	Text Box	
	GUID:	{guid {0374CE7E-E119-4264-8426-B9502000A391}}	
	OrdinalPosition:	7	
	Required:	False	
	SourceField:	GMO Nummer	
	SourceTable:	Eltern	
	TextAlign:	General	
Linie		Long Integer	4
	AggregateType:	-1	
	AllowZeroLength:	False	
	AppendOnly:	False	
	Attributes:	Fixed Size; Updatable	
	CollatingOrder:	General	
	ColumnHidden:	False	
	ColumnOrder:	Default	
	ColumnWidth:	480	
	DataUpdatable:	True	
	DecimalPlaces:	Auto	
	DisplayControl:	Text Box	
	GUID:	{guid {D523E511-9A67-4956-9BF5-D1965960274A}}	
	OrdinalPosition:	8	
	Required:	False	
	SourceField:	Linie	
	SourceTable:	Eltern	
	TextAlign:	General	
Transformationsdatum		Date/Time	8
	AggregateType:	-1	
	AllowZeroLength:	False	

AppendOnly:	False
Attributes:	Fixed Size; Updatable
CollatingOrder:	General
ColumnHidden:	False
ColumnOrder:	Default
ColumnWidth:	1365
DataUpdatable:	True
GUID:	{guid {001E5420-4F1C-4D41-9502-4F9521AA6AF1}}
IMEMode:	0
IMESentenceMode:	3
OrdinalPosition:	9
Required:	False
ShowDatePicker:	For dates
SourceField:	Transformationsdatum
SourceTable:	Pflanzentransformation
TextAlign:	General

Result Text 50

AggregateType:	-1
AllowMultipleValues:	False
AllowValueListEdits:	False
AllowZeroLength:	True
AppendOnly:	False
Attributes:	Variable Length; Updatable
BoundColumn:	1
CollatingOrder:	General
ColumnCount:	1
ColumnHeads:	False
ColumnHidden:	False
ColumnOrder:	Default
ColumnWidth:	2505
ColumnWidths:	2385
DataUpdatable:	True
DisplayControl:	Combo Box
GUID:	{guid {678F1CC4-5A06-4A1D-A7D4-BBBE683B3FA1}}
IMEMode:	0
IMESentenceMode:	3
LimitToList:	False
ListRows:	8
ListWidth:	2385twip
OrdinalPosition:	10
Required:	False
RowSource:	"übergeben";"kontaminiert";"zurückgezogen";"entsorgt: kein Kallus";"entsorgt: keine Regeneration";"andere"
RowSourceType:	Value List
ShowOnlyRowSourceValues:	False
SourceField:	Result
SourceTable:	Pflanzentransformation
TextAlign:	General
UnicodeCompression:	True

Enddatum Date/Time 8

AggregateType:	-1
AllowZeroLength:	False
AppendOnly:	False

Attributes:	Fixed Size; Updatable
CollatingOrder:	General
ColumnHidden:	False
ColumnOrder:	Default
ColumnWidth:	1335
DataUpdatable:	True
GUID:	{guid {9256514F-89E7-4417-895A-7E12A2ABA75E}}
IMEMode:	0
IMESentenceMode:	3
OrdinalPosition:	11
Required:	False
ShowDatePicker:	For dates
SourceField:	Enddatum
SourceTable:	Pflanzentransformation
TextAlign:	General

Number of Lines From 0

AggregateType:	-1
AllowZeroLength:	False
AppendOnly:	False
Attributes:	Fixed Size
CollatingOrder:	General
ColumnHidden:	False
ColumnWidth:	765
DataUpdatable:	False
OrdinalPosition:	12
Required:	False

Table Indexes

Name	Number of Fields
ArbeitsgruppenPflanzentransformation	1

Clustered:	False
DistinctCount:	1
Foreign:	True
IgnoreNulls:	False
Name:	ArbeitsgruppenPflanzentransformation
Primary:	False
Required:	False
Unique:	False
Fields:	
AG	Ascending

Id Nummer	1

Clustered:	False
DistinctCount:	4
Foreign:	False
IgnoreNulls:	False
Name:	Id Nummer
Primary:	False
Required:	False
Unique:	False
Fields:	
GVO Nummer	Ascending

Number of rooted LInes	1

	Clustered:	False
	DistinctCount:	3
	Foreign:	False
	IgnoreNulls:	False
	Name:	Number of rooted LInes
	Primary:	False
	Required:	False
	Unique:	False
	Fields:	
	Number of rooted LInes	Ascending
Number of Shots		1
	Clustered:	False
	DistinctCount:	2
	Foreign:	False
	IgnoreNulls:	False
	Name:	Number of Shots
	Primary:	False
	Required:	False
	Unique:	False
	Fields:	
	Number of Shots	Ascending
OperatorPflanzentransformation		1
	Clustered:	False
	DistinctCount:	2
	Foreign:	True
	IgnoreNulls:	False
	Name:	OperatorPflanzentransformation
	Primary:	False
	Required:	False
	Unique:	False
	Fields:	
	Worker	Ascending
PrimaryKey		1
	Clustered:	False
	DistinctCount:	7
	Foreign:	False
	IgnoreNulls:	False
	Name:	PrimaryKey
	Primary:	True
	Required:	True
	Unique:	True
	Fields:	
	ID	Ascending
Result_ID		1
	Clustered:	False
	DistinctCount:	2
	Foreign:	False
	IgnoreNulls:	False
	Name:	Result_ID
	Primary:	False
	Required:	False
	Unique:	False

	Fields:	
	Result_ID	Ascending
ConstructId		1
	Clustered:	False
	DistinctCount:	16
	Foreign:	False
	IgnoreNulls:	False
	Name:	ConstructId
	Primary:	True
	Required:	True
	Unique:	True
	Fields:	
	ConstructId	Ascending
ID		1
	Clustered:	False
	DistinctCount:	2
	Foreign:	False
	IgnoreNulls:	False
	Name:	ID
	Primary:	False
	Required:	False
	Unique:	False
	Fields:	
	ID	Ascending
ID_LIMS		1
	Clustered:	False
	DistinctCount:	2
	Foreign:	False
	IgnoreNulls:	False
	Name:	ID_LIMS
	Primary:	False
	Required:	False
	Unique:	False
	Fields:	
	ID_LIMS	Ascending
PrimaryKey		1
	Clustered:	False
	DistinctCount:	2
	Foreign:	False
	IgnoreNulls:	False
	Name:	PrimaryKey
	Primary:	True
	Required:	True
	Unique:	True
	Fields:	
	ID	Ascending
ID		1
	Clustered:	False
	DistinctCount:	12
	Foreign:	False
	IgnoreNulls:	False
	Name:	ID
	Primary:	False

	Required:	False
	Unique:	False
	Fields:	
	ID	Ascending
PrimaryKey		1
	Clustered:	False
	DistinctCount:	12
	Foreign:	False
	IgnoreNulls:	False
	Name:	PrimaryKey
	Primary:	True
	Required:	True
	Unique:	True
	Fields:	
	ID	Ascending
GMO Nummer		1
	Clustered:	False
	DistinctCount:	15
	Foreign:	False
	IgnoreNulls:	False
	Name:	GMO Nummer
	Primary:	False
	Required:	False
	Unique:	False
	Fields:	
	GMO Nummer	Ascending
ID		1
	Clustered:	False
	DistinctCount:	28
	Foreign:	False
	IgnoreNulls:	False
	Name:	ID
	Primary:	False
	Required:	False
	Unique:	False
	Fields:	
	ID	Ascending
ID_Pflanzenarten_Ref		1
	Clustered:	False
	DistinctCount:	4
	Foreign:	False
	IgnoreNulls:	False
	Name:	ID_Pflanzenarten_Ref
	Primary:	False
	Required:	False
	Unique:	False
	Fields:	
	ID_Pflanzenarten_Ref	Ascending
PrimaryKey		1
	Clustered:	False
	DistinctCount:	28
	Foreign:	False
	IgnoreNulls:	False

Name:	PrimaryKey
Primary:	True
Required:	True
Unique:	True
Fields:	
ID	Ascending

ArbeitsgruppenPflanzentransformation 1

Clustered:	False
DistinctCount:	1
Foreign:	True
IgnoreNulls:	False
Name:	ArbeitsgruppenPflanzentransformation
Primary:	False
Required:	False
Unique:	False
Fields:	
AG	Ascending

Id Nummer 1

Clustered:	False
DistinctCount:	4
Foreign:	False
IgnoreNulls:	False
Name:	Id Nummer
Primary:	False
Required:	False
Unique:	False
Fields:	
GVO Nummer	Ascending

Number of rooted LInes 1

Clustered:	False
DistinctCount:	3
Foreign:	False
IgnoreNulls:	False
Name:	Number of rooted LInes
Primary:	False
Required:	False
Unique:	False
Fields:	
Number of rooted LInes	Ascending

Number of Shots 1

Clustered:	False
DistinctCount:	2
Foreign:	False
IgnoreNulls:	False
Name:	Number of Shots
Primary:	False
Required:	False
Unique:	False
Fields:	
Number of Shots	Ascending

OperatorPflanzentransformation 1

Clustered:	False
DistinctCount:	2

	Foreign:	True
	IgnoreNulls:	False
	Name:	OperatorPflanzentransformation
	Primary:	False
	Required:	False
	Unique:	False
	Fields:	
	Worker	Ascending
PrimaryKey		1
	Clustered:	False
	DistinctCount:	7
	Foreign:	False
	IgnoreNulls:	False
	Name:	PrimaryKey
	Primary:	True
	Required:	True
	Unique:	True
	Fields:	
	ID	Ascending
Result_ID		1
	Clustered:	False
	DistinctCount:	2
	Foreign:	False
	IgnoreNulls:	False
	Name:	Result_ID
	Primary:	False
	Required:	False
	Unique:	False
	Fields:	
	Result_ID	Ascending

User Permissions

admin	Delete; Read Permissions; Set Permissions; Change Owner, Read Definition; Write Definition; Read Data; Insert Data; Update Data; Delete Data

Group Permissions

Admins	Delete; Read Permissions; Set Permissions; Change Owner, Read Definition; Write Definition; Read Data; Insert Data; Update Data; Delete Data
Users	Delete; Read Permissions; Set Permissions; Change Owner, Read Definition; Write Definition; Read Data; Insert Data; Update Data; Delete Data

Properties

DateCreated:	23.08.2007 14:47:53	DefaultView:	2
DOL:	Long binary data	GUID:	{guid {A287EC0F-517F-4456-8D85-F1E2CEBEFBED}}
LastUpdated:	09.04.2009 12:10:51	MaxRecords:	0
ODBCTimeout:	60	OrderByOn:	False
Orientation:	Left-to-Right	RecordLocks:	No Locks
RecordsAffected:	0	RecordsetType:	Dynaset
ReturnsRecords:	True	Type:	0
Updatable:	True		

SQL

```
SELECT [Abfrage Pflanzentransformation].ID, [Abfrage Pflanzentransformation].[GVO Nummer], [Abfrage
Pflanzentransformation].Konstruktname, Operator.Operator, [Abfrage
Pflanzentransformation].Transformationsdatum, Protokoll.Protokollname, [Abfrage
Pflanzentransformation].Protokoll, [Abfrage Pflanzentransformation].[Number of Lines]
FROM ([Abfrage Pflanzentransformation] INNER JOIN Protokoll ON [Abfrage
Pflanzentransformation].Protokoll=Protokoll.[Protokoll ID]) INNER JOIN Operator ON [Abfrage
Pflanzentransformation].Worker=Operator.ID
WHERE ((([Abfrage Pflanzentransformation].Transformationsdatum)>#1/1/2007#) AND
((Protokoll.Protokollname) Like "tomat*"));
```

Query Parameters

Name	Type
[Abfrage Pflanzentransformation].Konstruktname	Text

Columns

Name	Type	Size
ID	Long Integer	4

AggregateType:	-1
AllowZeroLength:	False
AppendOnly:	False
Attributes:	Fixed Size; Auto-Increment; Updatable
CollatingOrder:	General
ColumnHidden:	False
ColumnOrder:	Default
ColumnWidth:	Default
DataUpdatable:	True
GUID:	{guid {491011A1-F233-4D8B-AF6D-E85AC96DC00A}}
OrdinalPosition:	0
Required:	False
SourceField:	ID
SourceTable:	Pflanzentransformation
TextAlign:	General

Name	Type	Size
GVO Nummer	Long Integer	4

AggregateType:	-1
AllowZeroLength:	False

AppendOnly:	False
Attributes:	Fixed Size; Updatable
CollatingOrder:	General
ColumnHidden:	False
ColumnOrder:	Default
ColumnWidth:	1650
DataUpdatable:	True
DecimalPlaces:	Auto
DefaultValue:	0
DisplayControl:	Text Box
GUID:	{guid {78A41B5D-B257-469B-8FCD-4CB99C084269}}
OrdinalPosition:	1
Required:	True
SourceField:	GVO Nummer
SourceTable:	Pflanzentransformation
TextAlign:	General

Konstruktname From 0

AllowZeroLength:	False
AppendOnly:	False
Attributes:	Fixed Size
CollatingOrder:	General
DataUpdatable:	False
OrdinalPosition:	2
Required:	False

Operator Text 50

AggregateType:	-1
AllowZeroLength:	True
AppendOnly:	False
Attributes:	Variable Length; Updatable
CollatingOrder:	General
ColumnHidden:	False
ColumnOrder:	Default
ColumnWidth:	1935
DataUpdatable:	True
Description:	Familienname verantwortlicher Wissenschafter
DisplayControl:	Text Box
GUID:	{guid {2AA8B70A-AC14-469A-8FAC-3C278DCB2AC7}}
IMEMode:	0
IMESentenceMode:	3
OrdinalPosition:	3
Required:	False
SourceField:	Operator
SourceTable:	Operator
TextAlign:	General
UnicodeCompression:	True

Transformationsdatum Date/Time 8

AggregateType:	-1
AllowZeroLength:	False
AppendOnly:	False
Attributes:	Fixed Size; Updatable
CollatingOrder:	General
ColumnHidden:	False
ColumnOrder:	Default

ColumnWidth:	1440
DataUpdatable:	True
GUID:	{guid {001E5420-4F1C-4D41-9502-4F9521AA6AF1}}
IMEMode:	0
IMESentenceMode:	3
OrdinalPosition:	4
Required:	False
ShowDatePicker:	For dates
SourceField:	Transformationsdatum
SourceTable:	Pflanzentransformation
TextAlign:	General

Protokollname Text 50

AggregateType:	-1
AllowZeroLength:	True
AppendOnly:	False
Attributes:	Variable Length; Updatable
CollatingOrder:	General
ColumnHidden:	False
ColumnOrder:	Default
ColumnWidth:	4905
DataUpdatable:	True
Description:	Eindeutiger Name für das Protokoll
DisplayControl:	Text Box
GUID:	{guid {BA35B293-EC1A-433F-A9CA-F649675F83D2}}
IMEMode:	0
IMESentenceMode:	3
OrdinalPosition:	5
Required:	False
SourceField:	Protokollname
SourceTable:	Protokoll
TextAlign:	General
UnicodeCompression:	True

Abfrage Pflanzentransformation.Protokoll Long Integer 4

AggregateType:	-1
AllowZeroLength:	False
AppendOnly:	False
Attributes:	Fixed Size; Updatable
CollatingOrder:	General
ColumnHidden:	False
ColumnOrder:	Default
ColumnWidth:	Default
DataUpdatable:	True
DecimalPlaces:	Auto
DefaultValue:	0
DisplayControl:	Text Box
GUID:	{guid {66638D24-E387-4387-948D-82A060A41F76}}
OrdinalPosition:	6
Required:	False
SourceField:	Protokoll
SourceTable:	Pflanzentransformation
TextAlign:	General

Number of Lines Long Integer 4

AggregateType:	-1

AllowZeroLength:	False
AppendOnly:	False
Attributes:	Fixed Size; Updatable
CollatingOrder:	General
ColumnHidden:	False
ColumnOrder:	Default
ColumnWidth:	2130
DataUpdatable:	True
DecimalPlaces:	Auto
DefaultValue:	0
Description:	Number of Lines that have been cut (festgelegt 06.05.09)
DisplayControl:	Text Box
GUID:	{guid {85595EC7-656A-4467-A6CE-21630FBEB0B4}}
OrdinalPosition:	7
Required:	False
SourceField:	Number of Lines
SourceTable:	Pflanzentransformation
TextAlign:	General

Table Indexes

Name	Number of Fields
ArbeitsgruppenPflanzentransformation	1
Clustered:	False
DistinctCount:	1
Foreign:	True
IgnoreNulls:	False
Name:	ArbeitsgruppenPflanzentransformation
Primary:	False
Required:	False
Unique:	False
Fields:	
AG	Ascending
Id Nummer	1
Clustered:	False
DistinctCount:	4
Foreign:	False
IgnoreNulls:	False
Name:	Id Nummer
Primary:	False
Required:	False
Unique:	False
Fields:	
GVO Nummer	Ascending
Number of rooted LInes	1
Clustered:	False
DistinctCount:	3
Foreign:	False
IgnoreNulls:	False
Name:	Number of rooted LInes
Primary:	False
Required:	False
Unique:	False

	Fields:	
	Number of rooted LInes	Ascending
Number of Shots		1
	Clustered:	False
	DistinctCount:	2
	Foreign:	False
	IgnoreNulls:	False
	Name:	Number of Shots
	Primary:	False
	Required:	False
	Unique:	False
	Fields:	
	Number of Shots	Ascending
OperatorPflanzentransformation		1
	Clustered:	False
	DistinctCount:	2
	Foreign:	True
	IgnoreNulls:	False
	Name:	OperatorPflanzentransformation
	Primary:	False
	Required:	False
	Unique:	False
	Fields:	
	Worker	Ascending
PrimaryKey		1
	Clustered:	False
	DistinctCount:	7
	Foreign:	False
	IgnoreNulls:	False
	Name:	PrimaryKey
	Primary:	True
	Required:	True
	Unique:	True
	Fields:	
	ID	Ascending
Result_ID		1
	Clustered:	False
	DistinctCount:	2
	Foreign:	False
	IgnoreNulls:	False
	Name:	Result_ID
	Primary:	False
	Required:	False
	Unique:	False
	Fields:	
	Result_ID	Ascending
ID		1
	Clustered:	False
	DistinctCount:	2
	Foreign:	False
	IgnoreNulls:	False
	Name:	ID
	Primary:	False

	Required:	False
	Unique:	False
	Fields:	
	ID	Ascending
ID_LIMS		1
	Clustered:	False
	DistinctCount:	2
	Foreign:	False
	IgnoreNulls:	False
	Name:	ID_LIMS
	Primary:	False
	Required:	False
	Unique:	False
	Fields:	
	ID_LIMS	Ascending
PrimaryKey		1
	Clustered:	False
	DistinctCount:	2
	Foreign:	False
	IgnoreNulls:	False
	Name:	PrimaryKey
	Primary:	True
	Required:	True
	Unique:	True
	Fields:	
	ID	Ascending
ArbeitsgruppenPflanzentransformation		1
	Clustered:	False
	DistinctCount:	1
	Foreign:	True
	IgnoreNulls:	False
	Name:	ArbeitsgruppenPflanzentransformation
	Primary:	False
	Required:	False
	Unique:	False
	Fields:	
	AG	Ascending
Id Nummer		1
	Clustered:	False
	DistinctCount:	4
	Foreign:	False
	IgnoreNulls:	False
	Name:	Id Nummer
	Primary:	False
	Required:	False
	Unique:	False
	Fields:	
	GVO Nummer	Ascending
Number of rooted LInes		1
	Clustered:	False
	DistinctCount:	3
	Foreign:	False
	IgnoreNulls:	False

Name:	Number of rooted LInes
Primary:	False
Required:	False
Unique:	False
Fields:	
Number of rooted LInes	Ascending

Number of Shots 1

Clustered:	False
DistinctCount:	2
Foreign:	False
IgnoreNulls:	False
Name:	Number of Shots
Primary:	False
Required:	False
Unique:	False
Fields:	
Number of Shots	Ascending

OperatorPflanzentransformation 1

Clustered:	False
DistinctCount:	2
Foreign:	True
IgnoreNulls:	False
Name:	OperatorPflanzentransformation
Primary:	False
Required:	False
Unique:	False
Fields:	
Worker	Ascending

PrimaryKey 1

Clustered:	False
DistinctCount:	7
Foreign:	False
IgnoreNulls:	False
Name:	PrimaryKey
Primary:	True
Required:	True
Unique:	True
Fields:	
ID	Ascending

Result_ID 1

Clustered:	False
DistinctCount:	2
Foreign:	False
IgnoreNulls:	False
Name:	Result_ID
Primary:	False
Required:	False
Unique:	False
Fields:	
Result_ID	Ascending

PrimaryKey 1

Clustered:	False
DistinctCount:	3

	Foreign:	False
	IgnoreNulls:	False
	Name:	PrimaryKey
	Primary:	True
	Required:	True
	Unique:	True
	Fields:	
	Protokoll ID	Ascending
ProtokollArt		1
	Clustered:	False
	DistinctCount:	1
	Foreign:	False
	IgnoreNulls:	False
	Name:	ProtokollArt
	Primary:	False
	Required:	False
	Unique:	False
	Fields:	
	Art	Ascending
ArbeitsgruppenPflanzentransformation		1
	Clustered:	False
	DistinctCount:	1
	Foreign:	True
	IgnoreNulls:	False
	Name:	ArbeitsgruppenPflanzentransformation
	Primary:	False
	Required:	False
	Unique:	False
	Fields:	
	AG	Ascending
Id Nummer		1
	Clustered:	False
	DistinctCount:	4
	Foreign:	False
	IgnoreNulls:	False
	Name:	Id Nummer
	Primary:	False
	Required:	False
	Unique:	False
	Fields:	
	GVO Nummer	Ascending
Number of rooted LInes		1
	Clustered:	False
	DistinctCount:	3
	Foreign:	False
	IgnoreNulls:	False
	Name:	Number of rooted LInes
	Primary:	False
	Required:	False
	Unique:	False
	Fields:	
	Number of rooted LInes	Ascending
Number of Shots		1

Clustered:	False
DistinctCount:	2
Foreign:	False
IgnoreNulls:	False
Name:	Number of Shots
Primary:	False
Required:	False
Unique:	False
Fields:	
Number of Shots	Ascending

OperatorPflanzentransformation 1

Clustered:	False
DistinctCount:	2
Foreign:	True
IgnoreNulls:	False
Name:	OperatorPflanzentransformation
Primary:	False
Required:	False
Unique:	False
Fields:	
Worker	Ascending

PrimaryKey 1

Clustered:	False
DistinctCount:	7
Foreign:	False
IgnoreNulls:	False
Name:	PrimaryKey
Primary:	True
Required:	True
Unique:	True
Fields:	
ID	Ascending

Result_ID 1

Clustered:	False
DistinctCount:	2
Foreign:	False
IgnoreNulls:	False
Name:	Result_ID
Primary:	False
Required:	False
Unique:	False
Fields:	
Result_ID	Ascending

User Permissions

admin	Delete; Read Permissions; Set Permissions; Change Owner, Read Definition; Write Definition; Read Data; Insert Data; Update Data; Delete Data

Group Permissions

Admins	Delete; Read Permissions; Set Permissions; Change Owner, Read Definition; Write Definition; Read Data; Insert Data; Update Data; Delete Data
Users	Delete; Read Permissions; Set Permissions; Change Owner, Read Definition; Write Definition; Read Data; Insert Data; Update Data; Delete Data

Properties

DateCreated:	24.09.2009 09:54:13	DefaultView:	2
FilterOnLoad:	False	GUID:	{guid {89A39351-559F-473E-9BC6-E5142AFA2282}}
LastUpdated:	24.09.2009 09:54:13	MaxRecords:	0
ODBCTimeout:	60	OrderBy:	[Retrieve_Construct_Data_from_LIMS].[U_CONSTRUCT_ID]
OrderByOn:	True	OrderByOnLoad:	True
Orientation:	Left-to-Right	RecordLocks:	No Locks
RecordsAffected:	0	RecordsetType:	Dynaset
ReturnsRecords:	True	TotalsRow:	False
Type:	0	Updatable:	True

SQL

```
SELECT c.U_CONSTRUCT_ID, c.NAME, cu.U_M_RESISTANCE,
M_Resistance_Options_from_LIMS.ID_Resistenz_Bakterien, cu.U_PLANT_RESISTANCE,
Plant_Resistance_Options_from_LIMS.ID_Resistenz_Pflanze
FROM ((LIMS_SYS_U_CONSTRUCT AS c INNER JOIN LIMS_SYS_U_CONSTRUCT_USER AS cu ON
c.U_CONSTRUCT_ID = cu.U_CONSTRUCT_ID) LEFT JOIN M_Resistance_Options_from_LIMS ON
cu.U_M_RESISTANCE = M_Resistance_Options_from_LIMS.U_M_RESISTANCE) LEFT JOIN
Plant_Resistance_Options_from_LIMS ON cu.U_PLANT_RESISTANCE =
Plant_Resistance_Options_from_LIMS.U_PLANT_RESISTANCE
WHERE cu.U_CONSTRUCT_ID > (select max(con.ConstructId) from Construct con);
```

Columns

Name	Type	Size
U_CONSTRUCT_ID	Alias	16

AggregateType:	-1
AllowZeroLength:	False
AppendOnly:	False
Attributes:	Fixed Size
CollatingOrder:	16
ColumnHidden:	False
ColumnOrder:	Default
ColumnWidth:	2070
DataUpdatable:	False
DecimalPlaces:	Auto
DisplayControl:	Text Box
OrdinalPosition:	0
Required:	True
SourceField:	U_CONSTRUCT_ID
SourceTable:	LIMS_SYS_U_CONSTRUCT
TextAlign:	General

Name	Type	Size
NAME	Text	255

AggregateType:	-1
AllowZeroLength:	True
AppendOnly:	False
Attributes:	Variable Length
CollatingOrder:	General
ColumnHidden:	False
ColumnOrder:	Default

ColumnWidth:	Default	
DataUpdatable:	False	
DisplayControl:	Text Box	
IMEMode:	0	
IMESentenceMode:	3	
OrdinalPosition:	1	
Required:	False	
SourceField:	NAME	
SourceTable:	LIMS_SYS_U_CONSTRUCT	
TextAlign:	General	
UnicodeCompression:	False	

U_M_RESISTANCE	Text	30
AggregateType:	-1	
AllowZeroLength:	True	
AppendOnly:	False	
Attributes:	Variable Length	
CollatingOrder:	General	
ColumnHidden:	False	
ColumnOrder:	Default	
ColumnWidth:	2310	
DataUpdatable:	False	
DisplayControl:	Text Box	
IMEMode:	0	
IMESentenceMode:	3	
OrdinalPosition:	2	
Required:	False	
SourceField:	U_M_RESISTANCE	
SourceTable:	LIMS_SYS_U_CONSTRUCT_USER	
TextAlign:	General	
UnicodeCompression:	False	

ID_Resistenz_Bakterien	Long Integer	4
AggregateType:	-1	
AllowZeroLength:	False	
AppendOnly:	False	
Attributes:	Fixed Size	
CollatingOrder:	General	
ColumnHidden:	False	
ColumnOrder:	Default	
ColumnWidth:	3255	
DataUpdatable:	False	
DecimalPlaces:	Auto	
DisplayControl:	Text Box	
OrdinalPosition:	3	
Required:	False	
SourceField:	ID_Resistenz_Bakterien	
SourceTable:	M_Resistance_Options_from_LIMS	
TextAlign:	General	

U_PLANT_RESISTANCE	Text	30
AggregateType:	-1	
AllowZeroLength:	True	
AppendOnly:	False	
Attributes:	Variable Length	
CollatingOrder:	General	

ColumnHidden:	False
ColumnOrder:	Default
ColumnWidth:	2625
DataUpdatable:	False
DisplayControl:	Text Box
IMEMode:	0
IMESentenceMode:	3
OrdinalPosition:	4
Required:	False
SourceField:	U_PLANT_RESISTANCE
SourceTable:	LIMS_SYS_U_CONSTRUCT_USER
TextAlign:	General
UnicodeCompression:	False

ID_Resistenz_Pflanze Long Integer 4

AggregateType:	-1
AllowZeroLength:	False
AppendOnly:	False
Attributes:	Fixed Size
CollatingOrder:	General
ColumnHidden:	False
ColumnOrder:	Default
ColumnWidth:	2340
DataUpdatable:	False
DecimalPlaces:	Auto
DisplayControl:	Text Box
OrdinalPosition:	5
Required:	False
SourceField:	ID_Resistenz_Pflanze
SourceTable:	Plant_Resistance_Options_from_LIMS
TextAlign:	General

Table Indexes

Name	Number of Fields
ID_Resistenz_Bakterien	1

Clustered:	False
DistinctCount:	16
Foreign:	False
IgnoreNulls:	False
Name:	ID_Resistenz_Bakterien
Primary:	False
Required:	False
Unique:	False
Fields:	
ID_Resistenz_Bakterien	Ascending

ID_Resistenz_Pflanze 1

Clustered:	False
DistinctCount:	13
Foreign:	False
IgnoreNulls:	False
Name:	ID_Resistenz_Pflanze
Primary:	False
Required:	False

Unique:	False
Fields:	
ID_Resistenz_Pflanze	Ascending

User Permissions

admin	Delete; Read Permissions; Set Permissions; Change Owner, Read Definition; Write Definition; Read Data; Insert Data; Update Data; Delete Data

Group Permissions

Admins	Delete; Read Permissions; Set Permissions; Change Owner, Read Definition; Write Definition; Read Data; Insert Data; Update Data; Delete Data
Users	Delete; Read Permissions; Set Permissions; Change Owner, Read Definition; Write Definition; Read Data; Insert Data; Update Data; Delete Data

Properties

DatasheetFontHeight:	10	DatasheetFontItalic:	False
DatasheetFontName:	Arial	DatasheetFontUnderline:	False
DatasheetFontWeight:	Normal	DatasheetForeColor:	33554432
DatasheetForeColor12:	33554432	DateCreated:	15.10.2009 17:02:59
DefaultView:	2	DOL:	Long binary data
FilterOnLoad:	False	GUID:	{guid {AA70AB65-9308-4CF9-BE4D-FA59727D54A8}}
LastUpdated:	11.01.2010 09:48:40	MaxRecords:	0
ODBCTimeout:	60	OrderByOn:	False
OrderByOnLoad:	True	Orientation:	Left-to-Right
RecordLocks:	No Locks	RecordsAffected:	0
RecordsetType:	Dynaset	ReturnsRecords:	True
RowHeight:	495	TabularCharSet:	0
TabularFamily:	34	TotalsRow:	False
Type:	0	Updatable:	True

SQL

```
SELECT Pflanzentransformation.ID, Pflanzentransformation.[GVO Nummer] AS [Construct Id],
Operator.Operator, Protokoll.Protokollname AS Protocol, Eltern.Varietät AS Variety,
Pflanzentransformation.Transformationsdatum AS [Transformation date],
[Transformationsdatum]+[ZeitvonStart] AS Date7, Transformationsschritte.Prozess AS Step, [Resistenz
Pflanze].Resistenz AS Resistance, Pflanzentransformation.[Number of Lines]
FROM Operator INNER JOIN (([Resistenz Pflanze] RIGHT JOIN Construct ON [Resistenz Pflanze].ID =
Construct.[Plant Resistance]) INNER JOIN ((Protokoll INNER JOIN (Eltern RIGHT JOIN Pflanzentransformation
ON Eltern.ID = Pflanzentransformation.Pflanze) ON Protokoll.[Protokoll ID] =
Pflanzentransformation.Protokoll) INNER JOIN Transformationsschritte ON Protokoll.[Protokoll ID] =
Transformationsschritte.Art) ON Construct.ConstructId = Pflanzentransformation.[GVO Nummer]) ON
Operator.ID = Pflanzentransformation.Worker
WHERE ((([Transformationsdatum]+[ZeitvonStart])>=Date() And
([Transformationsdatum]+[ZeitvonStart])<=(Date()+31)) AND ((Pflanzentransformation.Enddatum) Is Null Or
(Pflanzentransformation.Enddatum)>=Date()))
ORDER BY [Transformationsdatum]+[ZeitvonStart];
```

Columns

Name	Type	Size
ID	Long Integer	4

AggregateType:	-1
AllowZeroLength:	False
AppendOnly:	False
Attributes:	Fixed Size; Auto-Increment
CollatingOrder:	General
ColumnHidden:	False
ColumnOrder:	1
ColumnWidth:	570
DataUpdatable:	False
GUID:	{guid {491011A1-F233-4D8B-AF6D-E85AC96DC00A}}
OrdinalPosition:	0
Required:	False
SourceField:	ID
SourceTable:	Pflanzentransformation
TextAlign:	General

Construct Id	Long Integer	4
AggregateType:	-1	
AllowZeroLength:	False	
AppendOnly:	False	
Attributes:	Fixed Size	
CollatingOrder:	General	
ColumnHidden:	False	
ColumnOrder:	5	
ColumnWidth:	945	
DataUpdatable:	False	
DecimalPlaces:	Auto	
DefaultValue:	0	
DisplayControl:	Text Box	
GUID:	{guid {78A41B5D-B257-469B-8FCD-4CB99C084269}}	
OrdinalPosition:	1	
Required:	True	
SourceField:	GVO Nummer	
SourceTable:	Pflanzentransformation	
TextAlign:	General	
Operator	Text	50
AggregateType:	-1	
AllowZeroLength:	True	
AppendOnly:	False	
Attributes:	Variable Length	
CollatingOrder:	General	
ColumnHidden:	False	
ColumnOrder:	9	
ColumnWidth:	1620	
DataUpdatable:	False	
Description:	Familienname verantwortlicher Wissenschafter	
DisplayControl:	Text Box	
GUID:	{guid {2AA8B70A-AC14-469A-8FAC-3C278DCB2AC7}}	
IMEMode:	0	
IMESentenceMode:	3	
OrdinalPosition:	2	
Required:	False	
SourceField:	Operator	
SourceTable:	Operator	
TextAlign:	General	
UnicodeCompression:	True	
Protocol	Text	50
AggregateType:	-1	
AllowZeroLength:	True	
AppendOnly:	False	
Attributes:	Variable Length	
CollatingOrder:	General	
ColumnHidden:	False	
ColumnOrder:	6	
ColumnWidth:	2835	
DataUpdatable:	False	
Description:	Eindeutiger Name für das Protokoll	
DisplayControl:	Text Box	
GUID:	{guid {BA35B293-EC1A-433F-A9CA-F649675F83D2}}	
IMEMode:	0	

IMESentenceMode:	3	
OrdinalPosition:	3	
Required:	False	
SourceField:	Protokollname	
SourceTable:	Protokoll	
TextAlign:	General	
UnicodeCompression:	True	

Variety	Text	50
AggregateType:	-1	
AllowZeroLength:	True	
AppendOnly:	False	
Attributes:	Variable Length	
CollatingOrder:	General	
ColumnHidden:	False	
ColumnOrder:	3	
ColumnWidth:	1095	
DataUpdatable:	False	
DisplayControl:	Text Box	
GUID:	{guid {1AF19923-4CB9-4A37-BDF6-9A285C5D1D00}}	
IMEMode:	0	
IMESentenceMode:	3	
OrdinalPosition:	4	
Required:	False	
SourceField:	Varietät	
SourceTable:	Eltern	
TextAlign:	General	
UnicodeCompression:	True	

Transformation date	Date/Time	8
AggregateType:	-1	
AllowZeroLength:	False	
AppendOnly:	False	
Attributes:	Fixed Size	
CollatingOrder:	General	
ColumnHidden:	False	
ColumnOrder:	8	
ColumnWidth:	1410	
DataUpdatable:	False	
GUID:	{guid {001E5420-4F1C-4D41-9502-4F9521AA6AF1}}	
IMEMode:	0	
IMESentenceMode:	3	
OrdinalPosition:	5	
Required:	False	
ShowDatePicker:	For dates	
SourceField:	Transformationsdatum	
SourceTable:	Pflanzentransformation	
TextAlign:	General	

Date7	Date/Time	8
AggregateType:	-1	
AllowZeroLength:	False	
AppendOnly:	False	
Attributes:	Fixed Size	
CollatingOrder:	General	
ColumnHidden:	False	

ColumnOrder:	2	
ColumnWidth:	1530	
DataUpdatable:	False	
GUID:	{guid {3074E61C-0000-0000-1CE6-743000000000}}	
OrdinalPosition:	6	
Required:	False	

Step	Text	50
AggregateType:	-1	
AllowZeroLength:	True	
AppendOnly:	False	
Attributes:	Variable Length	
CollatingOrder:	General	
ColumnHidden:	False	
ColumnOrder:	4	
ColumnWidth:	2625	
DataUpdatable:	False	
Description:	Arbeitsprozess	
DisplayControl:	Text Box	
GUID:	{guid {F145B5A2-C291-40CF-8D68-20B48F7A3039}}	
IMEMode:	0	
IMESentenceMode:	3	
OrdinalPosition:	7	
Required:	False	
SourceField:	Prozess	
SourceTable:	Transformationsschritte	
TextAlign:	General	
UnicodeCompression:	True	

Resistance	Text	50
AggregateType:	-1	
AllowZeroLength:	True	
AppendOnly:	False	
Attributes:	Variable Length	
CollatingOrder:	General	
ColumnHidden:	False	
ColumnOrder:	7	
ColumnWidth:	570	
DataUpdatable:	False	
DisplayControl:	Text Box	
GUID:	{guid {8D71BD08-D542-4B6A-8796-02E82F37D7E2}}	
IMEMode:	0	
IMESentenceMode:	3	
OrdinalPosition:	8	
Required:	False	
SourceField:	Resistenz	
SourceTable:	Resistenz Pflanze	
TextAlign:	General	
UnicodeCompression:	True	

Number of Lines	Long Integer	4
AggregateType:	-1	
AllowZeroLength:	False	
AppendOnly:	False	
Attributes:	Fixed Size	
CollatingOrder:	General	

ColumnHidden:	False
ColumnOrder:	Default
ColumnWidth:	Default
DataUpdatable:	False
DecimalPlaces:	Auto
DefaultValue:	0
Description:	Number of Lines that have been cut (festgelegt 06.05.09)
DisplayControl:	Text Box
GUID:	{guid {85595EC7-656A-4467-A6CE-21630FBEB0B4}}
OrdinalPosition:	9
Required:	False
SourceField:	Number of Lines
SourceTable:	Pflanzentransformation
TextAlign:	General

Table Indexes

Name		Number of Fields
ArbeitsgruppenPflanzentransformation		1
	Clustered:	False
	DistinctCount:	1
	Foreign:	True
	IgnoreNulls:	False
	Name:	ArbeitsgruppenPflanzentransformation
	Primary:	False
	Required:	False
	Unique:	False
	Fields:	
	AG	Ascending
Id Nummer		1
	Clustered:	False
	DistinctCount:	4
	Foreign:	False
	IgnoreNulls:	False
	Name:	Id Nummer
	Primary:	False
	Required:	False
	Unique:	False
	Fields:	
	GVO Nummer	Ascending
Number of rooted LInes		1
	Clustered:	False
	DistinctCount:	3
	Foreign:	False
	IgnoreNulls:	False
	Name:	Number of rooted LInes
	Primary:	False
	Required:	False
	Unique:	False
	Fields:	
	Number of rooted LInes	Ascending
Number of Shots		1
	Clustered:	False

	DistinctCount:	2
	Foreign:	False
	IgnoreNulls:	False
	Name:	Number of Shots
	Primary:	False
	Required:	False
	Unique:	False
	Fields:	
	Number of Shots	Ascending
OperatorPflanzentransformation		1
	Clustered:	False
	DistinctCount:	2
	Foreign:	True
	IgnoreNulls:	False
	Name:	OperatorPflanzentransformation
	Primary:	False
	Required:	False
	Unique:	False
	Fields:	
	Worker	Ascending
PrimaryKey		1
	Clustered:	False
	DistinctCount:	7
	Foreign:	False
	IgnoreNulls:	False
	Name:	PrimaryKey
	Primary:	True
	Required:	True
	Unique:	True
	Fields:	
	ID	Ascending
Result_ID		1
	Clustered:	False
	DistinctCount:	2
	Foreign:	False
	IgnoreNulls:	False
	Name:	Result_ID
	Primary:	False
	Required:	False
	Unique:	False
	Fields:	
	Result_ID	Ascending
ID		1
	Clustered:	False
	DistinctCount:	2
	Foreign:	False
	IgnoreNulls:	False
	Name:	ID
	Primary:	False
	Required:	False
	Unique:	False
	Fields:	
	ID	Ascending

ID_LIMS		1
	Clustered:	False
	DistinctCount:	2
	Foreign:	False
	IgnoreNulls:	False
	Name:	ID_LIMS
	Primary:	False
	Required:	False
	Unique:	False
	Fields:	
	ID_LIMS	Ascending
PrimaryKey		1
	Clustered:	False
	DistinctCount:	2
	Foreign:	False
	IgnoreNulls:	False
	Name:	PrimaryKey
	Primary:	True
	Required:	True
	Unique:	True
	Fields:	
	ID	Ascending
PrimaryKey		1
	Clustered:	False
	DistinctCount:	3
	Foreign:	False
	IgnoreNulls:	False
	Name:	PrimaryKey
	Primary:	True
	Required:	True
	Unique:	True
	Fields:	
	Protokoll ID	Ascending
ProtokollArt		1
	Clustered:	False
	DistinctCount:	1
	Foreign:	False
	IgnoreNulls:	False
	Name:	ProtokollArt
	Primary:	False
	Required:	False
	Unique:	False
	Fields:	
	Art	Ascending
GMO Nummer		1
	Clustered:	False
	DistinctCount:	15
	Foreign:	False
	IgnoreNulls:	False
	Name:	GMO Nummer
	Primary:	False
	Required:	False
	Unique:	False

Fields:
GMO Nummer Ascending
ID 1
Clustered: False
DistinctCount: 28
Foreign: False
IgnoreNulls: False
Name: ID
Primary: False
Required: False
Unique: False
Fields:
ID Ascending
ID_Pflanzenarten_Ref 1
Clustered: False
DistinctCount: 4
Foreign: False
IgnoreNulls: False
Name: ID_Pflanzenarten_Ref
Primary: False
Required: False
Unique: False
Fields:
ID_Pflanzenarten_Ref Ascending
PrimaryKey 1
Clustered: False
DistinctCount: 28
Foreign: False
IgnoreNulls: False
Name: PrimaryKey
Primary: True
Required: True
Unique: True
Fields:
ID Ascending
ArbeitsgruppenPflanzentransformation 1
Clustered: False
DistinctCount: 1
Foreign: True
IgnoreNulls: False
Name: ArbeitsgruppenPflanzentransformation
Primary: False
Required: False
Unique: False
Fields:
AG Ascending
Id Nummer 1
Clustered: False
DistinctCount: 4
Foreign: False
IgnoreNulls: False
Name: Id Nummer
Primary: False

	Required:	False
	Unique:	False
	Fields:	
	GVO Nummer	Ascending
Number of rooted LInes		1
	Clustered:	False
	DistinctCount:	3
	Foreign:	False
	IgnoreNulls:	False
	Name:	Number of rooted LInes
	Primary:	False
	Required:	False
	Unique:	False
	Fields:	
	Number of rooted LInes	Ascending
Number of Shots		1
	Clustered:	False
	DistinctCount:	2
	Foreign:	False
	IgnoreNulls:	False
	Name:	Number of Shots
	Primary:	False
	Required:	False
	Unique:	False
	Fields:	
	Number of Shots	Ascending
OperatorPflanzentransformation		1
	Clustered:	False
	DistinctCount:	2
	Foreign:	True
	IgnoreNulls:	False
	Name:	OperatorPflanzentransformation
	Primary:	False
	Required:	False
	Unique:	False
	Fields:	
	Worker	Ascending
PrimaryKey		1
	Clustered:	False
	DistinctCount:	7
	Foreign:	False
	IgnoreNulls:	False
	Name:	PrimaryKey
	Primary:	True
	Required:	True
	Unique:	True
	Fields:	
	ID	Ascending
Result_ID		1
	Clustered:	False
	DistinctCount:	2
	Foreign:	False
	IgnoreNulls:	False

	Name:	Result_ID
	Primary:	False
	Required:	False
	Unique:	False
	Fields:	
	Result_ID	Ascending
Old_ID		1
	Clustered:	False
	DistinctCount:	4
	Foreign:	False
	IgnoreNulls:	False
	Name:	Old_ID
	Primary:	False
	Required:	False
	Unique:	False
	Fields:	
	Old_ID	Ascending
PrimaryKey		1
	Clustered:	False
	DistinctCount:	9
	Foreign:	False
	IgnoreNulls:	False
	Name:	PrimaryKey
	Primary:	True
	Required:	True
	Unique:	True
	Fields:	
	ID	Ascending
ID		1
	Clustered:	False
	DistinctCount:	12
	Foreign:	False
	IgnoreNulls:	False
	Name:	ID
	Primary:	False
	Required:	False
	Unique:	False
	Fields:	
	ID	Ascending
PrimaryKey		1
	Clustered:	False
	DistinctCount:	12
	Foreign:	False
	IgnoreNulls:	False
	Name:	PrimaryKey
	Primary:	True
	Required:	True
	Unique:	True
	Fields:	
	ID	Ascending
ArbeitsgruppenPflanzentransformation		1
	Clustered:	False
	DistinctCount:	1

	Foreign:	True
	IgnoreNulls:	False
	Name:	ArbeitsgruppenPflanzentransformation
	Primary:	False
	Required:	False
	Unique:	False
	Fields:	
	AG	Ascending
Id Nummer		1
	Clustered:	False
	DistinctCount:	4
	Foreign:	False
	IgnoreNulls:	False
	Name:	Id Nummer
	Primary:	False
	Required:	False
	Unique:	False
	Fields:	
	GVO Nummer	Ascending
Number of rooted LInes		1
	Clustered:	False
	DistinctCount:	3
	Foreign:	False
	IgnoreNulls:	False
	Name:	Number of rooted LInes
	Primary:	False
	Required:	False
	Unique:	False
	Fields:	
	Number of rooted LInes	Ascending
Number of Shots		1
	Clustered:	False
	DistinctCount:	2
	Foreign:	False
	IgnoreNulls:	False
	Name:	Number of Shots
	Primary:	False
	Required:	False
	Unique:	False
	Fields:	
	Number of Shots	Ascending
OperatorPflanzentransformation		1
	Clustered:	False
	DistinctCount:	2
	Foreign:	True
	IgnoreNulls:	False
	Name:	OperatorPflanzentransformation
	Primary:	False
	Required:	False
	Unique:	False
	Fields:	
	Worker	Ascending
PrimaryKey		1

Clustered:	False
DistinctCount:	7
Foreign:	False
IgnoreNulls:	False
Name:	PrimaryKey
Primary:	True
Required:	True
Unique:	True
Fields:	
ID	Ascending

Result_ID 1

Clustered:	False
DistinctCount:	2
Foreign:	False
IgnoreNulls:	False
Name:	Result_ID
Primary:	False
Required:	False
Unique:	False
Fields:	
Result_ID	Ascending

User Permissions

admin	Delete; Read Permissions; Set Permissions; Change Owner, Read Definition; Write Definition; Read Data; Insert Data; Update Data; Delete Data

Group Permissions

Admins	Delete; Read Permissions; Set Permissions; Change Owner, Read Definition; Write Definition; Read Data; Insert Data; Update Data; Delete Data
Users	Delete; Read Permissions; Set Permissions; Change Owner, Read Definition; Write Definition; Read Data; Insert Data; Update Data; Delete Data

Properties

DatasheetFontHeight:	10	DatasheetFontItalic:	False
DatasheetFontName:	Arial	DatasheetFontUnderline:	False
DatasheetFontWeight:	Normal	DatasheetForeColor:	33554432
DatasheetForeColor12:	33554432	DateCreated:	03.07.2009 09:54:26
DefaultView:	2	DOL:	Long binary data
FilterOnLoad:	False	GUID:	{guid {1BF179B5-3EE5-4BAD-A75B-08B81389D20F}}
LastUpdated:	15.10.2009 17:00:06	MaxRecords:	0
ODBCTimeout:	60	OrderByOn:	False
OrderByOnLoad:	True	Orientation:	Left-to-Right
RecordLocks:	No Locks	RecordsAffected:	0
RecordsetType:	Dynaset	ReturnsRecords:	True
RowHeight:	495	TabularCharSet:	0
TabularFamily:	34	TotalsRow:	False
Type:	0	Updatable:	True

SQL

```
SELECT Pflanzentransformation.ID, Pflanzentransformation.[GVO Nummer] AS Construct, Operator.Operator
AS Scientist, Protokoll.Protokollname AS Method, Eltern.Varietät AS CV,
Pflanzentransformation.Transformationsdatum AS Transformation, [Transformationsdatum]+[ZeitvonStart] AS
[Date], Transformationsschritte.Prozess, [Resistenz Pflanze].Resistenz AS [Selection Plant],
Pflanzentransformation.[Number of Lines]
FROM Operator INNER JOIN (([Resistenz Pflanze] RIGHT JOIN Construct ON [Resistenz Pflanze].ID =
Construct.[Plant Resistance]) INNER JOIN ((Protokoll INNER JOIN (Eltern RIGHT JOIN Pflanzentransformation
ON Eltern.ID = Pflanzentransformation.Pflanze) ON Protokoll.[Protokoll ID] =
Pflanzentransformation.Protokoll) INNER JOIN Transformationsschritte ON Protokoll.[Protokoll ID] =
Transformationsschritte.Art) ON Construct.ConstructId = Pflanzentransformation.[GVO Nummer]) ON
Operator.ID = Pflanzentransformation.Worker
WHERE ((([Transformationsdatum]+[ZeitvonStart])>=Date() And
([Transformationsdatum]+[ZeitvonStart])<=(Date()+7)) AND ((Pflanzentransformation.Enddatum) Is Null Or
(Pflanzentransformation.Enddatum)>=Date()))
ORDER BY [Transformationsdatum]+[ZeitvonStart];
```

Columns

Name	Type	Size
ID	Long Integer	4

AggregateType:	-1
AllowZeroLength:	False
AppendOnly:	False
Attributes:	Fixed Size; Auto-Increment
CollatingOrder:	General
ColumnHidden:	False
ColumnOrder:	1
ColumnWidth:	570
DataUpdatable:	False
GUID:	{guid {491011A1-F233-4D8B-AF6D-E85AC96DC00A}}
OrdinalPosition:	0
Required:	False
SourceField:	ID
SourceTable:	Pflanzentransformation
TextAlign:	General

Construct	Long Integer	4
AggregateType:	-1	
AllowZeroLength:	False	
AppendOnly:	False	
Attributes:	Fixed Size	
CollatingOrder:	General	
ColumnHidden:	False	
ColumnOrder:	7	
ColumnWidth:	975	
DataUpdatable:	False	
DecimalPlaces:	Auto	
DefaultValue:	0	
DisplayControl:	Text Box	
GUID:	{guid {78A41B5D-B257-469B-8FCD-4CB99C084269}}	
OrdinalPosition:	1	
Required:	True	
SourceField:	GVO Nummer	
SourceTable:	Pflanzentransformation	
TextAlign:	General	
Scientist	Text	50
AggregateType:	-1	
AllowZeroLength:	True	
AppendOnly:	False	
Attributes:	Variable Length	
CollatingOrder:	General	
ColumnHidden:	False	
ColumnOrder:	10	
ColumnWidth:	1230	
DataUpdatable:	False	
Description:	Familienname verantwortlicher Wissenschafter	
DisplayControl:	Text Box	
GUID:	{guid {2AA8B70A-AC14-469A-8FAC-3C278DCB2AC7}}	
IMEMode:	0	
IMESentenceMode:	3	
OrdinalPosition:	2	
Required:	False	
SourceField:	Operator	
SourceTable:	Operator	
TextAlign:	General	
UnicodeCompression:	True	
Method	Text	50
AggregateType:	-1	
AllowZeroLength:	True	
AppendOnly:	False	
Attributes:	Variable Length	
CollatingOrder:	General	
ColumnHidden:	False	
ColumnOrder:	8	
ColumnWidth:	2835	
DataUpdatable:	False	
Description:	Eindeutiger Name für das Protokoll	
DisplayControl:	Text Box	
GUID:	{guid {BA35B293-EC1A-433F-A9CA-F649675F83D2}}	
IMEMode:	0	

	IMESentenceMode:	3	
	OrdinalPosition:	3	
	Required:	False	
	SourceField:	Protokollname	
	SourceTable:	Protokoll	
	TextAlign:	General	
	UnicodeCompression:	True	
CV		Text	50
	AggregateType:	-1	
	AllowZeroLength:	True	
	AppendOnly:	False	
	Attributes:	Variable Length	
	CollatingOrder:	General	
	ColumnHidden:	False	
	ColumnOrder:	4	
	ColumnWidth:	675	
	DataUpdatable:	False	
	DisplayControl:	Text Box	
	GUID:	{guid {1AF19923-4CB9-4A37-BDF6-9A285C5D1D00}}	
	IMEMode:	0	
	IMESentenceMode:	3	
	OrdinalPosition:	4	
	Required:	False	
	SourceField:	Varietät	
	SourceTable:	Eltern	
	TextAlign:	General	
	UnicodeCompression:	True	
Transformation		Date/Time	8
	AggregateType:	-1	
	AllowZeroLength:	False	
	AppendOnly:	False	
	Attributes:	Fixed Size	
	CollatingOrder:	General	
	ColumnHidden:	False	
	ColumnOrder:	9	
	ColumnWidth:	1410	
	DataUpdatable:	False	
	GUID:	{guid {001E5420-4F1C-4D41-9502-4F9521AA6AF1}}	
	IMEMode:	0	
	IMESentenceMode:	3	
	OrdinalPosition:	5	
	Required:	False	
	ShowDatePicker:	For dates	
	SourceField:	Transformationsdatum	
	SourceTable:	Pflanzentransformation	
	TextAlign:	General	
Date		Date/Time	8
	AggregateType:	-1	
	AllowZeroLength:	False	
	AppendOnly:	False	
	Attributes:	Fixed Size	
	CollatingOrder:	General	
	ColumnHidden:	False	

	ColumnOrder:	3
	ColumnWidth:	1530
	DataUpdatable:	False
	GUID:	{guid {8577F5F1-8644-4BC6-8790-4C8AE7F791F1}}
	OrdinalPosition:	6
	Required:	False
Prozess		Text	50
	AggregateType:	-1
	AllowZeroLength:	True
	AppendOnly:	False
	Attributes:	Variable Length
	CollatingOrder:	General
	ColumnHidden:	False
	ColumnOrder:	5
	ColumnWidth:	2625
	DataUpdatable:	False
	Description:	Arbeitsprozess
	DisplayControl:	Text Box
	GUID:	{guid {F145B5A2-C291-40CF-8D68-20B48F7A3039}}
	IMEMode:	0
	IMESentenceMode:	3
	OrdinalPosition:	7
	Required:	False
	SourceField:	Prozess
	SourceTable:	Transformationsschritte
	TextAlign:	General
	UnicodeCompression:	True
Selection Plant		Text	50
	AggregateType:	-1
	AllowZeroLength:	True
	AppendOnly:	False
	Attributes:	Variable Length
	CollatingOrder:	General
	ColumnHidden:	False
	ColumnOrder:	6
	ColumnWidth:	1170
	DataUpdatable:	False
	DisplayControl:	Text Box
	GUID:	{guid {8D71BD08-D542-4B6A-8796-02E82F37D7E2}}
	IMEMode:	0
	IMESentenceMode:	3
	OrdinalPosition:	8
	Required:	False
	SourceField:	Resistenz
	SourceTable:	Resistenz Pflanze
	TextAlign:	General
	UnicodeCompression:	True
Number of Lines		Long Integer	4
	AggregateType:	-1
	AllowZeroLength:	False
	AppendOnly:	False
	Attributes:	Fixed Size
	CollatingOrder:	General

ColumnHidden:	False
ColumnOrder:	2
ColumnWidth:	510
DataUpdatable:	False
DecimalPlaces:	Auto
DefaultValue:	0
Description:	Number of Lines that have been cut (festgelegt 06.05.09)
DisplayControl:	Text Box
GUID:	{guid {85595EC7-656A-4467-A6CE-21630FBEB0B4}}
OrdinalPosition:	9
Required:	False
SourceField:	Number of Lines
SourceTable:	Pflanzentransformation
TextAlign:	General

Table Indexes

Name	Number of Fields
ArbeitsgruppenPflanzentransformation	1
Clustered:	False
DistinctCount:	1
Foreign:	True
IgnoreNulls:	False
Name:	ArbeitsgruppenPflanzentransformation
Primary:	False
Required:	False
Unique:	False
Fields:	
AG	Ascending
Id Nummer	1
Clustered:	False
DistinctCount:	4
Foreign:	False
IgnoreNulls:	False
Name:	Id Nummer
Primary:	False
Required:	False
Unique:	False
Fields:	
GVO Nummer	Ascending
Number of rooted LInes	1
Clustered:	False
DistinctCount:	3
Foreign:	False
IgnoreNulls:	False
Name:	Number of rooted LInes
Primary:	False
Required:	False
Unique:	False
Fields:	
Number of rooted LInes	Ascending
Number of Shots	1
Clustered:	False

	DistinctCount:	2
	Foreign:	False
	IgnoreNulls:	False
	Name:	Number of Shots
	Primary:	False
	Required:	False
	Unique:	False
	Fields:	
	Number of Shots	Ascending
OperatorPflanzentransformation		1
	Clustered:	False
	DistinctCount:	2
	Foreign:	True
	IgnoreNulls:	False
	Name:	OperatorPflanzentransformation
	Primary:	False
	Required:	False
	Unique:	False
	Fields:	
	Worker	Ascending
PrimaryKey		1
	Clustered:	False
	DistinctCount:	7
	Foreign:	False
	IgnoreNulls:	False
	Name:	PrimaryKey
	Primary:	True
	Required:	True
	Unique:	True
	Fields:	
	ID	Ascending
Result_ID		1
	Clustered:	False
	DistinctCount:	2
	Foreign:	False
	IgnoreNulls:	False
	Name:	Result_ID
	Primary:	False
	Required:	False
	Unique:	False
	Fields:	
	Result_ID	Ascending
ID		1
	Clustered:	False
	DistinctCount:	2
	Foreign:	False
	IgnoreNulls:	False
	Name:	ID
	Primary:	False
	Required:	False
	Unique:	False
	Fields:	
	ID	Ascending

ID_LIMS		1
	Clustered:	False
	DistinctCount:	2
	Foreign:	False
	IgnoreNulls:	False
	Name:	ID_LIMS
	Primary:	False
	Required:	False
	Unique:	False
	Fields:	
	ID_LIMS	Ascending
PrimaryKey		1
	Clustered:	False
	DistinctCount:	2
	Foreign:	False
	IgnoreNulls:	False
	Name:	PrimaryKey
	Primary:	True
	Required:	True
	Unique:	True
	Fields:	
	ID	Ascending
PrimaryKey		1
	Clustered:	False
	DistinctCount:	3
	Foreign:	False
	IgnoreNulls:	False
	Name:	PrimaryKey
	Primary:	True
	Required:	True
	Unique:	True
	Fields:	
	Protokoll ID	Ascending
ProtokollArt		1
	Clustered:	False
	DistinctCount:	1
	Foreign:	False
	IgnoreNulls:	False
	Name:	ProtokollArt
	Primary:	False
	Required:	False
	Unique:	False
	Fields:	
	Art	Ascending
GMO Nummer		1
	Clustered:	False
	DistinctCount:	15
	Foreign:	False
	IgnoreNulls:	False
	Name:	GMO Nummer
	Primary:	False
	Required:	False
	Unique:	False

	Fields:	
	GMO Nummer	Ascending
ID		1
	Clustered:	False
	DistinctCount:	28
	Foreign:	False
	IgnoreNulls:	False
	Name:	ID
	Primary:	False
	Required:	False
	Unique:	False
	Fields:	
	ID	Ascending
ID_Pflanzenarten_Ref		1
	Clustered:	False
	DistinctCount:	4
	Foreign:	False
	IgnoreNulls:	False
	Name:	ID_Pflanzenarten_Ref
	Primary:	False
	Required:	False
	Unique:	False
	Fields:	
	ID_Pflanzenarten_Ref	Ascending
PrimaryKey		1
	Clustered:	False
	DistinctCount:	28
	Foreign:	False
	IgnoreNulls:	False
	Name:	PrimaryKey
	Primary:	True
	Required:	True
	Unique:	True
	Fields:	
	ID	Ascending
ArbeitsgruppenPflanzentransformation		1
	Clustered:	False
	DistinctCount:	1
	Foreign:	True
	IgnoreNulls:	False
	Name:	ArbeitsgruppenPflanzentransformation
	Primary:	False
	Required:	False
	Unique:	False
	Fields:	
	AG	Ascending
Id Nummer		1
	Clustered:	False
	DistinctCount:	4
	Foreign:	False
	IgnoreNulls:	False
	Name:	Id Nummer
	Primary:	False

	Required:	False
	Unique:	False
	Fields:	
	GVO Nummer	Ascending
Number of rooted LInes		1
	Clustered:	False
	DistinctCount:	3
	Foreign:	False
	IgnoreNulls:	False
	Name:	Number of rooted LInes
	Primary:	False
	Required:	False
	Unique:	False
	Fields:	
	Number of rooted LInes	Ascending
Number of Shots		1
	Clustered:	False
	DistinctCount:	2
	Foreign:	False
	IgnoreNulls:	False
	Name:	Number of Shots
	Primary:	False
	Required:	False
	Unique:	False
	Fields:	
	Number of Shots	Ascending
OperatorPflanzentransformation		1
	Clustered:	False
	DistinctCount:	2
	Foreign:	True
	IgnoreNulls:	False
	Name:	OperatorPflanzentransformation
	Primary:	False
	Required:	False
	Unique:	False
	Fields:	
	Worker	Ascending
PrimaryKey		1
	Clustered:	False
	DistinctCount:	7
	Foreign:	False
	IgnoreNulls:	False
	Name:	PrimaryKey
	Primary:	True
	Required:	True
	Unique:	True
	Fields:	
	ID	Ascending
Result_ID		1
	Clustered:	False
	DistinctCount:	2
	Foreign:	False
	IgnoreNulls:	False

	Name:	Result_ID
	Primary:	False
	Required:	False
	Unique:	False
	Fields:	
	Result_ID	Ascending
Old_ID		1
	Clustered:	False
	DistinctCount:	4
	Foreign:	False
	IgnoreNulls:	False
	Name:	Old_ID
	Primary:	False
	Required:	False
	Unique:	False
	Fields:	
	Old_ID	Ascending
PrimaryKey		1
	Clustered:	False
	DistinctCount:	9
	Foreign:	False
	IgnoreNulls:	False
	Name:	PrimaryKey
	Primary:	True
	Required:	True
	Unique:	True
	Fields:	
	ID	Ascending
ID		1
	Clustered:	False
	DistinctCount:	12
	Foreign:	False
	IgnoreNulls:	False
	Name:	ID
	Primary:	False
	Required:	False
	Unique:	False
	Fields:	
	ID	Ascending
PrimaryKey		1
	Clustered:	False
	DistinctCount:	12
	Foreign:	False
	IgnoreNulls:	False
	Name:	PrimaryKey
	Primary:	True
	Required:	True
	Unique:	True
	Fields:	
	ID	Ascending
ArbeitsgruppenPflanzentransformation		1
	Clustered:	False
	DistinctCount:	1

	Foreign:	True
	IgnoreNulls:	False
	Name:	ArbeitsgruppenPflanzentransformation
	Primary:	False
	Required:	False
	Unique:	False
	Fields:	
	AG	Ascending
Id Nummer		1
	Clustered:	False
	DistinctCount:	4
	Foreign:	False
	IgnoreNulls:	False
	Name:	Id Nummer
	Primary:	False
	Required:	False
	Unique:	False
	Fields:	
	GVO Nummer	Ascending
Number of rooted LInes		1
	Clustered:	False
	DistinctCount:	3
	Foreign:	False
	IgnoreNulls:	False
	Name:	Number of rooted LInes
	Primary:	False
	Required:	False
	Unique:	False
	Fields:	
	Number of rooted LInes	Ascending
Number of Shots		1
	Clustered:	False
	DistinctCount:	2
	Foreign:	False
	IgnoreNulls:	False
	Name:	Number of Shots
	Primary:	False
	Required:	False
	Unique:	False
	Fields:	
	Number of Shots	Ascending
OperatorPflanzentransformation		1
	Clustered:	False
	DistinctCount:	2
	Foreign:	True
	IgnoreNulls:	False
	Name:	OperatorPflanzentransformation
	Primary:	False
	Required:	False
	Unique:	False
	Fields:	
	Worker	Ascending
PrimaryKey		1

Clustered:	False
DistinctCount:	7
Foreign:	False
IgnoreNulls:	False
Name:	PrimaryKey
Primary:	True
Required:	True
Unique:	True
Fields:	
ID	Ascending

Result_ID 1

Clustered:	False
DistinctCount:	2
Foreign:	False
IgnoreNulls:	False
Name:	Result_ID
Primary:	False
Required:	False
Unique:	False
Fields:	
Result_ID	Ascending

User Permissions

admin	Delete; Read Permissions; Set Permissions; Change Owner, Read Definition; Write Definition; Read Data; Insert Data; Update Data; Delete Data

Group Permissions

Admins	Delete; Read Permissions; Set Permissions; Change Owner, Read Definition; Write Definition; Read Data; Insert Data; Update Data; Delete Data
Users	Delete; Read Permissions; Set Permissions; Change Owner, Read Definition; Write Definition; Read Data; Insert Data; Update Data; Delete Data

Properties

DatasheetFontHeight:	10	DatasheetFontItalic:	False
DatasheetFontName:	Arial	DatasheetFontUnderline:	False
DatasheetFontWeight:	Normal	DatasheetForeColor:	33554432
DatasheetForeColor12:	33554432	DateCreated:	03.07.2009 09:54:26
DefaultView:	2	DOL:	Long binary data
FilterOnLoad:	False	GUID:	{guid {8CEC9363-0D91-47B1-B965-BC0D3D34BD0F}}
LastUpdated:	08.01.2010 18:13:37	MaxRecords:	0
ODBCTimeout:	60	OrderByOn:	False
OrderByOnLoad:	True	Orientation:	Left-to-Right
RecordLocks:	No Locks	RecordsAffected:	0
RecordsetType:	Dynaset	ReturnsRecords:	True
RowHeight:	495	TabularCharSet:	0
TabularFamily:	34	TotalsRow:	False
Type:	0	Updatable:	True

SQL

```
SELECT Pflanzentransformation.ID, Pflanzentransformation.[GVO Nummer] AS [Construct Id],
Operator.Operator, Protokoll.Protokollname AS Protocol, Eltern.Varietät AS Variety,
Pflanzentransformation.Transformationsdatum AS [Transformation date],
[Transformationsdatum]+[ZeitvonStart] AS Date7, Transformationsschritte.Prozess AS Step, [Resistenz
Pflanze].Resistenz AS Resistance, Pflanzentransformation.[Number of Lines]
FROM Operator INNER JOIN (([Resistenz Pflanze] RIGHT JOIN Construct ON [Resistenz Pflanze].ID =
Construct.[Plant Resistance]) INNER JOIN ((Protokoll INNER JOIN (Eltern RIGHT JOIN Pflanzentransformation
ON Eltern.ID = Pflanzentransformation.Pflanze) ON Protokoll.[Protokoll ID] =
Pflanzentransformation.Protokoll) INNER JOIN Transformationsschritte ON Protokoll.[Protokoll ID] =
Transformationsschritte.Art) ON Construct.ConstructId = Pflanzentransformation.[GVO Nummer]) ON
Operator.ID = Pflanzentransformation.Worker
WHERE ((([Transformationsdatum]+[ZeitvonStart])>=Date() And
([Transformationsdatum]+[ZeitvonStart])<=(Date()+9)) AND ((Pflanzentransformation.Enddatum) Is Null Or
(Pflanzentransformation.Enddatum)>=Date()))
ORDER BY [Transformationsdatum]+[ZeitvonStart];
```

Columns

Name	Type	Size
ID	Long Integer	4

AggregateType:	-1
AllowZeroLength:	False
AppendOnly:	False
Attributes:	Fixed Size; Auto-Increment
CollatingOrder:	General
ColumnHidden:	False
ColumnOrder:	1
ColumnWidth:	570
DataUpdatable:	False
GUID:	{guid {491011A1-F233-4D8B-AF6D-E85AC96DC00A}}
OrdinalPosition:	0
Required:	False
SourceField:	ID
SourceTable:	Pflanzentransformation
TextAlign:	General

Construct Id Long Integer 4

AggregateType: -1
AllowZeroLength: False
AppendOnly: False
Attributes: Fixed Size
CollatingOrder: General
ColumnHidden: False
ColumnOrder: 5
ColumnWidth: 945
DataUpdatable: False
DecimalPlaces: Auto
DefaultValue: 0
DisplayControl: Text Box
GUID: {guid {78A41B5D-B257-469B-8FCD-4CB99C084269}}
OrdinalPosition: 1
Required: True
SourceField: GVO Nummer
SourceTable: Pflanzentransformation
TextAlign: General

Operator Text 50

AggregateType: -1
AllowZeroLength: True
AppendOnly: False
Attributes: Variable Length
CollatingOrder: General
ColumnHidden: False
ColumnOrder: 9
ColumnWidth: 1620
DataUpdatable: False
Description: Familienname verantwortlicher Wissenschafter
DisplayControl: Text Box
GUID: {guid {2AA8B70A-AC14-469A-8FAC-3C278DCB2AC7}}
IMEMode: 0
IMESentenceMode: 3
OrdinalPosition: 2
Required: False
SourceField: Operator
SourceTable: Operator
TextAlign: General
UnicodeCompression: True

Protocol Text 50

AggregateType: -1
AllowZeroLength: True
AppendOnly: False
Attributes: Variable Length
CollatingOrder: General
ColumnHidden: False
ColumnOrder: 6
ColumnWidth: 2835
DataUpdatable: False
Description: Eindeutiger Name für das Protokoll
DisplayControl: Text Box
GUID: {guid {BA35B293-EC1A-433F-A9CA-F649675F83D2}}
IMEMode: 0

IMESentenceMode:	3	
OrdinalPosition:	3	
Required:	False	
SourceField:	Protokollname	
SourceTable:	Protokoll	
TextAlign:	General	
UnicodeCompression:	True	

Variety	Text	50
AggregateType:	-1	
AllowZeroLength:	True	
AppendOnly:	False	
Attributes:	Variable Length	
CollatingOrder:	General	
ColumnHidden:	False	
ColumnOrder:	3	
ColumnWidth:	1095	
DataUpdatable:	False	
DisplayControl:	Text Box	
GUID:	{guid {1AF19923-4CB9-4A37-BDF6-9A285C5D1D00}}	
IMEMode:	0	
IMESentenceMode:	3	
OrdinalPosition:	4	
Required:	False	
SourceField:	Varietät	
SourceTable:	Eltern	
TextAlign:	General	
UnicodeCompression:	True	

Transformation date	Date/Time	8
AggregateType:	-1	
AllowZeroLength:	False	
AppendOnly:	False	
Attributes:	Fixed Size	
CollatingOrder:	General	
ColumnHidden:	False	
ColumnOrder:	8	
ColumnWidth:	1410	
DataUpdatable:	False	
GUID:	{guid {001E5420-4F1C-4D41-9502-4F9521AA6AF1}}	
IMEMode:	0	
IMESentenceMode:	3	
OrdinalPosition:	5	
Required:	False	
ShowDatePicker:	For dates	
SourceField:	Transformationsdatum	
SourceTable:	Pflanzentransformation	
TextAlign:	General	

Date7	Date/Time	8
AggregateType:	-1	
AllowZeroLength:	False	
AppendOnly:	False	
Attributes:	Fixed Size	
CollatingOrder:	General	
ColumnHidden:	False	

	ColumnOrder:	2
	ColumnWidth:	1530
	DataUpdatable:	False
	GUID:	{guid {3074E61C-0000-0000-1CE6-743000000000}}
	OrdinalPosition:	6
	Required:	False
Step		Text 50
	AggregateType:	-1
	AllowZeroLength:	True
	AppendOnly:	False
	Attributes:	Variable Length
	CollatingOrder:	General
	ColumnHidden:	False
	ColumnOrder:	4
	ColumnWidth:	2625
	DataUpdatable:	False
	Description:	Arbeitsprozess
	DisplayControl:	Text Box
	GUID:	{guid {F145B5A2-C291-40CF-8D68-20B48F7A3039}}
	IMEMode:	0
	IMESentenceMode:	3
	OrdinalPosition:	7
	Required:	False
	SourceField:	Prozess
	SourceTable:	Transformationsschritte
	TextAlign:	General
	UnicodeCompression:	True
Resistance		Text 50
	AggregateType:	-1
	AllowZeroLength:	True
	AppendOnly:	False
	Attributes:	Variable Length
	CollatingOrder:	General
	ColumnHidden:	False
	ColumnOrder:	7
	ColumnWidth:	570
	DataUpdatable:	False
	DisplayControl:	Text Box
	GUID:	{guid {8D71BD08-D542-4B6A-8796-02E82F37D7E2}}
	IMEMode:	0
	IMESentenceMode:	3
	OrdinalPosition:	8
	Required:	False
	SourceField:	Resistenz
	SourceTable:	Resistenz Pflanze
	TextAlign:	General
	UnicodeCompression:	True
Number of Lines		Long Integer 4
	AggregateType:	-1
	AllowZeroLength:	False
	AppendOnly:	False
	Attributes:	Fixed Size
	CollatingOrder:	General

ColumnHidden:	False
ColumnOrder:	Default
ColumnWidth:	Default
DataUpdatable:	False
DecimalPlaces:	Auto
DefaultValue:	0
Description:	Number of Lines that have been cut (festgelegt 06.05.09)
DisplayControl:	Text Box
GUID:	{guid {85595EC7-656A-4467-A6CE-21630FBEB0B4}}
OrdinalPosition:	9
Required:	False
SourceField:	Number of Lines
SourceTable:	Pflanzentransformation
TextAlign:	General

Table Indexes

Name		Number of Fields
ArbeitsgruppenPflanzentransformation		1
	Clustered:	False
	DistinctCount:	1
	Foreign:	True
	IgnoreNulls:	False
	Name:	ArbeitsgruppenPflanzentransformation
	Primary:	False
	Required:	False
	Unique:	False
	Fields:	
	AG	Ascending
Id Nummer		1
	Clustered:	False
	DistinctCount:	4
	Foreign:	False
	IgnoreNulls:	False
	Name:	Id Nummer
	Primary:	False
	Required:	False
	Unique:	False
	Fields:	
	GVO Nummer	Ascending
Number of rooted LInes		1
	Clustered:	False
	DistinctCount:	3
	Foreign:	False
	IgnoreNulls:	False
	Name:	Number of rooted LInes
	Primary:	False
	Required:	False
	Unique:	False
	Fields:	
	Number of rooted LInes	Ascending
Number of Shots		1
	Clustered:	False

	DistinctCount:	2
	Foreign:	False
	IgnoreNulls:	False
	Name:	Number of Shots
	Primary:	False
	Required:	False
	Unique:	False
	Fields:	
	Number of Shots	Ascending
OperatorPflanzentransformation		1
	Clustered:	False
	DistinctCount:	2
	Foreign:	True
	IgnoreNulls:	False
	Name:	OperatorPflanzentransformation
	Primary:	False
	Required:	False
	Unique:	False
	Fields:	
	Worker	Ascending
PrimaryKey		1
	Clustered:	False
	DistinctCount:	7
	Foreign:	False
	IgnoreNulls:	False
	Name:	PrimaryKey
	Primary:	True
	Required:	True
	Unique:	True
	Fields:	
	ID	Ascending
Result_ID		1
	Clustered:	False
	DistinctCount:	2
	Foreign:	False
	IgnoreNulls:	False
	Name:	Result_ID
	Primary:	False
	Required:	False
	Unique:	False
	Fields:	
	Result_ID	Ascending
ID		1
	Clustered:	False
	DistinctCount:	2
	Foreign:	False
	IgnoreNulls:	False
	Name:	ID
	Primary:	False
	Required:	False
	Unique:	False
	Fields:	
	ID	Ascending

ID_LIMS		1
	Clustered:	False
	DistinctCount:	2
	Foreign:	False
	IgnoreNulls:	False
	Name:	ID_LIMS
	Primary:	False
	Required:	False
	Unique:	False
	Fields:	
	ID_LIMS	Ascending
PrimaryKey		1
	Clustered:	False
	DistinctCount:	2
	Foreign:	False
	IgnoreNulls:	False
	Name:	PrimaryKey
	Primary:	True
	Required:	True
	Unique:	True
	Fields:	
	ID	Ascending
PrimaryKey		1
	Clustered:	False
	DistinctCount:	3
	Foreign:	False
	IgnoreNulls:	False
	Name:	PrimaryKey
	Primary:	True
	Required:	True
	Unique:	True
	Fields:	
	Protokoll ID	Ascending
ProtokollArt		1
	Clustered:	False
	DistinctCount:	1
	Foreign:	False
	IgnoreNulls:	False
	Name:	ProtokollArt
	Primary:	False
	Required:	False
	Unique:	False
	Fields:	
	Art	Ascending
GMO Nummer		1
	Clustered:	False
	DistinctCount:	15
	Foreign:	False
	IgnoreNulls:	False
	Name:	GMO Nummer
	Primary:	False
	Required:	False
	Unique:	False

	Fields:	
	GMO Nummer	Ascending
ID		1
	Clustered:	False
	DistinctCount:	28
	Foreign:	False
	IgnoreNulls:	False
	Name:	ID
	Primary:	False
	Required:	False
	Unique:	False
	Fields:	
	ID	Ascending
ID_Pflanzenarten_Ref		1
	Clustered:	False
	DistinctCount:	4
	Foreign:	False
	IgnoreNulls:	False
	Name:	ID_Pflanzenarten_Ref
	Primary:	False
	Required:	False
	Unique:	False
	Fields:	
	ID_Pflanzenarten_Ref	Ascending
PrimaryKey		1
	Clustered:	False
	DistinctCount:	28
	Foreign:	False
	IgnoreNulls:	False
	Name:	PrimaryKey
	Primary:	True
	Required:	True
	Unique:	True
	Fields:	
	ID	Ascending
ArbeitsgruppenPflanzentransformation		1
	Clustered:	False
	DistinctCount:	1
	Foreign:	True
	IgnoreNulls:	False
	Name:	ArbeitsgruppenPflanzentransformation
	Primary:	False
	Required:	False
	Unique:	False
	Fields:	
	AG	Ascending
Id Nummer		1
	Clustered:	False
	DistinctCount:	4
	Foreign:	False
	IgnoreNulls:	False
	Name:	Id Nummer
	Primary:	False

	Required:	False
	Unique:	False
	Fields:	
	GVO Nummer	Ascending
Number of rooted LInes		1
	Clustered:	False
	DistinctCount:	3
	Foreign:	False
	IgnoreNulls:	False
	Name:	Number of rooted LInes
	Primary:	False
	Required:	False
	Unique:	False
	Fields:	
	Number of rooted LInes	Ascending
Number of Shots		1
	Clustered:	False
	DistinctCount:	2
	Foreign:	False
	IgnoreNulls:	False
	Name:	Number of Shots
	Primary:	False
	Required:	False
	Unique:	False
	Fields:	
	Number of Shots	Ascending
OperatorPflanzentransformation		1
	Clustered:	False
	DistinctCount:	2
	Foreign:	True
	IgnoreNulls:	False
	Name:	OperatorPflanzentransformation
	Primary:	False
	Required:	False
	Unique:	False
	Fields:	
	Worker	Ascending
PrimaryKey		1
	Clustered:	False
	DistinctCount:	7
	Foreign:	False
	IgnoreNulls:	False
	Name:	PrimaryKey
	Primary:	True
	Required:	True
	Unique:	True
	Fields:	
	ID	Ascending
Result_ID		1
	Clustered:	False
	DistinctCount:	2
	Foreign:	False
	IgnoreNulls:	False

	Name:	Result_ID
	Primary:	False
	Required:	False
	Unique:	False
	Fields:	
	Result_ID	Ascending
Old_ID		1
	Clustered:	False
	DistinctCount:	4
	Foreign:	False
	IgnoreNulls:	False
	Name:	Old_ID
	Primary:	False
	Required:	False
	Unique:	False
	Fields:	
	Old_ID	Ascending
PrimaryKey		1
	Clustered:	False
	DistinctCount:	9
	Foreign:	False
	IgnoreNulls:	False
	Name:	PrimaryKey
	Primary:	True
	Required:	True
	Unique:	True
	Fields:	
	ID	Ascending
ID		1
	Clustered:	False
	DistinctCount:	12
	Foreign:	False
	IgnoreNulls:	False
	Name:	ID
	Primary:	False
	Required:	False
	Unique:	False
	Fields:	
	ID	Ascending
PrimaryKey		1
	Clustered:	False
	DistinctCount:	12
	Foreign:	False
	IgnoreNulls:	False
	Name:	PrimaryKey
	Primary:	True
	Required:	True
	Unique:	True
	Fields:	
	ID	Ascending
ArbeitsgruppenPflanzentransformation		1
	Clustered:	False
	DistinctCount:	1

	Foreign:	True
	IgnoreNulls:	False
	Name:	ArbeitsgruppenPflanzentransformation
	Primary:	False
	Required:	False
	Unique:	False
	Fields:	
	AG	Ascending
Id Nummer		1
	Clustered:	False
	DistinctCount:	4
	Foreign:	False
	IgnoreNulls:	False
	Name:	Id Nummer
	Primary:	False
	Required:	False
	Unique:	False
	Fields:	
	GVO Nummer	Ascending
Number of rooted LInes		1
	Clustered:	False
	DistinctCount:	3
	Foreign:	False
	IgnoreNulls:	False
	Name:	Number of rooted LInes
	Primary:	False
	Required:	False
	Unique:	False
	Fields:	
	Number of rooted LInes	Ascending
Number of Shots		1
	Clustered:	False
	DistinctCount:	2
	Foreign:	False
	IgnoreNulls:	False
	Name:	Number of Shots
	Primary:	False
	Required:	False
	Unique:	False
	Fields:	
	Number of Shots	Ascending
OperatorPflanzentransformation		1
	Clustered:	False
	DistinctCount:	2
	Foreign:	True
	IgnoreNulls:	False
	Name:	OperatorPflanzentransformation
	Primary:	False
	Required:	False
	Unique:	False
	Fields:	
	Worker	Ascending
PrimaryKey		1

Clustered:	False
DistinctCount:	7
Foreign:	False
IgnoreNulls:	False
Name:	PrimaryKey
Primary:	True
Required:	True
Unique:	True
Fields:	
ID	Ascending

Result_ID 1

Clustered:	False
DistinctCount:	2
Foreign:	False
IgnoreNulls:	False
Name:	Result_ID
Primary:	False
Required:	False
Unique:	False
Fields:	
Result_ID	Ascending

User Permissions

admin	Delete; Read Permissions; Set Permissions; Change Owner, Read Definition; Write Definition; Read Data; Insert Data; Update Data; Delete Data

Group Permissions

Admins	Delete; Read Permissions; Set Permissions; Change Owner, Read Definition; Write Definition; Read Data; Insert Data; Update Data; Delete Data
Users	Delete; Read Permissions; Set Permissions; Change Owner, Read Definition; Write Definition; Read Data; Insert Data; Update Data; Delete Data

Properties

DateCreated:	14.04.2009 16:38:59	DefaultView:	2
DOL:	Long binary data	FilterOnLoad:	False
GUID:	{guid {30CC39D6-7810-4FA9-8F9E-913714E441D2}}	LastUpdated:	26.10.2009 16:57:48
MaxRecords:	0	ODBCTimeout:	60
OrderByOn:	False	OrderByOnLoad:	True
Orientation:	Left-to-Right	RecordLocks:	Edited Record
RecordsAffected:	0	RecordsetType:	Dynaset
ReturnsRecords:	True	TotalsRow:	False
Type:	80	Updatable:	True
UseTransaction:	True		

SQL

```
SELECT Pflanzentransformation.ID AS Trafoid, Pflanzentransformation.[GVO Nummer], Operator.Operator,
Pflanzentransformation.Transformationsdatum, [Transformationsdatum]+[ZeitvonStart] AS Datum,
Transformationsschritte.Prozess, Protokoll.Protokollname, ([Medien ID]*1) AS Experimentmedienid, [Resistenz
Pflanze].Resistenz, Transformationsschritte.Bemerkungen INTO Laborbuch
FROM (Operator INNER JOIN ([Resistenz Pflanze] INNER JOIN ((Protokoll INNER JOIN Pflanzentransformation
ON Protokoll.[Protokoll ID] = Pflanzentransformation.Protokoll) INNER JOIN Transformationsschritte ON
Protokoll.[Protokoll ID] = Transformationsschritte.Art) ON [Resistenz Pflanze].ID =
Pflanzentransformation.[Resistenzmarker Pflanze]) ON Operator.ID = Pflanzentransformation.Worker) INNER
JOIN (Medien INNER JOIN Media_Step ON Medien.[Medien ID] = Media_Step.Media_id) ON
Transformationsschritte.ID = Media_Step.Trafostep_id
ORDER BY [Transformationsdatum]+[ZeitvonStart];
```

User Permissions

admin	Delete; Read Permissions; Set Permissions; Change Owner, Read Definition; Write Definition; Read Data; Insert Data; Update Data; Delete Data

Group Permissions

Admins	Delete; Read Permissions; Set Permissions; Change Owner, Read Definition; Write Definition; Read Data; Insert Data; Update Data; Delete Data
Users	Delete; Read Permissions; Set Permissions; Change Owner, Read Definition; Write Definition; Read Data; Insert Data; Update Data; Delete Data

Properties

DatasheetFontHeight:	10	DatasheetFontItalic:	False
DatasheetFontName:	Arial	DatasheetFontUnderline:	False
DatasheetFontWeight:	Normal	DatasheetForeColor:	33554432
DatasheetForeColor12:	33554432	DateCreated:	03.07.2009 09:54:26
DefaultView:	2	DOL:	Long binary data
FilterOnLoad:	False	GUID:	{guid {BED6B32D-5153-4A63-AA2C-8A191CDF0EEE}}
LastUpdated:	09.10.2009 15:55:34	MaxRecords:	0
ODBCTimeout:	60	OrderByOn:	False
OrderByOnLoad:	True	Orientation:	Left-to-Right
RecordLocks:	No Locks	RecordsAffected:	0
RecordsetType:	Dynaset	ReturnsRecords:	True
RowHeight:	495	TabularCharSet:	0
TabularFamily:	34	TotalsRow:	False
Type:	0	Updatable:	True

SQL

```
SELECT Pflanzentransformation.ID, Pflanzentransformation.[GVO Nummer], Operator.Operator,
Protokoll.Protokollname, Eltern.Varietät, Pflanzentransformation.Transformationsdatum,
[Transformationsdatum]+[ZeitvonStart] AS Datum, Transformationsschritte.Prozess, [Resistenz
Pflanze].Resistenz
FROM Operator INNER JOIN (([Resistenz Pflanze] RIGHT JOIN Construct ON [Resistenz Pflanze].ID =
Construct.[Plant Resistance]) INNER JOIN ((Protokoll INNER JOIN (Eltern RIGHT JOIN Pflanzentransformation
ON Eltern.ID = Pflanzentransformation.Pflanze) ON Protokoll.[Protokoll ID] =
Pflanzentransformation.Protokoll) INNER JOIN Transformationsschritte ON Protokoll.[Protokoll ID] =
Transformationsschritte.Art) ON Construct.ConstructId = Pflanzentransformation.[GVO Nummer]) ON
Operator.ID = Pflanzentransformation.Worker
WHERE ((([Transformationsdatum]+[ZeitvonStart])=Date()) AND ((Pflanzentransformation.Enddatum) Is Null
Or (Pflanzentransformation.Enddatum)>=Date()) AND ((Pflanzentransformation.TA)="Brigitte Buchwald"))
ORDER BY [Transformationsdatum]+[ZeitvonStart];
```

Columns

Name	Type	Size
ID	Long Integer	4

AggregateType:	-1
AllowZeroLength:	False
AppendOnly:	False
Attributes:	Fixed Size; Auto-Increment
CollatingOrder:	General
ColumnHidden:	False
ColumnOrder:	1
ColumnWidth:	570
DataUpdatable:	False
GUID:	{guid {491011A1-F233-4D8B-AF6D-E85AC96DC00A}}
OrdinalPosition:	0
Required:	False
SourceField:	ID
SourceTable:	Pflanzentransformation
TextAlign:	General

Name	Type	Size
GVO Nummer	Long Integer	4

AggregateType:	-1
AllowZeroLength:	False
AppendOnly:	False
Attributes:	Fixed Size
CollatingOrder:	General
ColumnHidden:	False
ColumnOrder:	5
ColumnWidth:	1005
DataUpdatable:	False
DecimalPlaces:	Auto
DefaultValue:	0
DisplayControl:	Text Box
GUID:	{guid {78A41B5D-B257-469B-8FCD-4CB99C084269}}
OrdinalPosition:	1
Required:	True
SourceField:	GVO Nummer
SourceTable:	Pflanzentransformation
TextAlign:	General

Operator Text 50

AggregateType:	-1
AllowZeroLength:	True
AppendOnly:	False
Attributes:	Variable Length
CollatingOrder:	General
ColumnHidden:	False
ColumnOrder:	9
ColumnWidth:	1620
DataUpdatable:	False
Description:	Familienname verantwortlicher Wissenschafter
DisplayControl:	Text Box
GUID:	{guid {2AA8B70A-AC14-469A-8FAC-3C278DCB2AC7}}
IMEMode:	0
IMESentenceMode:	3
OrdinalPosition:	2
Required:	False
SourceField:	Operator
SourceTable:	Operator
TextAlign:	General
UnicodeCompression:	True

Protokollname Text 50

AggregateType:	-1
AllowZeroLength:	True
AppendOnly:	False
Attributes:	Variable Length
CollatingOrder:	General
ColumnHidden:	False
ColumnOrder:	6
ColumnWidth:	3120
DataUpdatable:	False
Description:	Eindeutiger Name für das Protokoll
DisplayControl:	Text Box
GUID:	{guid {BA35B293-EC1A-433F-A9CA-F649675F83D2}}
IMEMode:	0
IMESentenceMode:	3

OrdinalPosition:	3	
Required:	False	
SourceField:	Protokollname	
SourceTable:	Protokoll	
TextAlign:	General	
UnicodeCompression:	True	

Varietät	Text	50
AggregateType:	-1	
AllowZeroLength:	True	
AppendOnly:	False	
Attributes:	Variable Length	
CollatingOrder:	General	
ColumnHidden:	False	
ColumnOrder:	3	
ColumnWidth:	1245	
DataUpdatable:	False	
DisplayControl:	Text Box	
GUID:	{guid {1AF19923-4CB9-4A37-BDF6-9A285C5D1D00}}	
IMEMode:	0	
IMESentenceMode:	3	
OrdinalPosition:	4	
Required:	False	
SourceField:	Varietät	
SourceTable:	Eltern	
TextAlign:	General	
UnicodeCompression:	True	

Transformationsdatum	Date/Time	8
AggregateType:	-1	
AllowZeroLength:	False	
AppendOnly:	False	
Attributes:	Fixed Size	
CollatingOrder:	General	
ColumnHidden:	False	
ColumnOrder:	8	
ColumnWidth:	1410	
DataUpdatable:	False	
GUID:	{guid {001E5420-4F1C-4D41-9502-4F9521AA6AF1}}	
IMEMode:	0	
IMESentenceMode:	3	
OrdinalPosition:	5	
Required:	False	
ShowDatePicker:	For dates	
SourceField:	Transformationsdatum	
SourceTable:	Pflanzentransformation	
TextAlign:	General	

Datum	Date/Time	8
AggregateType:	-1	
AllowZeroLength:	False	
AppendOnly:	False	
Attributes:	Fixed Size	
CollatingOrder:	General	
ColumnHidden:	False	
ColumnOrder:	2	

ColumnWidth:	1530	
DataUpdatable:	False	
GUID:	{guid {8EA3B6E5-518B-4887-8283-3402A7CD7985}}	
OrdinalPosition:	6	
Required:	False	

Prozess	Text	50
AggregateType:	-1	
AllowZeroLength:	True	
AppendOnly:	False	
Attributes:	Variable Length	
CollatingOrder:	General	
ColumnHidden:	False	
ColumnOrder:	4	
ColumnWidth:	2625	
DataUpdatable:	False	
Description:	Arbeitsprozess	
DisplayControl:	Text Box	
GUID:	{guid {F145B5A2-C291-40CF-8D68-20B48F7A3039}}	
IMEMode:	0	
IMESentenceMode:	3	
OrdinalPosition:	7	
Required:	False	
SourceField:	Prozess	
SourceTable:	Transformationsschritte	
TextAlign:	General	
UnicodeCompression:	True	

Resistenz	Text	50
AggregateType:	-1	
AllowZeroLength:	True	
AppendOnly:	False	
Attributes:	Variable Length	
CollatingOrder:	General	
ColumnHidden:	False	
ColumnOrder:	7	
ColumnWidth:	645	
DataUpdatable:	False	
DisplayControl:	Text Box	
GUID:	{guid {8D71BD08-D542-4B6A-8796-02E82F37D7E2}}	
IMEMode:	0	
IMESentenceMode:	3	
OrdinalPosition:	8	
Required:	False	
SourceField:	Resistenz	
SourceTable:	Resistenz Pflanze	
TextAlign:	General	
UnicodeCompression:	True	

Table Indexes

Name	Number of Fields
ArbeitsgruppenPflanzentransformation	1
Clustered:	False
DistinctCount:	1

	Foreign:	True
	IgnoreNulls:	False
	Name:	ArbeitsgruppenPflanzentransformation
	Primary:	False
	Required:	False
	Unique:	False
	Fields:	
	AG	Ascending
Id Nummer		1
	Clustered:	False
	DistinctCount:	4
	Foreign:	False
	IgnoreNulls:	False
	Name:	Id Nummer
	Primary:	False
	Required:	False
	Unique:	False
	Fields:	
	GVO Nummer	Ascending
Number of rooted LInes		1
	Clustered:	False
	DistinctCount:	3
	Foreign:	False
	IgnoreNulls:	False
	Name:	Number of rooted LInes
	Primary:	False
	Required:	False
	Unique:	False
	Fields:	
	Number of rooted LInes	Ascending
Number of Shots		1
	Clustered:	False
	DistinctCount:	2
	Foreign:	False
	IgnoreNulls:	False
	Name:	Number of Shots
	Primary:	False
	Required:	False
	Unique:	False
	Fields:	
	Number of Shots	Ascending
OperatorPflanzentransformation		1
	Clustered:	False
	DistinctCount:	2
	Foreign:	True
	IgnoreNulls:	False
	Name:	OperatorPflanzentransformation
	Primary:	False
	Required:	False
	Unique:	False
	Fields:	
	Worker	Ascending
PrimaryKey		1

	Clustered:	False
	DistinctCount:	7
	Foreign:	False
	IgnoreNulls:	False
	Name:	PrimaryKey
	Primary:	True
	Required:	True
	Unique:	True
	Fields:	
	ID	Ascending
Result_ID		1
	Clustered:	False
	DistinctCount:	2
	Foreign:	False
	IgnoreNulls:	False
	Name:	Result_ID
	Primary:	False
	Required:	False
	Unique:	False
	Fields:	
	Result_ID	Ascending
ID		1
	Clustered:	False
	DistinctCount:	2
	Foreign:	False
	IgnoreNulls:	False
	Name:	ID
	Primary:	False
	Required:	False
	Unique:	False
	Fields:	
	ID	Ascending
ID_LIMS		1
	Clustered:	False
	DistinctCount:	2
	Foreign:	False
	IgnoreNulls:	False
	Name:	ID_LIMS
	Primary:	False
	Required:	False
	Unique:	False
	Fields:	
	ID_LIMS	Ascending
PrimaryKey		1
	Clustered:	False
	DistinctCount:	2
	Foreign:	False
	IgnoreNulls:	False
	Name:	PrimaryKey
	Primary:	True
	Required:	True
	Unique:	True

	Fields:	
	ID	Ascending
PrimaryKey		1
	Clustered:	False
	DistinctCount:	3
	Foreign:	False
	IgnoreNulls:	False
	Name:	PrimaryKey
	Primary:	True
	Required:	True
	Unique:	True
	Fields:	
	Protokoll ID	Ascending
ProtokollArt		1
	Clustered:	False
	DistinctCount:	1
	Foreign:	False
	IgnoreNulls:	False
	Name:	ProtokollArt
	Primary:	False
	Required:	False
	Unique:	False
	Fields:	
	Art	Ascending
GMO Nummer		1
	Clustered:	False
	DistinctCount:	15
	Foreign:	False
	IgnoreNulls:	False
	Name:	GMO Nummer
	Primary:	False
	Required:	False
	Unique:	False
	Fields:	
	GMO Nummer	Ascending
ID		1
	Clustered:	False
	DistinctCount:	28
	Foreign:	False
	IgnoreNulls:	False
	Name:	ID
	Primary:	False
	Required:	False
	Unique:	False
	Fields:	
	ID	Ascending
ID_Pflanzenarten_Ref		1
	Clustered:	False
	DistinctCount:	4
	Foreign:	False
	IgnoreNulls:	False
	Name:	ID_Pflanzenarten_Ref
	Primary:	False

	Required:	False
	Unique:	False
	Fields:	
	ID_Pflanzenarten_Ref	Ascending
PrimaryKey		1
	Clustered:	False
	DistinctCount:	28
	Foreign:	False
	IgnoreNulls:	False
	Name:	PrimaryKey
	Primary:	True
	Required:	True
	Unique:	True
	Fields:	
	ID	Ascending
ArbeitsgruppenPflanzentransformation		1
	Clustered:	False
	DistinctCount:	1
	Foreign:	True
	IgnoreNulls:	False
	Name:	ArbeitsgruppenPflanzentransformation
	Primary:	False
	Required:	False
	Unique:	False
	Fields:	
	AG	Ascending
Id Nummer		1
	Clustered:	False
	DistinctCount:	4
	Foreign:	False
	IgnoreNulls:	False
	Name:	Id Nummer
	Primary:	False
	Required:	False
	Unique:	False
	Fields:	
	GVO Nummer	Ascending
Number of rooted LInes		1
	Clustered:	False
	DistinctCount:	3
	Foreign:	False
	IgnoreNulls:	False
	Name:	Number of rooted LInes
	Primary:	False
	Required:	False
	Unique:	False
	Fields:	
	Number of rooted LInes	Ascending
Number of Shots		1
	Clustered:	False
	DistinctCount:	2
	Foreign:	False
	IgnoreNulls:	False

	Name:	Number of Shots
	Primary:	False
	Required:	False
	Unique:	False
	Fields:	
	Number of Shots	Ascending
OperatorPflanzentransformation		1
	Clustered:	False
	DistinctCount:	2
	Foreign:	True
	IgnoreNulls:	False
	Name:	OperatorPflanzentransformation
	Primary:	False
	Required:	False
	Unique:	False
	Fields:	
	Worker	Ascending
PrimaryKey		1
	Clustered:	False
	DistinctCount:	7
	Foreign:	False
	IgnoreNulls:	False
	Name:	PrimaryKey
	Primary:	True
	Required:	True
	Unique:	True
	Fields:	
	ID	Ascending
Result_ID		1
	Clustered:	False
	DistinctCount:	2
	Foreign:	False
	IgnoreNulls:	False
	Name:	Result_ID
	Primary:	False
	Required:	False
	Unique:	False
	Fields:	
	Result_ID	Ascending
Old_ID		1
	Clustered:	False
	DistinctCount:	4
	Foreign:	False
	IgnoreNulls:	False
	Name:	Old_ID
	Primary:	False
	Required:	False
	Unique:	False
	Fields:	
	Old_ID	Ascending
PrimaryKey		1
	Clustered:	False
	DistinctCount:	9

	Foreign:	False
	IgnoreNulls:	False
	Name:	PrimaryKey
	Primary:	True
	Required:	True
	Unique:	True
	Fields:	
	ID	Ascending
ID		1
	Clustered:	False
	DistinctCount:	12
	Foreign:	False
	IgnoreNulls:	False
	Name:	ID
	Primary:	False
	Required:	False
	Unique:	False
	Fields:	
	ID	Ascending
PrimaryKey		1
	Clustered:	False
	DistinctCount:	12
	Foreign:	False
	IgnoreNulls:	False
	Name:	PrimaryKey
	Primary:	True
	Required:	True
	Unique:	True
	Fields:	
	ID	Ascending

User Permissions

admin	Delete; Read Permissions; Set Permissions; Change Owner, Read Definition; Write Definition; Read Data; Insert Data; Update Data; Delete Data

Group Permissions

Admins	Delete; Read Permissions; Set Permissions; Change Owner, Read Definition; Write Definition; Read Data; Insert Data; Update Data; Delete Data
Users	Delete; Read Permissions; Set Permissions; Change Owner, Read Definition; Write Definition; Read Data; Insert Data; Update Data; Delete Data

Properties

DateCreated:	26.10.2009 16:10:28	DefaultView:	2
DisplayViewsOnSharePointSit	1	FilterOnLoad:	False
GUID:	{guid {0383D401-25B2-4A1B-917D-9F4FC82E182D}}	HideNewField:	False
LastUpdated:	26.10.2009 16:10:28	MaxRecords:	0
NameMap:	Long binary data	ODBCTimeout:	60
OrderByOn:	False	OrderByOnLoad:	True
Orientation:	Left-to-Right	RecordLocks:	No Locks
RecordsAffected:	0	ReturnsRecords:	True
TotalsRow:	False	Type:	0
Updatable:	True		

SQL

```
SELECT Medien.Medienname, Medien.[Medien ID], Medien.Storagecondition, Medien.Storagesite,
Medien.Bezeichner
FROM Medien
WHERE (((Medien.Medienname) Not Like "*obsolet*"))
ORDER BY Medien.Medienname;
```

Columns

Name	Type	Size
Medienname	Text	50

AggregateType:	-1
AllowZeroLength:	True
AppendOnly:	False
Attributes:	Variable Length; Updatable
CollatingOrder:	General
ColumnHidden:	False
ColumnOrder:	Default
ColumnWidth:	3615
DataUpdatable:	True
DisplayControl:	Text Box
GUID:	{guid {39A7EAC5-34A3-4BBD-A594-4E88A4399D39}}
IMEMode:	0
IMESentenceMode:	3
OrdinalPosition:	0
Required:	True
SourceField:	Medienname
SourceTable:	Medien
TextAlign:	General
UnicodeCompression:	True

Name	Type	Size
Medien ID	Long Integer	4

AggregateType:	-1
AllowZeroLength:	False
AppendOnly:	False
Attributes:	Fixed Size; Auto-Increment; Updatable
CollatingOrder:	General
ColumnHidden:	False
ColumnOrder:	1

ColumnWidth:	Default
DataUpdatable:	True
GUID:	{guid {FBE4AC72-E216-4CE4-ACB3-246BABE85369}}
OrdinalPosition:	1
Required:	False
SourceField:	Medien ID
SourceTable:	Medien
TextAlign:	General

Storagecondition Long Integer 4

AggregateType:	-1
AllowValueListEdits:	False
AllowZeroLength:	False
AppendOnly:	False
Attributes:	Fixed Size; Updatable
BoundColumn:	1
CollatingOrder:	General
ColumnCount:	2
ColumnHeads:	False
ColumnHidden:	False
ColumnOrder:	Default
ColumnWidth:	2070
ColumnWidths:	0;1440
DataUpdatable:	True
DecimalPlaces:	Auto
DisplayControl:	Combo Box
LimitToList:	True
ListRows:	8
ListWidth:	1440twip
OrdinalPosition:	2
Required:	False
RowSource:	SELECT [Storagecondition].[Storagecondition_ID], [Storagecondition].[Storagecondition_Name] FROM [Storagecondition] ORDER BY [Storagecondition_Name];
RowSourceType:	Table/Query
ShowOnlyRowSourceValues:	False
SourceField:	Storagecondition
SourceTable:	Medien
TextAlign:	General

Storagesite Long Integer 4

AggregateType:	-1
AllowValueListEdits:	False
AllowZeroLength:	False
AppendOnly:	False
Attributes:	Fixed Size; Updatable
BoundColumn:	1
CollatingOrder:	General
ColumnCount:	2
ColumnHeads:	False
ColumnHidden:	False
ColumnOrder:	Default
ColumnWidth:	2925
ColumnWidths:	0;1440

DataUpdatable:	True
DecimalPlaces:	Auto
DisplayControl:	Combo Box
LimitToList:	True
ListRows:	16
ListWidth:	1440twip
OrdinalPosition:	3
Required:	False
RowSource:	SELECT [Storagesite].[Storagesite_ID], [Storagesite].[Storagesite_Name] FROM [Storagesite] ORDER BY [Storagesite_Name];
RowSourceType:	Table/Query
ShowOnlyRowSourceValues:	False
SourceField:	Storagesite
SourceTable:	Medien
TextAlign:	General

Bezeichner OLE Object N/A

AggregateType:	-1
AllowZeroLength:	False
AppendOnly:	False
Attributes:	Variable Length; Updatable
CollatingOrder:	General
ColumnHidden:	False
ColumnOrder:	Default
ColumnWidth:	1965
DataUpdatable:	True
GUID:	{guid {A52CDB4F-F76D-43DE-B348-217A2AD512CB}}
OrdinalPosition:	4
Required:	False
SourceField:	Bezeichner
SourceTable:	Medien
TextAlign:	General

Table Indexes

Name	Number of Fields
Medien ID	1

Clustered:	False
DistinctCount:	36
Foreign:	False
IgnoreNulls:	False
Name:	Medien ID
Primary:	False
Required:	False
Unique:	False
Fields:	
Medien ID	Ascending

Name	Number of Fields
Medien ID old	1

Clustered:	False
DistinctCount:	11
Foreign:	False
IgnoreNulls:	False

	Name:	Medien ID old
	Primary:	False
	Required:	False
	Unique:	False
	Fields:	
	Medien ID old	Ascending
Medienname		1
	Clustered:	False
	DistinctCount:	36
	Foreign:	False
	IgnoreNulls:	False
	Name:	Medienname
	Primary:	False
	Required:	False
	Unique:	True
	Fields:	
	Medienname	Ascending
PrimaryKey		1
	Clustered:	False
	DistinctCount:	36
	Foreign:	False
	IgnoreNulls:	False
	Name:	PrimaryKey
	Primary:	True
	Required:	True
	Unique:	True
	Fields:	
	Medien ID	Ascending

User Permissions

admin	Delete; Read Permissions; Set Permissions; Change Owner, Read Definition; Write Definition; Read Data; Insert Data; Update Data; Delete Data

Group Permissions

Admins	Delete; Read Permissions; Set Permissions; Change Owner, Read Definition; Write Definition; Read Data; Insert Data; Update Data; Delete Data
Users	Delete; Read Permissions; Set Permissions; Change Owner, Read Definition; Write Definition; Read Data; Insert Data; Update Data; Delete Data
